# H-Bond donor parameters for cations†
†Electronic supplementary information (ESI) available: Titration data and details of fitting the binding isotherm. See DOI: 10.1039/c9sc00721k


**DOI:** 10.1039/c9sc00721k

**Published:** 2019-05-16

**Authors:** Sarah J. Pike, Ennio Lavagnini, Lisa M. Varley, Joanne L. Cook, Christopher A. Hunter

**Affiliations:** a Department of Chemistry , University of Cambridge , Lensfield Road , Cambridge , CB2 1EW , UK . Email: herchelsmith.orgchem@ch.cam.ac.uk; b Department of Chemistry , University of Sheffield , Sheffield , S3 7HF , UK; c Unilever R&D Port Sunlight , Quarry Road East , Bebington , Wirral CH63 3JW , UK

## Abstract

UV/Vis absorption and NMR spectroscopy titrations have been used to investigate the formation of complexes between cations and neutral H-bond acceptors in organic solvents. Complexes formed by two different H-bond acceptors with fifteen different cations were studied in acetone and in acetonitrile. The effects of water and ion pairing with the counter-anion were found to be negligible in the two polar solvents employed for this study. The data were used to determine self-consistent H-bond donor parameters (*α*) for a series of organic and inorganic cations; guanidinium, primary, tertiary and quaternary ammonium, imidazolium, methylpyridinium, lithium, sodium, potassium, rubidium and caesium. The results demonstrate the transferability of *α* parameters for cations between different solvents and different H-bond acceptor partners, allowing reliable prediction of cation recognition properties in different environments. Lithium and protonated nitrogen cations form the most stable complexes, but the *α* parameter is only 5.0, which is similar to the neutral H-bond donor 3-trifluoromethyl,4-nitrophenol (*α* = 5.1). Quaternary ammonium is the weakest H-bond donor investigated with an *α* value of 2.7, which is comparable to an alcohol. The *α* parameters for alkali metal cations decrease down the group from 5.0 (Li^+^) to 3.5 (Cs^+^).

## Abstract 2

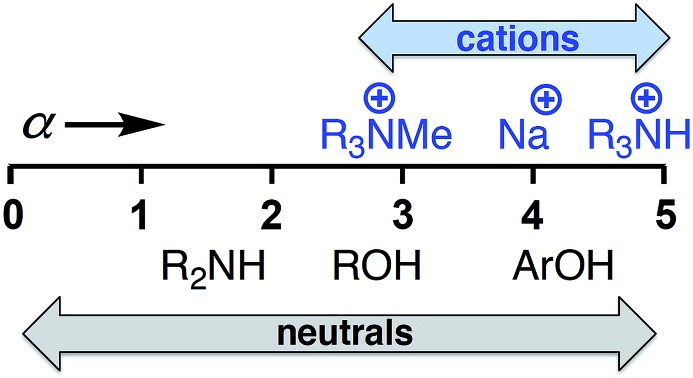
Parameters that provide a quantitative description of the free energy of interaction of cations with any H-bond acceptor in any solvent have been experimentally determined.

## Introduction

H-bonding interactions with cations play an important role in bimolecular recognition.^1^,^2^ In synthetic systems, H-bonding interactions to cations^3^ have found applications in a number of fields including organocatalysis,^4^ crystal engineering,^5^,^6^ materials chemistry,^7^ receptors^8^,^9^ and ion sensing for use in clinical diagnostics and environmental monitoring.^10^ However, the development of a quantitative understanding of the factors that govern the thermodynamic properties of this important class of non-covalent interactions in solution is still required to use them in rational design of new supramolecular systems.

**Fig. 1:**
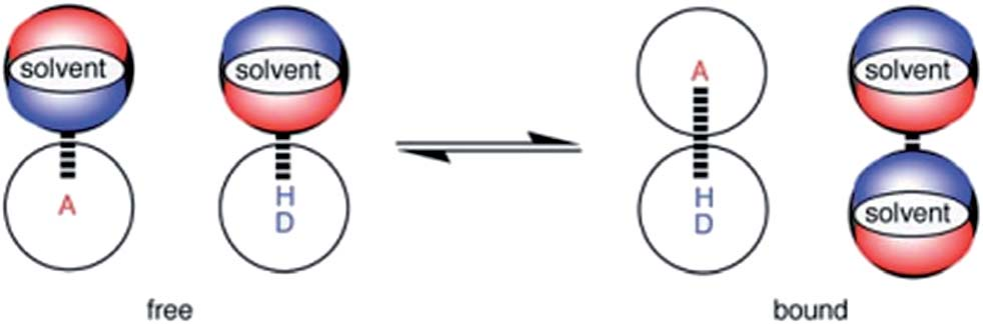
The solvent competition model for the formation of a H-bonded complex between two solutes. The position of equilibrium is determined by the energies of the solute–solvent interactions in the free state, and the solute–solute and solvent–solvent interactions in the bound state. A represents a H-bond acceptor solute and DH a H-bond donor solute.

Quantitative scales that describe the H-bond acceptor (HBA) and H-bond donor (HBD) properties of a wide variety of neutral organic functional groups have been developed by Abraham.^11^ Experimentally determined association constants (*K*) for simple complexes that form a single H-bond in non-polar solvents are the basis upon which the scales were established.^11^–^14^ To develop a universal H-bonding scale, Hunter extended this method to address the influence of solvent on solution phase equilibria between H-bonded solutes. The solvent competition model illustrated in Fig. 1 treats solution phase H-bonding interactions as an equilibrium between pairwise contacts between solvent and solute.^15^

The H-bond donor and acceptor parameters for the solute (*α* and *β*) and the solvent (*α*_s_ and *β*_s_) can be used in eqn (1) to predict the Gibbs free energy change (Δ*G*°) for formation of a H-bonded complex in any solvent.^16^

1 Δ*G*°/kJ mol^–1^ = –(*α* – *α*_s_)(*β* – *β*_s_) + 6

where the adverse free energy associated with formation of a bimolecular complex in solution has been experimentally determined to be 6 kJ mol^–1^ in carbon tetrachloride and is assumed to be a constant in other solvents.

Experimentally measured association constants for H-bond formation (*K*) can be used in conjunction with eqn (1) to determine H-bond parameters for solutes or solvents.^15^–^20^ For example, the *α* value of a HBD can be determined through the rearrangement of eqn (1) to give eqn (2), when the *β*, *α*_s_ and *β*_s_ parameters are known.

2 *α* = *α*_s_ + (*RT* ln *K* + 6)/(*β* – *β*_s_)

This approach has been employed to quantify the H-bond properties of neutral organic functional groups,^21^–^23^ and we have recently demonstrated that charged species can be placed on the same H-bonding scale through the determination of *β* values for a range of anions.^19^ Here, the same methodology is used to place cations on the H-bond scale through the experimental determination of *α* values.

To date, there have been few systematic studies of non-covalent interactions with cations. Marcus investigated the solvation properties of cations using linear solvation energy relationships.^24^ The thermodynamics of phase transfer of ions from water to organic solvents was used to determine empirical parameters to describe the solvation properties of ions.^25^ Gilkerson used conductance experiments and UV/Vis absorption titrations to study interactions between cations and HBAs in organic solvents.^26^–^30^ The relative HBD strength of the cations was found to decrease in the order protonated amine > alkali metal cation > quaternary ammonium.28*a* Conductance experiments were also used to measure association constants (*K*) for 1 : 1 complexes formed between triethylammonium picrate and a series of pyridines in nitrobenzene. A linear correlation was found between the value of log *K* and the corresponding Hammett parameter for the substituent on the H-bond acceptor.^31^,^32^

In this paper, we report experiments that establish *α* H-bond donor parameters for a number of inorganic and organic monovalent cations by using the results of titration experiments conducted in two different solvents with two different HBAs in conjunction with eqn (2). The metal ions used in these experiments are not H-bond donors, but as we will show, it is possible to provide a quantitative description of the stabilities of the complexes formed with H-bond acceptors using the same *α* parameter scale that is used to describe the non-covalent interaction properties of H-bond donors.

## Results and discussion

**Scheme 1:**
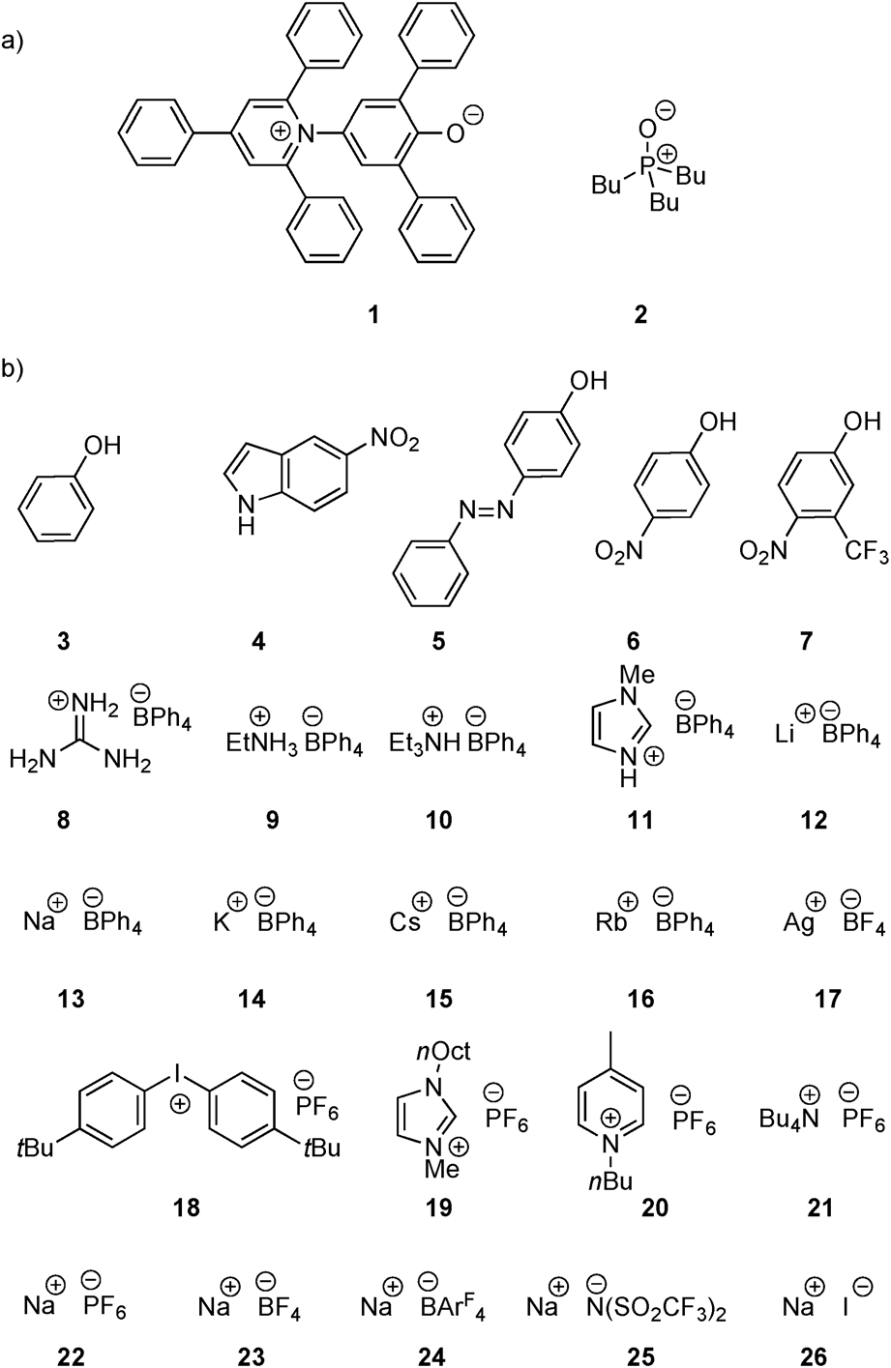
(a) H-Bond acceptors and (b) cations (Ar^F^ = 3,5-bis(trifluoromethyl)phenyl, R = 2-ethylhexyl, **12** is the tris(1,2-dimethoxyethane) adduct).

**Scheme 2:**
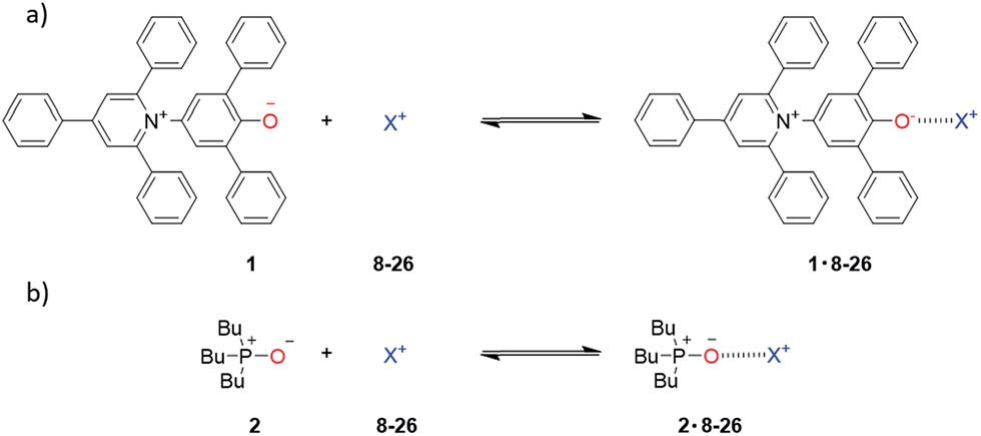
Formation of H-bonded complexes between HBAs and cations.

Two H-bond acceptors with different HBA properties, Reichardt's dye^33^ (**1***β* = 14.0) and tri-*n*-butylphosphine oxide (**2***β* = 10.7), were selected to study the formation of H-bonded complexes with cations (Scheme 1a). Both HBAs have sufficiently high *β* values to form stable complexes in the competitive polar solvents required to dissolve the salts and have spectroscopic properties that are sensitive to H-bond formation (Scheme 2). Reichardt's dye, **1**, has a strong UV/Vis absorption band, and formation of H-bonds is associated with a significant hypsochromic shift.^34^ Formation of H-bonds with phosphine oxide **2** is detected *via* the associated increase in the ^31^P NMR chemical shift.

Twenty different salts were used in this study providing variation in both the counter-anion and the cation (Scheme 1b). Weakly coordinating anions, tetraphenylborate, hexafluorophosphate and tetrafluoroborate, were used as counterions to minimize competing interactions with the formation of H-bonded complexes. The influence of the counterion on the stabilities of the complexes was investigated by studying Na^+^ salts with six different anions. The influence of the water content of the solvent on the association constant was also studied for a subset of the cation complexes. Salts **8–11** were prepared from the corresponding hydrochloride salt using an anion exchange reaction with NaBPh_4_ in water.^26^,^35^,^36^ The other salts (**12–26**) are commercially available.

A number of additional titration experiments were carried out to extend the range of cations, but the results proved unsuitable for accurate determination of *α* parameters. For example, association constants were too high to be measured for the iodide salts of Ca^2+^ and Mg^2+^ and the tetrafluoroborate salts of Pd^2+^, Fe^2+^ and Cu^+^. The tetrafluoroborate salt of methyl(diphenyl)sulfonium caused methylation of **1** and did not bind strongly enough to **2** to measure association constants. The hexafluorophosphate salt of the trityl cation caused disappearance of the UV/Vis absorption of **1** and gave an anomalously large change in the ^31^P chemical shift of **2**, suggesting that the interactions are covalent in nature.

Two different solvents were used for the titration experiments: acetonitrile and acetone. Acetonitrile is a stronger H-bond donor but significantly weaker H-bond acceptor than acetone,^15^ and the two solvents have different dielectric constants (37.5 for acetonitrile, and 20.7 for acetone). The original neutral H-bond scales were established based on association constants measured in carbon tetrachloride and 1,1,1-trichloroethane,^11^–^13^ so in order to establish transferability between solvents the complexes formed by **1** and **2** with a series of neutral HBDs (**3–7** in Scheme 1b) were studied in four solvents, carbon tetrachloride, chloroform, acetonitrile and acetone.^21^,^22^ The neutral HBDs (**3–7**) all have a UV/Vis absorption band that is sensitive to H-bond formation, so for the titrations with **2**, these compounds were used as hosts in UV/Vis absorption titration experiments.

### Neutral HBD complexes

To establish a set of self-consistent of solvent H-bond parameters that can be used for the cation experiments, the association constants for complexes formed by **1** and **2** with a series of neutral HBD, **3–7**, were measured in carbon tetrachloride, chloroform, acetonitrile and acetone and analysed using eqn (1). In cases where the same association constant could be measured using both UV/Vis absorption and NMR spectroscopy, the results were comparable. For example, **2·6** has association constants of 190 M^–1^ (UV/Vis) and 240 M^–1^ (NMR) in chloroform, 48 M^–1^ (UV/Vis) and 58 M^–1^ (NMR) in acetone, 69 M^–1^ (UV/Vis) and 80 M^–1^ (NMR) in acetonitrile (see ESI†). The average association constants are given in Table 1. For **1**, protonation occurred with **6** and **7** in carbon tetrachloride.

**Table 1:** Association constants (*K*/M^–1^) for neutral complexes measured by UV/Vis absorption and ^31^P NMR titration experiments at 298 K in different solvents^*a*^

HBD	HBA	MeCN	Acetone	CHCl_3_	CCl_4_
**3**	**1**	260 ± 63	340 ± 6	260 ± 55	—^*c*^
**4**	**1**	200 ± 50	860 ± 350	1200 ± 500	—^*c*^
**5**	**1**	3100 ± 650	2000 ± 500	4000 ± 700	—^*c*^
**3**	**2**	20 ± 1	26 ± 1	47 ± 24^*d*^	1860 ± 226^*d*^
**4**	**2**	6 ± 1	11 ± 1	18 ± 6^*e*^	1280 ± 226^*d*^
**5**	**2**	63 ± 18^*d*^	64 ± 45^*d*^	217 ± 66^*d*^	11 775 ± 7280^*d*^
**6**	**2**	106 ± 11^*d*^	134 ± 18	1410 ± 23^*d*^	120 300 ± 61 376^*d*^
**7**	**2**	283 ± 48^*d*^	259 ± 80	5400 ± 42	—^*b*^

^*a*^Average of at least two titrations. Errors are quoted at the 95% confidence limit.

^*b*^Association constant too high to be measured using UV/Vis spectroscopy.

^*c*^Poor solubility in carbon tetrachloride prevented the acquisition of data.

^*d*^Average of *K* values obtained from both ^31^P NMR and UV/Vis spectroscopy data spectroscopy titrations.

^*e*^

**Fig. 2:**
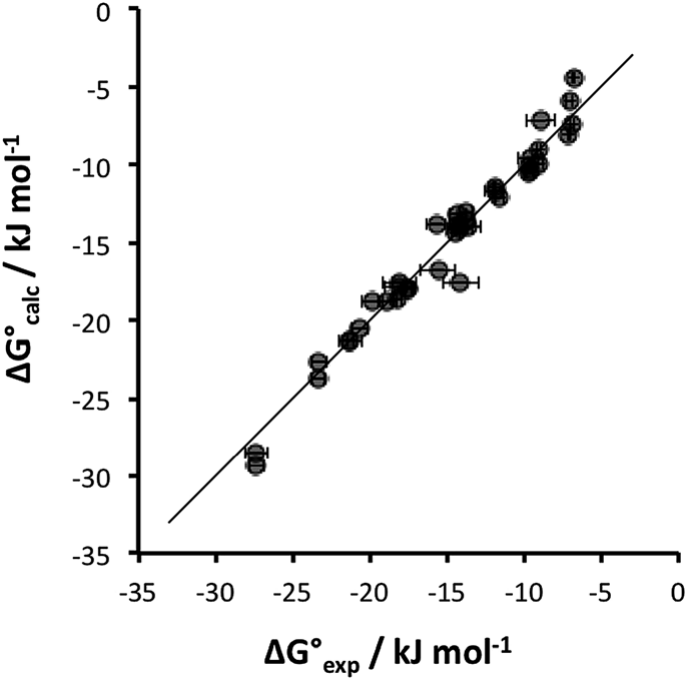
Comparison of experimental free energies of complexation 
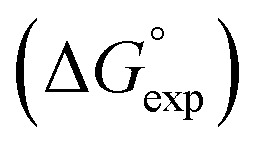
 with values calculated using eqn (1)
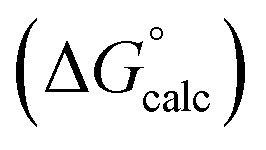
 for complexes formed by **1** and **2** with neutral HBDs in carbon tetrachloride, chloroform, acetonitrile and acetone. The line represents 
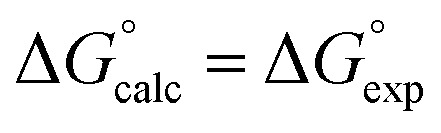
.

Some of the relevant H-bond parameters are available in the literature,^15^–^21^ and the other H-bond parameters were optimized to obtain the best fit of the experimental association constants to eqn (1). The resulting H-bond parameters are shown in Table 2. Fig. 2 shows that eqn (1) provides an excellent description of the association constants shown in Table 1, if the H-bond parameters in Table 2 are used.

**Table 2:** H-bond parameters for neutral solutes and solvents

	*β*	*α*	*α*_s_	*β*_s_
**1**	14.0	—	—	—
**2**	10.7^*a*^	—	—	—
**3**	—	3.9^*b*^	—	—
**4**	—	3.8	—	—
**5**	—	4.3^*a*^	—	—
**6**	—	4.7^*a*^	—	—
**7**	—	5.1^*a*^	—	—
CCl_4_	—	—	1.4^*b*^	0.6^*b*^
MeCN	—	—	1.5^*a*^	5.1^*a*^
Acetone	—	—	1.2	5.7^*c*^
CHCl_3_	—	—	2.2^*a*^	1.3^*a*^

^*a*^Value in ref. 17.

^*b*^Value in ref. 15.

^*c*^Value in ref. 37.

### Cation HBD complexes

**Fig. 3:**
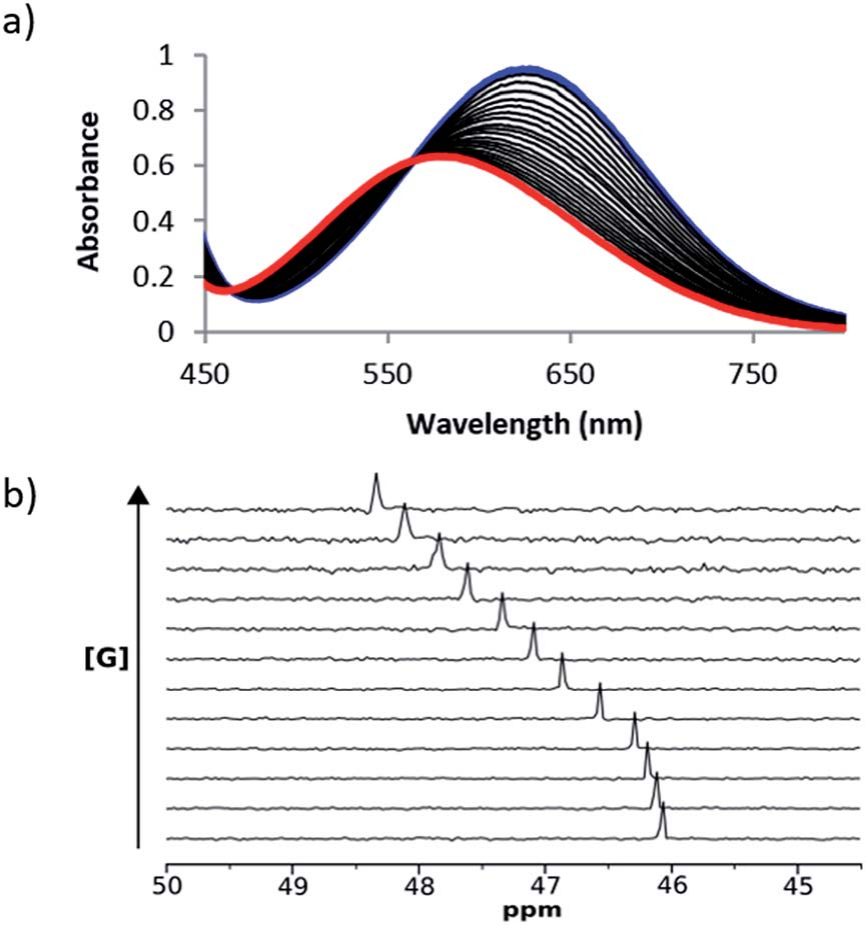
(a) UV/Vis absorption spectra for titration of **24** into **1** (0.2 mM) in acetonitrile at 298 K. The initial spectrum of unbound **1** is shown in blue, and the final spectrum corresponding to the bound complex **1·24** is shown in red. (b) 162 MHz ^31^P NMR spectra for titration of **24** into **2** (6 mM) in acetonitrile at 298 K.

UV/Vis absorption and ^31^P NMR titration experiments were performed with **1** and **2** respectively for all of the cations in Scheme 1 in acetonitrile and in acetone. Fig. 3 shows representative titration data. Addition of increasing quantities of salt to **1** leads to a hypsochromic shift in the UV/Vis absorption band: for example on formation of the **1·24** complex, the UV/Vis absorption maximum moved from 636 nm to 482 nm in acetonitrile and from 676 nm to 520 nm in acetone (Fig. 3a). The magnitude of the complexation-induced change in ^31^P NMR chemical shift for the complexes formed with **2** was dependent on both the solvent and the salt. For example, the upfield shift observed for formation of the **2·24** complex was 3.0 ppm in acetonitrile and 4.5 ppm in acetone (Fig. 3b). The titration data fit well to either a 1 : 1 binding isotherm^19^ or a 1 : 1 binding isotherm that allowed for a second very weak interaction,^16^ and the resulting association constants are shown in Table 3. Protonation of **1** was observed on addition of protonated amines (**9–11**), so association constants are not reported for these complexes (see ESI†).

**Table 3:** Association constants (*K*/M^–1^) for 1 : 1 complexes formed with cations measured by UV/Vis absorption and ^31^P NMR titration experiments at 298 K^*a*^

Anion	Cation	HBD	HBA/solvent			
			**1**		**2**	
			MeCN	Acetone	MeCN	Acetone
BPh_4_^–^	Guanidinium	**8**	33 800 ± 2000	—^*b*^	198 ± 61	225 ± 40
BPh_4_^–^	2-Ethylhexyl ammonium	**9**	—^*c*^	—^*c*^	147 ± 25	123 ± 34
BPh_4_^–^	Triethyl ammonium	**10**	—^*c*^	—^*c*^	85 ± 6	203 ± 50
BPh_4_^–^	*N*-Methyl imidazolium	**11**	—^*c*^	—^*c*^	102 ± 12	156 ± 18
BPh_4_^–^	Li^+^	^*d*^	27 400 ± 5900	—^*b*^	—^*b*^	—
BPh_4_^–^	Na^+^	**13**	390 ± 51	1700 ± 160	—^*e*^	73 ± 5
BPh_4_^–^	K^+^	**14**	—^*e*^	270 ± 75	—^*e*^	13 ± 3
BPh_4_^–^	Rb^+^	**15**	—^*e*^	220 ± 50	—^*e*^	—^*e*^
BPh_4_^–^	Cs^+^	**16**	—^*e*^	210 ± 35	—^*e*^	—^*e*^
BF_4_^–^	Ag^+^	**17**	—^*f*^	—^*b*^	8 ± 1	966 ± 40
PF_6_^–^	^+^I(4-^*t*^BuPh)_2_	**18**	38 300 ± 1100	48 000 ± 3000	62 ± 3	83 ± 12
PF_6_^–^	MOIM^+^	**19**	23 ± 9	200 ± 60	—^*e*^	—^*e*^
PF_6_^–^	*N*-Butyl-4-methyl pyridinium	**20**	12 ± 2	180 ± 40	—^*e*^	—^*e*^
PF_6_^–^	Tetra(*n*-butyl) ammonium	**21**	—^*e*^	10 ± 2	—^*e*^	—^*e*^
PF_6_^–^	Na^+^	**22**	391 ± 35	1700 ± 140	—^*e*^	55 ± 13
BF_4_^–^	Na^+^	**23**	300 ± 90	1900 ± 100	57 ± 6	57 ± 2
BAr^F^_4_^–^	Na^+^	**24**	400 ± 160	1400 ± 400	53 ± 3	72 ± 2
N(SO_2_CF_3_)_2_^–^	Na^+^	**25**	410 ± 30	1500 ± 200	56 ± 5	78 ± 12
I^–^	Na^+^	**26**	320 ± 120	1000 ± 300	45 ± 1	59 ± 6

^*a*^Average of at least two titrations. Errors are quoted at the 95% confidence limit. Unless otherwise stated greater than 50% saturation of the binding isotherm was achieved.

^*b*^Association constant was too high to be measured using UV/Vis spectroscopy.

^*c*^Protonation of the H-bond acceptor was observed upon addition of guest.

^*d*^Li salt is LiBPh_4_ tris(1,2-dimethyoxyethane).

^*e*^Association constant was too low to be measured, because the salt was not sufficiently soluble to obtain 50% saturation of the binding isotherm.

^*f*^Poor fit to a 1 : 1 binding isotherm.

The association constants measured for the complexes span four orders of magnitude (Table 3). The relative polarities of the solvents and solutes determine the stabilities (Table 2). The complexes formed with **1** are generally more stable than the corresponding complexes formed with **2**, which is in agreement with the H-bond parameters of the two acceptors (*β* = 14.0 and 10.7 respectively). The differences in association constant are most significant in acetone, where the complexes formed with **1** are up to three orders of magnitude more stable than corresponding complexes formed with **2**. For the weaker HBA **2**, the association constants for the complexes formed with **14–16**, **19** and **21–23** were too low to be measured in acetonitrile. For stronger HBA **1**, the association constants for the complexes formed with **6–8**, **12** and **17** were too high to be measured in acetone.

**Fig. 4:**
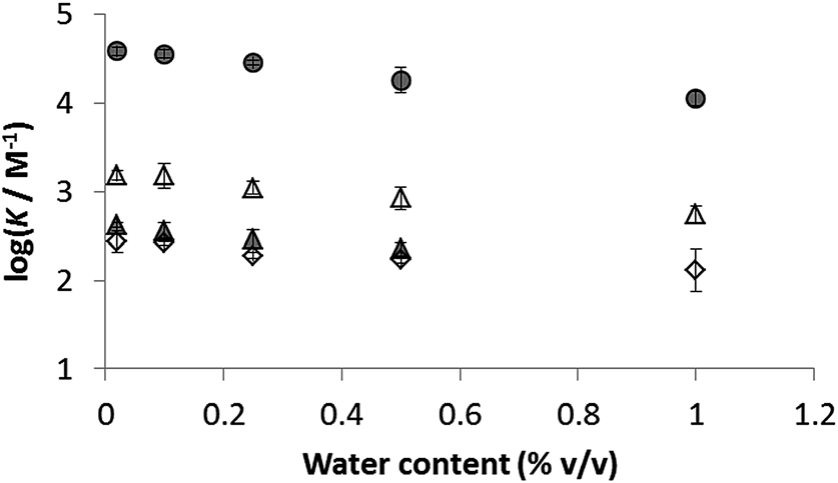
Effect of added water on association constants measured in acetonitrile (shaded) and acetone (open): **1·8** circles, **1·26** triangles and **1·14** diamonds. The water content of both solvents without addition of water is 0.02%.

The presence of traces of water might perturb the measured values of the association constants reported in Table 3. The water content of acetonitrile and acetone used in these experiments was determined to be 0.02% in both cases.^38^ To establish whether these quantities of water can influence the stabilities of the complexes and hence perturb the measured association constants, water was deliberately added to the stock solutions of both solvents, and the titration experiments were repeated for a subset of the complexes. The influence of added water on the association constants measured in acetonitrile and in acetone is illustrated in Fig. 4. Large quantities of water are required to have a significant effect in either solvent, so we conclude that the traces of water present in the titration experiments do not affect the values of the association constants in Table 3.

### H-Bond donor parameters

The association constants in Table 3 can be used in eqn (2) with the H-bond parameters from Table 2 to determine *α* values for the cations in salts **8–26**. Table 4 shows the values of *α* derived from each of the experimentally determined association constants in Table 3. There is generally good agreement for *α* values measured with different HBAs in different solvents and with different counterions. For example, the three measurements for the guanidinium cations are all 5.0 ± 0.1. The only major discrepancy observed is for the silver cation: only two association constants were measured for this cation, and the resulting *α* values are very different.

**Table 4:** *α* values for cations^*a*^

Anion	Cation	HBD	HBD/solvent			
			**1**		**2**	
			MeCN	Acetone	MeCN	Acetone
BPh_4_^–^	Guanidinium^+^	**8**	5.0 ± 0.1	—^*b*^	4.9 ± 0.1	5.1 ± 0.1
BPh_4_^–^	2-Ethylhexyl ammonium	**9**	—^*b*^	—^*b*^	4.8 ± 0.1	4.8 ± 0.2
BPh_4_^–^	Triethyl ammonium	**10**	—^*b*^	—^*b*^	4.5 ± 0.1	5.0 ± 0.1
BPh_4_^–^	*N*-Methyl imidazolium^+^	**11**	—^*b*^	—^*b*^	4.6 ± 0.1	4.9 ± 0.1
BPh_4_^–^	Li^+^	**12**	5.0 ± 0.2	—^*b*^	—^*b*^	—^*b*^
BPh_4_^–^	Na^+^	**13**	3.8 ± 0.1	4.1 ± 0.1	—^*b*^	4.5 ± 0.1
BPh_4_^–^	K^+^	**14**	—^*b*^	3.6 ± 0.1	—^*b*^	3.7 ± 0.1
BPh_4_^–^	Rb^+^	**15**	—^*b*^	3.5 ± 0.1	—^*b*^	—^*b*^
BPh_4_^–^	Cs^+^	**16**	—^*b*^	3.5 ± 0.1	—^*b*^	—^*b*^
BF_4_^–^	Ag^+^	**17**	—^*b*^	—^*b*^	3.5 ± 0.1	5.8 ± 0.1
PF_6_^–^	^+^I(4-^*t*^BuPh)_2_	**18**	5.1 ± 0.1	5.1 ± 0.1	4.4 ± 0.1	4.6 ± 0.1
PF_6_^–^	MOIM^+^	**19**	3.0 ± 0.4	3.5 ± 0.1	—^*b*^	—^*b*^
PF_6_^–^	*N*-Butyl-4-methyl pyridinium	**20**	2.9 ± 0.1	3.5 ± 0.1	—^*b*^	—^*b*^
PF_6_^–^	Tetra(*n*-butyl) ammonium	**21**	—^*b*^	2.6 ± 0.1	—^*b*^	—^*b*^
PF_6_^–^	Na^+^	**22**	3.8 ± 0.1	4.1 ± 0.1	—^*b*^	4.4 ± 0.2
BF_4_^–^	Na^+^	**23**	3.8 ± 0.1	4.2 ± 0.1	4.4 ± 0.1	4.4 ± 0.1
BAr^F–^	Na^+^	**24**	3.8 ± 0.2	4.1 ± 0.3	4.3 ± 0.1	4.5 ± 0.1
N(SO_2_CF_3_)_2_^–^	Na^+^	**25**	3.8 ± 0.1	4.1 ± 0.2	4.4 ± 0.1	4.6 ± 0.2
I^–^	Na^+^	**26**	3.8 ± 0.2	4.0 ± 0.2	4.3 ± 0.1	4.4 ± 0.1

^*a*^Errors at the 95% confidence limit.

^*b*^No experimental data available.

The type of ion pair and/or aggregate formed by salts in solution is highly dependent on both the nature of the counterion and the polarity of the solvent.^39^–^41^ The dielectric constant of a solvent is known to be inversely proportional to the association constant for the ion pairing of the salt^42^ and in solvents with high dielectric constants the presence of loose ion pairs (solvent-shared ion pairs wherein oppositely charged species are separated by one layer of solvent molecules and solvent-separated ion pairs which have more than one layer of solvent separating the oppositely charged species) are known to exist.^40^ Contact ion pairs which involve direct interaction between oppositely charged species and higher aggregates of ions are generally found in solvents of low dielectric constant and at high salt concentrations.^41^ Acetonitrile and acetone have high dielectric constants of 37.5 and 20.7 respectively.^42^ In both solvents loose ion pairs are mostly likely to dominate, either as solvent-separated or solvent-shared, whilst the presence of contact ion-pairs (and aggregates) would not be expected to be significant.^40^

To establish whether ion pairing (or aggregation) of the salt has a significant effect on the H-bond parameters reported in Table 4, six different counter-anions were used for the sodium cation; tetraphenylborate (**13**), hexafluorophosphate (**23**), tetrafluoroborate (**24**), tetrakis(3,5-bis(trifluoromethyl)phenyl)borate (**25**), trifluoromethanesulfonimide (**26**) and iodide (**27**) (Scheme 1). While most of the anions are weakly coordinating, iodide is a relatively strongly coordinating counterion. The HBA parameters (*β*) of three of the counter-anions have previously been determined:19*a* hexafluorophosphate (7.0) trifluoromethanesulfonimide (7.3) and iodide (8.9).19*a* Accordingly, the influence of the strength of the H-bond acceptor parameter of the counterion on the interaction of Na^+^ with HBAs **1** and **2** was investigated. Although there are some variations in the value of *α* measured for Na^+^ with different HBAs and solvents, the values for different counterions are practically identical in all cases. This result suggests that the values reported in Table 4 are not significantly perturbed by interactions with the counter-anion.

**Fig. 5:**
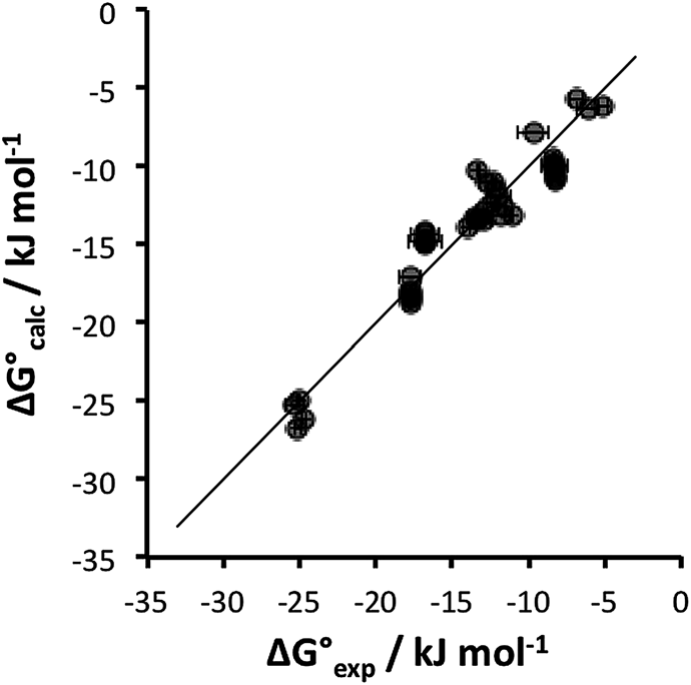
Comparison of experimental free energies of complexation 
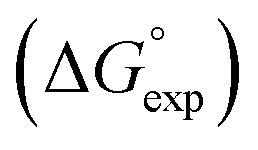
 with values calculated using eqn (1)
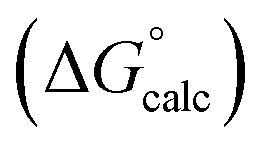
 for 1 : 1 complexes formed with cations. The line represents 
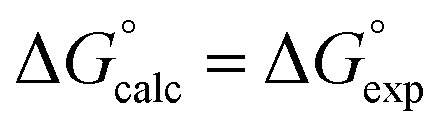
.

Table 4 shows that the analysis described here results in a range of different *α* parameters for the same cation. For example, the 22 independent measurements of the value *α* for Na^+^ fall between 3.8 and 4.6. This variation between individual measurements of *α* for the same cation reflects the magnitude of the errors associated with the approach. However, it is possible to describe the all of the experimental data by using a single representative value of *α* for each cation. Fig. 5 shows the result of optimising a single *α* parameter for each cation to fit the experimental association constants in Table 3. The calculated free energies of complexation in Fig. 5 agree well with the experimental data. The rmsd between the experimental and calculated values is 1.5 kJ mol^–1^, which provides an indicator of the error associated with the spread of values of the individual measurements of *α* for each cation in Table 4. The optimised cation *α* values are reported in Table 5. These *α* parameters can be used to describe the interactions of cations with different HBAs in different solvent environments and with different counterions.

**Table 5:** Consensus *α* parameters for cations

Cation	*α*
Guanidinium	5.0
2-Ethylhexyl ammonium	4.8
Triethyl ammonium	4.8
*N*-Methyl imidazolium	4.7
Li^+^	5.0
Na^+^	4.1
K^+^	3.6
Rb^+^	3.5
Cs^+^	3.5
^+^I(4-^*t*^BuPh)_2_	4.9
MOIM^+^	3.3
*N*-Butyl-4-methyl pyridinium	3.5
Tetra(*n*-butyl)ammonium	2.7

**Fig. 6:**
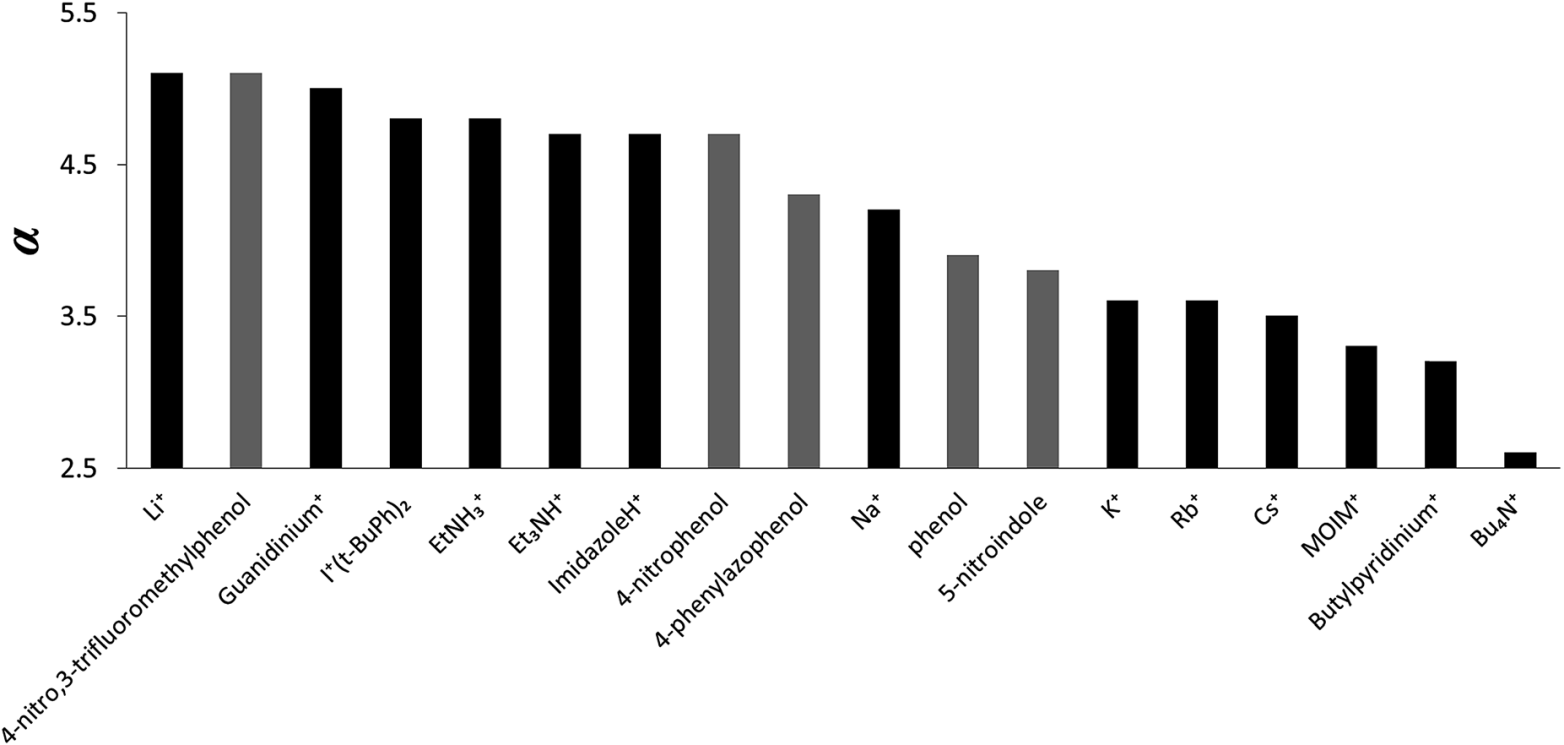
*α* values for cations (the neutral donors are shown in grey for comparison).

The *α* parameters measured for the cations are illustrated graphically in Fig. 6. Lithium and guanidinium form the most stable complexes, but the value of *α* is only 5.0, which is similar to the most polar neutral HBDs, *e.g.* 4-nitro,3-trifluoromethyl-phenol (*α* = 5.1). The HBD properties of protonated nitrogen donors are all very similar and are comparable to hexafluoropropan-2-ol (*α* = 4.7–4.8).^15^ The *α* parameters for group 1 metal cations decrease in strength upon progression down the group. Sodium has properties similar to phosphoric acid (*α* = 4.1), and potassium, rubidium and caesium are similar to a carboxylic acid (*α* = 3.6).^15^ For the organic cations, changing the nature of donor from the NH in protonated amines to the CH in quaternary ammonium cations reduces *α* from 4.7–5.0 to 2.7–3.5. Tetrabutyl ammonium is the weakest HBD (*α* = 2.7), but the HBD properties are still significant, comparable to those of an alcohol (*α* = 2.7).^15^

## Conclusions

UV/Vis absorption and NMR titrations have been employed to study the complexes formed between fifteen different cations and two different H-bond acceptors in acetonitrile and acetone. In the polar solvents employed for this study, neither ion pairing nor small amounts of water compete with complex formation. With the exception of the results obtained for the silver cation, the experimental data conform to Hunter's solvent competition model, allowing the H-bond donor parameters (*α*) for the cations to be determined. The results demonstrate the transferability of *α* parameters for cations between different solvents and different H-bond acceptor partners, allowing reliable prediction of cation recognition properties in different solvent environments.

Measurement of the *α* parameters for cations does not extend the *α* scale beyond the values measured for neutral functional groups. The highest *α* values are found for lithium and protonated nitrogens, with values similar to the strongest neutral H-bond donors (*α* ≈ 5).^30^ The variation in *α* values measured for alkali metal cations shows that upon progression down the group, the interaction properties of the cation are reduced. In organic cations, the nature of the donor heavy atom is important, so protonated amines have significantly higher HBD parameters than the CH donor in quaternary ammonium cations. These new H-donor parameters for cations will be useful in the development of our understanding and prediction of the behaviour of charged species in organic solution with applications in the design of new supramolecular systems.

## Experimental section

### General experimental procedure

All compounds were purchased from Sigma-Aldrich unless otherwise stated. Acetonitrile, acetone and chloroform were purchased from Acros as 99+% for spectroscopic grade and were used as received. All NMR spectroscopy was carried out on a Bruker AVI400 spectrometer using the residual solvent as the internal standard. All the chemical shifts (*δ*) are quoted in ppm and coupling constants are given in Hz. Splitting patterns are given as follows: s (singlet), br (broad), d (doublet), q (quartet), t (triplet), m (multiplet). Melting points were measured in a Mettler Toledo MP50 Melting Point System. ES+ was carried out on a Waters LCT-TOF spectrometer or a Waters Xevo G2-S bench top QTOF machine. All compounds were used as received. The measurements of solids were carried out on a Precisa 125A balance.

### Guanidinium tetraphenylborate (**8**)

To a solution of guanidinium hydrochloride (0.028 g, 0.29 mmol) in H_2_O (5 mL) was added a solution of sodium tetraphenylborate (100 mg, 0.29 mmol) in H_2_O (5 mL) and the resultant solution was left stirring at room temperature for 10 min. The white precipitate that had formed was filtered off, washed with H_2_O (3 × 10 mL) and dried to give the desired compound as a white solid. (105 mg, 95%) m.p. 220–223 °C; ^1^H NMR (400 MHz, CD_3_CN, 298 K): *δ* 7.30–7.25 (8H, m, Ar*H*), 7.00 (8H, t, 4*J* = 8.0 Hz, Ar*H*), 6.87–6.82 (4H, m, Ar*H*), 5.92 (6H, s, br, 2 × NH_2_ and ^+^NH_2_); ^13^C NMR (100 MHz, CD_3_CN, 298 K): *δ* 164.8 (q), 158.8, 136.7 (q), 126.6 (q), 122.8; MS (ESI^+^): *m*/*z* (%): 60(100); found: M, 60.0559, C_1_H_6_N_3_ requires 60.0556.

### Triethylammonium tetraphenylborate (**9**)

To a solution of triethylamine hydrochloride (0.040 g, 0.29 mmol) in H_2_O (5 mL) was added a solution of sodium tetraphenylborate (0.10 g, 0.29 mmol) in H_2_O (5 mL). The reaction mixture was left stirring for 10 min at ambient temperature and the white precipitate that had formed was filtered off, collected, washed with H_2_O (3 × 10 mL) and dried to yield the desired product as a white solid (0.12 g, 93%). m.p. 182–184 °C; ^1^H NMR (400 MHz, (CD_3_)_2_SO, 298 K): *δ* 8.79 (1H, s, br, N*H*^+^), 7.16–7.11 (8H, m, Ar*H*), 6.88 (8H, t, *J* = 8 Hz, Ar*H*), 6.75 (4H, t, *J* = 8 Hz, Ar*H*), 3.04 (6H, q, *J* = 8 Hz, C*H*_2_), 1.12 (9H, t, *J* = 8 Hz, C*H*_3_) ppm; ^13^C NMR (100 MHz, (CD_3_)_2_SO, 298 K): *δ* 163.3 (q), 135.6, 125.3 (q), 121.6, 45.8, 8.7 ppm; MS (ESI^+^): *m*/*z* (%): 102(100); found: M, 102.1278, C_6_H_16_N requires 102.1277.

### 2-Ethylhexylammonium tetraphenylborate (**10**)

To a solution of 2-ethylhexylamine (0.5 mL, 2.9 mmol) in 1 M HCl (25 mL) was added a solution of sodium tetraphenylborate (1.0 g, 2.9 mmol) in H_2_O (25 mL). The reaction mixture was left stirring for 10 min at ambient temperature and the white precipitate that had formed was filtered off, collected, washed with H_2_O (3 × 25 mL) and dried to yield the desired product as a white solid (1.2 g, 92%). ^1^H NMR (400 MHz, (CD_3_)_2_CO, 298 K): *δ* 6.92 (3H, s, br, N*H*_3_^+^), 6.55–6.51 (8H, m, Ar*H*), 6.13 (8H, t, *J* = 8 Hz, Ar*H*), 5.98 (4H, t, *J* = 8 Hz, Ar*H*), 2.18 (2H, s, C*H*_2_), 0.94–0.87 (1H, m, C*H*), 0.65–0.45 (8H, m, C*H*_2_), 0.09–0.05 (6H, m, C*H*_3_) ppm; ^13^C NMR (100 MHz, (THF-d8, 298 K)): *δ* 165.1 (q), 137.0, 126.2 (q), 122.3, 44.4, 35.8, 30.8, 29.3, 23.81, 23.79, 14.4, 10.5 ppm; MS (ESI^+^): *m*/*z* (%): 130(100); found: M, 130.1585, C_8_H_20_N requires 130.1590.

### 1-Methylimidazolium tetraphenylborate (**11**)

To a solution of 1-methylimidazolium chloride (0.035 g, 0.29 mmol) in H_2_O (5 mL) was added a solution of sodium tetraphenylborate (0.10 g, 0.29 mmol) in H_2_O (5 mL). The reaction mixture was left stirring for 10 min at ambient temperature and the white precipitate that had formed was filtered off, collected, washed with H_2_O (3 × 10 mL) and dried to yield the desired product as a white solid (0.11 g, 92%). m.p. 240–242 °C; ^1^H NMR (400 MHz, (CD_3_)_2_SO 298 K): *δ* 8.85 (1H, s, C*H*), 7.55 (2H, d, *J* = 16 Hz, C*H*), 7.30–7.26 (8H, m, Ar*H*), 7.00 (8H, t, *J* = 8 Hz, Ar*H*), 6.85 (4H, t, *J* = 8.0 Hz, Ar*H*), 3.74 (3H, s, C*H*_3_) ppm; ^13^C NMR (100 MHz, (CD_3_)_2_SO 298 K): *δ* 163.4 (q), 135.64, 135.63, 125.4 (q), 123.1, 121.7, 119.8, 35.3 ppm; MS (ESI^+^): *m*/*z* 83(100%); found: M 83.0606, C_4_H_7_N_2_ requires 83.0604.

### UV/Vis absorption titrations

Titrations were carried out on a Cary 3 Bio UV-Vis spectrophotometer, using standard titration protocols.^17^,^19^ A 10 mL sample of the host, Reichardt' dye (**1**) was prepared at a known concentration (typically between 0.15 mM and 0.24 mM in MeCN (**1**), 0.16 mM and 0.20 mM in acetone (**1**), 0.04 mM and 0.12 mM in CHCl_3_ (**1**)). A 2 mL portion of this solution was removed and added to a quartz cuvette, and the UV/Vis spectrum was recorded. The guest (**3–27**) was dissolved in 1–2 mL of the host solution. Aliquots of this solution were successively added to the cuvette, and the UV/Vis absorption spectrum was recorded after each addition. In the presence of large quantities of water (1%) in acetonitrile, the **1·26** H-bonded complex displayed significant quantities of decomposition and thus the data was not used. The UV/Vis absorption spectra were analysed using a Microsoft Excel spreadsheet to fit the changes in the absorption at fixed wavelengths to a 1 : 1 binding isotherm by optimizing the association constant and absorption of the free and bound host using purpose-written VBA macros.

### NMR titrations

Titrations were carried out on a BB 500 MHz spectrometer, using standard titration protocols.^16^ A 5 mL sample of the host, *n*-tributylphosphine oxide (**2**) was prepared at a known concentration (typically between 4 mM and 7 mM in MeCN (**1**), 4 mM and 7 mM in acetone (**1**), 0.1 mM and 5 mM in CHCl_3_ (**1**) and 0.10 mM and 1.5 mM in CCl_4_ (**1**)). A 0.6 mL portion of this solution was removed and added to a NMR tube, and the NMR spectrum was recorded. The guest (**3–27**) was dissolved in 2.5 mL of the host solution to avoid dilution of the host during the titration experiments. Aliquots of this solution were successively added to the NMR tube, and the NMR spectrum was recorded after each addition. The NMR spectra were analysed using a Microsoft Excel spreadsheet to fit the changes in the ^31^P NMR chemical shift as a function of concentration of the guest species to a 1 : 1 binding isotherm by optimizing the association constant and absorption of the free and bound host using purpose-written VBA macros. Deuterated solvents were used for titrations in acetonitrile and chloroform whilst titration experiments conducted in acetone ((CH_3_)_2_CO) and carbon tetrachloride, a capillary containing D_2_O was added to the NMR tube.

## Conflicts of interest

There are no conflicts to declare.

## Abbreviations

Following is a summary of the abbreviations used herein

BAr^F^: Tetrakis(3,5-bis(trifluoromethyl)phenyl)borate
br: Broad
Bu: Butyl
Bz: Benzoyl
d: Doublet
DMF: Dimethylformamide
DMSO: Dimethylsulfoxide
Et: Ethyl
HBA: H-bond acceptor
HBD: H-bond donor
HMPA: Hexamethylphosphoramide
LFER: Linear free energy relationship
m: Multiplet
Me: Methyl
m.p.: Melting point
MOIM: 1-Methyl,-3-octyl,imidazolium
Ph: Phenyl
Pip: Piperidine
q: Quartet
R: 2-Ethylhexyl
s: Singlet
t: Triplet
TBA: Tetrabutylammonium
THF: Tetrahydrofuran

## Supplementary Material

Supplementary information
