# Supplementary material for: H-Bond donor parameters for cations
Source: Chem Sci. 2019 May 16;10(23):5943–51. doi: 10.1039/c9sc00721k (PMC6566073; doi:10.1039/c9sc00721k)
Supplement: Supplementary file 1 [file SC-010-C9SC00721K-s001.pdf]

Electronic Supplementary Material (ESI) for Chemical Science.
This journal is © The Royal Society of Chemistry 2019

Supporting Information

H-Bond Donor Parameters for Cations

Sarah J. Pike,[a] Ennio Lavagnini,[a] Lisa M. Varley,[b] Joanne L. Cook,[c]

Christopher A. Hunter[a*]

[a]Department of Chemistry, University of Cambridge, Cambridge, CB2 1EW, UK.

[b]Department of Chemistry, University of Sheffield, Sheffield, S3 7HF, UK.

[c]Unilever, R&D Port Sunlight, Quarry Road East, Bebington, Wirral, CH63 3JW, UK.

Email: hershelsmith.orgchem@ch.cam.ac.uk

Contents *S2*

General Experimental Details *S4*

Standard Method for UV/vis Absorption Spectroscopy Titrations *S5*

Standard Method for NMR Spectroscopy Titrations *S6*

Synthetic Details *S7*

Titration Curves for Reichardt's dye (1) in Acetonitrile *S11*

Titration Curves for Reichardt's dye (1) in Acetone *S27*

Titration Curves for Reichardt's dye(1) in Acetonitrile with 0.1%H_2O *S44*

Titration Curves for Reichardt's dye(1) I n MeCN with 0.25% H_2O *S47*

Titration Curves for Reichardt's dye(1) in MeCN with 0.5% H_2O *S50*

Titration Curves for Reichardt's dye(1) in MeCN with 1% H_2O *S53*

Titration Curves for Reichardt's dye(1) in Acetone with 0.1% H_2O *S55*

Titration Curves for Reichardt's dye(1) in acetone with 0.25% H_2O *S58*

Titration Curves for Reichardt's dye(1) in acetone with 0.5% H_2O *S61*

Titration Curves for Reichardt's dye(1) in acetone with 1% H_2O *S64*

Titration Curves for Reichardt's dye (1) in Chloroform - UV/vis *S67*

Titration Curves for Phosphine oxide (2) in Acetonitrile - UV/vis *S70*

Titration Curves for Phosphine Oxide (2) in Chloroform – UV/vis *S73*

Titration Curves for Phosphine oxide (2) in Acetone – UV/vis *S77*

Titration Curves for Phosphine oxide (2) in Carbon Tetrachloride- UV/vis *S78*

Titration Curves for Phosphine oxide (2) in Acetonitrile - NMR *S81*

Titration Curves for Phosphine oxide (2) in Acetone - NMR *S97*

Titration Curves for Phosphine oxide (2) in Chloroform – NMR *S116*

Titration Curves for Phosphine oxide (2) in Carbon Tetrachloride- NMR *S120*

Summary of Association Constants for H-bonded complexes *S125*

Counterion Influence in Acetonitrile on *K* values of H-bonded complexes *S128*

Influence of the H_2O on *K* values of H-bonded complexes in Acetonitrile *S130*

Influence of the H_2O on *K* values of H-bonded complexes in Acetone *S135*

Appendix: ^{1}H and ^{13}C NMR spectra for new compounds *S140*

References *S152*

General Experimental Details

All compounds were purchased from Sigma-Aldrich unless otherwise stated. 4-Nitrophenol, 4-nitro,3-trifluoromethylphenol, tributylphosphine oxide, sodium trifluoromethanesulfonimide and bis(4-tert-butylphenyl)iodonium hexafluorophosphate were purchased from Aldrich. Sodium hexafluorophosphate, 5-nitroindole, potassium tetraphenylborate, 1-butyl-3-methylimidazolium hexafluorophosphate and 1-butyl-4-methylpyridinium hexafluorophosphate were purchased from Alfa Aesar. Sodium tetrafluoroborate was purchased from BDH Chemicals Ltd. Cesium tetraphenylborate, rubidium tetraphenylborate and lithium tetraphenylborate tris(1,2-dimethoxyethane) were purchased from Chem Cruz. Silver tetrafluoroborate was purchased from Acros Organic. Acetonitrile, acetone and chloroform were purchased from Acros as 99+% for spectroscopic grade. Carbon tetrachloride was purchased from Fluka Chemicals. All chemicals were used as received. All NMR spectroscopy was carried out on a Bruker AVI400 spectrometer using the residual solvent as the internal standard. All the chemical shifts (δ) are quoted in ppm and coupling constants are given in Hz. Splitting patterns are given as follows: s (singlet), br (broad), d (doublet), t (triplet), m (multiplet). FT-IR spectra were measured on a PerkinElmer Spectrum 100. Melting points were measured in a Mettler Toledo MP50 Melting Point System. ES+ was carried out on a Waters LCT-TOF spectrometer or a Waters Xevo G2-S bench top QTOF machine. All compounds were used as received. The measurements of solids were carried out on a Precisa 125A balance. All NMR experiments were carried out on a 400 MHz Bruker AMX400 spectrometer.

The following abbreviations are employed: Ar = aromatic, BA^rF = tetrakis(3,5-bis(trifluoromethyl)phenyl)borate, br = broad, Bu = butyl, Bz = benzyl, d = doublet, DMA = N,N-dimethylacetamide, DMF = dimethylformamide, DMSO = dimethylsulfoxide, Et = ethyl,

$EtPipH^+$ = 1-ethylpiperidinium, HBA = H-bond acceptor, HBD = H-bond donor, HMPA = hexamethylphosphoramide, m = multiplet, Me = methyl, m.p. = melting point, $MePipH^+$ = 1-methylpiperidinium, NMA = N,methylacetamide, Oct = octyl, Ph = phenyl, $PipH^+$ = piperidinium, R = 2-ethylhexyl, s = singlet, t = triplet, TBA = tetrabutylammonium, TFA = trifluoroacetic acid, THF = tetrahydrofuran.

Standard Method for UV/vis Absorption Titrations[1-4]

Titrations were carried out on a Cary 3 Bio UV-Vis spectrophotometer, using standard titration protocols. A 10 mL sample of the host, Reichardt' dye (**1**) was prepared at a known concentration (typically between 0.15 mM and 0.24 mM in MeCN (**1**), 0.16 mM and 0.20 mM in acetone (**1**), 0.04 mM and 0.12 mM in $CHCl_3$ (**1**). A 2 mL portion of this solution was removed and added to a quartz cuvette, and the UV/vis spectrum was recorded. The guest (**3**-**27**) was dissolved in 1-2 mL of the host solution. Aliquots of this solution were successively added to the cuvette, and the UV/vis absorption spectrum was recorded after each addition. The UV/vis absorption spectra were analysed using a Microsoft Excel spreadsheet to fit the changes in the absorption at fixed wavelengths to a 1:1 binding isotherm by optimizing the association constant and absorption of the free and bound host using purpose-written VBA macros.

For a given estimate of the association constant (*K*), an iterative VBA macro was used to solve Equations S1-S3 for the concentrations of all species present in the cuvette at every point in the titration.

$[H{\bullet}G] = K\,[H]\,[G]$ (Equ. S1)

where [H] is the concentration of free host, [G] is the concentration of free guest and [H•G] is the concentration of complex.

$[G]_0 = [G] + [H{\bullet}G]$ (Equ. S2)

where $[G]_0$ is the total concentration of guest in the cuvette.

$[H]_0 = [H] + [H•G]$ (Equ. S3)
where $[H]_0$ is the total concentration of guest in the cuvette.

For a given estimate of the extinction coefficients of the free and bound host (ε_H and $\varepsilon_{H•G}$), the absorbance (A) at every point in the titration was calculated using Equation S4.

$A = \varepsilon_H [H] + \varepsilon_{H•G} [H•G]$ (Equ. S4)
The values of K, ε_H and $\varepsilon_{H•G}$ were then optimised to minimise the difference between the experimental and calculated values of A using an iterative VBA macro. In a small number of cases, only 50% saturation of the isotherm was reached, but the consistency in the fitted values of the bound extinction coefficients with experiments that did reach saturation provides confidence in the accuracy of the association constant (Table S1).

Standard Method for NMR Spectroscopy Titrations[5]

Titrations were carried out on a BB 500 MHz spectrometerr, using standard titration protocols.[1] A 5 mL sample of the host was prepared at a known concentration (typically between 4 mM and 7 mM in MeCN (**2**), 4 mM and 7 mM in acetone (**2**), 0.1 mM and 5 mM in $CHCl_3$ (**2**) and 0.10 mM and 1.5 mM in CCl_4 (HBD). A 0.6 mL portion of this solution was removed and added to a NMR tube, and the NMR spectrum was recorded. The guest (**3**-**27** in MeCN or acetone, and **2** in CCl_4) was dissolved in 2.5 mL of the host solution to avoid dilution of the host during the titration experiments. Aliquots of this solution were successively added to the NMR tube, and the NMR spectrum was recorded after each addition. The NMR spectra were analysed using a Microsoft Excel spreadsheet to fit the changes in the ^{31}P or ^{1}H NMR chemical shift as a function of concentration of the guest species to a 1:1 binding isotherm by optimizing the association constant and absorption of the free and bound host using purpose-written VBA macros. Deuterated solvents were used for titrations in acetonitrile and chloroform whilst titration experiments conducted in acetone ($(CH_3)_2CO$) and carbon tetrachloride, a capillary containing D_2O was added to the NMR tube.

For a given estimate of the association constant (K), an iterative VBA macro was used to solve Equations S1-S3 for the concentrations of all species present in the NMR tube at every point in the titration. For a given estimate of the chemical shifts of the free and bound host (δ_H and $\delta_{H\bullet G}$), the chemical shift (δ) at every point in the titration was calculated using Equation S5.

$$\delta = \delta_H [H]/[H]_0 + \delta_{H\bullet G} [H\bullet G]/[H]_0 \qquad \text{(Equ. S5)}$$

The values of K, δ_H and $\delta_{H\bullet G}$ were then optimised to minimise the difference between the experimental and calculated values of δ using an iterative VBA macro. In all cases, greater than 60% saturation of the binding isotherm was reached.

Synthetic Details

Guanidinium tetraphenylborate

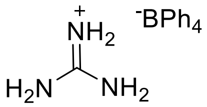

To a solution of guanidinium hydrochloride (0.028 g, 0.29 mmol) in H_2O (5 mL) was added a solution of sodium tetraphenylborate (100 mg, 0.29 mmol) in H_2O (5 mL) and the resultant solution was left stirring at room temperature for 10 mins. The white precipitate that had formed was filtered off, washed with H_2O (3 x 10 mL) and dried to give the desired compound as a white solid. (105 mg, 95%) m.p. 220-223 °C; ^1H NMR (400 MHz, CD_3CN, 298 K): δ 7.30 – 7.25 (8H, m, Ar*H*), 7.00 (8H, t, 4J = 8.0 Hz, Ar*H*), 6.87 – 6.82 (4H, m, Ar*H*), 5.92 (6H, s, br, 2 × NH_2 and $^+NH_2$); ^{13}C NMR (100 MHz, CD_3CN, 298 K): δ 164.8 (q), 158.8, 136.7 (q), 126.6 (q), 122.8; MS(ESI^+): m/z (%): 60(100); Found: M, 60.0559, $C_1H_6N_3$ requires 60.0556).

Triethylammonium tetraphenylborate

$Et_3NH^+ \ ^-BPh_4$

To a solution of triethylamine hydrochloride (0.040 g, 0.29 mmol) in H_2O (5 mL) was added a solution of sodium tetraphenylborate (0.10 g, 0.29 mmol) in H_2O (5 mL). The reaction mixture was left stirring for 10 mins at ambient temperature and the white precipitate that had formed was filtered off, collected, washed with H_2O (3 x 10 mL) and dried to yield the desired product as a white solid (0.12 g, 93%). m.p. 182-184 °C; ^{1}H NMR (400 MHz, $(CD_3)_2SO$, 298 K): δ 8.79 (1H, s, br, NH^+), 7.16-7.11 (8H, m, Ar*H*), 6.88 (8H, t, J = 8 Hz, Ar*H*), 6.75 (4H, t, J = 8 Hz, Ar*H*), 3.04 (6H, q, J = 8 Hz, CH_2), 1.12 (9H, t, J = 8 Hz, CH_3) ppm; ^{13}C NMR (100 MHz, $(CD_3)_2SO$, 298 K): δ 163.3 (q), 135.6, 125.3 (q), 121.6, 45.8, 8.7 ppm; MS(ESI$^+$): m/z (%): 102(100); Found: M, 102.1278, $C_6H_{16}N$ requires 102.1277).

2-Ethylhexylammonium tetraphenylborate

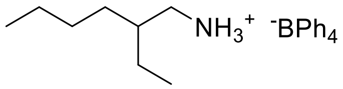

To a solution of 2-ethylhexylamine (0.5 mL, 2.9 mmol) in 1 M HCl (25 mL) was added a solution of sodium tetraphenylborate (1.0 g, 2.9 mmol) in H_2O (25 mL). The reaction mixture was left stirring for 10 mins at ambient temperature and the white precipitate that had formed was filtered off, collected, washed with H_2O (3 x 25 mL) and dried to yield the desired product as a white solid (1.2 g, 92%). ^{1}H NMR (400 MHz, $(CD_3)_2CO$, 298 K): δ 6.92 (3H, s, br, NH_3^+), 6.55-6.51 (8H, m, Ar*H*), 6.13 (8H, t, J = 8 Hz, Ar*H*), 5.98 (4H, t, J = 8 Hz, Ar*H*), 2.18 (2H, s, CH_2), 0.94-0.87 (1H, m, C*H*), 0.65-0.45 (8H, m, CH_2), 0.09-0.05 (6H, m, CH_3) ppm; 13C NMR (100 MHz, (THF-d_8, 298 K): δ 165.1 (q), 137.0, 126.2 (q), 122.3, 44.4, 35.8, 30.8, 29.3, 23.81, 23.79, 14.4, 10.5 ppm; MS(ESI$^+$): m/z (%): 130(100); Found: M, 130.1585, $C_8H_{20}N$ requires 130.1590).

1-methylimidazolium tetraphenylborate

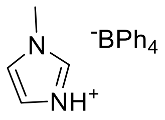

To a solution of 1-methylimidazolium chloride (0.035 g, 0.29 mmol) in H_2O (5 mL) was added a solution of sodium tetraphenylborate (0.10 g, 0.29 mmol) in H_2O (5 mL). The reaction mixture was left stirring for 10 mins at ambient temperature and the white precipitate that had formed was filtered off, collected, washed with H_2O (3 x 10 mL) and dried to yield the desired product as a white solid (0.11 g, 92%). m.p. 240-242 °C; ^{1}H NMR (400 MHz, $(CD_3)_2SO$ 298 K): δ 8.85 (1H, s, C*H*), 7.55 (2H, d, J = 16 Hz, C*H*), 7.30-7.26 (8H, m, Ar*H*), 7.00 (8H, t, J = 8 Hz, Ar*H*), 6.85 (4H , t, J = 8.0 Hz, Ar*H*), 3.74 (3H, s, C*H*$_3$) ppm; ^{13}C NMR (100 MHz, $(CD_3)_2SO$ 298 K): δ 163.4 (q), 135.64, 135.63, 125.4 (q), 123.1, 121.7, 119.8, 35.3 ppm; MS (ESI$^+$): m/z 83(100%); Found: M 83.0606, $C_4H_7N_2$ requires 83.0604).

2. Titration Curves for Reichardt's dye (1) in Acetonitrile

a) <u>Titration of Reichardt's dye with 4-azophenol in acetonitrile</u>

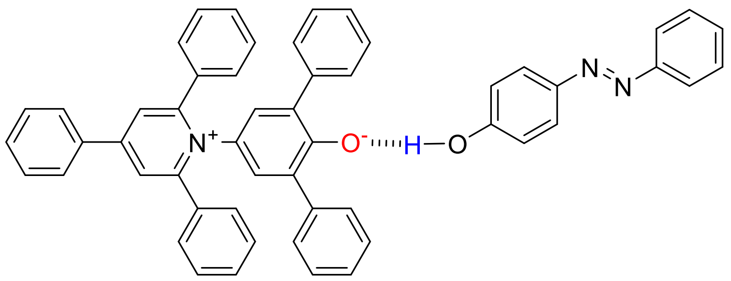

Host: Reichardt's dye = 0.16 mM
Guest: 4-phenylazophenol = 42 mM

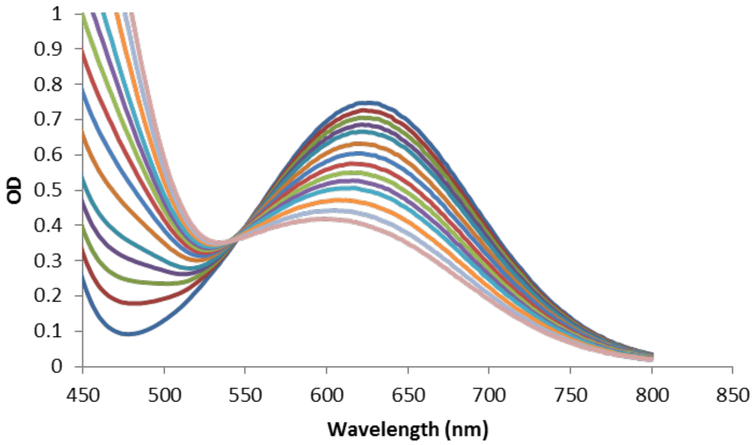

Graph S1. UV/vis spectra of titration of Reichardt's dye with 4-phenylazophenol in acetonitrile.

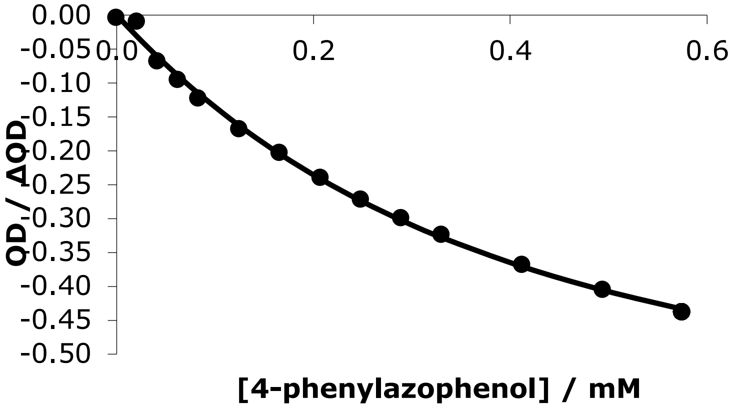

Graph S2. Binding isotherms for titration using 1:1 fitting program for titration of 4-phenylazophenol against Reichardt's dye in acetonitrile. [Reichardt's dye] = 0.16 mM and [4-phenylazophenol] = 42 mM, ΔOD = -0.694.

K_a = 3100 ± 650 M^{-1} 63% bound

b) Titration of Reichardt's dye with phenol in acetonitrile

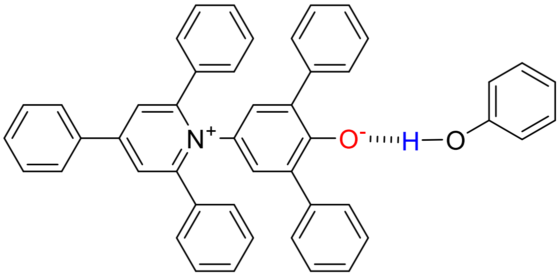

Host: Reichardt's dye = 0.16 mM
Guest: phenol = 95 mM

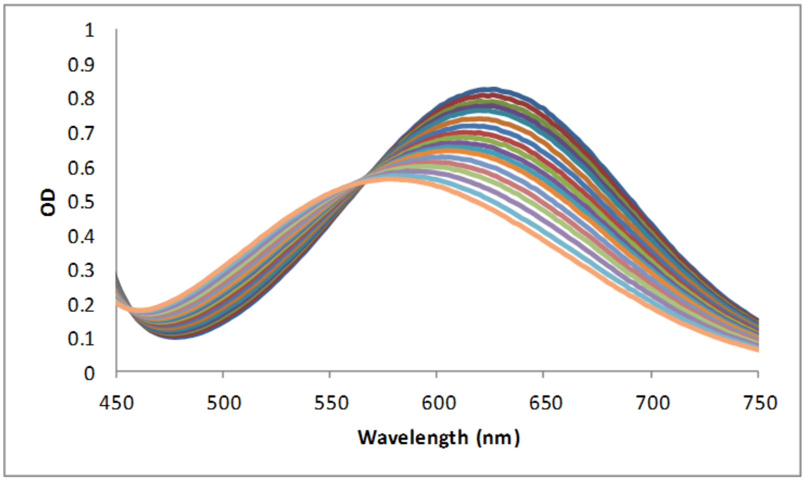

Graph S3. UV/vis spectra of titration of phenol with Reichardt's dye in acetonitrile.

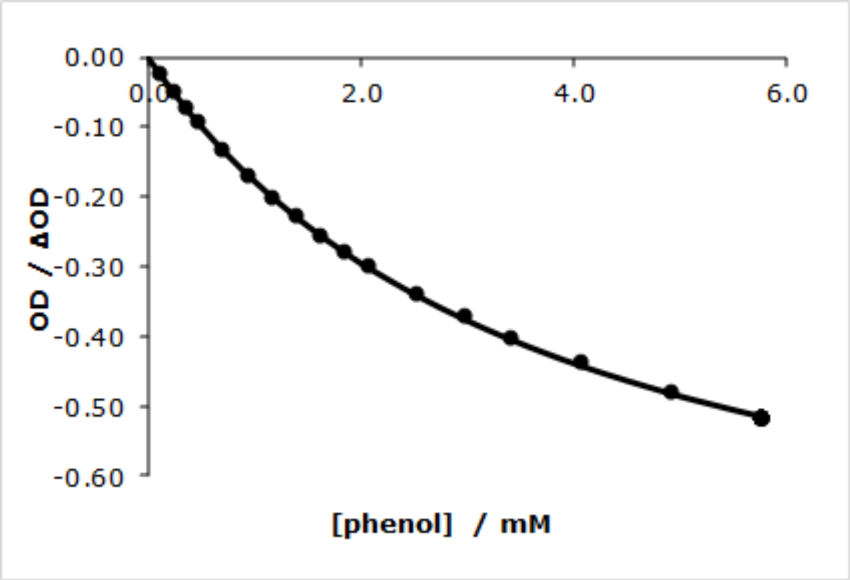

Graph S4. Binding isotherms for titration using 1:1 fitting program for titration of phenol against Reichardt's dye in acetonitrile.[Reichardt's dye] = 0.16 mM and [phenol] =95mM,ΔOD = -0.844

K_a = 260 ± 63 M^{-1} 61% bound

c) <u>Titration of Reichardt's dye with 5-nitroindole in acetonitrile</u>

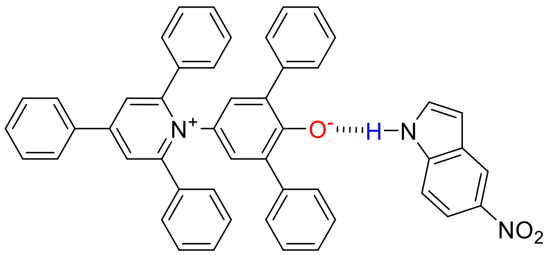

Host: Reichardt's dye = 0.17 mM
Guest: 5-nitroindole = 23 mM

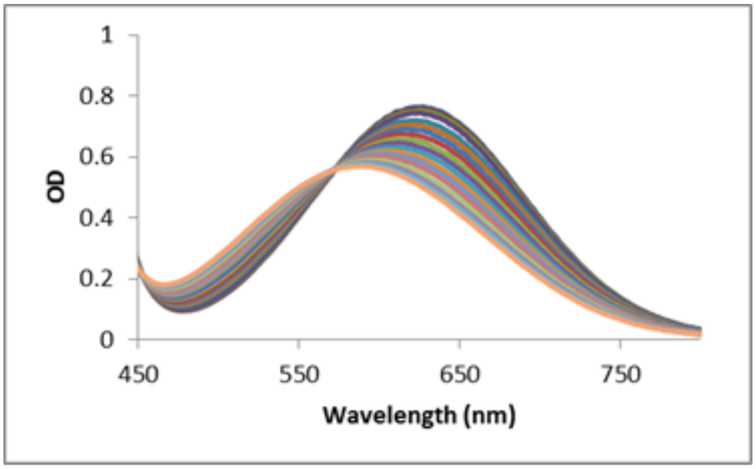

Graph S5. UV/vis spectra of titration of Reichardt's dye with 5-nitroindole in acetonitrile.

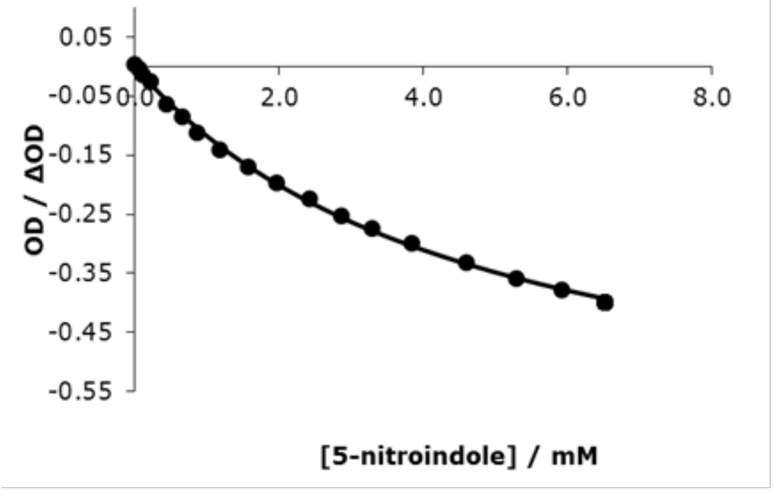

Graph S6. Binding isotherms for titration using 1:1 fitting program for titration of 5-nitroindole against Reichardt's dye in acetonitrile. [Reichardt's dye] = 0.17 mM and [5-nitroindole] = 23 mM, ΔOD = -0.671.

K_a = 200 ± 50 M^{-1} 58% bound

d) Titration of Reichardt's dye with TFA in acetonitrile

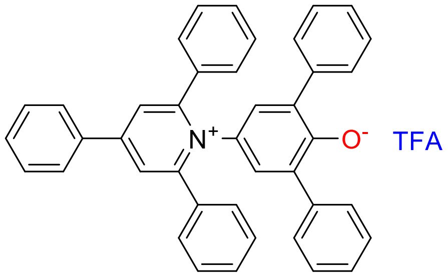

Host: Reichardt's dye = 0.16 mM

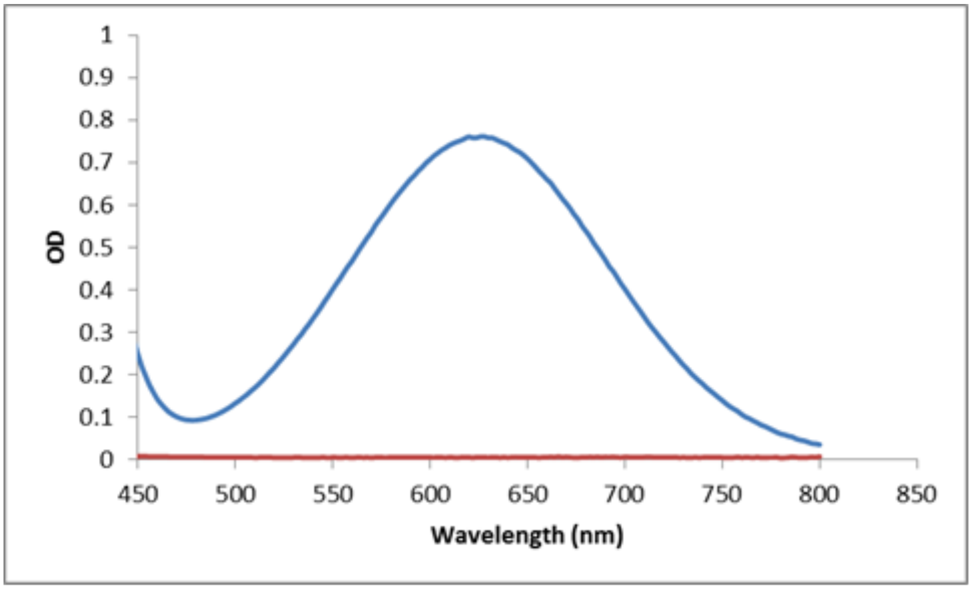

Graph S7. UV/vis spectra of Reichardt's dye when treated with 10 μL of TFA in acetonitrile.

e) <u>Titration of Reichardt's dye with 4-nitro,3-trifluoromethylphenol in acetonitrile</u>

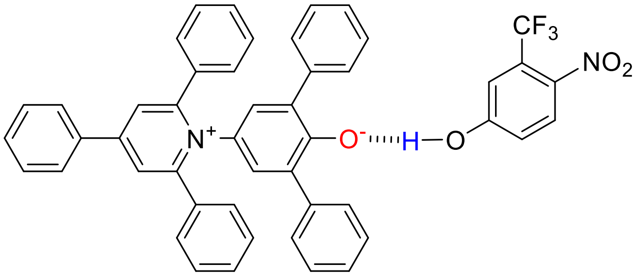

Host: Reichardt's dye = 0.15 mM
Guest: 4-nitro,3-trifluoromethylphenol = 11 mM

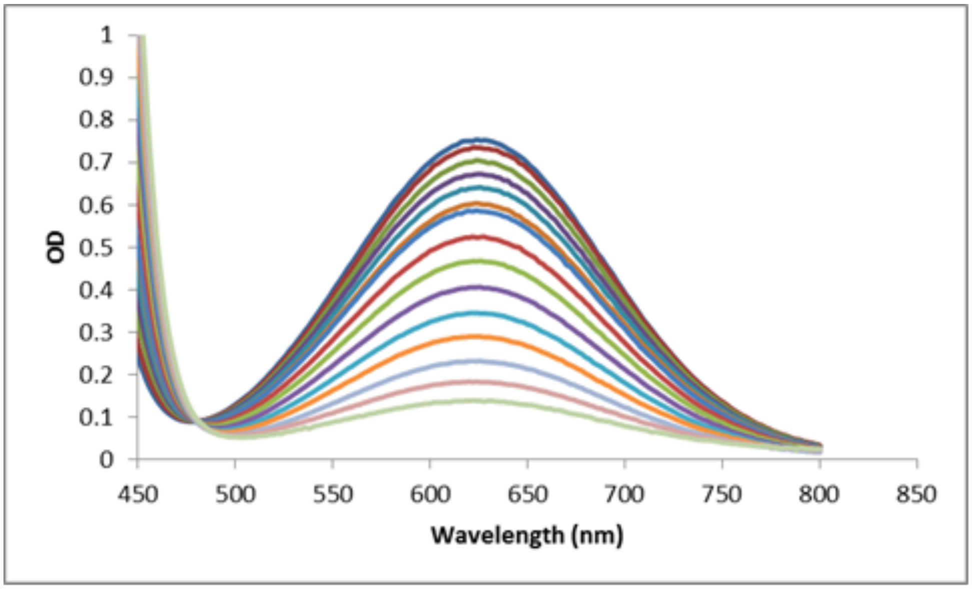

Graph S8. UV/vis spectra of titration of 4-nitro,3-trifluoromethylphenol with Reichardt's dye in acetonitrile.

f) Titration of Reichardt's dye with $Ph_3C^+.BF_4$ in acetonitrile

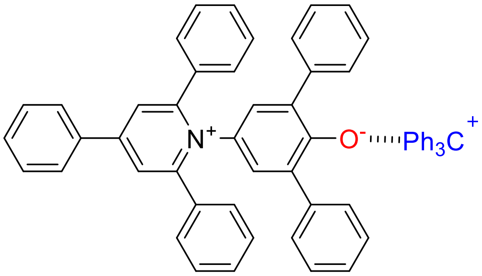

Host: Reichardt's dye = 0.16 mM
Guest: $PhC_3.BF_4$ = 15 mM

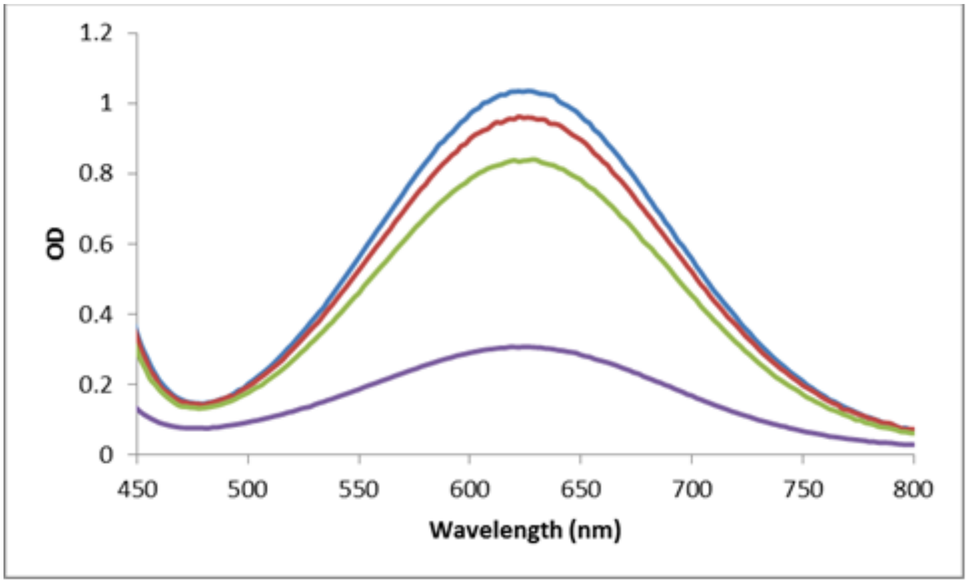

Graph S9. UV/vis spectra of titration of Reichardt's dye with $Ph_3C^+.BF_4$ in acetonitrile.

g) <u>Titration of Reichardt's dye with guanidinium.BPh_4 in acetonitrile</u>

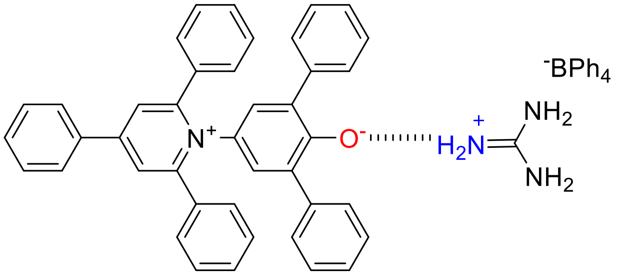

Host: Reichardt's dye = 0.20 mM
Guest: guanidinium.BPh_4 = 15 mM

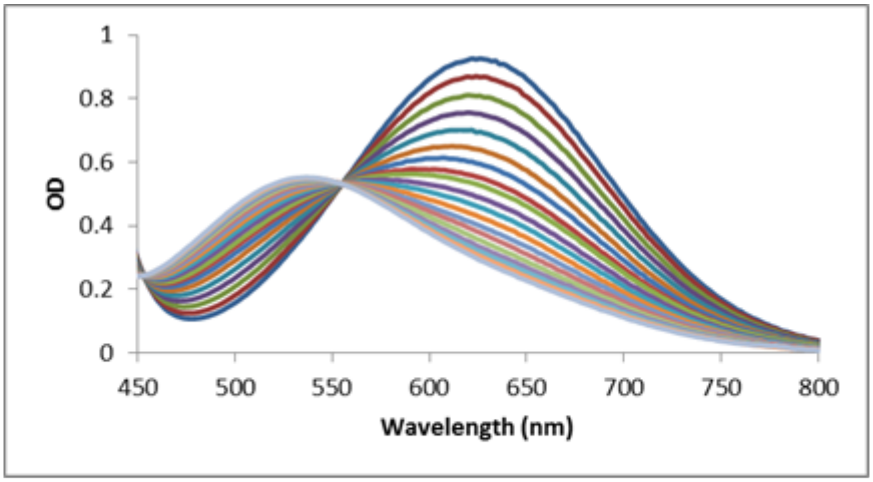

Graph S10. UV/vis spectra of titration of guanidinium.BPh_4 with Reichardt's dye in acetonitrile.

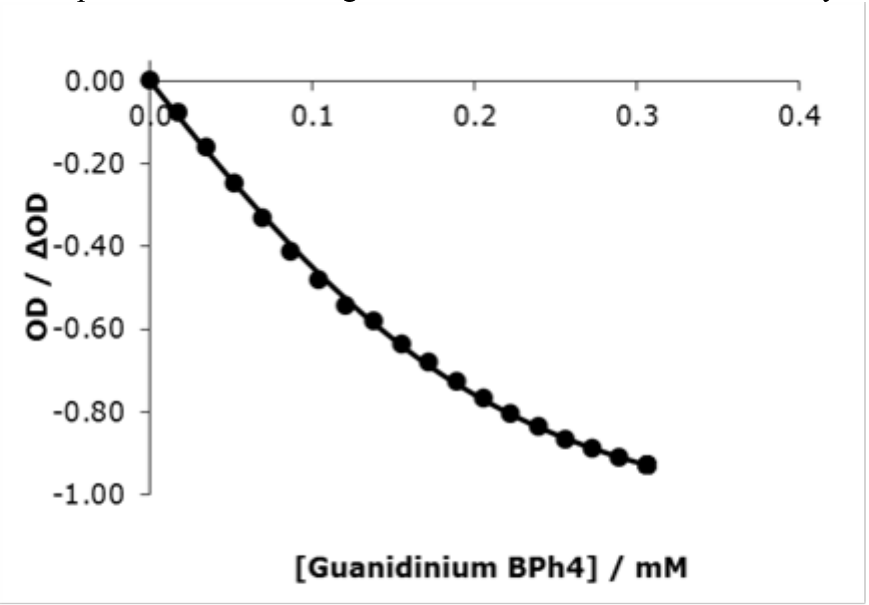

Graph S11. Binding isotherms for titration using 1:1 fitting program for titration of phenol against Reichardt's dye in acetonitrile. [Reichardt's dye] = 0.20 mM and [guanidinium.BPh_4] = 14 mM, ΔOD = -1.178.

K_a = 33800 ± 2000 M^{-1} 79% bound

h) Titration of Reichardt's dye with $LiBPh_4$ tris(1,2-dimethoxyethane) in acetonitrile

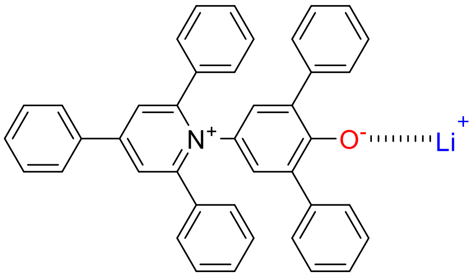

Host: Reichardt's dye = 0.2 mM
Guest: $LiBPh_4$ tris(1,2-dimethoxyethane) = 3 mM

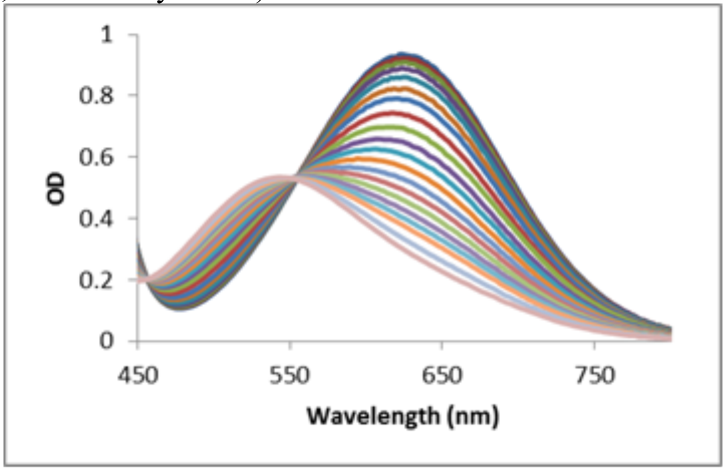

Graph S12. UV/vis spectra of titration of $LiBPh_4$ tris(1,2-dimethoxyethane with Reichardt's dye in acetonitrile.

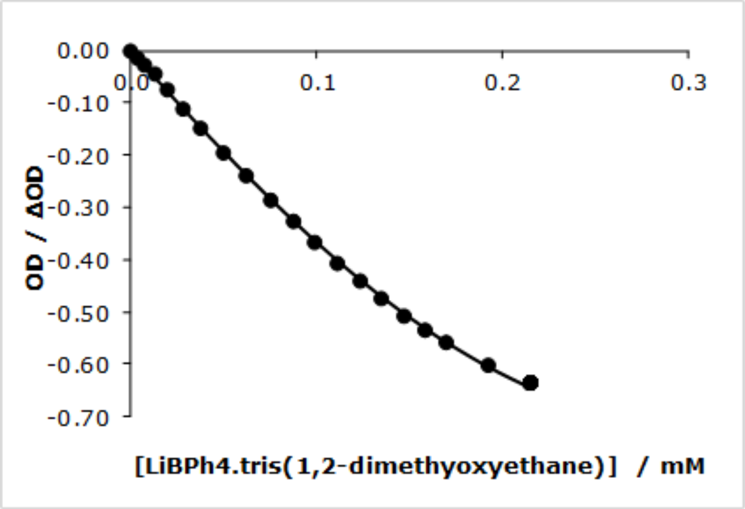

Graph S13. Binding isotherms for titration using 1:1 fitting program for titration of $LiBPh_4$ tris(1,2-dimethoxyethane) against Reichardt's dye in acetonitrile. [Reichardt's dye] = 0.2 mM and [$LiBPh_4$ tris(1,2-dimethoxyethane)] = 3 mM, $\Delta OD = -0.924$.

.

$$K_a = \textbf{27400} \pm \textbf{5900 M}^{-1}$$ **70% bound**

i) Titration of Reichardt's dye with $NaBPh_4$ in acetonitrile

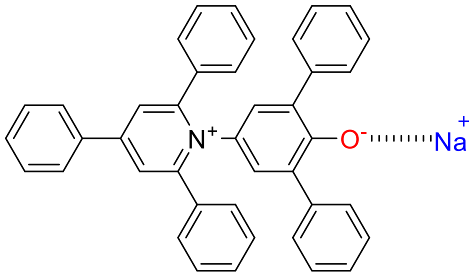

Host: Reichardt's dye = 0.2 mM
Guest: $NaBPh_4$ = 104 mM

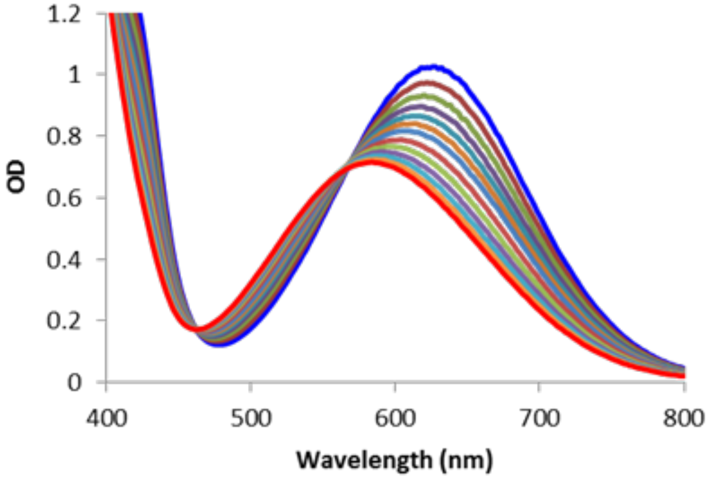

Graph S14. UV/vis spectra of titration of $NaBPh_4$ with Reichardt's dye in acetonitrile.

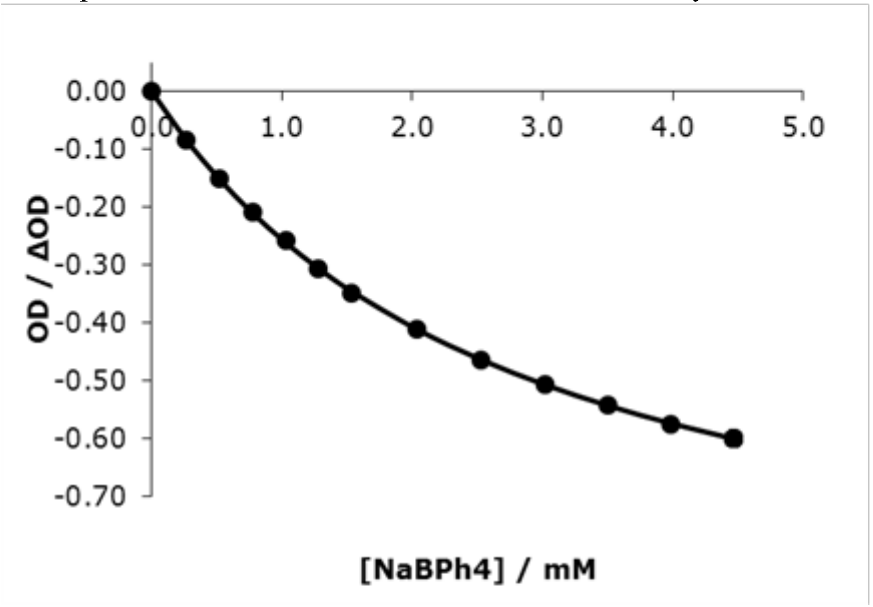

Graph S15. Binding isotherms for titration using 1:1 fitting program for titration of phenol against Reichardt's dye in acetonitrile. [Reichardt's dye] = 0.2 mM and [$NaBPh_4$] = 104 mM, ΔOD = -0.959.

K_a = 390 ± 51 M^{-1} 63% bound

j) Titration of Reichardt's dye with $NaBF_4$ in acetonitrile

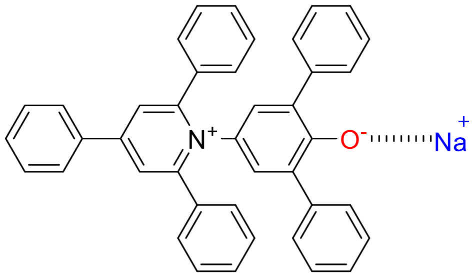

Host: Reichardt's dye = 0.20 mM
Guest: $NaBF_4$ = 76 mM

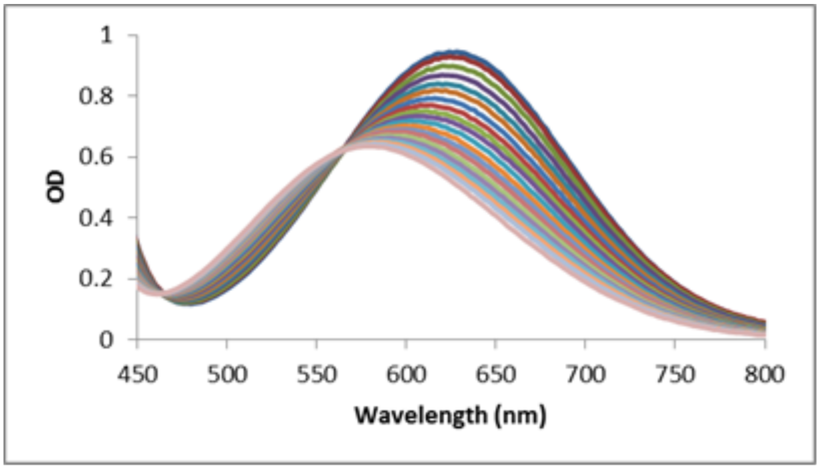

Graph S16. UV/vis spectra of titration of $NaBF_4$ with Reichardt's dye in acetonitrile.

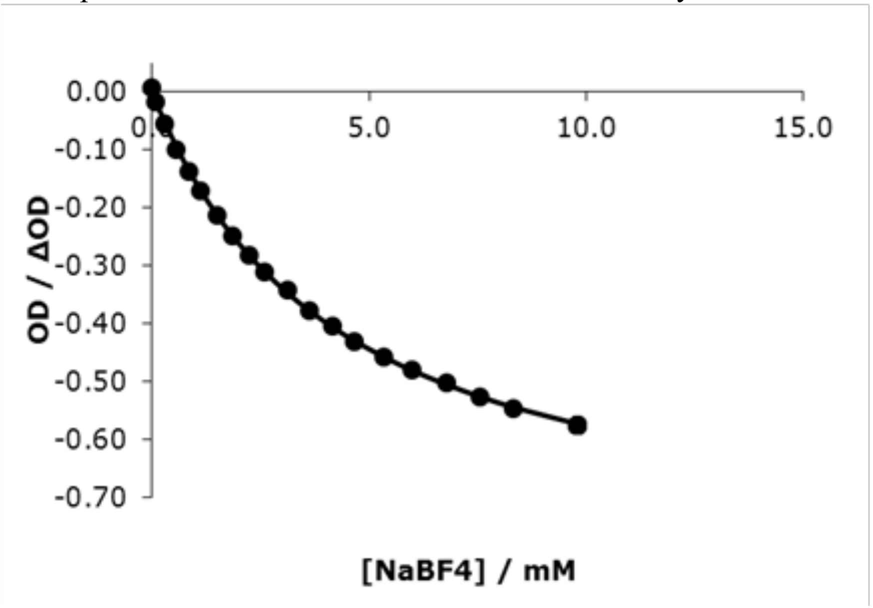

Graph S17. Binding isotherms for titration using 1:1 fitting program for titration of phenol against Reichardt's dye in acetonitrile. [Reichardt's dye] = 0.20 mM and [$NaBF_4$] = 76 mM, ΔOD = -0.817.

K_a = 300 ± 90 M^{-1} 70% bound

k) Titration of Reichardt's dye with 1-butyl-4-methylpyridiniumPF_6 in acetonitrile

Host: Reichardt's dye = 0.16 mM
Guest: 1-butyl-4-methylpyridiniumPF_6 = 569 mM

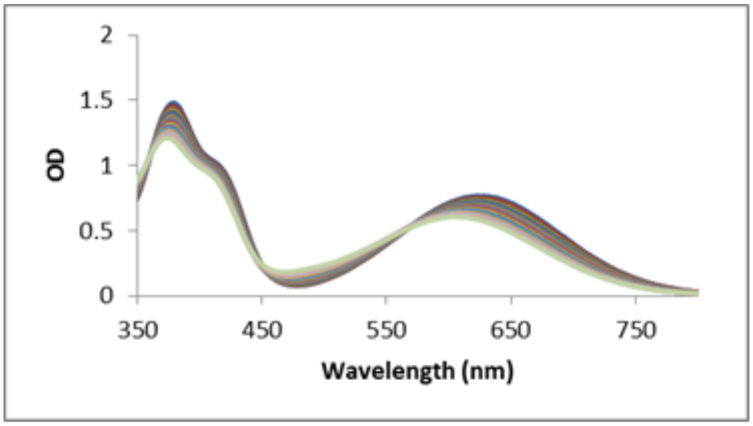

Graph S18. UV/vis spectra of titration of1-butyl-4-methylpyridiniumPF_6 with Reichardt's dye in acetonitrile.

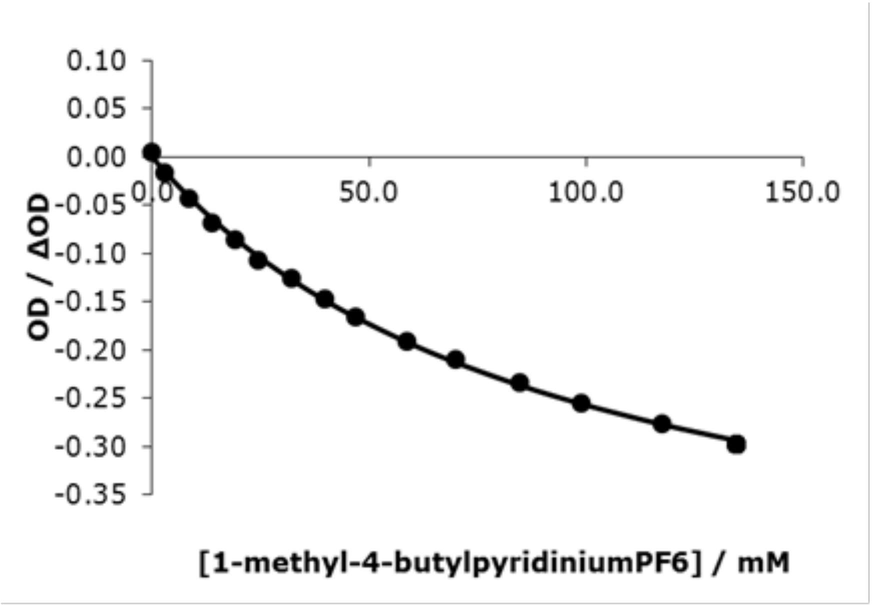

Graph S19. Binding isotherms for titration using 1:1 fitting program for titration of 1-butyl-4-methylpyridiniumPF_6 against Reichardt's dye in acetonitrile. [Reichardt's dye] = 0.16 mM and [1-butyl-4-methylpyridiniumPF_6] = 569 mM, ΔOD = -0.498.

K_a = 12 ± 2 M^{-1} 59% bound

l) <u>Titration of Reichardt's dye with 1-butyl-4-methylpyridiniumPF_6 in acetonitrile</u>

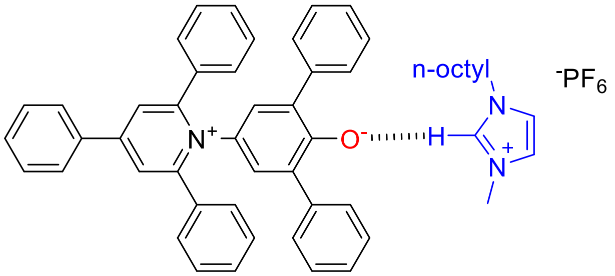

Host: Reichardt's dye = 0.16 mM
Guest: imidiazoliumPF_6 = 374 mM

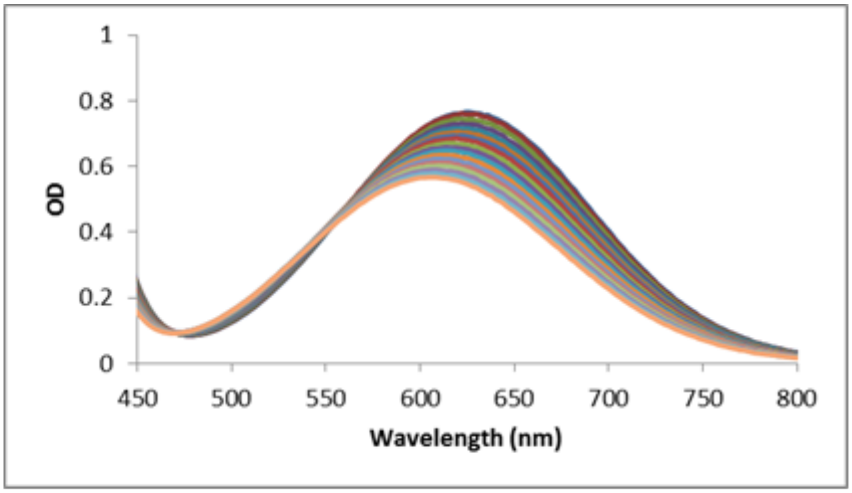

Graph S20. UV/vis spectra of titration of imidiazoliumPF_6 with Reichardt's dye in acetonitrile.

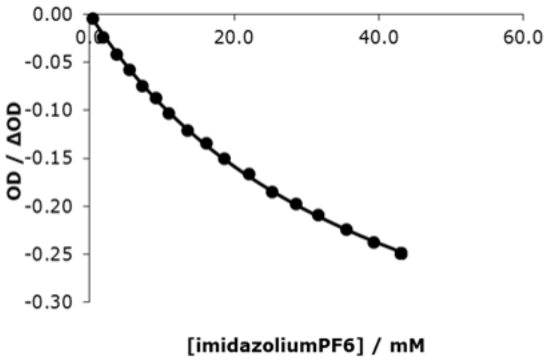

Graph S21. Binding isotherms for titration using 1:1 fitting program for titration of imidazoliumPF_6 against Reichardt's dye in acetonitrile. [Reichardt's dye] = 0.16 mM and [imidiazoliumPF_6] = 374 mM, ΔOD = -0.481.

K_a = 23 ± 9 M^{-1} 51% bound

m) <u>Titration of Reichardt's dye with IPh_2BF_4 in acetonitrile</u>

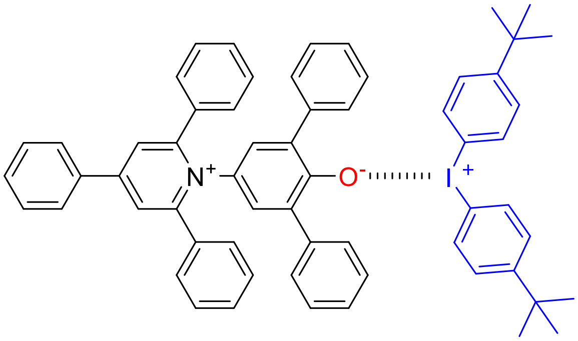

Host: Reichardt's dye = 0.24 mM
Guest: IPh_2BF_4 = 44 mM

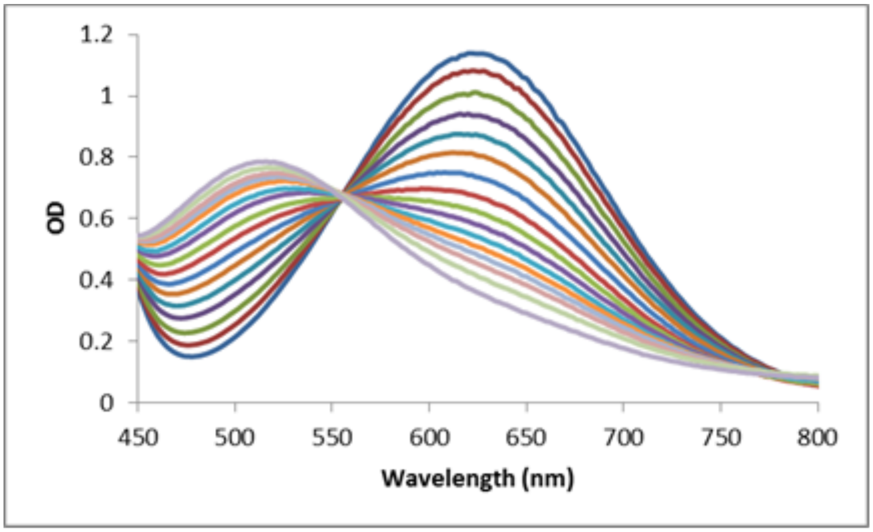

Graph S22. UV/vis spectra of titration of $IPh_2BF_4$with Reichardt's dye in acetonitrile.

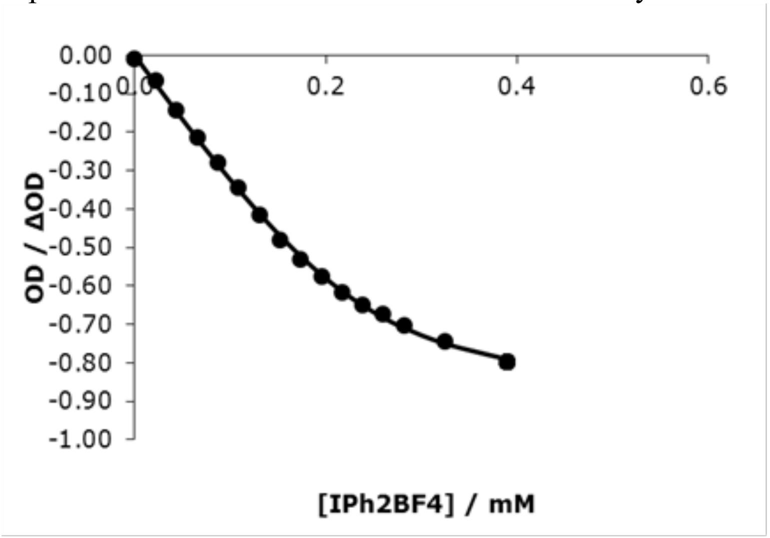

Graph S23. Binding isotherms for titration using 1:1 fitting program for titration of phenol against Reichardt's dye in acetonitrile. [Reichardt's dye] = 0.24 mM and [IPh_2BF_4] = 44 mM, ΔOD = -0.904.

K_a = 38300 ± 1100 M^{-1} 87% bound

n) Titration of Reichardt's dye with $NaBAr^F$ in acetonitrile

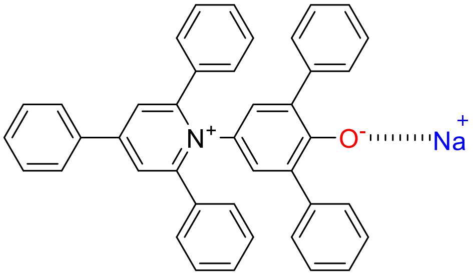

Host: Reichardt's dye = 0.16 mM
Guest: $NaBAr^F$ = 123 mM

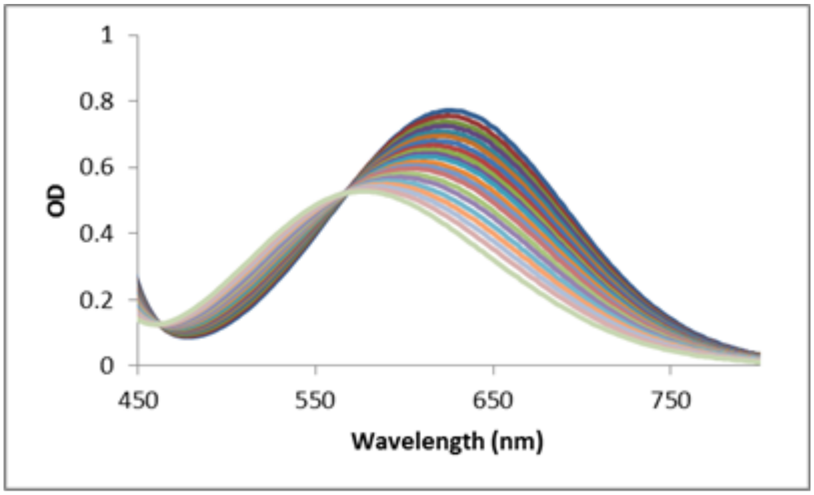

Graph S24. UV/vis spectra of titration of $NaBAr^F$ with Reichardt's dye in acetonitrile.

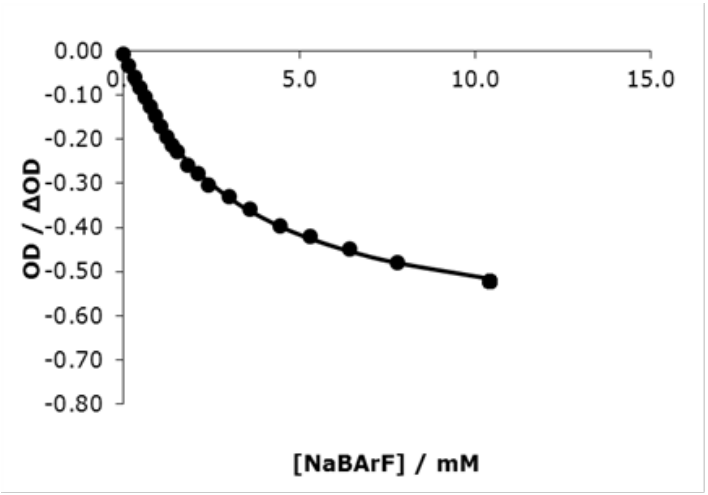

Graph S25. Binding isotherms for titration using 1:1 fitting program for titration of phenol against Reichardt's dye in acetontrile. [Reichardt's dye] = 0.16 mM and [$NaBAr^F$] = 123 mM, ΔOD = -0.660.

$\boldsymbol{K_a}$ **= 400 ± 160 M^{-1}** **78% bound**

o) <u>Titration of Reichardt's dye with $NaN(SO_2CF_3)_2$ in acetonitrile</u>

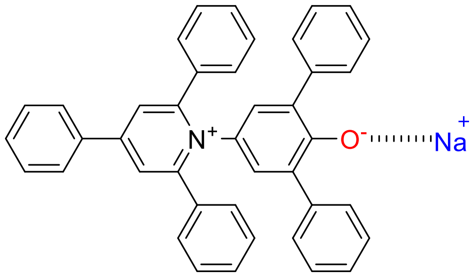

Host: Reichardt's dye = 0.17 mM
Guest: $NaN(SO_2CF_3)_2$ = 76 mM

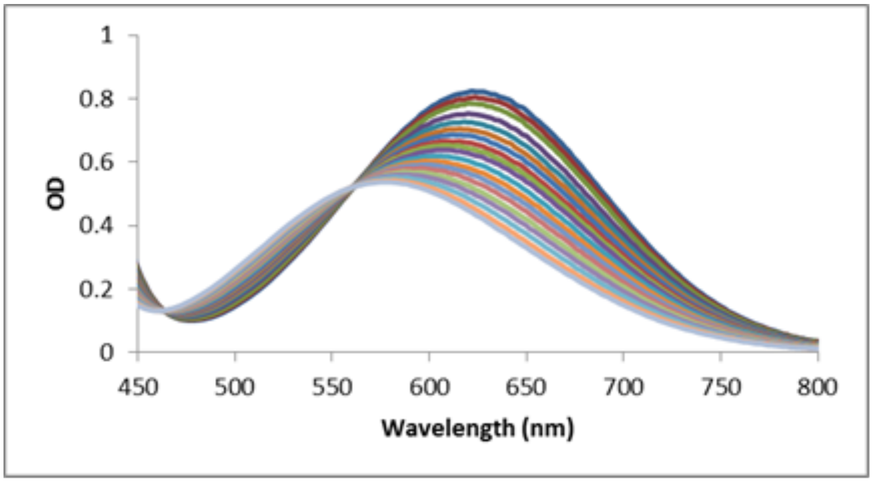

Graph S26. UV/vis spectra of titration of $NaN(SO_2CF_3)_2$ with Reichardt's dye in acetonitrile.

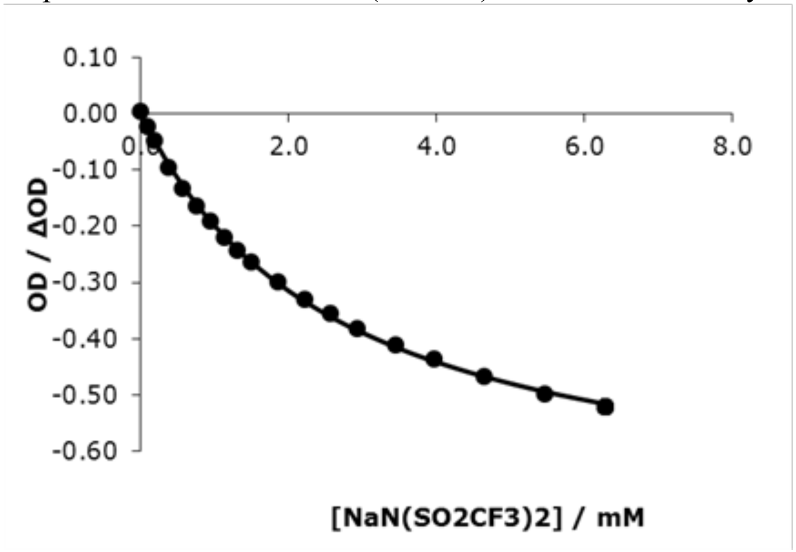

Graph S27. Binding isotherms for titration using 1:1 fitting program for titration of phenol against Reichardt's dye in acetonitrile. [Reichardt's dye] = 0.17 mM and [$NaN(SO_2CF_3)_2$] = 76 mM, ΔOD = -0.726.

K_a = 410 ± 30 M^{-1} 71% bound

p) <u>Titration of Reichardt's dye with NaI in acetonitrile</u>

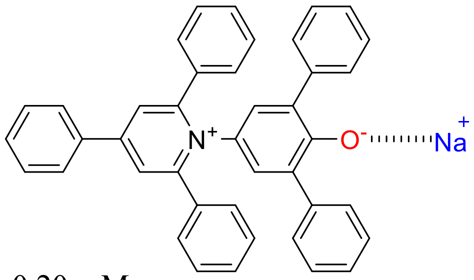

Host: Reichardt's dye = 0.20 mM
Guest: NaI = 142 mM

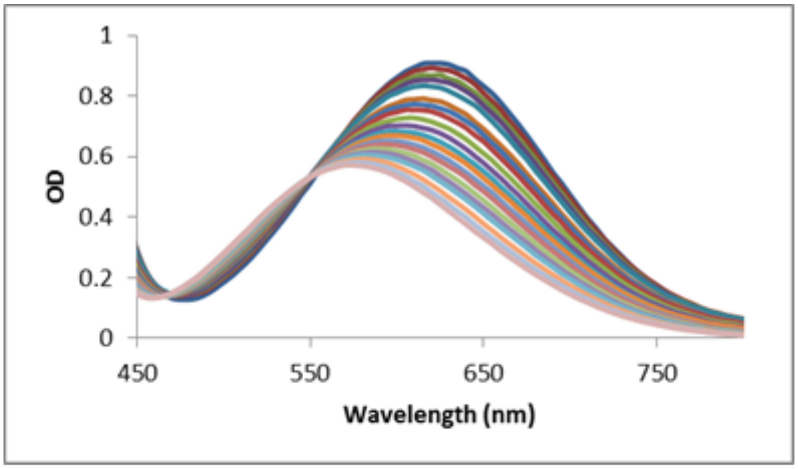

Graph S28. UV/vis spectra of titration of NaI with Reichardt's dye in acetonitrile.

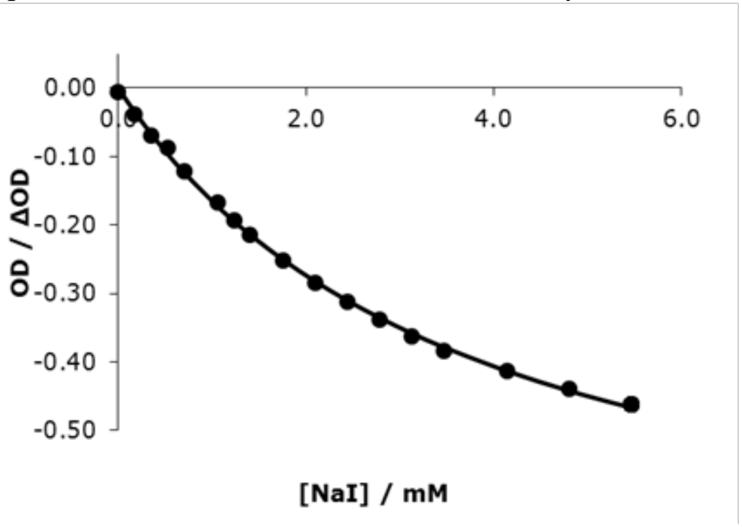

Graph S29. Binding isotherms for titration using 1:1 fitting program for titration of NaI against Reichardt's dye in acetone. [Reichardt's dye] = 0.20 mM and [NaI] = 142 mM, ΔOD = -0.777.

K_a = 320 ± 120 M^{-1} 60% bound

3. Titration Curves for Reichardt's dye (1) in Acetone

a) Titration of Reichardt's dye with 4-phenylazophenol in acetone

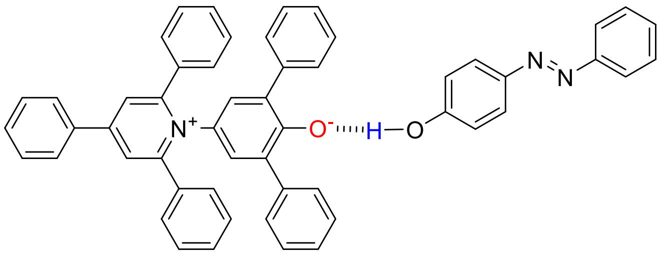

Host: Reichardt's dye = 0.17 mM
Guest: 4-phenylazophenol = 21 mM

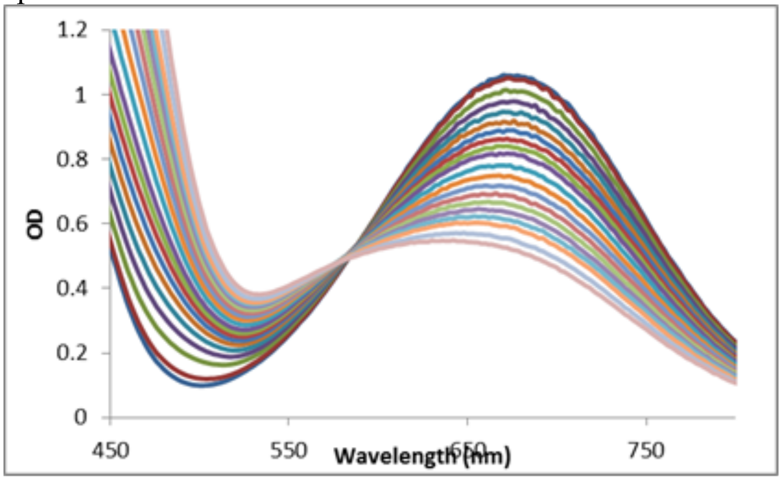

Graph S30. UV/vis spectra of titration of Reichardt's dye with 4-phenylazophenol in acetone.

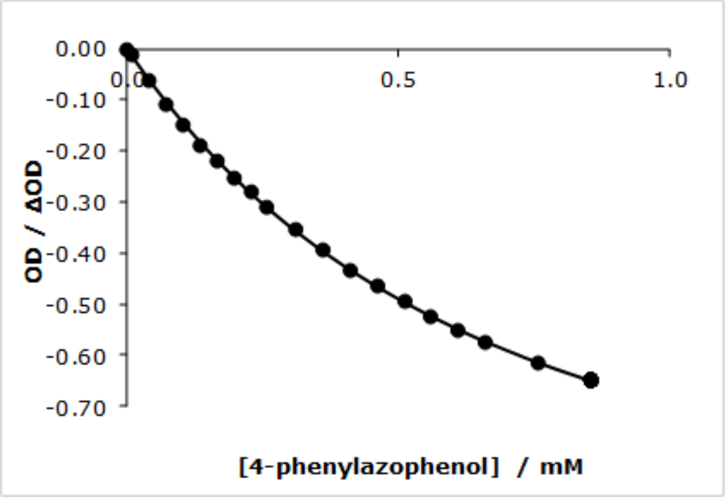

Graph S31. Binding isotherms for titration using 1:1 fitting program for titration of 4-phenylazophenol against Reichardt's dye in acetone. [Reichardt's dye] = 0.17 mM and [4-phenylazophenol] = 21 mM, ΔOD = -1.124.

K_a = 2000 ± 500 M^{-1} 58% boun

b) <u>Titration of Reichardt's dye with 5-nitroindole in acetone</u>

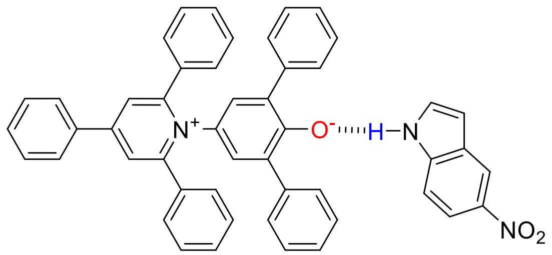

Host: Reichardt's dye = 0.15 mM
Guest: 5-nitroindole = 12 mM

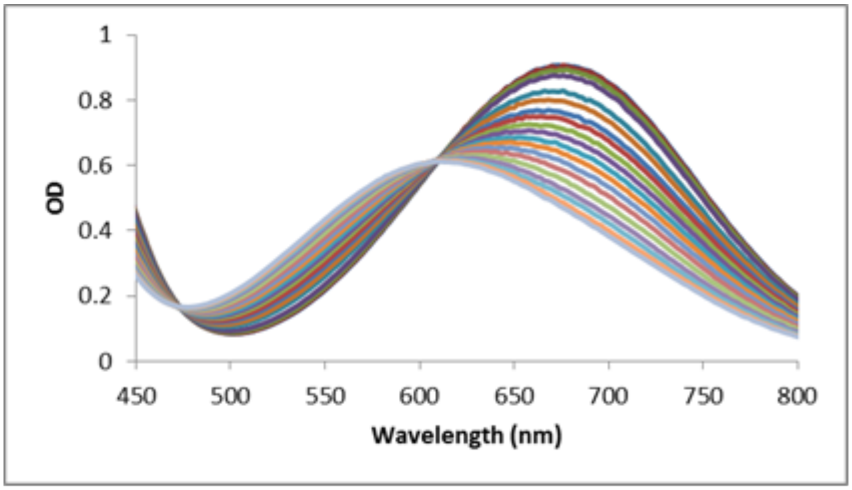

Graph S32. UV/vis spectra of titration of Reichardt's dye with 5-nitroindole in acetone.

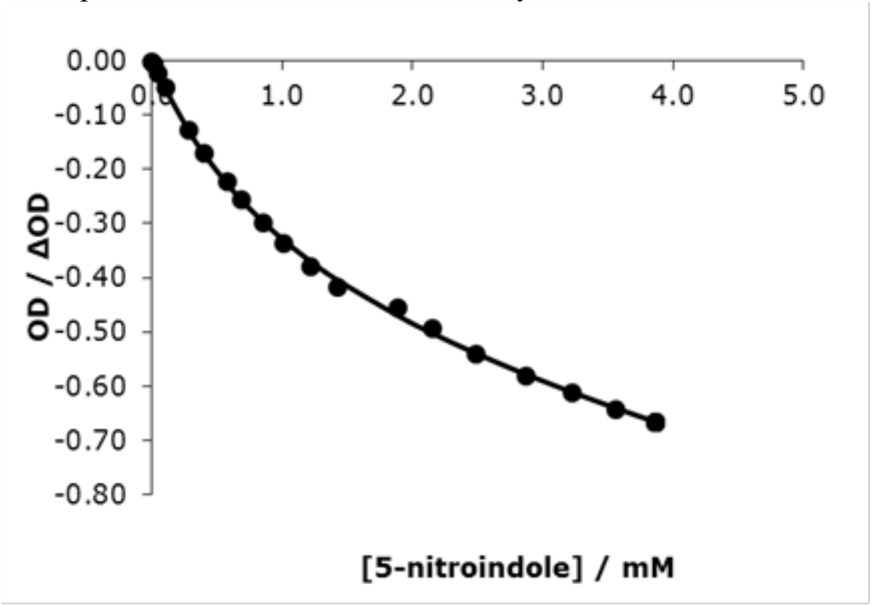

Graph S33. Binding isotherms for titration using 1:1 fitting program for titration of 5-nitroindole against Reichardt's dye in acetone. [Reichardt's dye] = 0.15 mM and [5-nitroindole] = 12 mM, ΔOD = -0.634.

K_a = 860 ± 350 M^{-1} 79% bound

c) <u>Titration of Reichardt's dye with phenol in acetone</u>

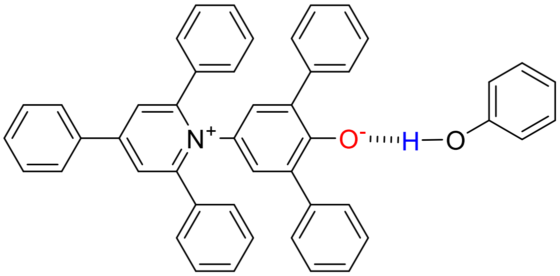

Host: Reichardt's dye = 0.15 mM
Guest: phenol = 108 mM

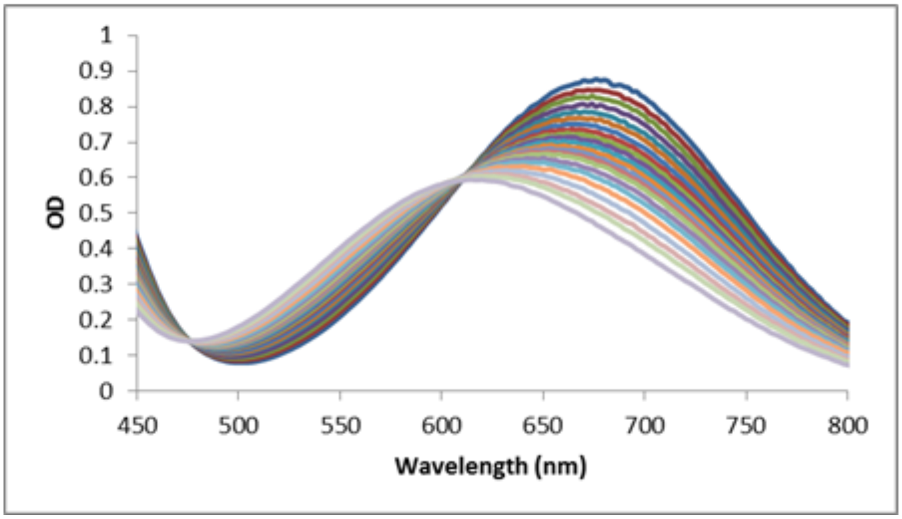

Graph S34. UV/vis spectra of titration of phenol with Reichardt's dye in acetone.

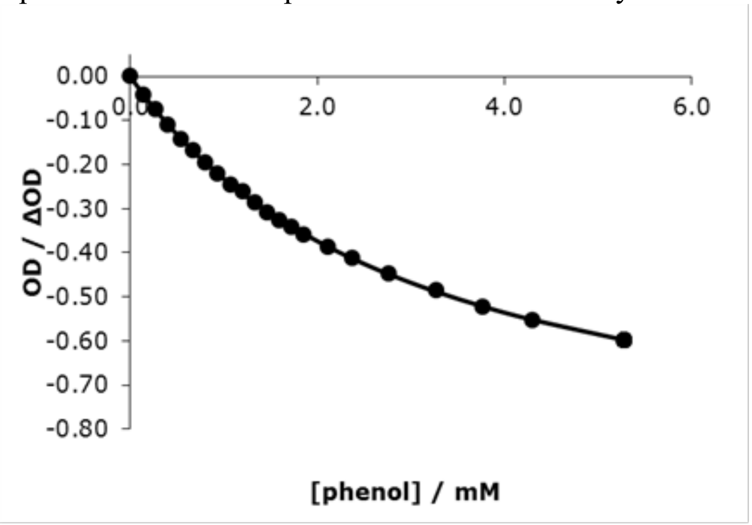

Graph S35. Binding isotherms for titration using 1:1 fitting program for titration of phenol against Reichardt's dye in acetone. [Reichardt's dye] = 0.15 mM and [phenol] = 108 mM, ΔOD = -0.932

K_a = 340 ± 6 M^{-1} 64% bound

d) Titration of Reichardt's dye with $NaBPh_4$ in acetone

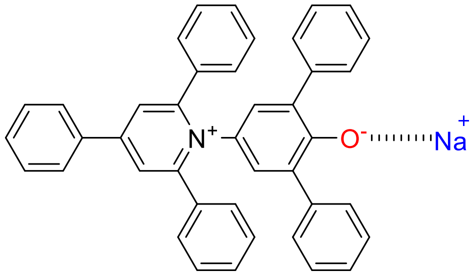

Host: Reichardt's dye = 0.15 mM
Guest: $NaBPh_4$ = 26 mM

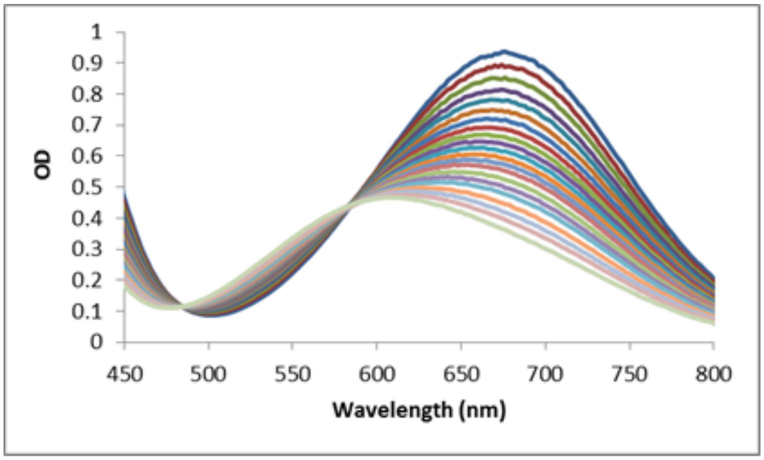

Graph S36. UV/vis spectra of titration of $NaBPh_4$ with Reichardt's dye in acetone.

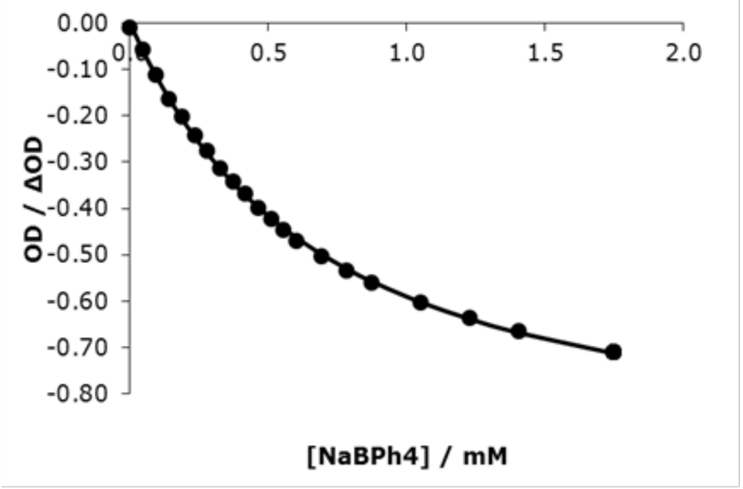

Graph S37. Binding isotherms for titration using 1:1 fitting program for titration of $NaBPh_4$ against Reichardt's dye in acetone. [Reichardt's dye] = 0.15 mM and [$NaBPh_4$] = 26 mM, ΔOD = -0.959.

K_a = 1700 ± 160 M^{-1} 74% bound

e) Titration of Reichardt's dye with $RbBPh_4$ in acetone

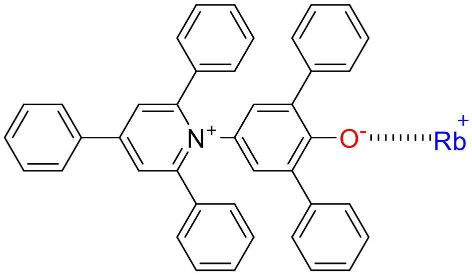

Host: Reichardt's dye = 0.15 mM
Guest: $RbBPh_4$ = 28 mM

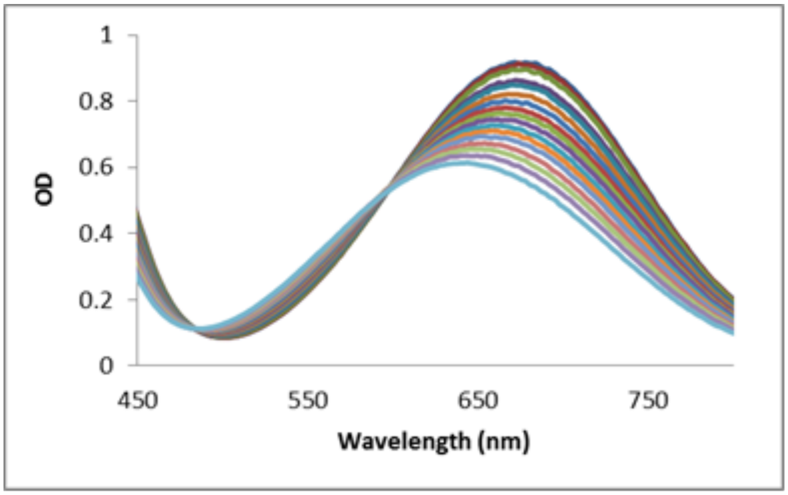

Graph S38. UV/vis spectra of titration of $RbBPh_4$ with Reichardt's dye in acetone.

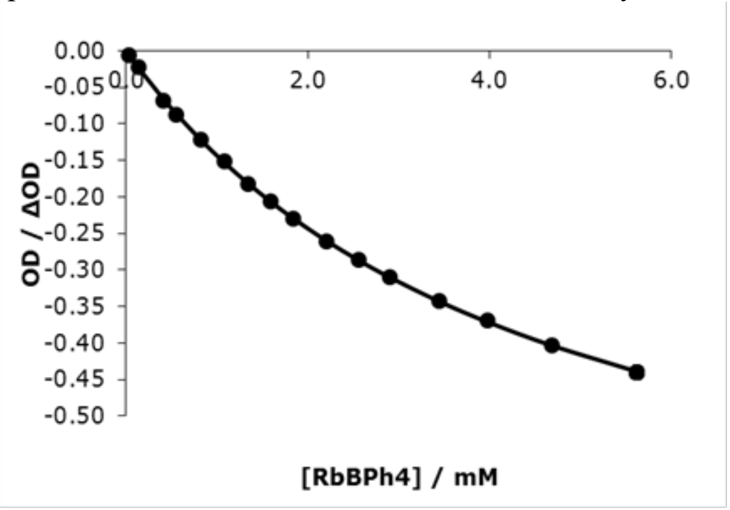

Graph S39. Binding isotherms for titration using 1:1 fitting program for titration of $RbBPh_4$ against Reichardt's dye in acetone. [Reichardt's dye] = 0.15 mM and [$RbBPh_4$] = 28 mM, ΔOD = -0.782.

K_a = 220 ± 50 M^{-1} 56% bound

f) Titration of Reichardt's dye with $CsBPh_4$ in acetone

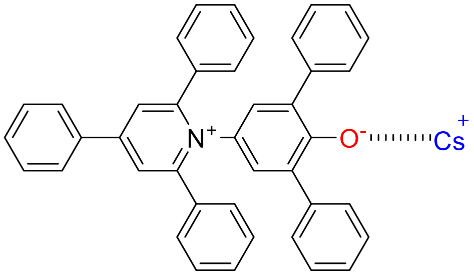

Host: Reichardt's dye = 0.15 mM
Guest: $CsBPh_4$ = 24 mM

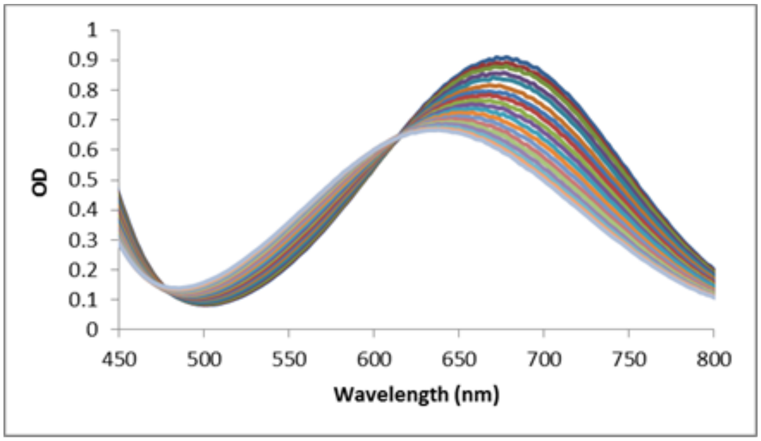

Graph S40. UV/vis spectra of titration of $CsBPh_4$ with Reichardt's dye in acetone.

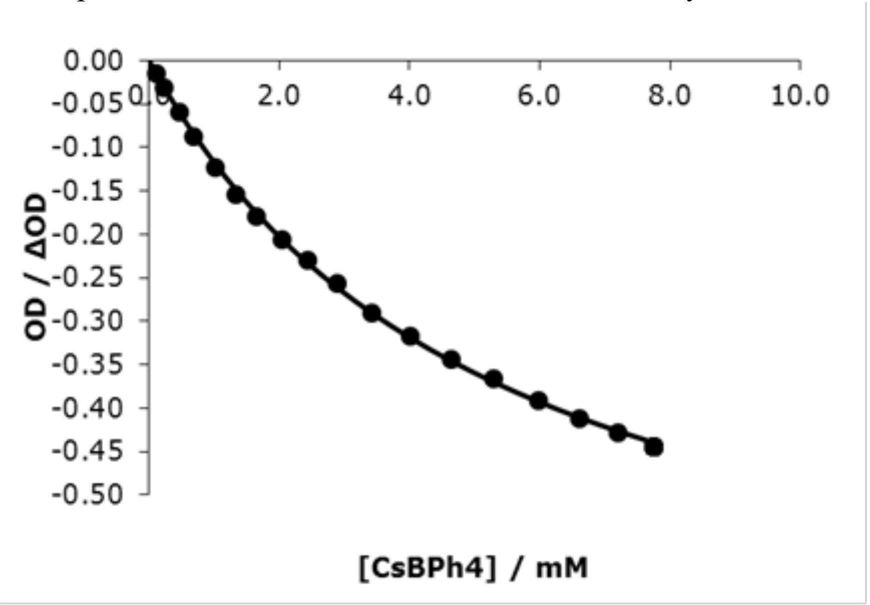

Graph S41. Binding isotherms for titration using 1:1 fitting program for titration of $CsBPh_4$ against Reichardt's dye in acetone. [Reichardt's dye] = 0.15 mM and [$CsBPh_4$] = 24 mM, ΔOD = -0.734.

K_a = 210 ± 35 M^{-1} 60% bound

g) Titration of Reichardt's dye with IPh_2BF_4 in acetone

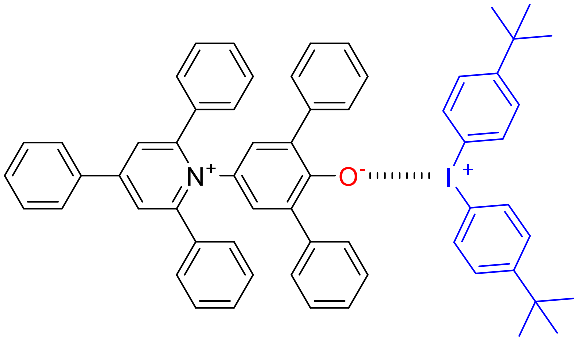

Host: Reichardt's dye = 0.15 mM
Guest: IPh_2BF_4 = 11 mM

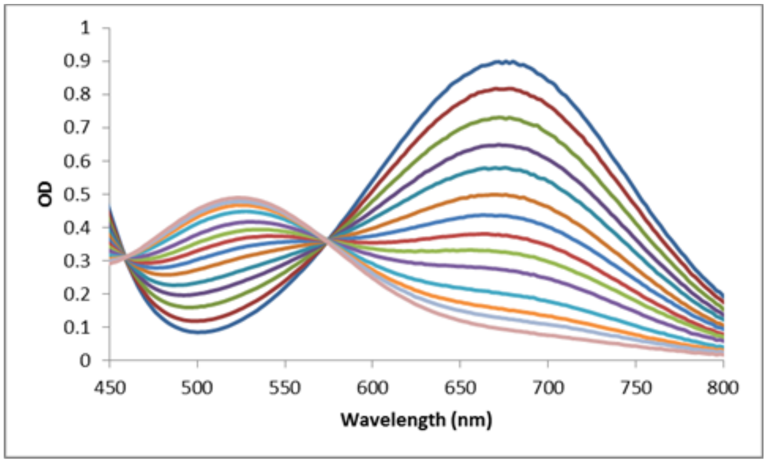

Graph S42. UV/vis spectra of titration of $IPh_2BF_4$with Reichardt's dye in acetone.

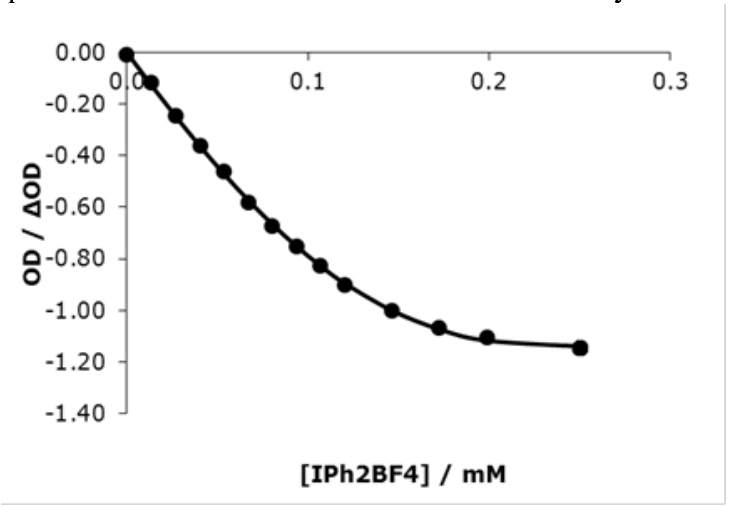

Graph S43. Binding isotherms for titration using 1:1 fitting program for titration of phenol against Reichardt's dye in acetone. [Reichardt's dye] = 0.15 mM and [IPh_2BF_4] = 11 mM, ΔOD = -1.777.

K_a = 48000 ± 3000 M^{-1} 79% bound

h) Titration of Reichardt's dye with $KBPh_4$ in acetone

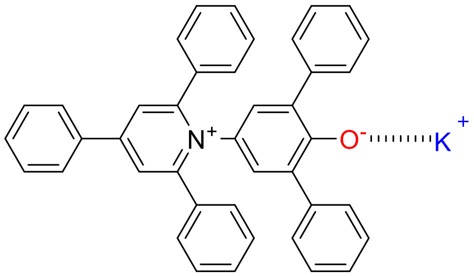

Host: Reichardt's dye = 0.15 mM
Guest: $KBPh_4$ = 19 mM

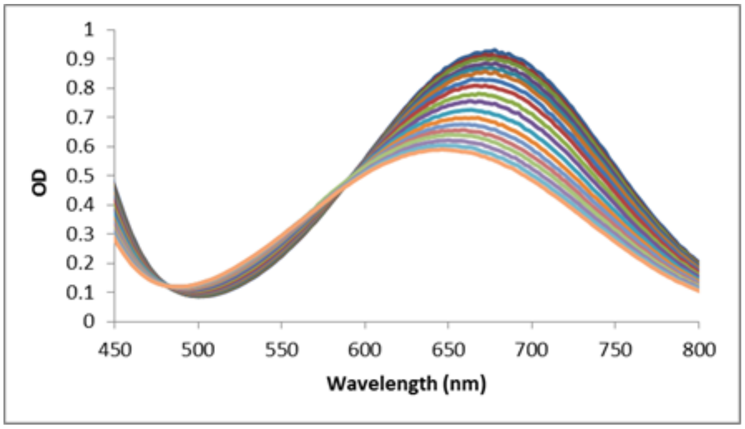

Graph S44. UV/vis spectra of titration of $KBPh_4$ with Reichardt's dye in acetone.

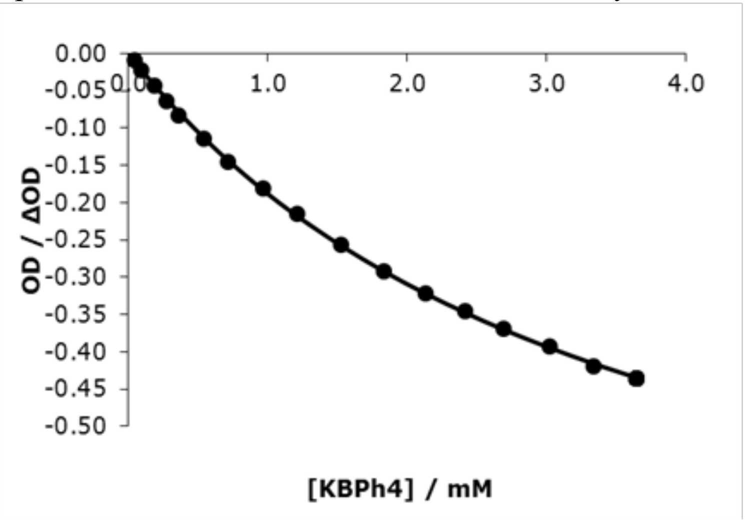

Graph S45. Binding isotherms for titration using 1:1 fitting program for titration of $KBPh_4$ against Reichardt's dye in acetone. [Reichardt's dye] = 0.15 mM and [$KBPh_4$] = 19 mM, ΔOD = -0.846.

K_a = 270 ± 75 M^{-1} **51% bound**

i) Titration of Reichardt's dye with 1-butyl-4-methylpyridiniumPF_6 in acetone

Host: Reichardt's dye = 0.15 mM
Guest: 1-butyl-4-methylpyridiniumPF_6 = 252 mM

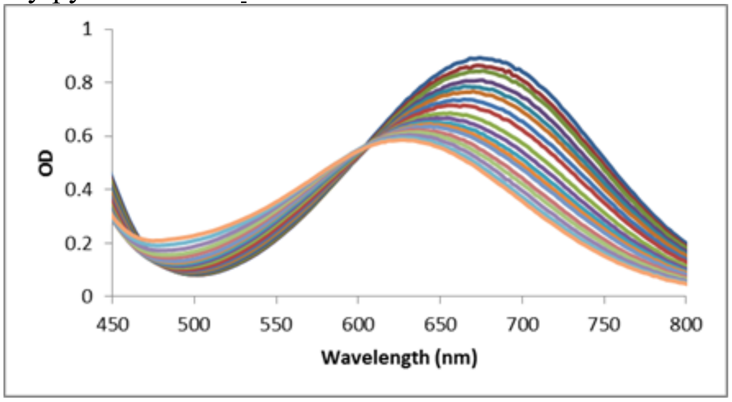

Graph S46. UV/vis spectra of titration of1-butyl-4-methylpyridiniumPF_6 with Reichardt's dye in acetone.

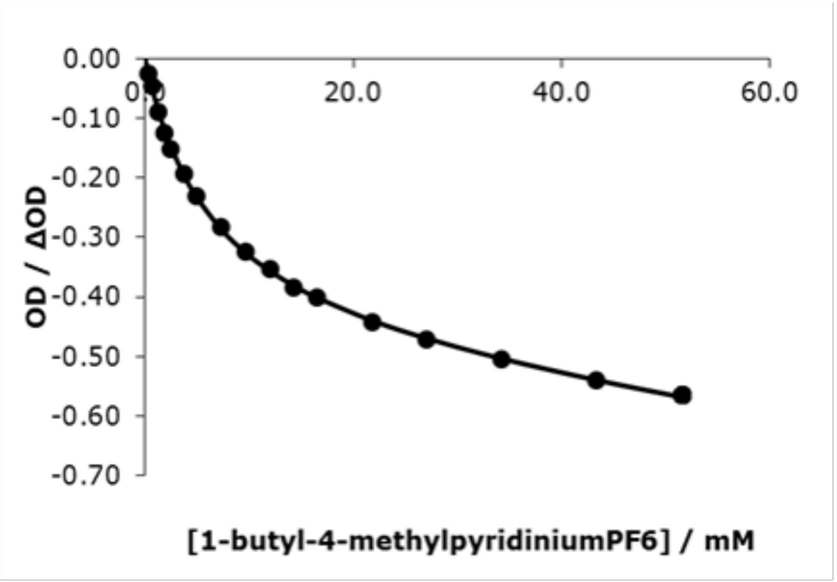

Graph S47. Binding isotherms for titration using 1:1 fitting program for titration of 1-butyl-4-methylpyridiniumPF_6 against Reichardt's dye in acetone. [Reichardt's dye] = 0.15 mM and [1-butyl-4-methylpyridiniumPF_6] = 252 mM, ΔOD = -0.640.

.

K_a = 180 ± 40 M^{-1} 84% bound

j) Titration of Reichardt's dye with imidazoliumPF_6 in acetone

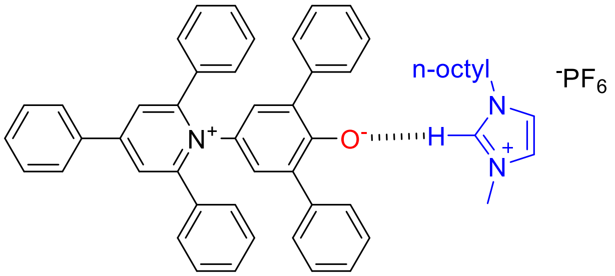

Host: Reichardt's dye = 0.15 mM
Guest: imidazoliumPF_6 = 144 mM

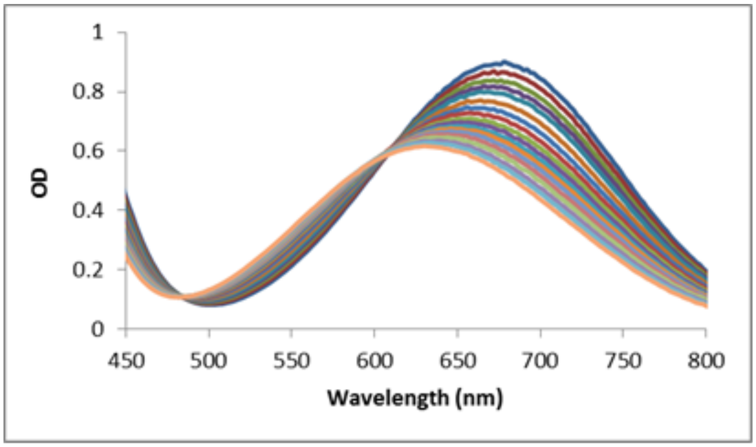

Graph S48. UV/vis spectra of titration of imidiazoliumPF_6 with Reichardt's dye in acetone.

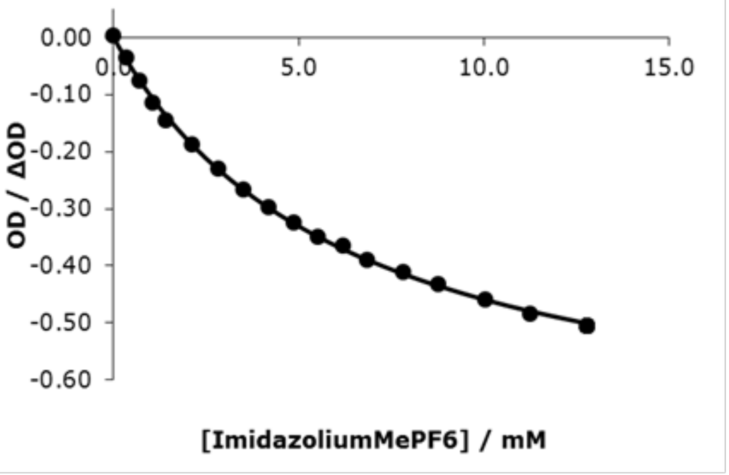

Graph S49. Binding isotherms for titration using 1:1 fitting program for titration of imidazoliumPF_6 against Reichardt's dye in acetone. [Reichardt's dye] = 0.15 mM and [imidiazoliumPF_6] = 144 mM, ΔOD = -0.745.

K_a = 200 ± 60 M^{-1} **67% bound**

k) Titration of Reichardt's dye with Bu_4NBF_4 in acetone

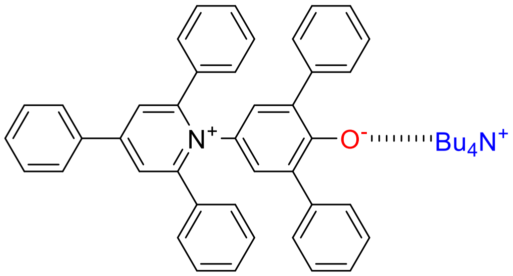

Host: Reichardt's dye = 0.15 mM
Guest: Bu_4NBF_4 = 334 mM

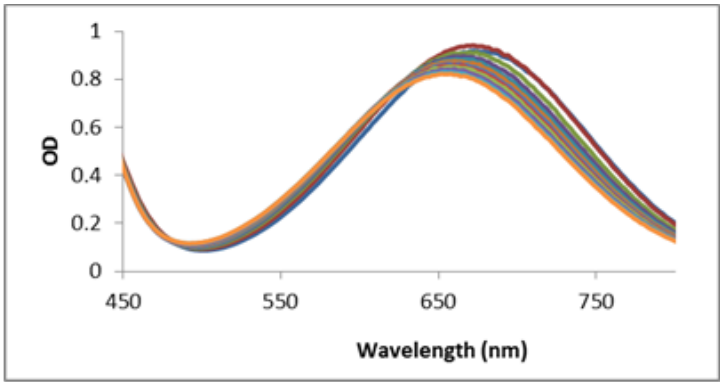

Graph S50. UV/vis spectra of titration of Bu_4NBF_4 with Reichardt's dye in acetone.

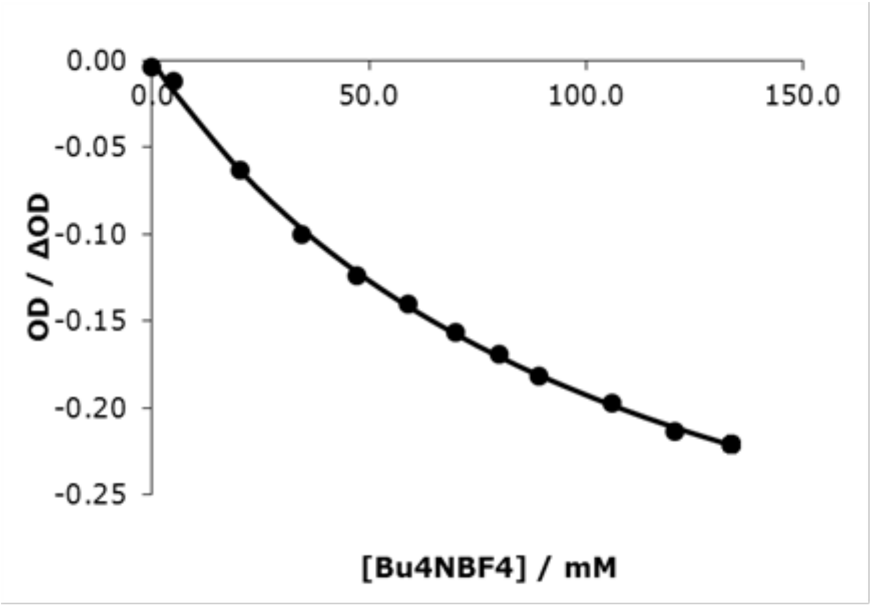

Graph S51. Binding isotherms for titration using 1:1 fitting program for titration of Bu_4NBF_4 against Reichardt's dye in acetone. [Reichardt's dye] = 0.15 mM and [Bu_4NBF_4] = 334 mM, ΔOD = -0.401.

K_a = 10 ± 2 M^{-1} 55% bound

l) Titration of Reichardt's dye with $NaBF_4$ in acetone

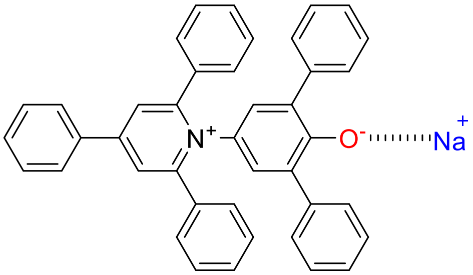

Host: Reichardt's dye = 0.15 mM
Guest: $NaBF_4$ = 96 mM

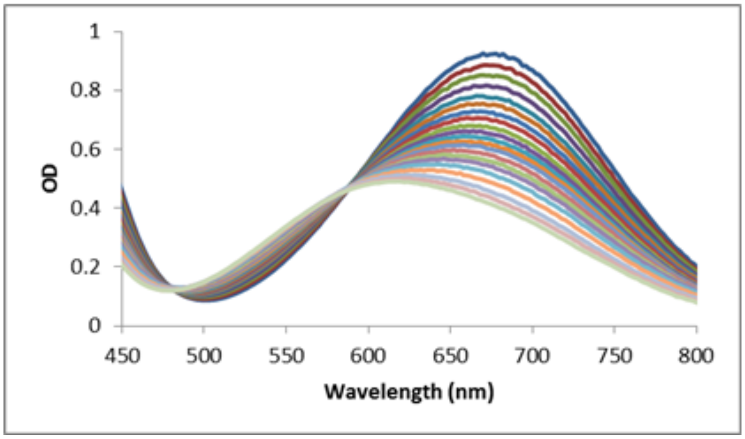

Graph S52. UV/vis spectra of titration of $NaBF_4$ with Reichardt's dye in acetone.

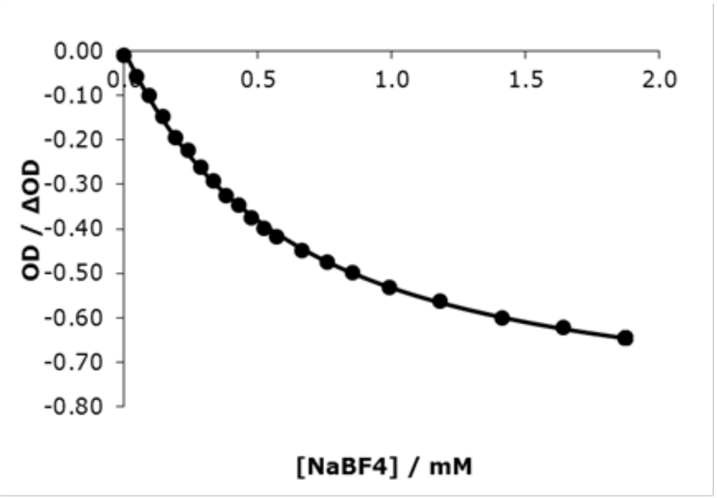

Graph S53. Binding isotherms for titration using 1:1 fitting program for titration of $NaBF_4$ against Reichardt's dye in acetone. [Reichardt's dye] = 0.15 mM and [$NaBF_4$] = 96 mM, ΔOD = -0.834.

K_a = 1900 ± 100 M^{-1} **77% bound**

m) Titration of Reichardt's dye with $NaPF_6$ in acetone

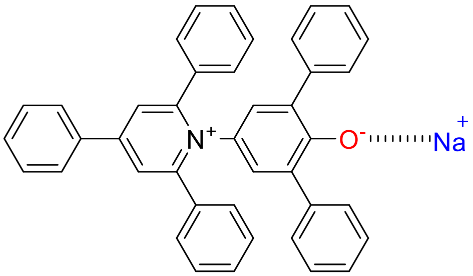

Host: Reichardt's dye = 0.15 mM
Guest: $NaPF_6$ = 59 mM

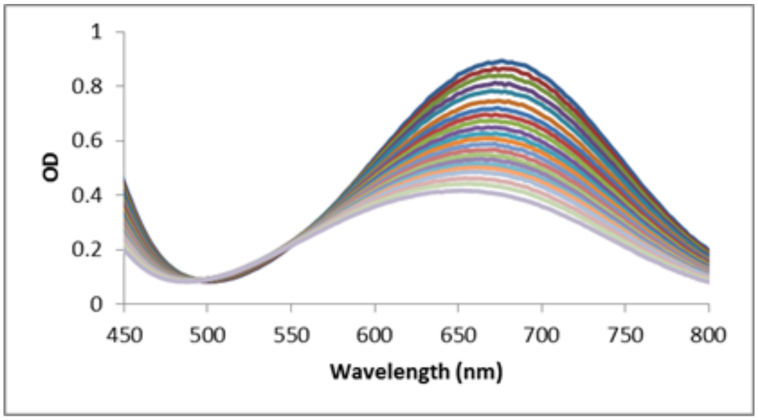

Graph S54. UV/vis spectra of titration of $NaPF_6$ with Reichardt's dye in acetone.

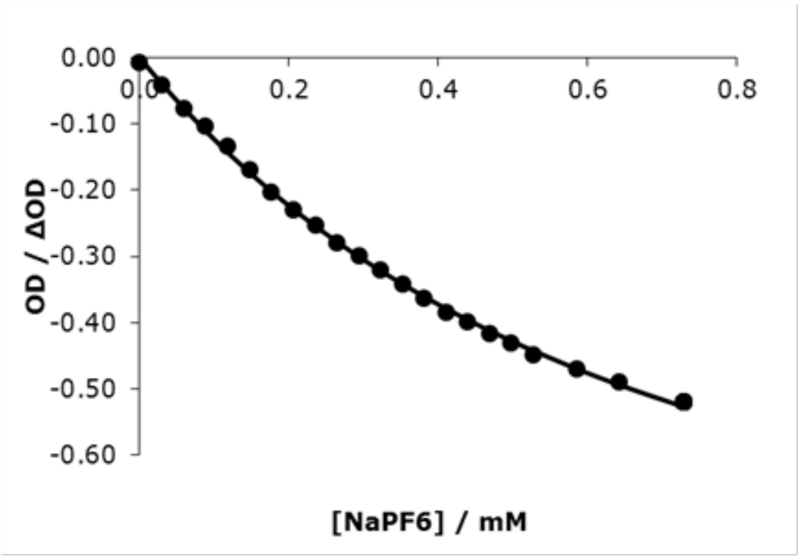

Graph S55. Binding isotherms for titration using 1:1 fitting program for titration of $NAPF_6$ against Reichardt's dye in acetone. [Reichardt's dye] = 0.15 mM and [$NaPF_6$] = 59 mM, ΔOD = -0.989.

K_a = 1700 ± 140 M^{-1} **53% bound**

n) <u>Titration of Reichardt's dye with NaBAr^F in acetone</u>

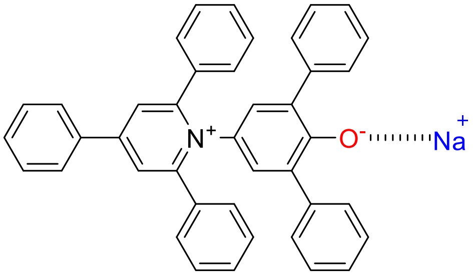

Host: Reichardt's dye = 0.15 mM
Guest: NaBAr^F = 35 mM

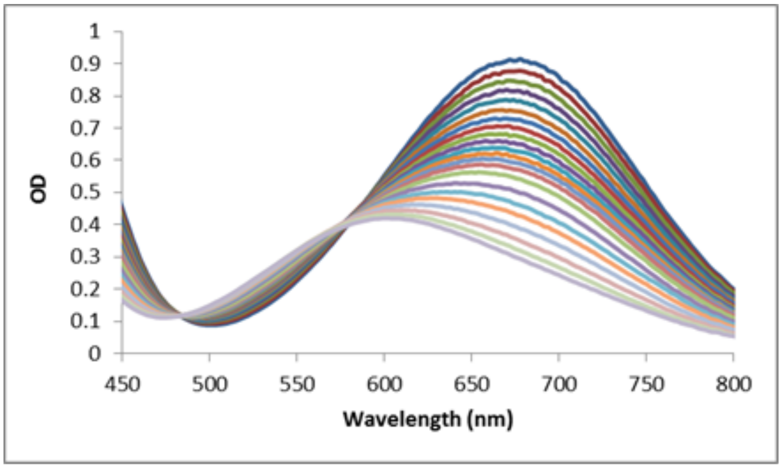

Graph S56. UV/vis spectra of titration of NaBAr^F with Reichardt's dye in acetone.

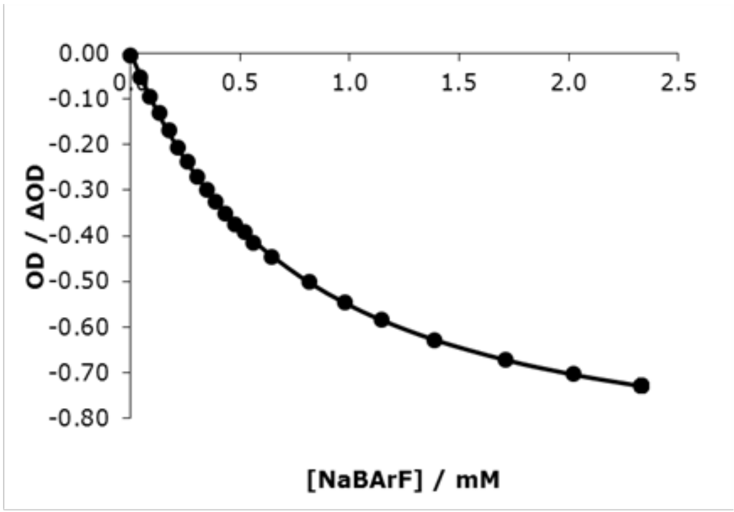

Graph S57. Binding isotherms for titration using 1:1 fitting program for titration of phenol against Reichardt's dye in acetone. [Reichardt's dye] = 0.15 mM and [NaBAr^F] = 35 mM, ΔOD = -0.943.

K_a = 1400 ± 400 M^{-1} 71% bound

o) <u>Titration of Reichardt's dye with $NaN(SO_2CF_3)_2$ in acetone</u>

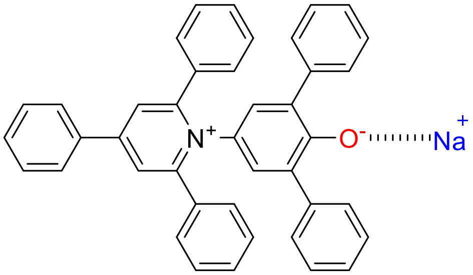

Host: Reichardt's dye = 0.15 mM
Guest: $NaN(SO_2CF_3)_2$ = 46 mM

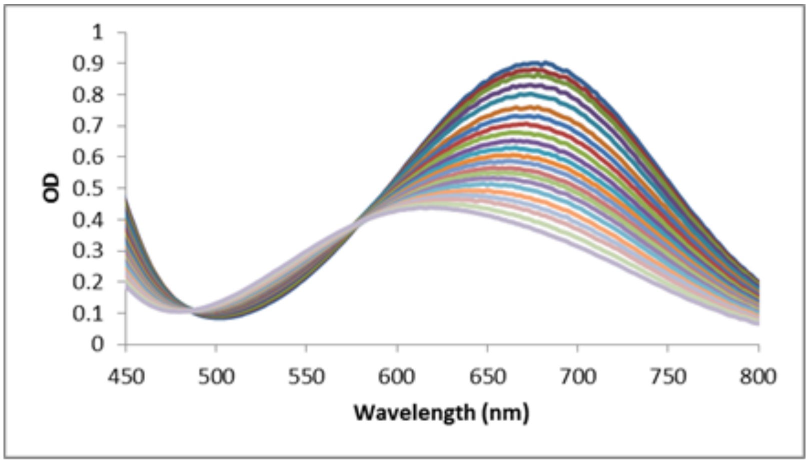

Graph S58. UV/vis spectra of titration of $NaN(SO_2CF_3)_2$ with Reichardt's dye in acetone.

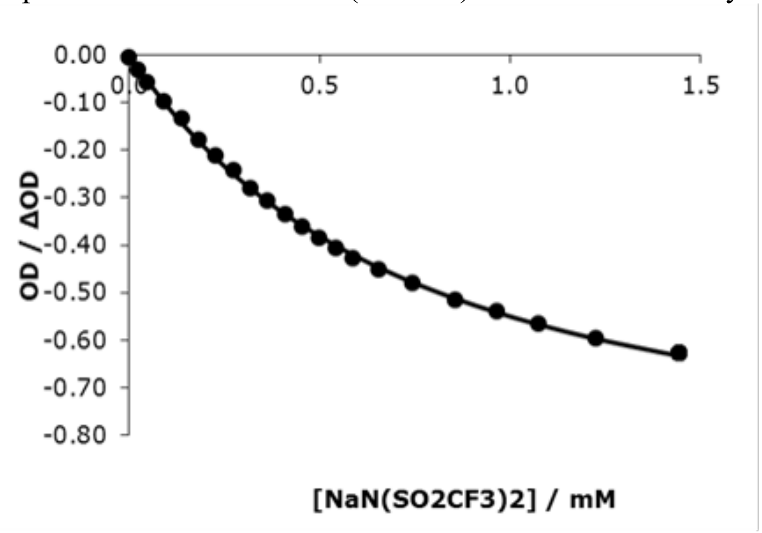

Graph S59. Binding isotherms for titration using 1:1 fitting program for titration of phenol against Reichardt's dye in acetone. [Reichardt's dye] = 0.15 mM and [$NaN(SO_2CF_3)_2$] = 46 mM, ΔOD = -0.930.

K_a = 1500 ± 200 M^{-1} 68% bound

p) <u>Titration of Reichardt's dye with NaI in acetone</u>

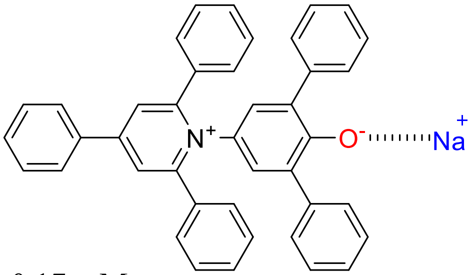

Host: Reichardt's dye = 0.17 mM
Guest: NaI = 184 mM

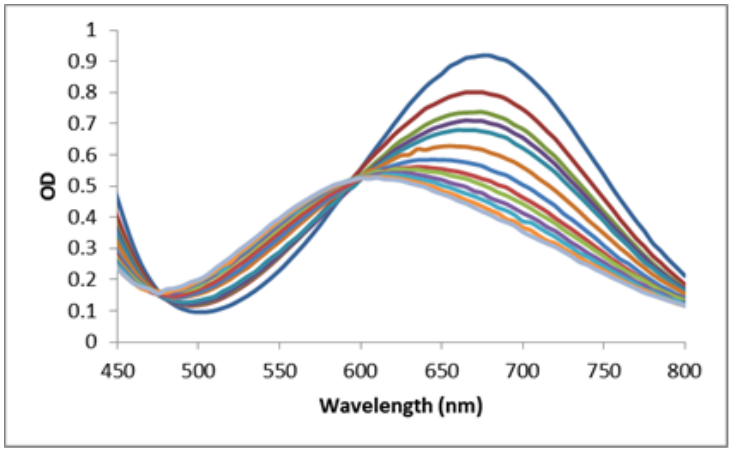

Graph S60. UV/vis spectra of titration of NaI with Reichardt's dye in acetone.

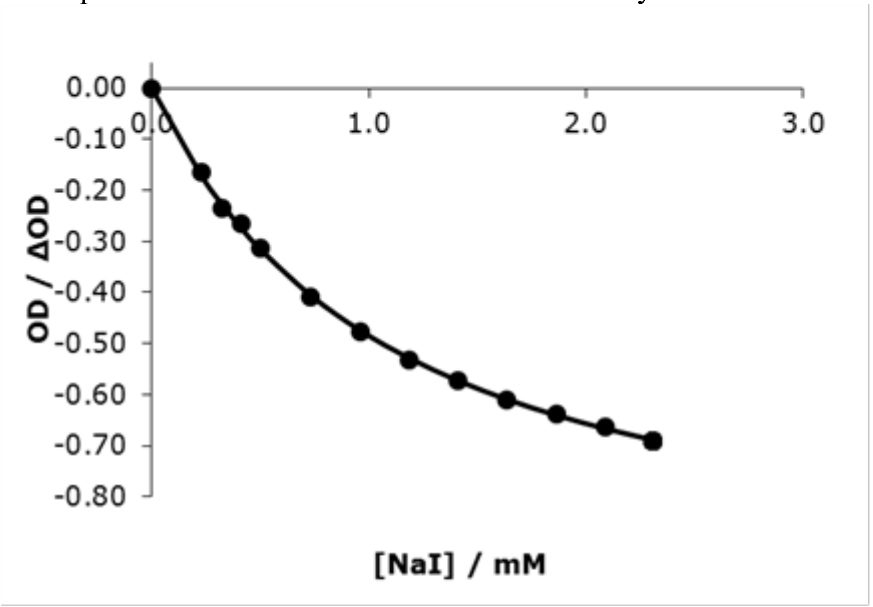

Graph S61. Binding isotherms for titration using 1:1 fitting program for titration of NaI against Reichardt's dye in acetone. [Reichardt's dye] = 0.17 mM and [NaI] = 183 mM, ΔOD = -0.987.

K_a = 1000 ± 300 M^{-1} 70% bound

q) <u>Titration of Reichardt's dye with TFA in acetone</u>

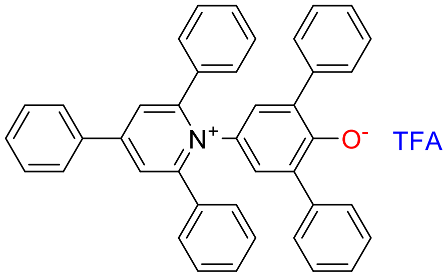

Host: Reichardt's dye = 0.21 mM

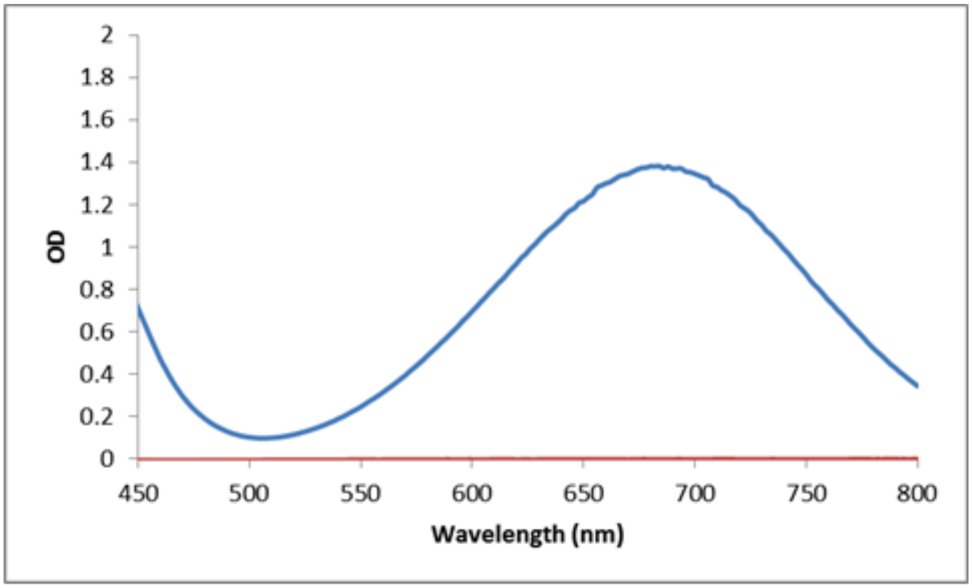

Graph S62. UV/vis spectra of Reichardt's dye when treated with 100 µL of TFA in acetonitrile.

4. Titration Curves for Reichardt's dye (1) in Acetonitrile with 0.1% H_2O added

a) Titration of Reichardt's dye with phenol in acetonitrile + 0.1% H_2O added

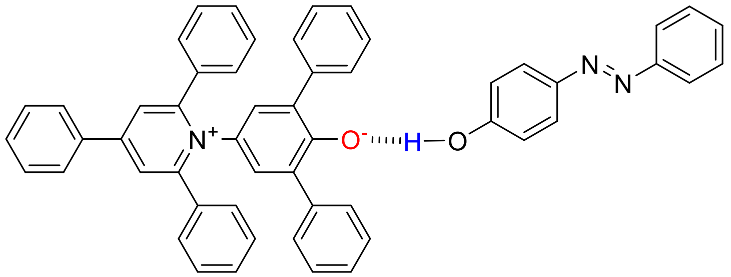

Host: Reichardt's dye = 0.21 mM

Guest: 4-phenylazophenol = 18 mM

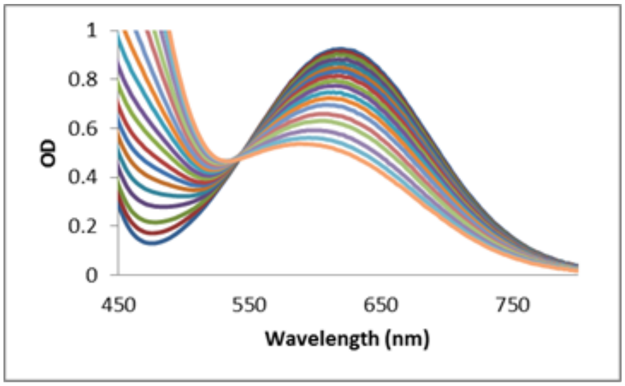

Graph S63. UV/vis spectra of titration of Reichardt's dye with 4-phenylazophenol in acetonitrile with 0.1% H_2O added.

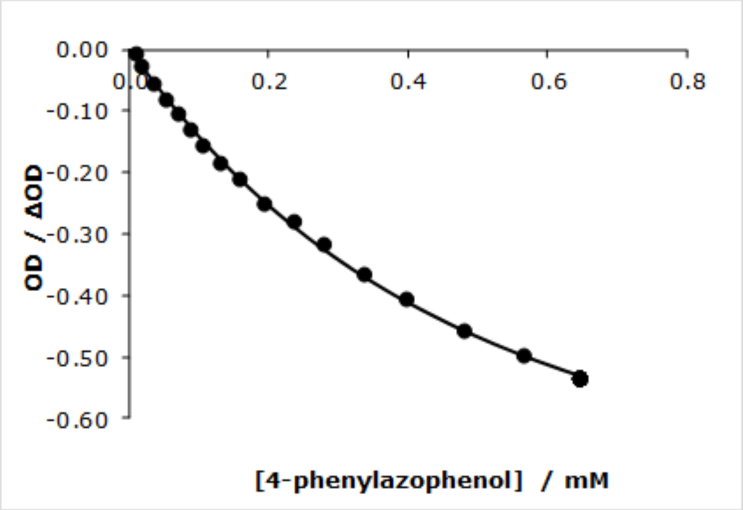

Graph S64. Binding isotherms for titration using 1:1 and accounting for a non-specific secondary interaction fitting program for titration of 4-phenylazophenol against Reichardt's dye in acetonitrile with 0.1% H_2O added. [Reichardt's dye] = 0.21 mM and [4-phenylazophenol] = 18 mM, ΔOD = -0.887.

K_a = 2700 ± 560 M^{-1} 60% bound

b) Titration of Reichardt's dye with guanidinium.BPh_4 in acetonitrile with 0.1% H_2O added

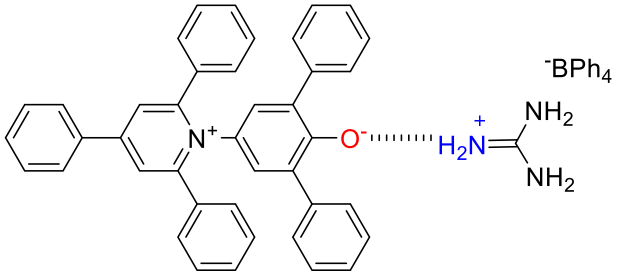

Host: Reichardt's dye = 0.21 mM
Guest: guanidinium.BPh_4 = 45 mM

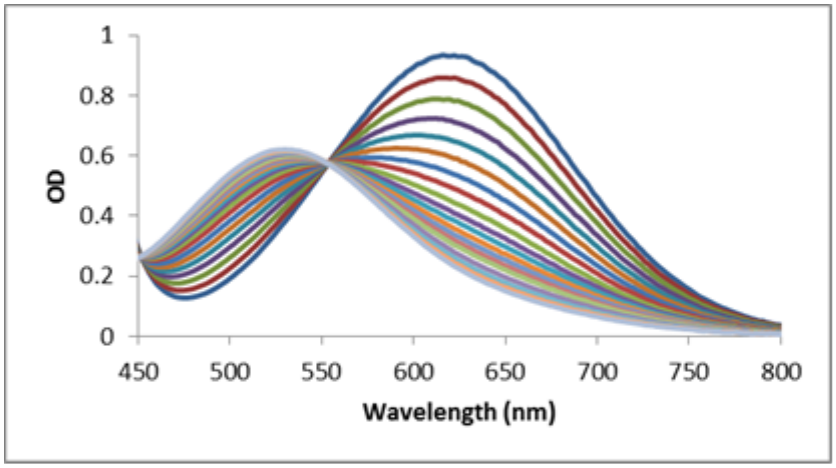

Graph S65. UV/vis spectra of titration of guanidinium.BPh_4 with Reichardt's dye in acetonitrile with 0.1% H_2O added.

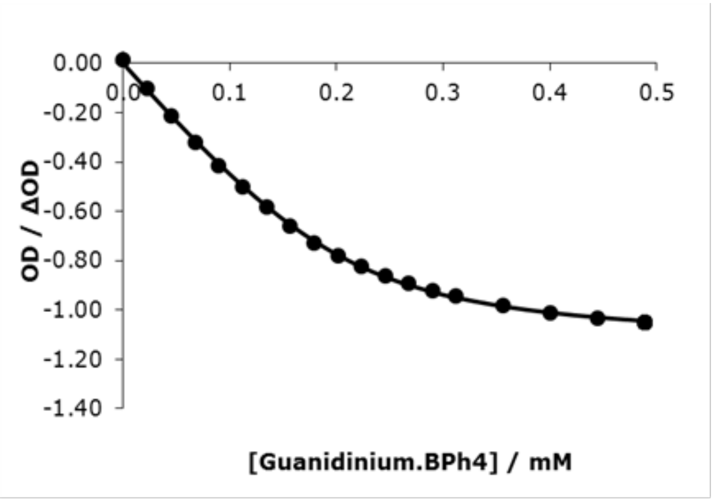

Graph S66. Binding isotherms for titration using 1:1 fitting program for titration of phenol against Reichardt's dye in acetonitrile with 0.1% H_2O added. [Reichardt's dye] = 0.21 mM and [guanidinium.BPh_4] = 45 mM, ΔOD = -1.143.

$\boldsymbol{K_a}$ **= 34000 ± 3800 M**$^{-1}$ **91% bound**

c) <u>Titration of Reichardt's dye with $NaN(SO_2CF_3)_2$ in acetonitrile with 0.1% H_2O added</u>

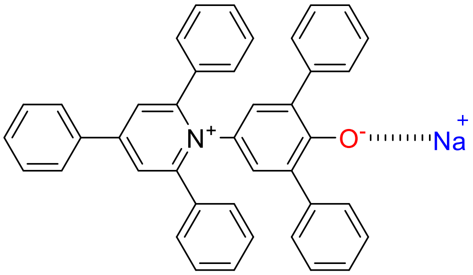

Host: Reichardt's dye = 0.21 mM
Guest: $NaN(SO_2CF_3)_2$ = 84 mM

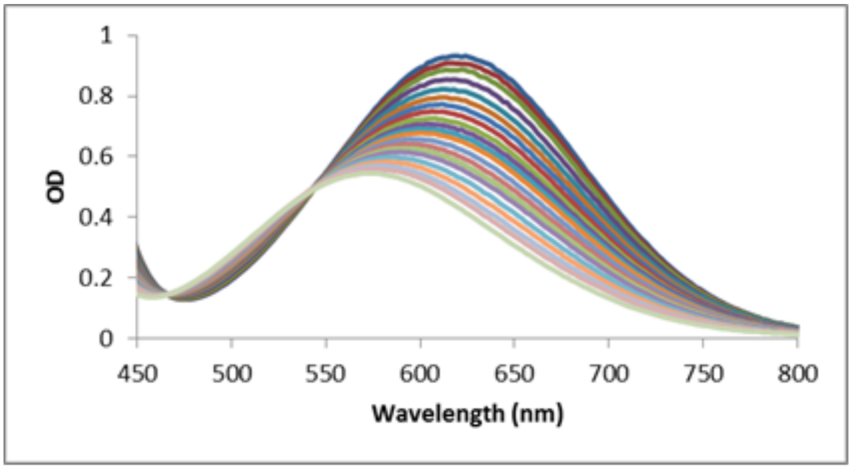

Graph S67. UV/vis spectra of titration of $NaN(SO_2CF_3)_2$ with Reichardt's dye in acetonitrile with 0.1% H_2O added.

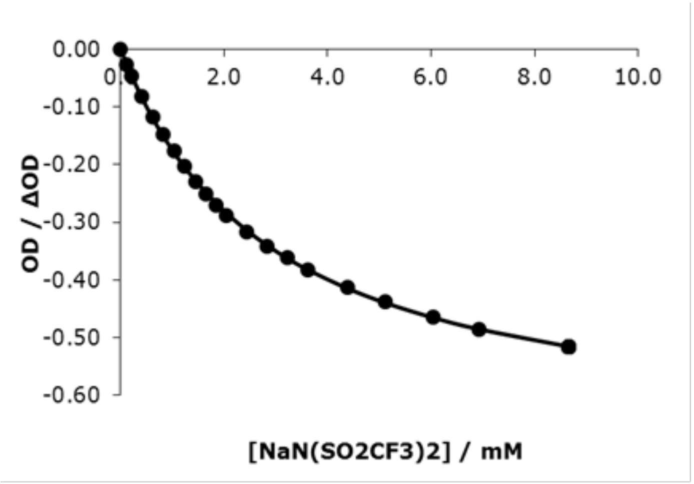

Graph S68. Binding isotherms for titration using 1:1 fitting program for titration of phenol against Reichardt's dye in acetonitrile with 0.1% H_2O added. [Reichardt's dye] = 0.21 mM and [$NaN(SO_2CF_3)_2$] = 84 mM, ΔOD = -0.684.

K_a = 380 ± 38 M^{-1} 75% bound

5. Titration Curves for Reichardt's dye (1) in Acetonitrile with 0.25% H_2O added

a) Titration of Reichardt's dye with phenol in acetonitrile + 0.25% H_2O added

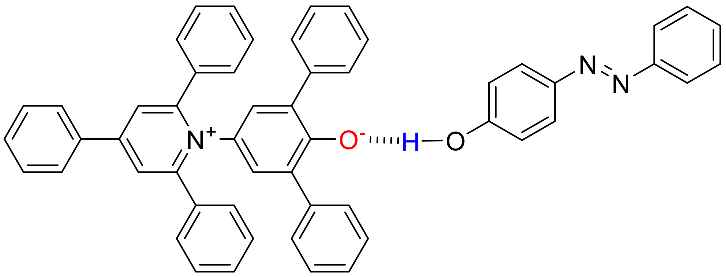

Host: Reichardt's dye = 0.24 mM

Guest: 4-phenylazophenol = 24 mM

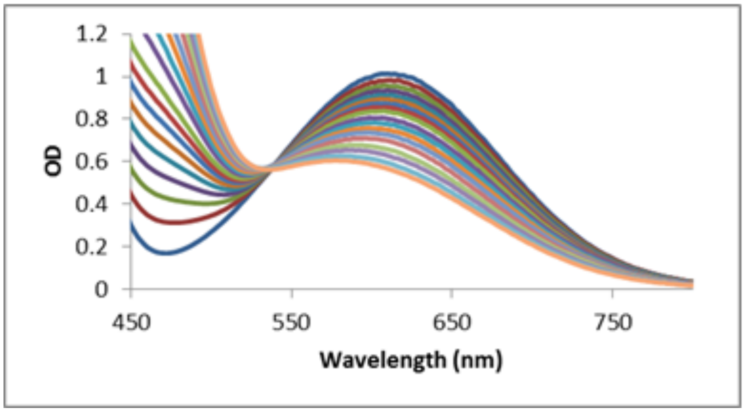

Graph S69. UV/vis spectra of titration of Reichardt's dye with 4-phenylazophenol in acetonitrile with 0.25% H_2O added.

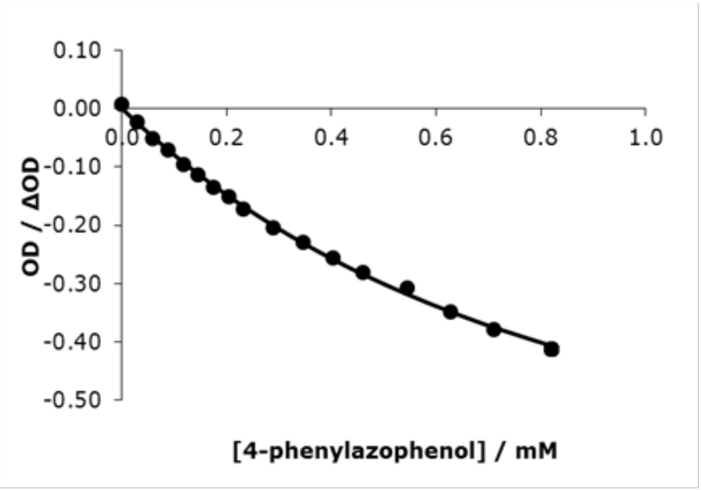

Graph S70. Binding isotherms for titration using 1:1 and accounting for a non-specific secondary interaction fitting program for titration of 4-phenylazophenol against Reichardt's dye in acetonitrile with 0.25% H_2O added. [Reichardt's dye] = 0.24 mM and [4-phenylazophenol] = 24 mM, ΔOD = -0.816.

K_a = 1600 ± 380 M^{-1} 50% bound

b) Titration of Reichardt's dye with guanidinium.BPh_4 in acetonitrile with 0.25% H_2O added

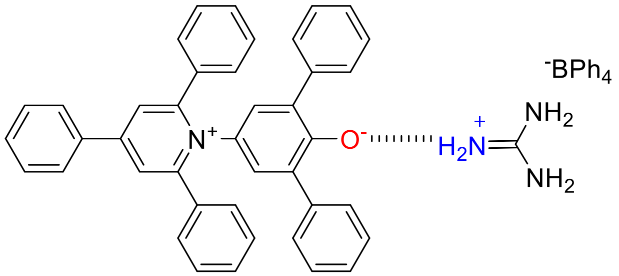

Host: Reichardt's dye = 0.24 mM
Guest: guanidinium.BPh_4 = 49 mM

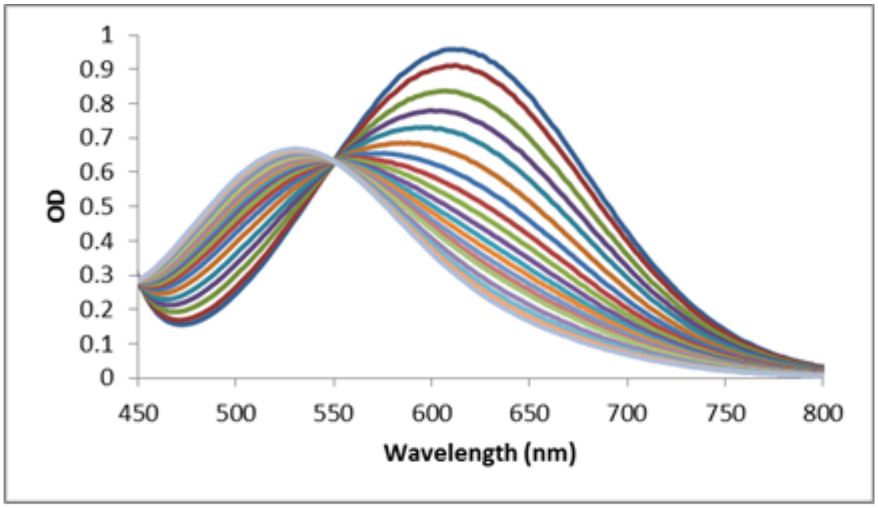

Graph S71. UV/vis spectra of titration of guanidinium.BPh_4 with Reichardt's dye in acetonitrile with 0.25% H_2O added.

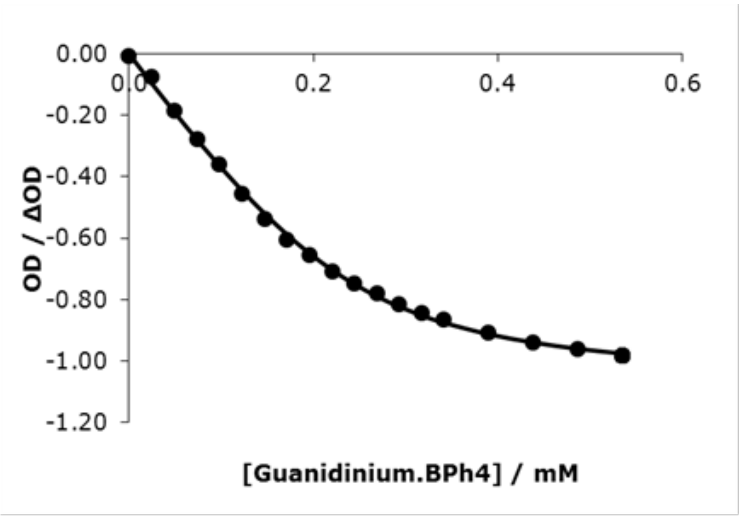

Graph S72. Binding isotherms for titration using 1:1 fitting program for titration of guanidinium.BPh_4 against Reichardt's dye in acetonitrile with 0.25% H_2O added. [Reichardt's dye] = 0.24 mM and [guanidinium.BPh_4] = 49 mM, ΔOD = -1.083.

.

K_a = 27000 ± 2000 M^{-1} 90% bound

c) <u>Titration of Reichardt's dye with $NaN(SO_2CF_3)_2$ in acetonitrile with 0.25% H_2O added</u>

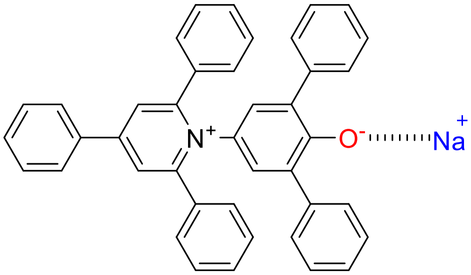

Host: Reichardt's dye = 0.24 mM
Guest: $NaN(SO_2CF_3)_2$ = 70 mM

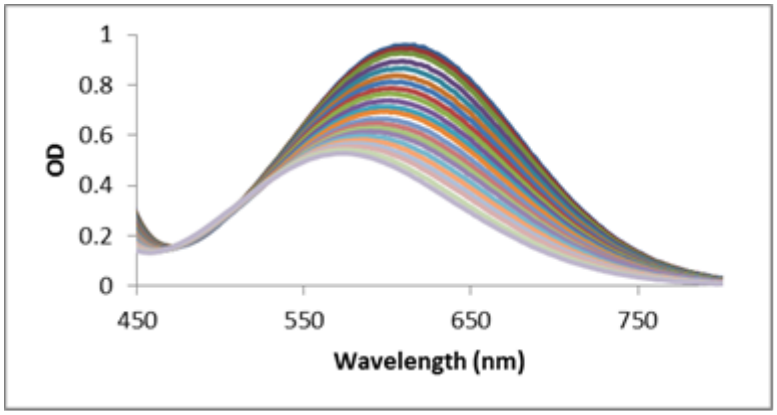

Graph S73. UV/vis spectra of titration of $NaN(SO_2CF_3)_2$ with Reichardt's dye in acetonitrile with 0.25% H_2O added.

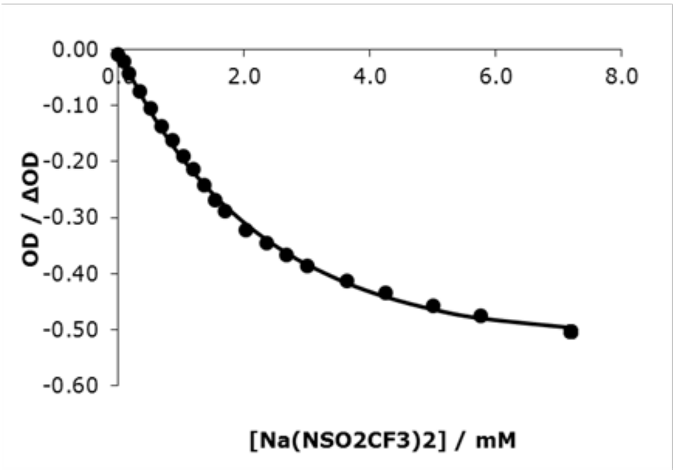

Graph S74. Binding isotherms for titration using 1:1 fitting program and accounting for a second weaker interaction for titration of $NaN(SO_2CF_3)_2$ against Reichardt's dye in acetonitrile with 0.25% H_2O added. [Reichardt's dye] = 0.24 mM and [$NaN(SO_2CF_3)_2$] = 70 mM,

K_a = 290 ± 18 M^{-1} 75% bound

6. Titration Curves for Reichardt's dye (1) in Acetonitrile with 0.5 % H_2O added

a) <u>Titration of Reichardt's dye with phenol in acetonitrile + 0.5% H_2O added</u>

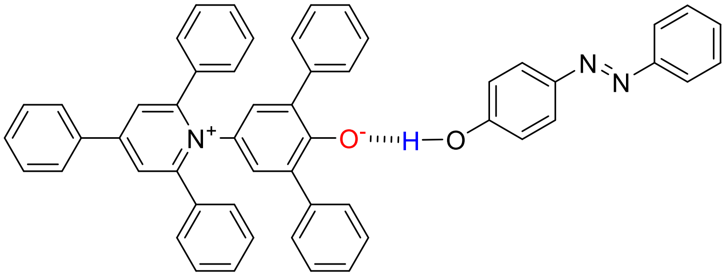

Host: Reichardt's dye = 0.24 mM
Guest: 4-phenylazophenol = 24 mM

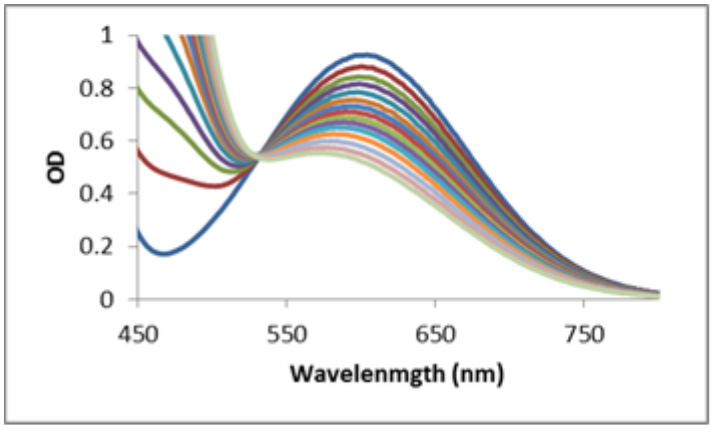

Graph S75. UV/vis spectra of titration of Reichardt's dye with 4-phenylazophenol in acetonitrile with 0.5% H_2O added.

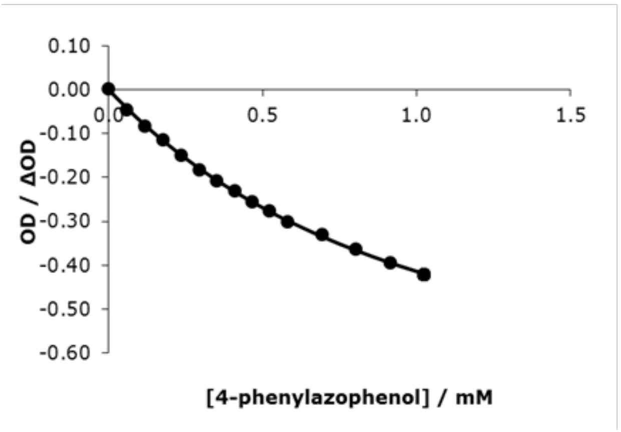

Graph S76. Binding isotherms for titration using 1:1 and accounting for a non-specific secondary interaction fitting program for titration of 4-phenylazophenol against Reichardt's dye in acetonitrile with 0.5% H_2O added. [Reichardt's dye] = 0.24 mM and [4-phenylazophenol] = 24 mM, ΔOD = -0.823.

K_a = 1100 ± 180 M^{-1} 51% bound

b) Titration of Reichardt's dye with guanidinium.BPh_4 in acetonitrile with 0.5% H_2O added

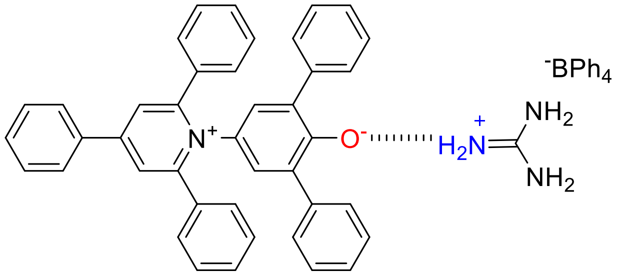

Host: Reichardt's dye = 0.24 mM
Guest: guanidinium.BPh_4 = 41 mM

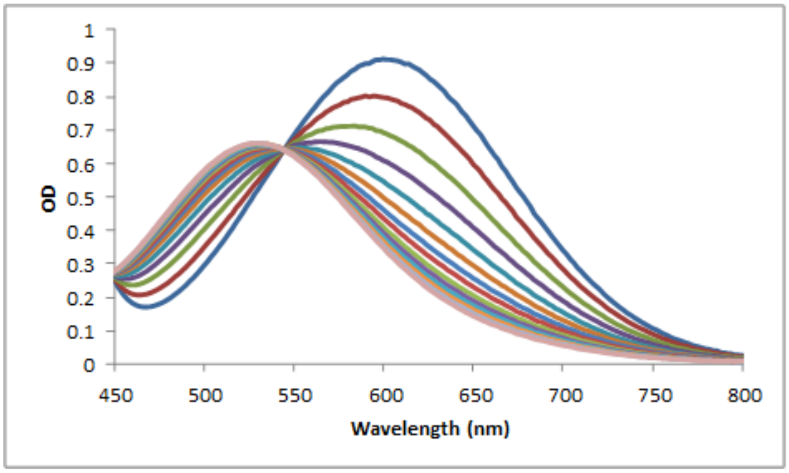

Graph S77. UV/vis spectra of titration of guanidinium.BPh_4 with Reichardt's dye in acetonitrile with 0.5% H_2O added.

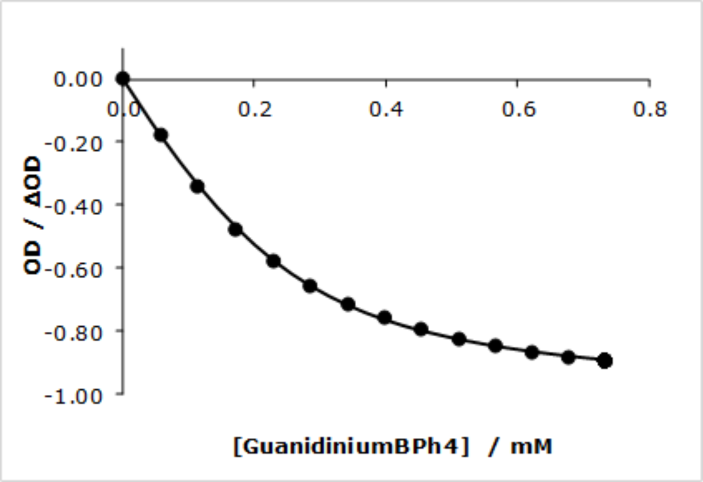

Graph S78. Binding isotherms for titration using 1:1 fitting program for titration of phenol against Reichardt's dye in acetonitrile with 0.5% H_2O added. [Reichardt's dye] = 0.24 mM and [guanidinium.BPh_4] = 41 mM, ΔOD = -1.011.

K_a = 12600 ± 4200 M^{-1} 88% bound

c) Titration of Reichardt's dye with $NaN(SO_2CF_3)_2$ in acetonitrile with 0.5% H_2O added

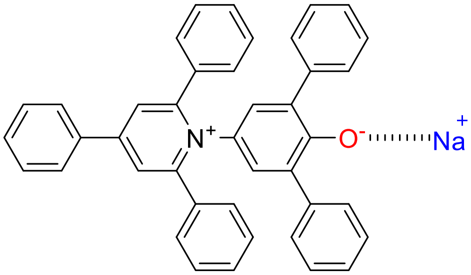

Host: Reichardt's dye = 0.24 mM
Guest: $NaN(SO_2CF_3)_2$ = 68 mM

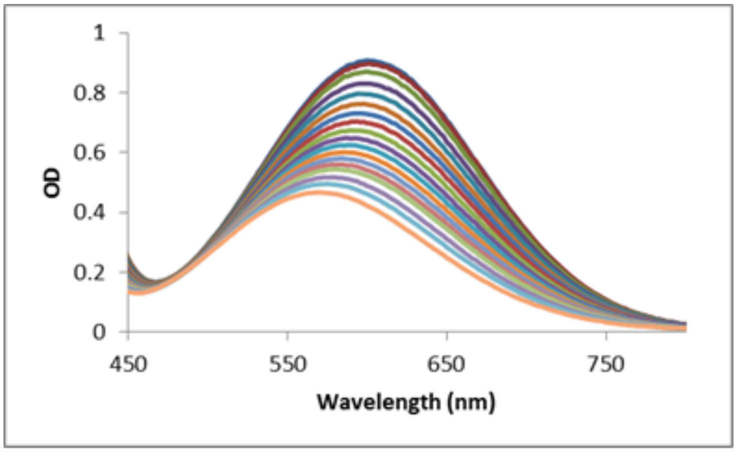

Graph S79. UV/vis spectra of titration of $NaN(SO_2CF_3)_2$ with Reichardt's dye in acetonitrile with 0.5% H_2O added.

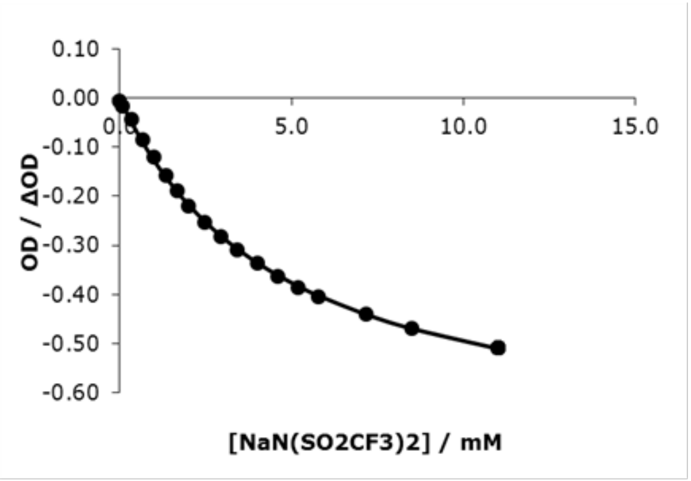

Graph S80. Binding isotherms for titration using 1:1 fitting program for titration of phenol against Reichardt's dye in acetonitrile with 0.5% H_2O added. [Reichardt's dye] = 0.24 mM and [$NaN(SO_2CF_3)_2$] = 68 mM, ΔOD = -0.721.

K_a = 250 ± 60 M^{-1} 71% bound

7. Titration Curves for Reichardt's dye (1) in Acetonitrile with 1.0 % H_2O added

a) <u>Titration of Reichardt's dye with phenol in acetonitrile + 1.0% H_2O added</u>

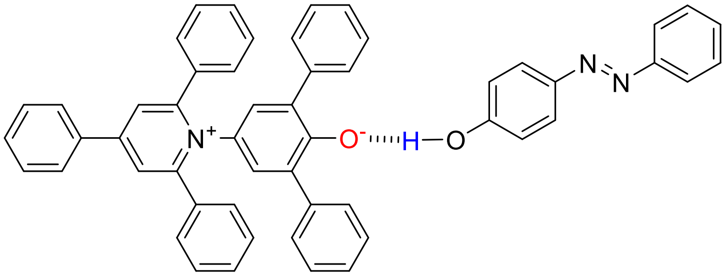

Host: Reichardt's dye = 0.26 mM
Guest: 4-phenylazophenol = 22 mM

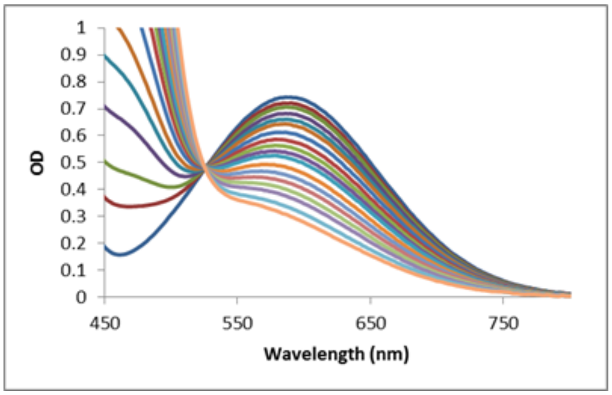

Graph S81. UV/vis spectra of titration of Reichardt's dye with 4-phenylazophenol in acetonitrile with 1% H_2O added.

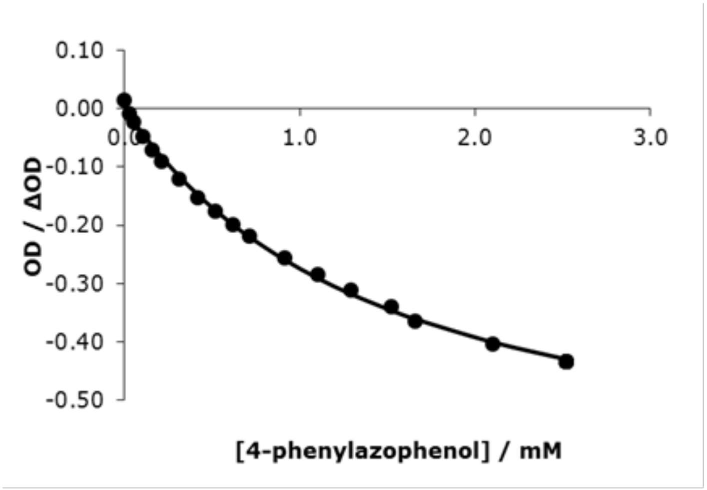

Graph S82. Binding isotherms for titration using 1:1 and accounting for a non-specific secondary interaction fitting program for titration of 4-phenylazophenol against Reichardt's dye in acetonitrile with 1% H_2O added. [Reichardt's dye] = 0.26 mM and [4-phenylazophenol] = 22 mM, $\Delta OD = -0.659$.

K_a = 800 ± 20 M^{-1} **65% bound**

b) Titration of Reichardt's dye with guanidinium.BPh_4 in acetonitrile with 1% H_2O added

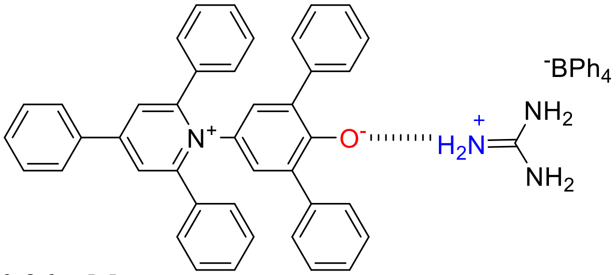

Host: Reichardt's dye = 0.26 mM
Guest: guanidinium.BPh_4 = 27 mM

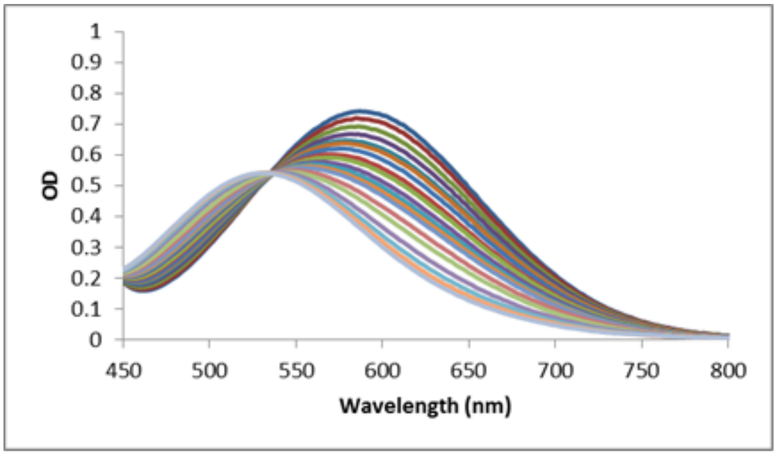

Graph S83. UV/vis spectra of titration of guanidinium.BPh_4 with Reichardt's dye in acetonitrile with 1% H_2O added.

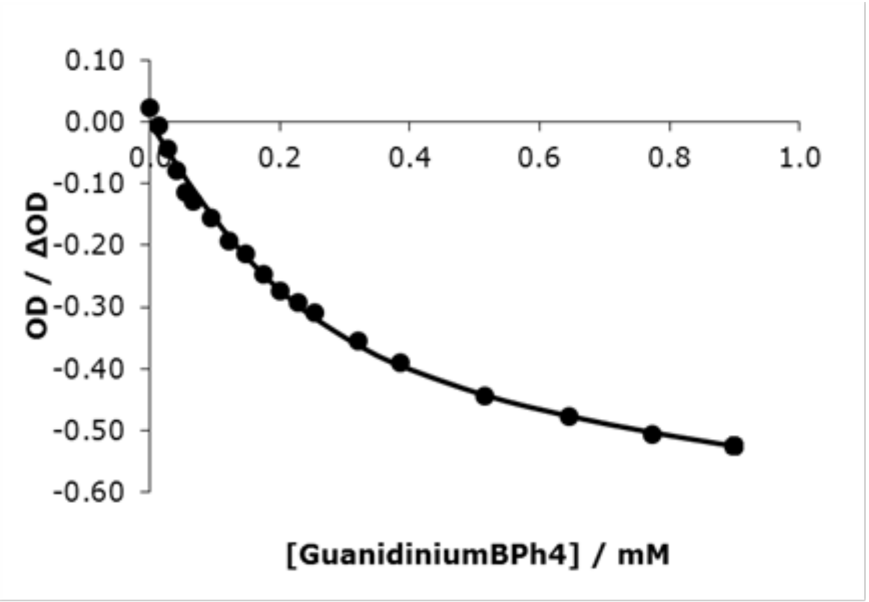

Graph S84. Binding isotherms for titration using 1:1 fitting program for titration of phenol against Reichardt's dye in acetonitrile with 1% H_2O added. [Reichardt's dye] = 0.26 mM and [guanidinium.BPh_4] = 27 mM, ΔOD = -0.519.

K_a = 11000 ± 2000 M^{-1} 89% bound

8. Titration Curves for Reichardt's dye (1) in Acetone with 0.1 % H_2O added

a) Titration of Reichardt's dye with phenol in acetone + 0.1% H_2O added

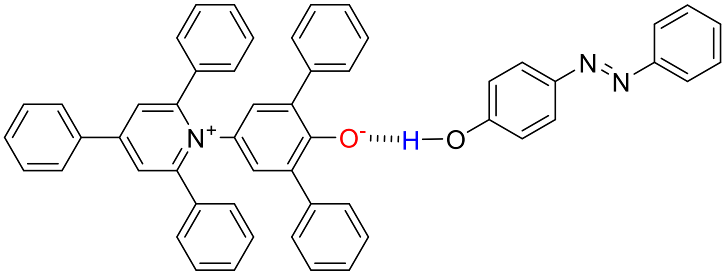

Host: Reichardt's dye = 0.16 mM

Guest: 4-phenylazophenol = 22 mM

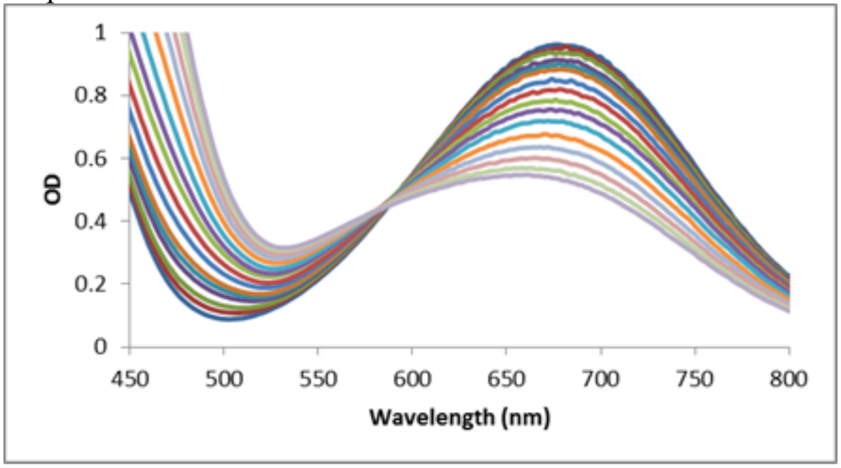

Graph S85. UV/vis spectra of titration of Reichardt's dye with 4-phenylazophenol in acetone with 0.1% H_2O added.

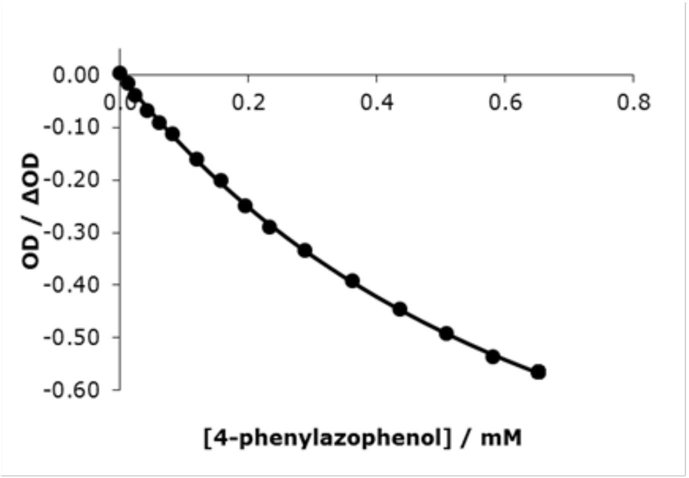

Graph S86. Binding isotherms for titration using 1:1 and accounting for a non-specific secondary interaction fitting program for titration of 4-phenylazophenol against Reichardt's in acetone with 0.1% H_2O added. [Reichardt's dye] = 0.16 mM and [4-phenylazophenol] = 22 mM, ΔOD = -0.946.

K_a = **1800 ± 280 M^{-1}** **67% bound**

b) <u>Titration of Reichardt's dye with $KBPh_4$ in acetone + 0.1 % H_2O</u>

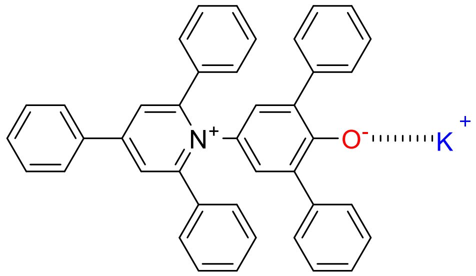

Host: Reichardt's dye = 0.15 mM
Guest: $KBPh_4$ = 34 mM

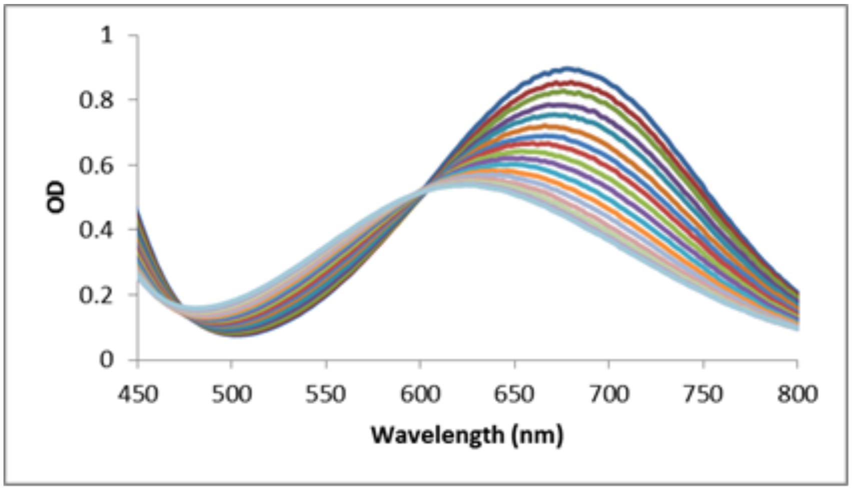

Graph S87. UV/vis spectra of titration of $KBPh_4$ with Reichardt's dye in acetone + 0.1% H_2O.

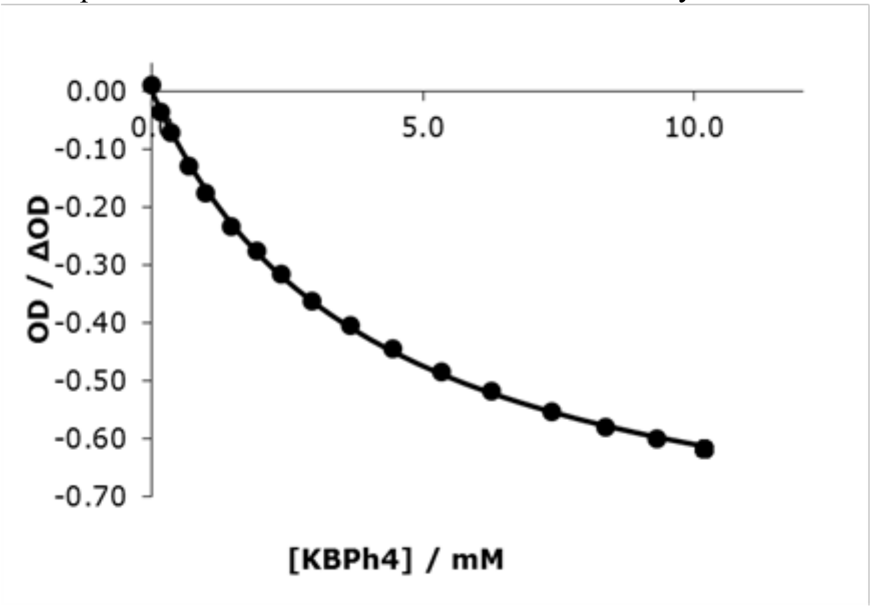

Graph S88. Binding isotherms for titration using 1:1 fitting program for titration of $KBPh_4$ against Reichardt's dye in acetone + 0.1% H_2O. [Reichardt's dye] = 0.15 mM and [$KBPh_4$] = 34 mM, ΔOD = -0.847.

K_a = 260 ± 21 M^{-1} 72% bound

c) Titration of Reichardt's dye with $NaN(SO_2CF_3)_2$ in acetone with 0.1% H_2O added

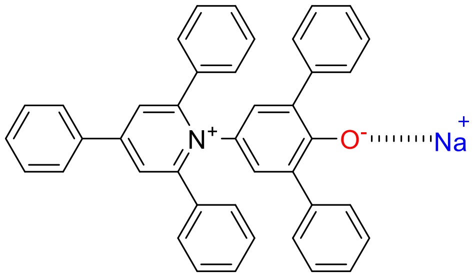

Host: Reichardt's dye = 0.16 mM
Guest: $NaN(SO_2CF_3)_2$ = 81 mM

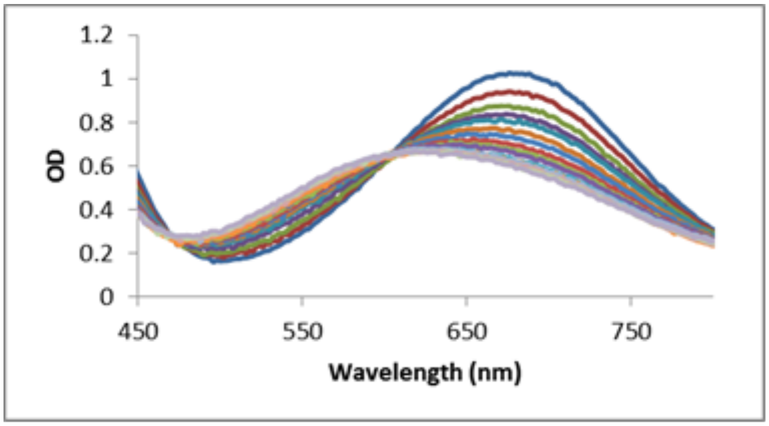

Graph S89. UV/vis spectra of titration of $NaN(SO_2CF_3)_2$ with Reichardt's dye in acetone with 0.1% H_2O added.

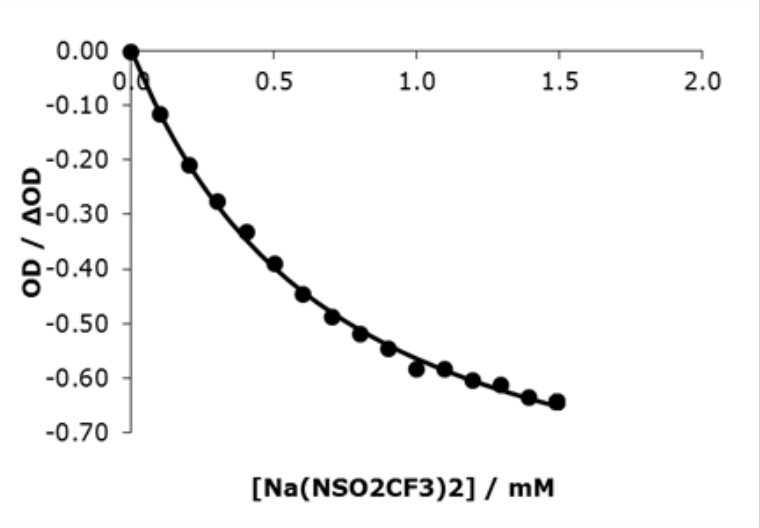

Graph S90. Binding isotherms for titration using 1:1 fitting program for titration of phenol against Reichardt's dye in acetone with 0.1% H_2O added. [Reichardt's dye] = 0.16 mM and [$NaN(SO_2CF_3)_2$] = 81 mM, ΔOD = -0.921.

K_a = 1500 ± 500 M^{-1} 71% bound

9. Titration Curves for Reichardt's dye (1) in Acetone with 0.25 % H_2O added

a) Titration of Reichardt's dye with phenol in acetone + 0.25% H_2O added

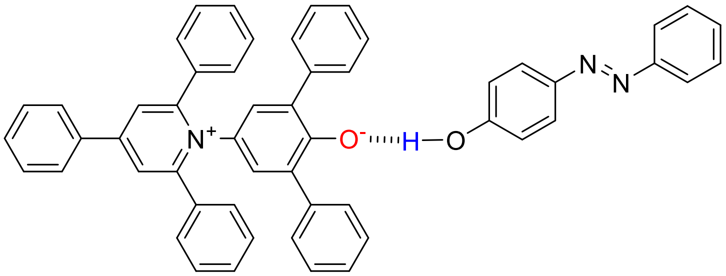

Host: Reichardt's dye = 0.15 mM

Guest: 4-phenylazophenol = 17 mM

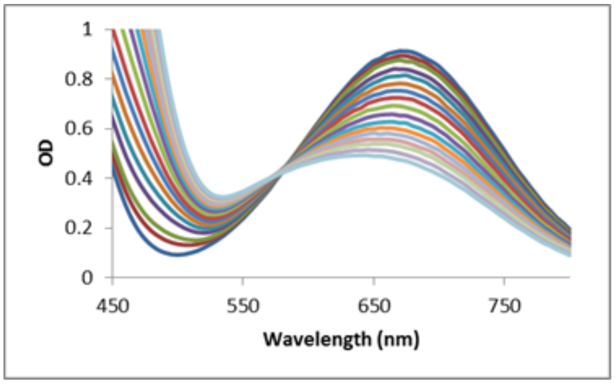

Graph S91. UV/vis spectra of titration of Reichardt's dye with 4-phenylazophenol in acetone with 0.25% H_2O added.

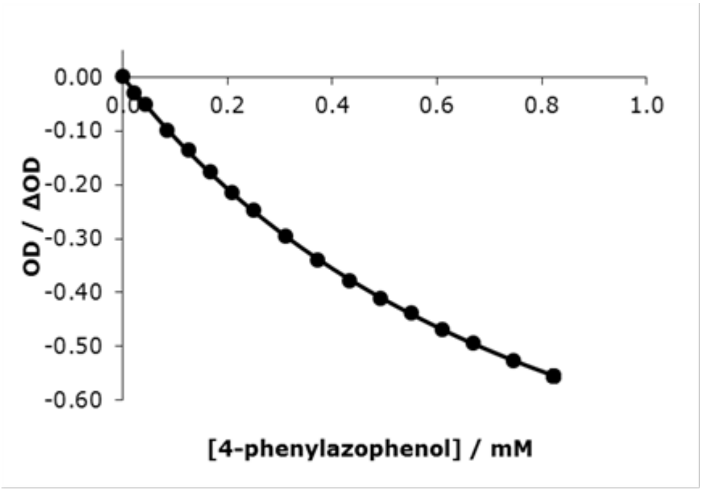

Graph S92. Binding isotherms for titration using 1:1 and accounting for a non-specific secondary interaction fitting program for titration of 4-phenylazophenol against Reichardt's dye in acetone with 0.25% H_2O added. [Reichardt's dye] = 0.15 mM and [4-phenylazophenol] = 17 mM, ΔOD = -1.100.

K_a = 1300 ± 71 M^{-1} **50% bound**

b) <u>Titration of Reichardt's dye with $NaN(SO_2CF_3)_2$ in acetone with 0.25% H_2O added</u>

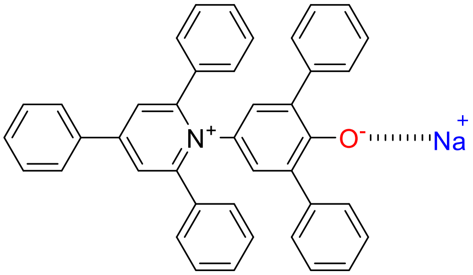

Host: Reichardt's dye = 0.15 mM
Guest: $NaN(SO_2CF_3)_2$ = 89 mM

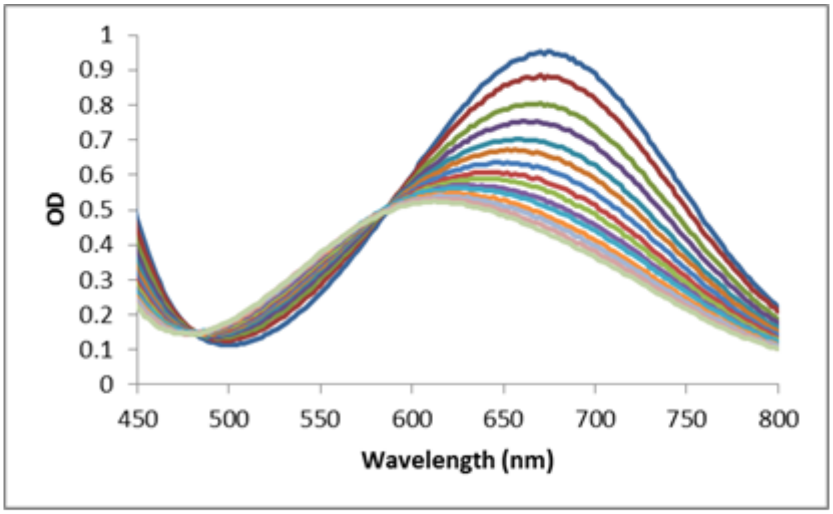

Graph S93. UV/vis spectra of titration of $NaN(SO_2CF_3)_2$ with Reichardt's dye in acetone with 0.25% H_2O added.

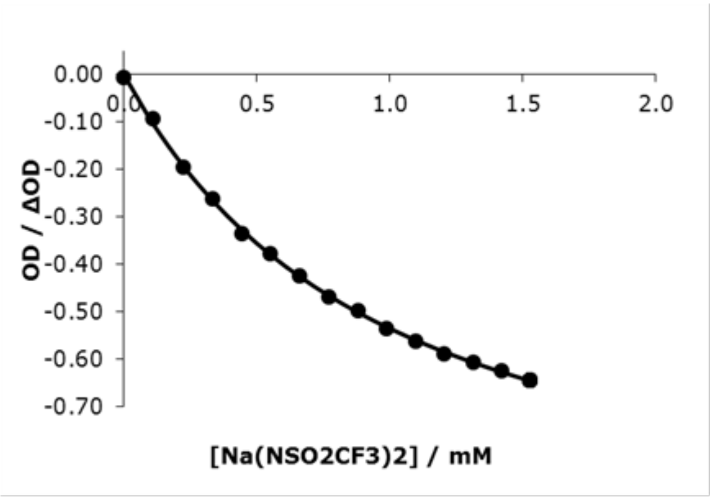

Graph S94. Binding isotherms for titration using 1:1 fitting program for titration of phenol against Reichardt's dye in acetone with 0.5% H_2O added. [Reichardt's dye] = 0.15 mM and [$NaN(SO_2CF_3)_2$] = 89 mM, ΔOD = -1.023.

K_a = 1100 ± 200 M^{-1} 63% bound

c) <u>Titration of Reichardt's dye with $KBPh_4$ in acetone with 0.25% H_2O</u>

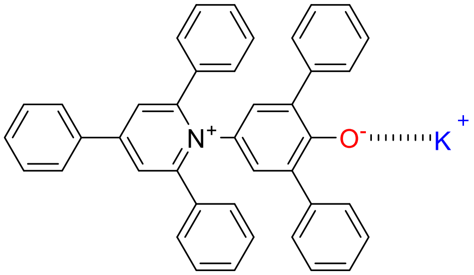

Host: Reichardt's dye = 0.15 mM
Guest: $KBPh_4$ = 45 mM

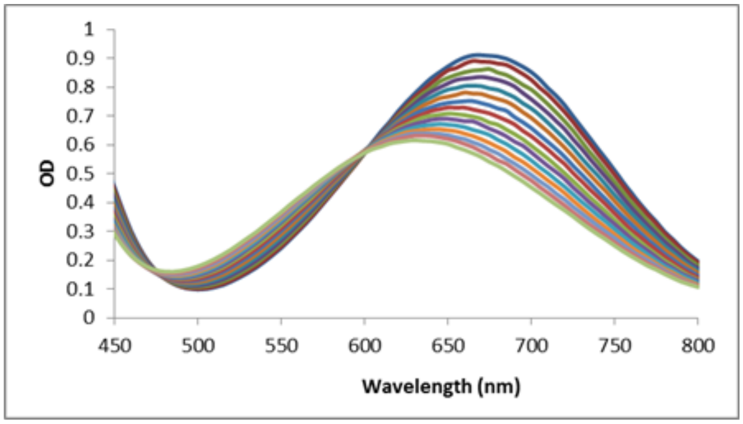

Graph S95. UV/vis spectra of titration of $KBPh_4$ with Reichardt's dye in acetone + 0.25% H_2O.

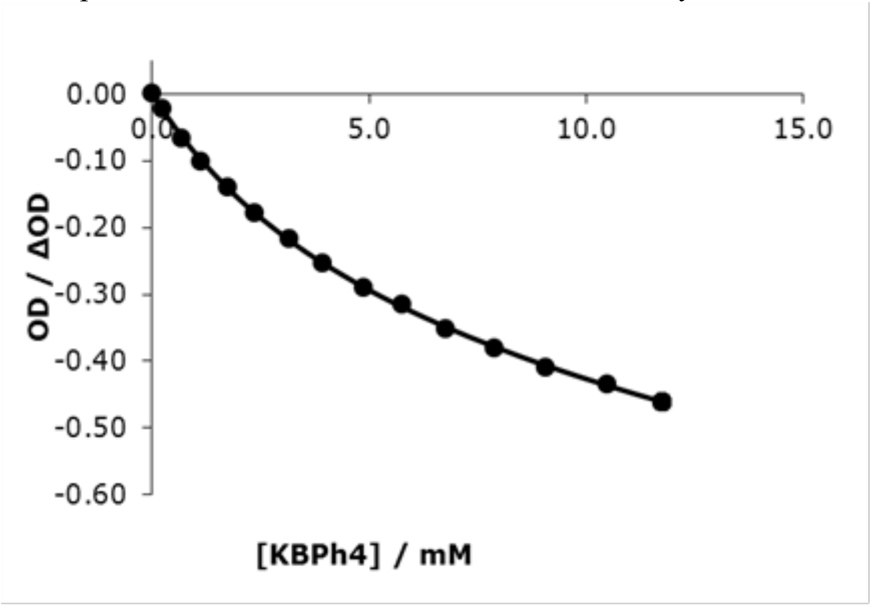

Graph S96. Binding isotherms for titration using 1:1 fitting program and accounting for a non-specific interaction for titration of $KBPh_4$ against Reichardt's dye in acetone + 0.25% H_2O. [Reichardt's dye] = 0.15 mM and [$KBPh_4$] = 45 mM, $\Delta OD = -0.569$.

K_a = 190 ± 15 M^{-1} 68% bound

10. Titration Curves for Reichardt's dye (1) in Acetone with 0.5 % H_2O added

a) Titration of Reichardt's dye with phenol in acetone + 0.5% H_2O added

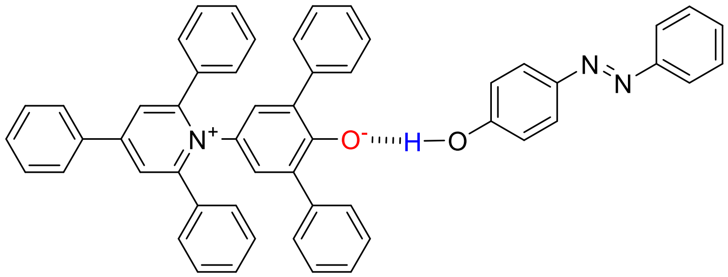

Host: Reichardt's dye = 0.16 mM

Guest: 4-phenylazophenol = 19 mM

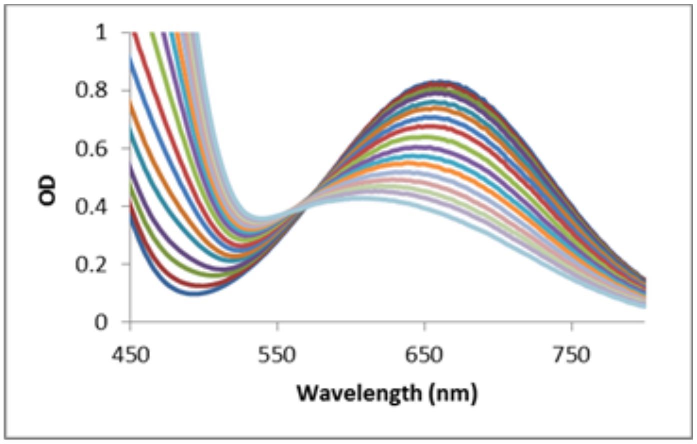

Graph S97. UV/vis spectra of titration of Reichardt's dye with 4-phenylazophenol in acetone with 0.5% H_2O added.

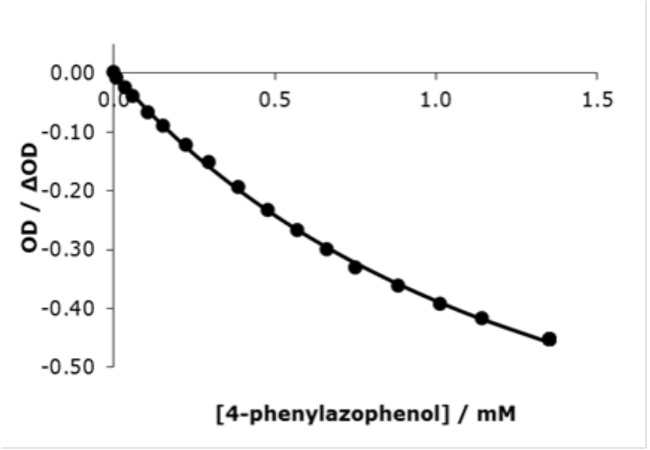

Graph S98. Binding isotherms for titration using 1:1 and accounting for a non-specific secondary interaction fitting program for titration of 4-phenylazophenol against Reichardt's dye in acetone with 0.5% H_2O added. [Reichardt's dye] = 0.16 mM and [4-phenylazophenol] = 19 mM, ΔOD = -0.922.

K_a = 860 ± 240 M^{-1} 50% bound

b) Titration of Reichardt's dye with $NaN(SO_2CF_3)_2$ in acetone with 0.5% H_2O added

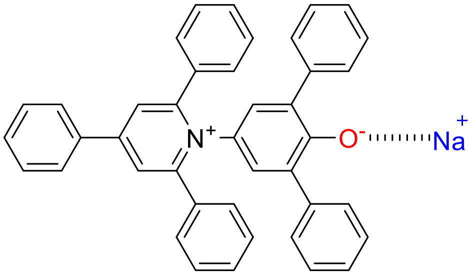

Host: Reichardt's dye = 0.15 mM
Guest: $NaN(SO_2CF_3)_2$ = 112 mM

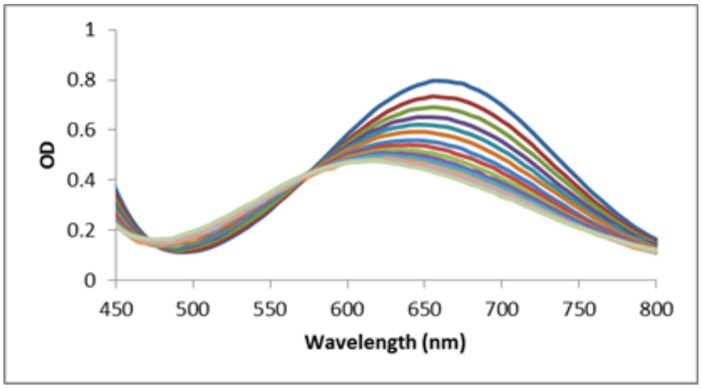

Graph S99. UV/vis spectra of titration of $NaN(SO_2CF_3)_2$ with Reichardt's dye in acetone with 0.5% H_2O added.

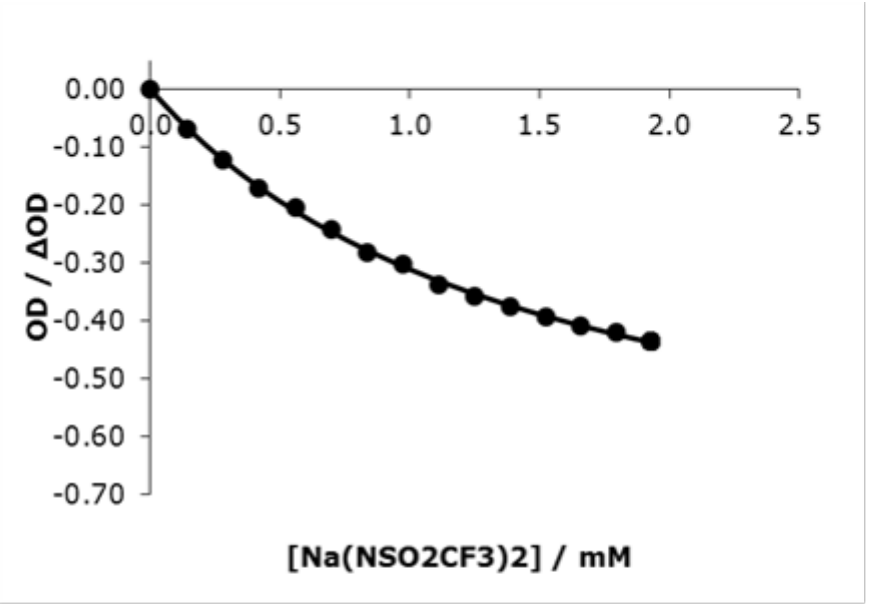

Graph S100. Binding isotherms for titration using 1:1 fitting program for titration of phenol against Reichardt's dye in acetone with 0.5% H_2O added. [Reichardt's dye] = 0.15 mM and [$NaN(SO_2CF_3)_2$] = 112 mM, ΔOD = -0.754.

K_a = 840 ± 280 M^{-1} 58% bound

c) Titration of Reichardt's dye with $KBPh_4$ in acetone with 0.5% H_2O

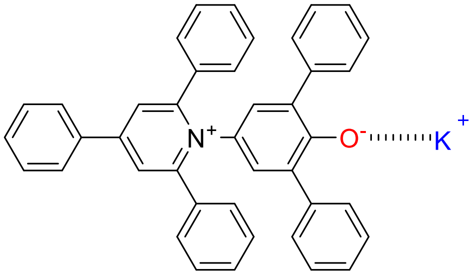

Host: Reichardt's dye = 0.15 mM
Guest: $KBPh_4$ = 20 mM

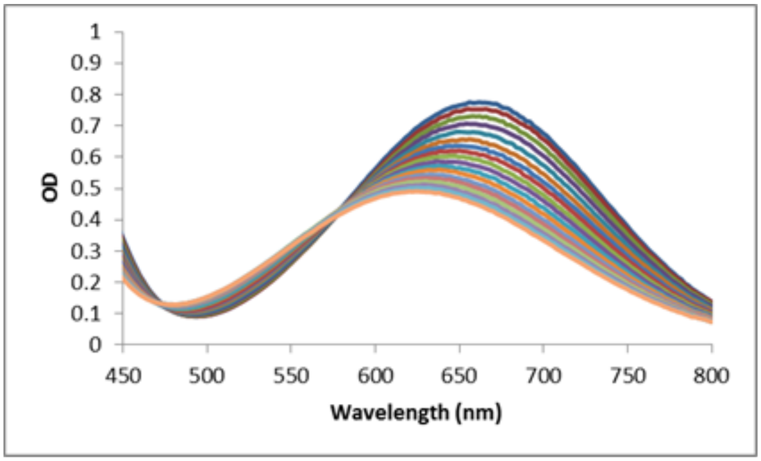

Graph S101. UV/vis spectra of titration of $KBPh_4$ with Reichardt's dye in acetone + 0.5% H_2O.

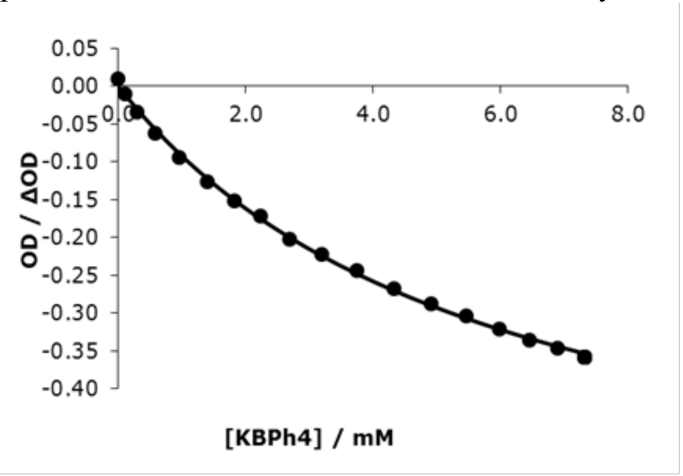

Graph S102. Binding isotherms for titration using 1:1 fitting program for titration of $KBPh_4$ against Reichardt's dye in acetone + 0.5% H_2O. [Reichardt's dye] = 0.15 mM and [$KBPh_4$] = 20 mM, ΔOD = -0.632.

K_a = **170 ± 18 M^{-1}** **56% bound**

11. Titration Curves for Reichardt's dye (1) in Acetone with 1 % H_2O added

a) <u>Titration of Reichardt's dye with phenol in acetone + 1% H_2O added</u>

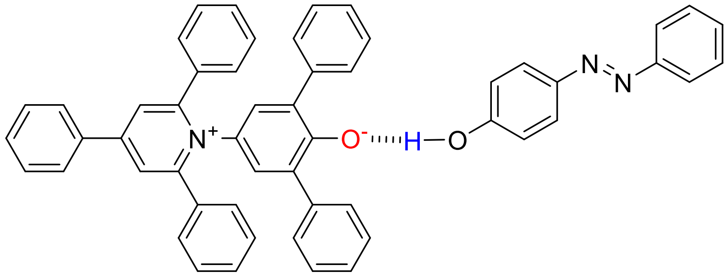

Host: Reichardt's dye = 0.21 mM
Guest: 4-phenylazophenol = 19 mM

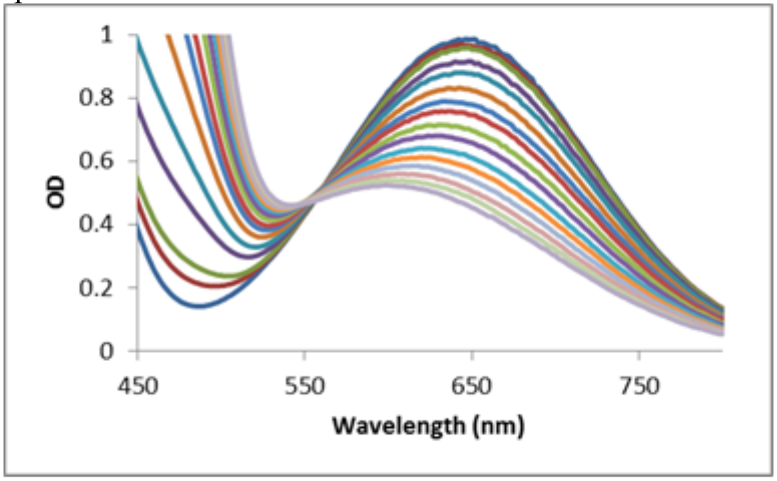

Graph S103. UV/vis spectra of titration of Reichardt's dye with 4-phenylazophenol in acetone with 1% H_2O added.

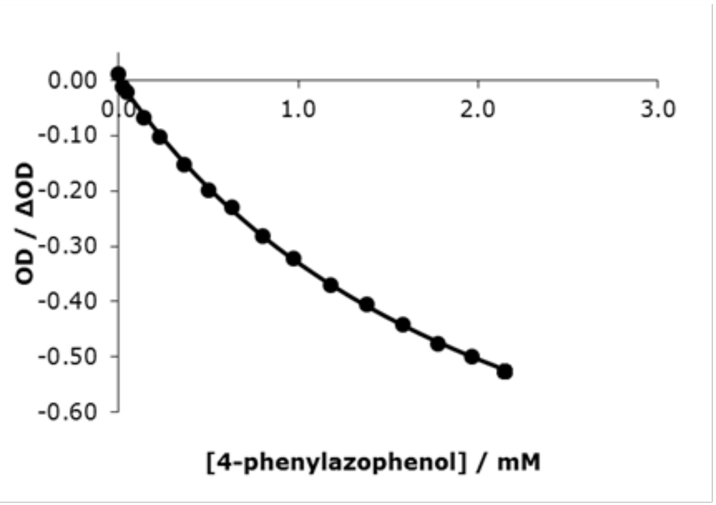

Graph S104. Binding isotherms for titration using 1:1 fitting program for titration of 4-phenylazophenol against Reichardt's dye in acetone with % H_2O added. [Reichardt's dye] = 0.21 mM and [4-phenylazophenol] = 19 mM, ΔOD = -1.028.

K_a = 600 ± 200 M^{-1} **51% bound**

b) Titration of Reichardt's dye with $NaN(SO_2CF_3)_2$ in acetone with 1% H_2O added

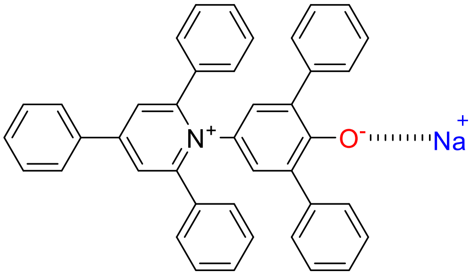

Host: Reichardt's dye = 0.25 mM
Guest: $NaN(SO_2CF_3)_2$ = 120 mM

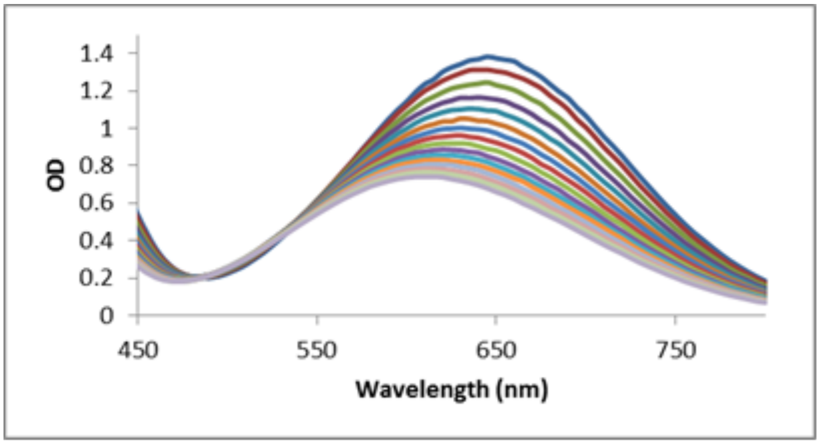

Graph S105. UV/vis spectra of titration of $NaN(SO_2CF_3)_2$ with Reichardt's dye in acetone with 1% H_2O added.

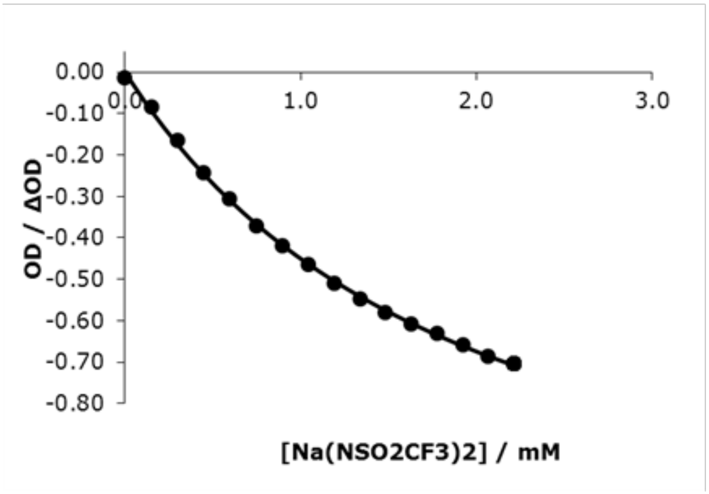

Graph S106. Binding isotherms for titration using 1:1 fitting program for titration of phenol against Reichardt's dye in acetone with 1% H_2O added. [Reichardt's dye] = 0.25 mM and $[NaN(SO_2CF_3)_2]$ = 120 mM, ΔOD = -1.287.

K_a = 550 ± 120 M^{-1} **55% bound**

c) <u>Titration of Reichardt's dye with $KBPh_4$ in acetone with 1% H_2O</u>

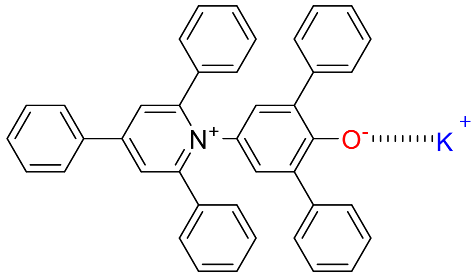

Host: Reichardt's dye = 0.25 mM
Guest: $KBPh_4$ = 25 mM

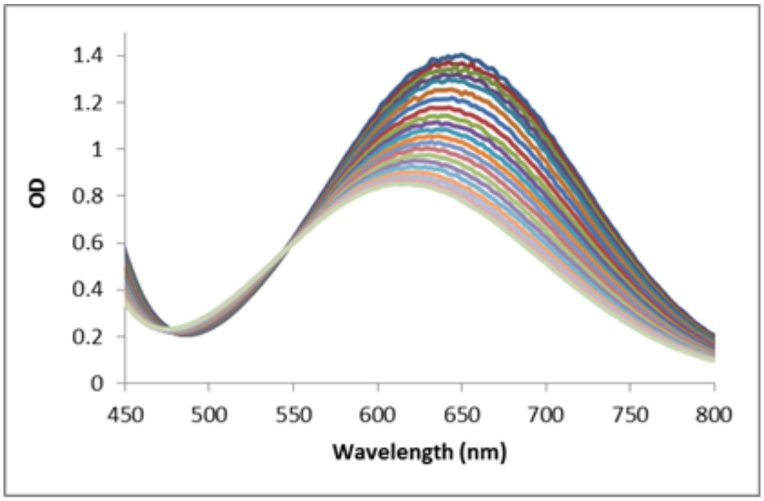

Graph S107. UV/vis spectra of titration of $KBPh_4$ with Reichardt's dye in acetone + 1% H_2O.

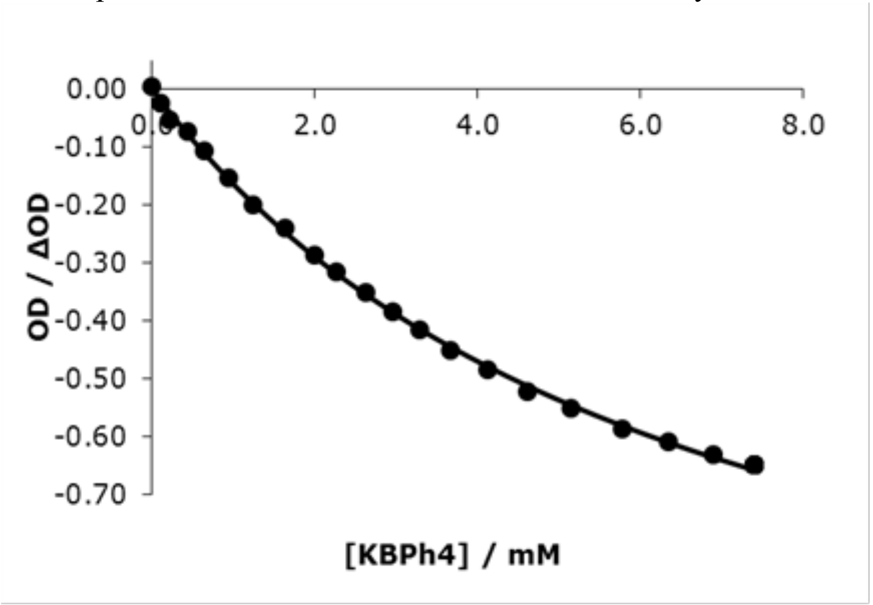

Graph S108. Binding isotherms for titration using 1:1 fitting program for titration of $KBPh_4$ against Reichardt's dye in acetone + 1% H_2O. [Reichardt's dye] = 0.25 mM and [$KBPh_4$] = 22 mM, ΔOD = -1.226.

K_a = 132 ± 70 M^{-1} **54% bound**

12. Titration Curves for Reichardt's dye (1) in Chloroform

a) Titration of Reichardt's dye with 4-phenylazophenol in chloroform

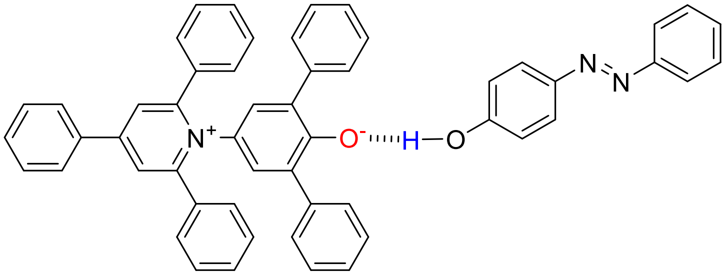

Host: Reichardt's dye = 0.056 mM

Guest: 4-phenylazophenol = 13 mM

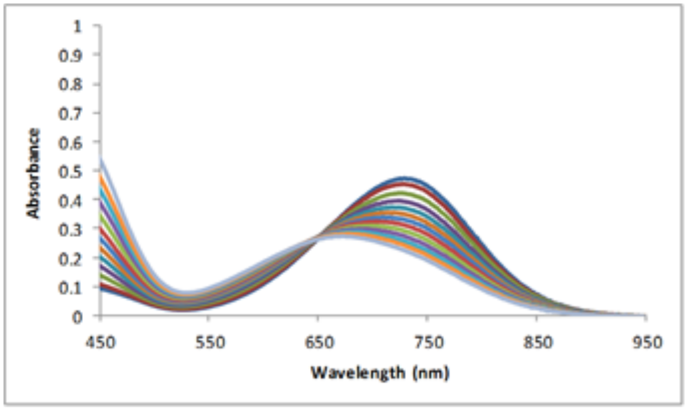

Graph S109. UV/vis spectra of titration of 4-phenylazophenol with Reichardt's dye in chloroform.

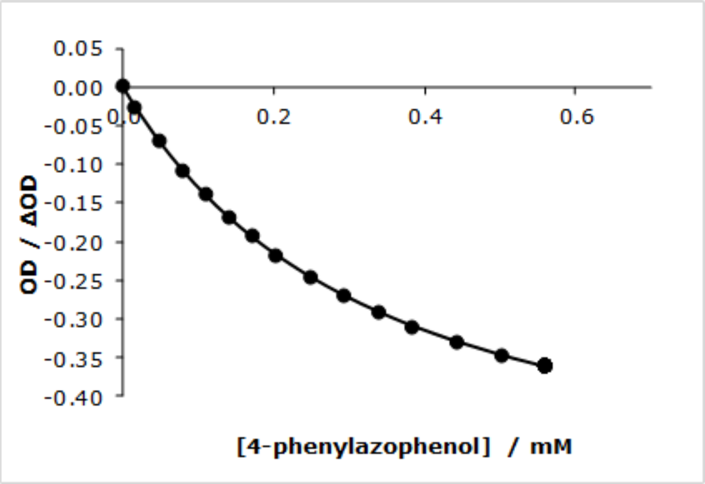

Graph S110. Binding isotherms for titration using 1:1 fitting program for titration of 4-phenylazophenol against Reichardt's dye in acetonitrile. [Reichardt's dye] = 0.056 mM and [4-phenylazophenol] = 13 mM, ΔOD = -0.560.

K_a = 3970 ± 707 M^{-1} 65% bound

b) <u>Titration of Reichardt's dye with phenol in chloroform</u>

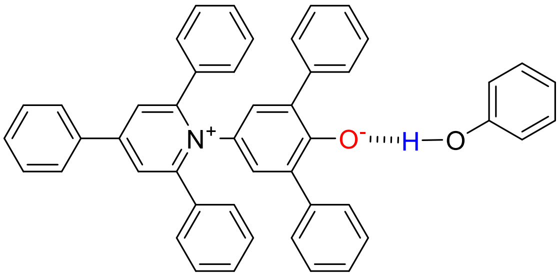

Host: Reichardt's dye = 0.11 mM
Guest: phenol = 110 mM

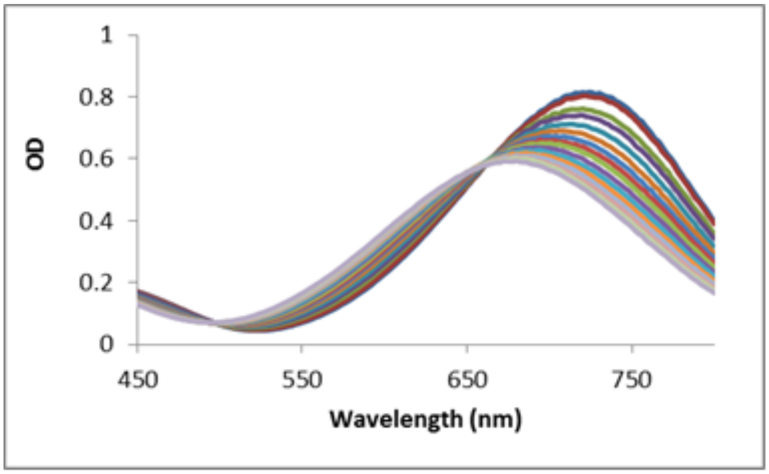

Graph S111. UV/vis spectra of titration of phenol with Reichardt's dye in chloroform.

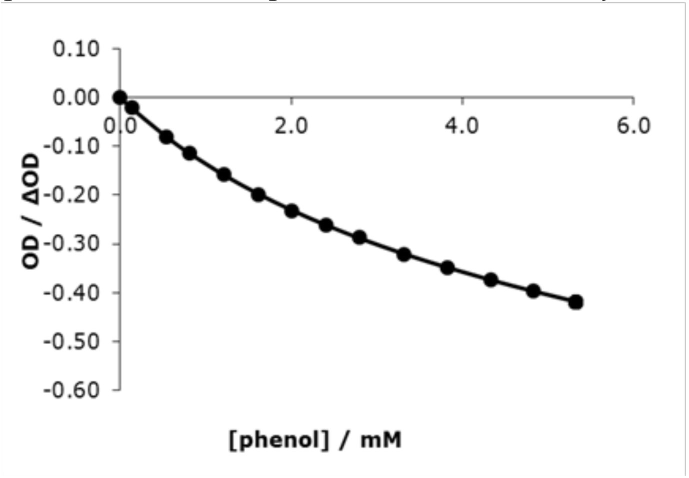

Graph S112. Binding isotherms for titration using 1:1 fitting program and accounting for a nonspecific interaction for titration of phenol against Reichardt's dye in chloroform. [Reichardt's dye] = 0.11 mM and [phenol] = 110 mM, ΔOD = -0.628.

K_a = 260 ± 55 M^{-1} 59% bound

c) <u>Titration of Reichardt's dye with 5-nitroindole in chloroform</u>

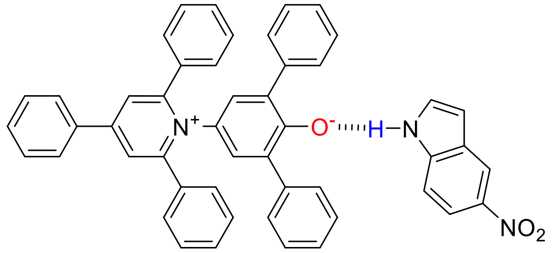

Host: Reichardt's dye = 0.11 mM
Guest: 5-nitroindole = 30 mM

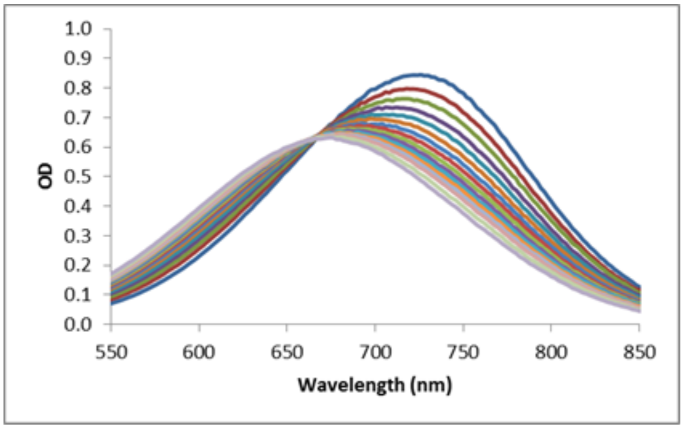

Graph S113. UV/vis spectra of titration of Reichardt's dye with 5-nitroindole in chloroform.

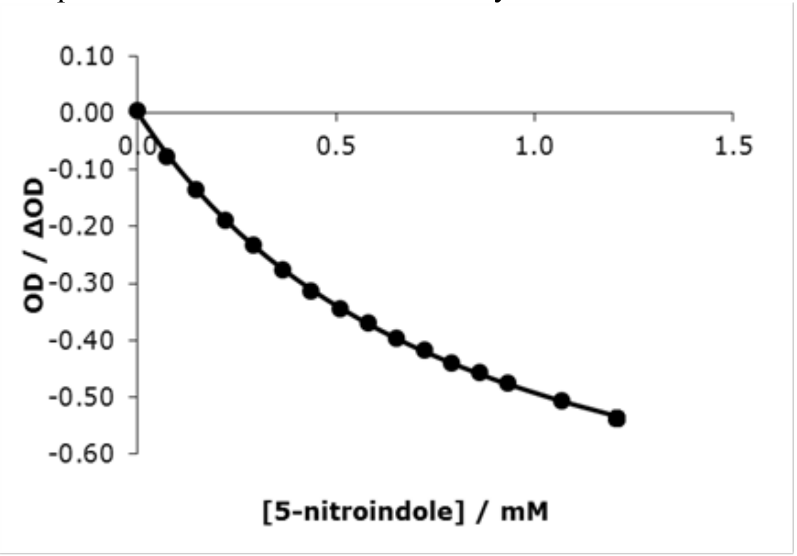

Graph S114. Binding isotherms for titration using 1:1 fitting program for titration of 5-nitroindole against Reichardt's dye in chloroform. [Reichardt's dye] = 0.11 mM and [5-nitroindole] = 30 mM, ΔOD = -0.868.

K_a = 1200 ± 500 M^{-1} 61% bound

13. Titration Curves for Phosphine oxide (2) in Acetonitrile

a) Titration of 4-nitro,3-trifluoromethylphenol with phosphine oxide in acetonitrile

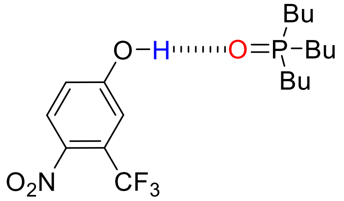

Host: 4-nitro,3-trifluoromethylphenol = 0.12 mM
Guest: phosphine oxide = 184 mM

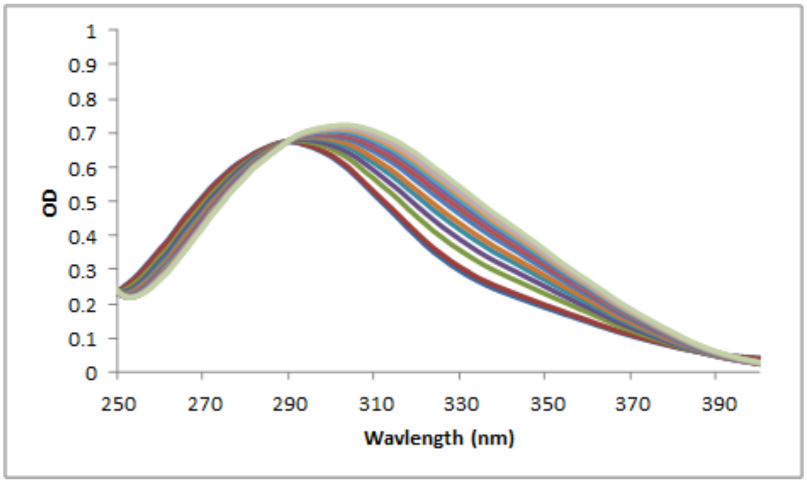

Graph S115. UV/vis spectra of titration of phosphine oxide with 4-nitro,3-trifluoromethylphenol in acetonitrile.

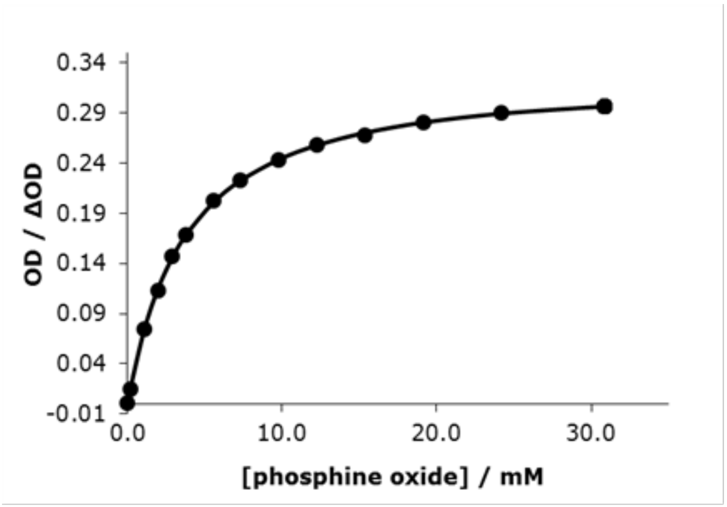

Graph S116. Binding isotherm for titration using 1:1 fitting program and accounting for a non-specific interaction for titration of phosphine oxide against 4-nitro,3-trifluoromethylphenol in acetonitrile. [4-nitro,3-trifluoromethylphenol] = 0.12 mM and [phosphine oxide] = 184 mM, ΔOD = 0.334.

K_a = 300 ± 98 M^{-1} 89% bound

b) Titration of 4-phenylazophenol with phosphine oxide in acetonitrile

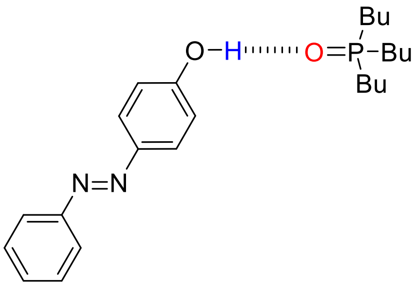

Host: 4-phenylazophenol = 0.029 mM
Guest: phosphine oxide = 114 mM

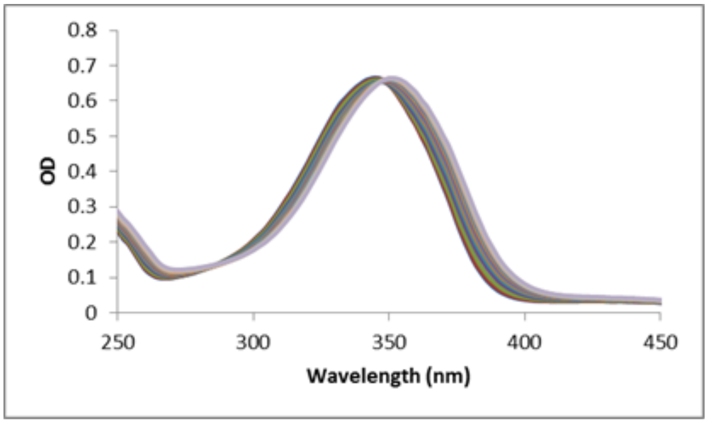

Graph S117. UV/vis spectra of titration of phosphine oxide with 4-phenylazophenol in acetonitrile.

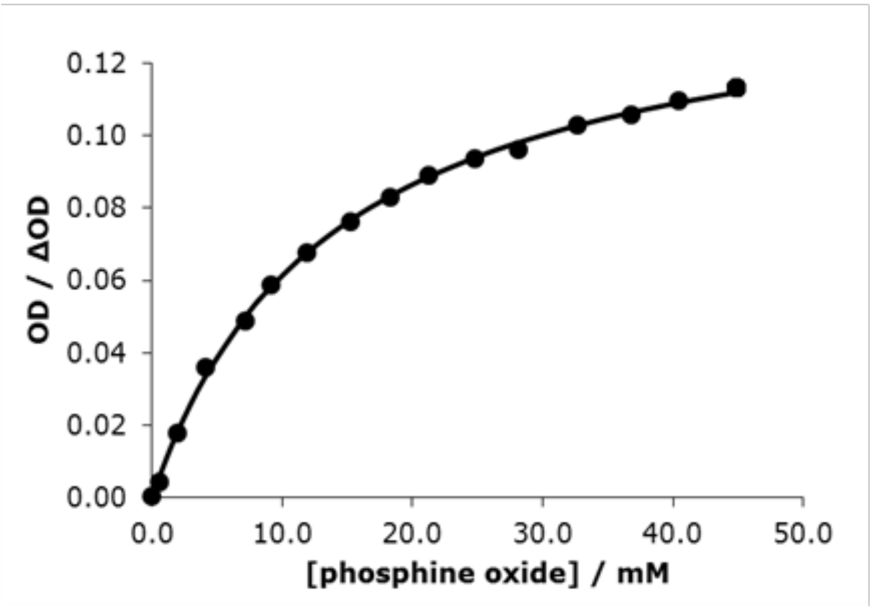

Graph S118. Binding isotherm for titration using 1:1 fitting program and accounting for a non-specific interaction for titration of phosphine oxide against 4-phenylazophenol in acetonitrile. [4-phenylazophenol] = 0.029 mM and [phosphine oxide] = 114 mM, ΔOD = 0.146.

K_a = 69 ± 9 M^{-1} 77% bound

c) <u>Titration of 4-nitrophenol with phosphine oxide in acetonitrile</u>

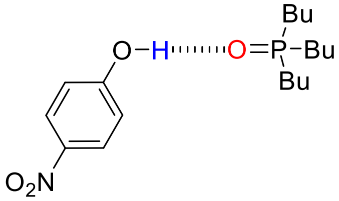

Host: 4-nitrophenol = 0.085 mM
Guest: phosphine oxide = 48 mM

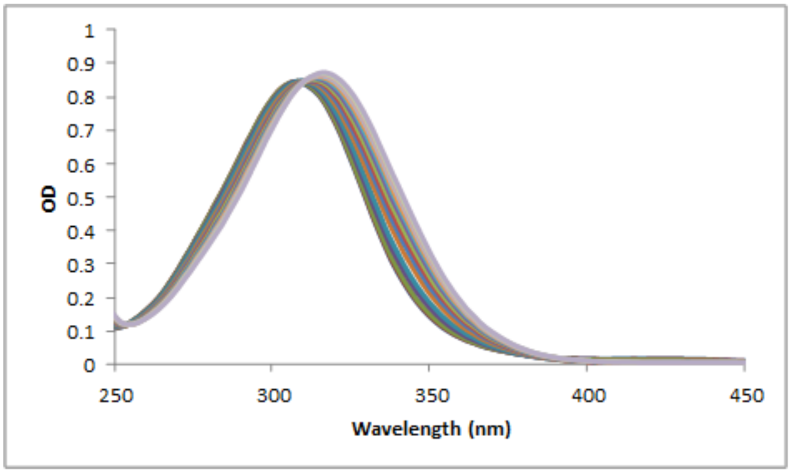

Graph S119. UV/vis spectra of titration of phosphine oxide with 4-nitrophenol in acetonitrile.

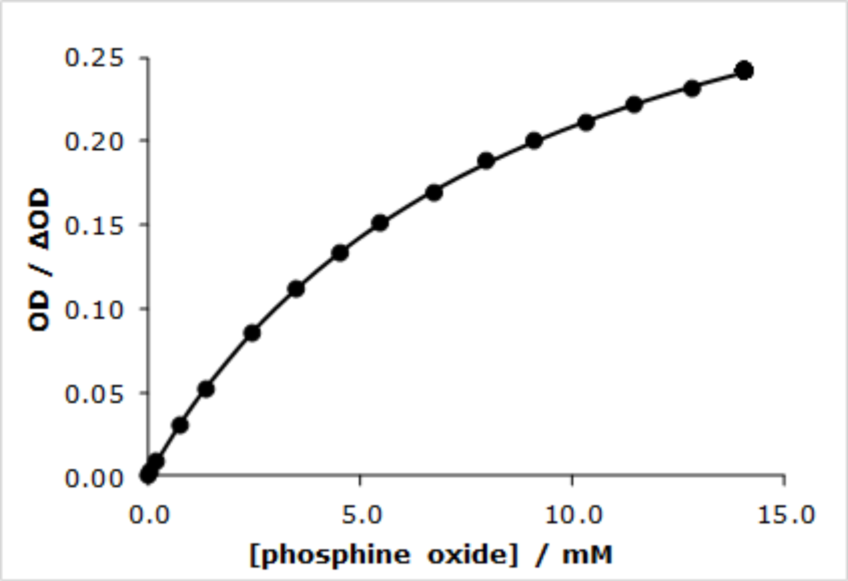

Graph S120. Binding isotherm for titration using 1:1 fitting program and accounting for a non-specific interaction for titration of phosphine oxide against 4-nitrophenol in acetonitrile. [4-nitrophenol] = 0.085 mM and [phosphine oxide] = 48 mM, ΔOD = 0.389.

K_a = 110 ± 36 M^{-1} 62% bound

14. Titration Curves for phosphine oxide (2) in Chloroform

a) Titration of 4-nitro,3-trifluoromethylphenol with phosphine oxide in chloroform

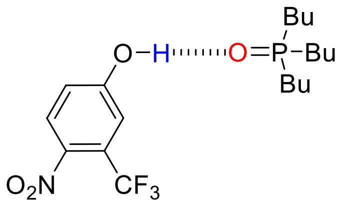

Host: 4-nitro,3-trifluoromethylphenol = 0.12 mM
Guest: phosphine oxide = 47 mM

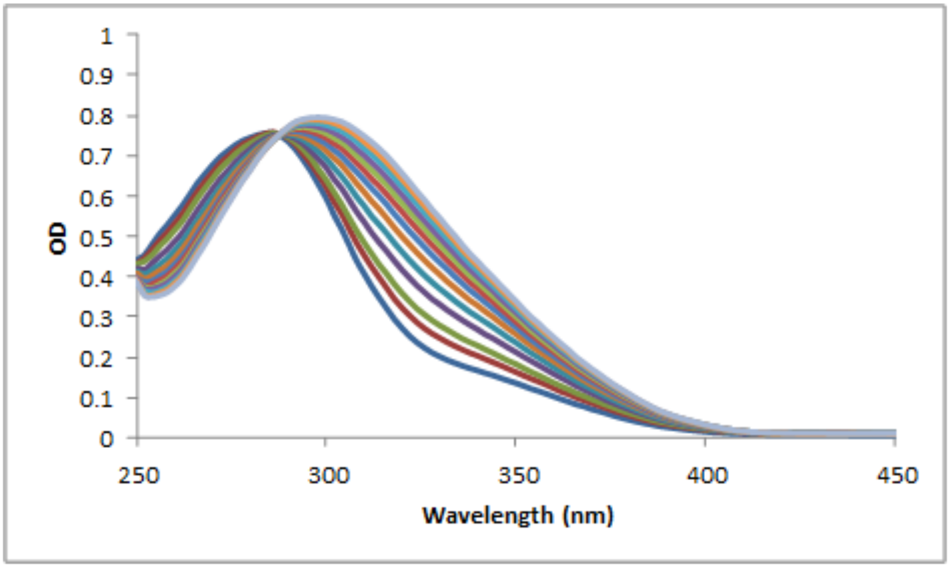

Graph S121. UV/vis spectra of titration of phosphine oxide with 4-nitro,3-trifluoromethylphenol in chloroform.

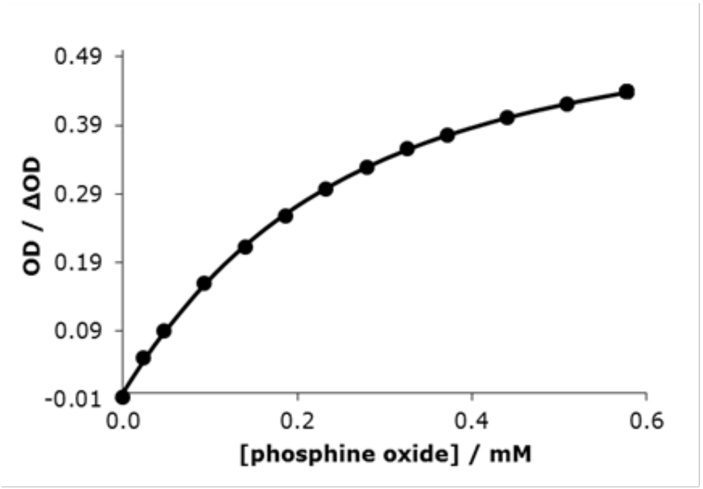

Graph S122. Binding isotherm for titration using 1:1 fitting program and accounting for a non-specific interaction for titration of phosphine oxide against 4-nitro,3-trifluoromethylphenol in chloroform. [4-nitro,3-trifluoromethylphenol] = 0.12 mM and [phosphine oxide] = 47 mM, ΔOD = 0.588.

K_a = 5400 ± 1700 M^{-1} 75% bound

b) Titration of 4-nitrophenol with phosphine oxide in chloroform

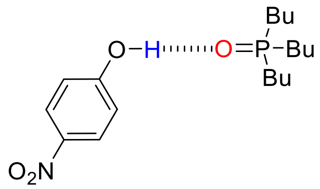

Host: 4-nitrophenol = 0.072 mM
Guest: phosphine oxide = 30 mM

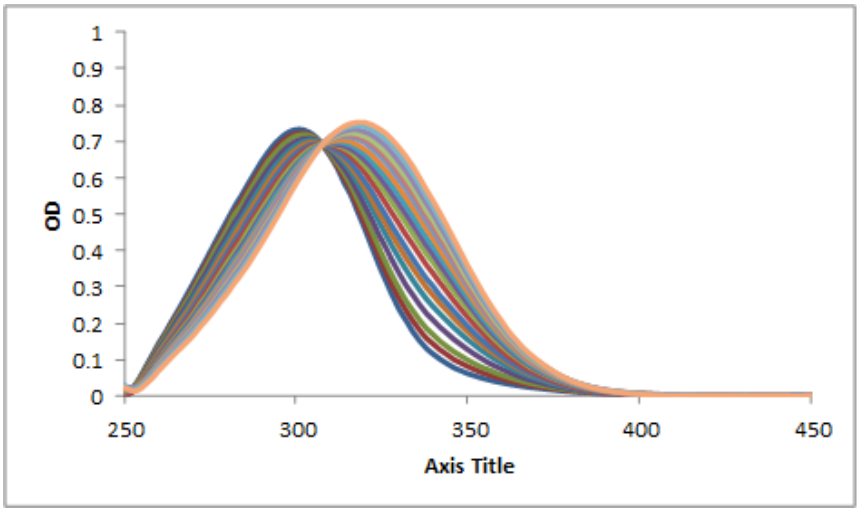

Graph S123. UV/vis spectra of titration of phosphine oxide with 4-nitrophenol in $CHCl_3$.

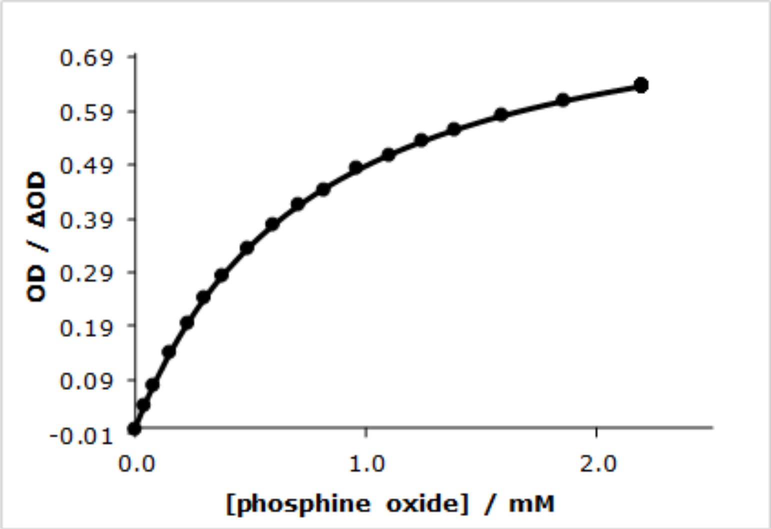

Graph S124. Binding isotherm for titration using 1:1 fitting program and accounting for a non-specific interaction for titration of phosphine oxide against 4-nitrophenol in chloroform. [4-nitrophenol] = 0.072 mM and [phosphine oxide] = 30 mM, ΔOD = 0.847.

K_a = 1400 ± 110 M^{-1} 75% bound

c) Titration of 4-phenylazophenol with phosphine oxide in chloroform

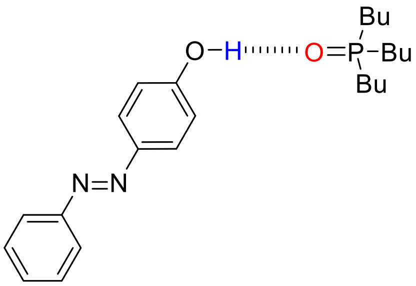

Host: 4-phenylazophenol = 0.049 mM
Guest: phosphine oxide = 68 mM

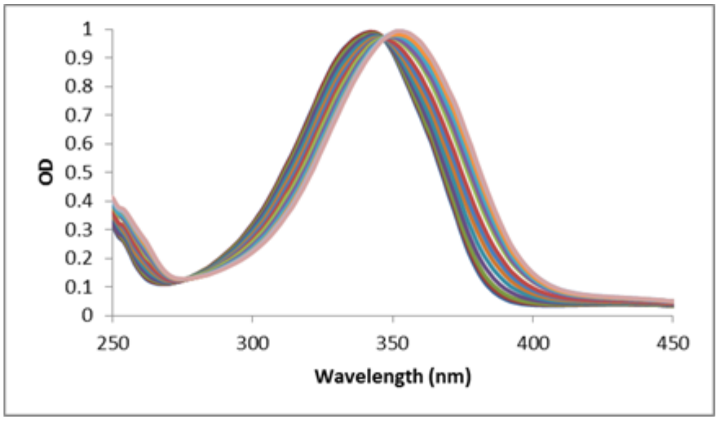

Graph S125. UV/vis spectra of titration of phosphine oxide with 4-phenylazophenol in chloroform.

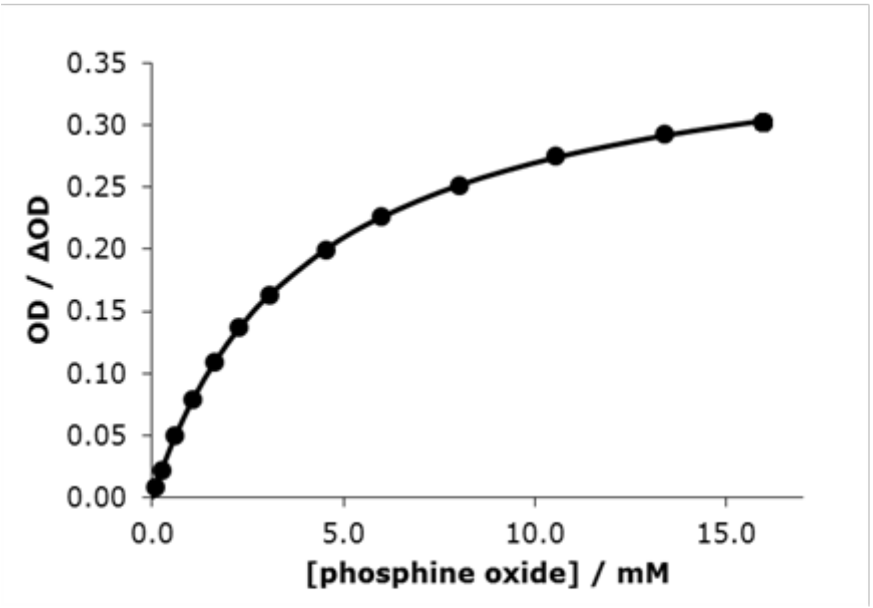

Graph S126. Binding isotherm for titration using 1:1 fitting program and accounting for a non-specific interaction for titration of phosphine oxide against 4-phenylazophenol in chloroform. [4-phenylazophenol] = 0.049 mM and [phosphine oxide] = 68 mM, ΔOD = 0.371.

$\boldsymbol{K_a}$ **= 240 ± 45** $\mathbf{M^{-1}}$ **80% bound**

d) Titration of 5-nitroindole with phosphine oxide in chloroform

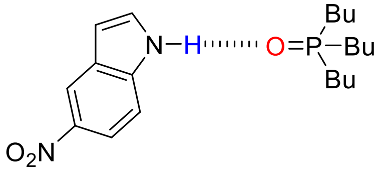

Host: 5-nitroindole = 0.09 mM
Guest: phosphine oxide = 116 mM

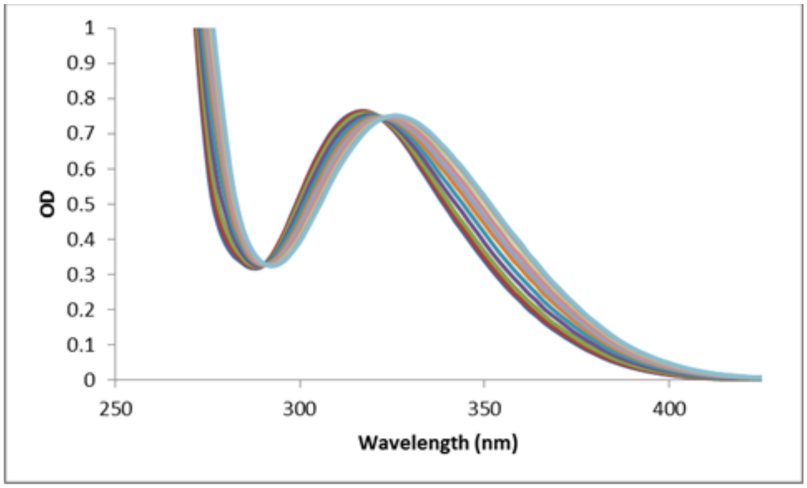

Graph S127. UV/vis spectra of titration of phosphine oxide with 5-nitroindole in chloroform.

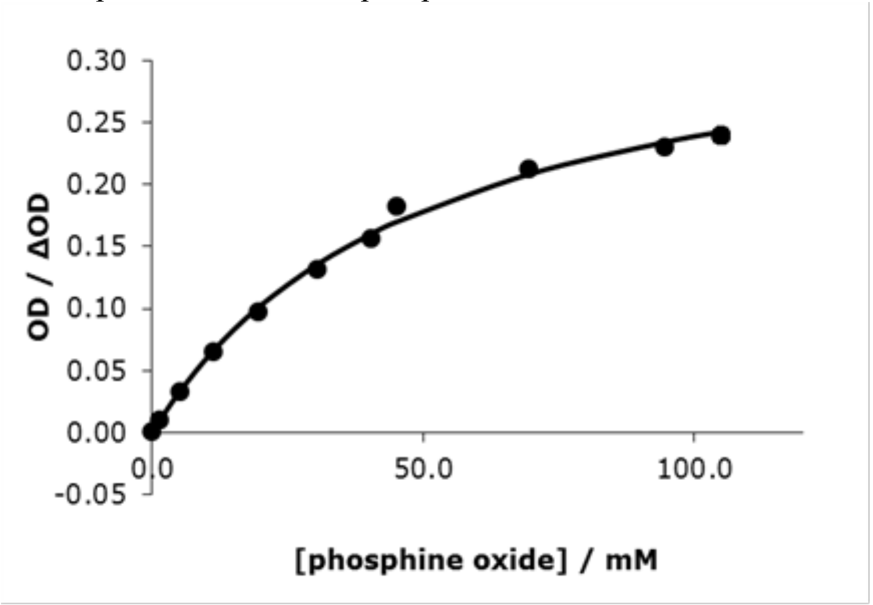

Graph S128. Binding isotherm for titration using 1:1 fitting program for titration of phosphine oxide against 5-nitroindole in chloroform. [5-nitroindole] = 0.09 mM and [phosphine oxide] = 116 mM, ΔOD = 0.357.

K_a = 18 ± 6 M^{-1} 68% bound

15. Titration Curves for phosphine oxide (2) in Acetone

a) Titration of 4-phenylazophenol with phosphine oxide in acetone

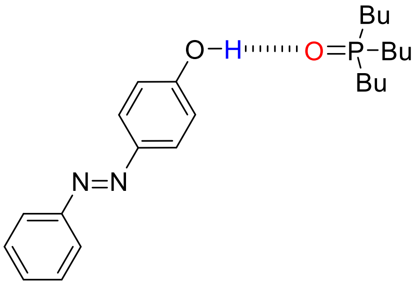

Host: 4-phenylazophenol = 0.052 mM
Guest: phosphine oxide = 166 mM

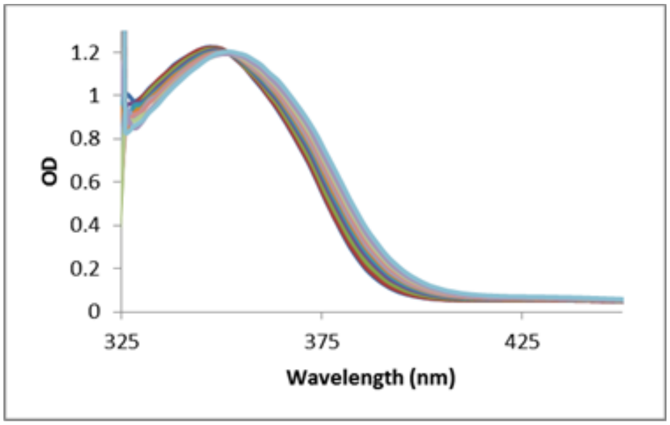

Graph S129. UV/vis spectra of titration of phosphine oxide with 4-phenylazophenol in acetone.

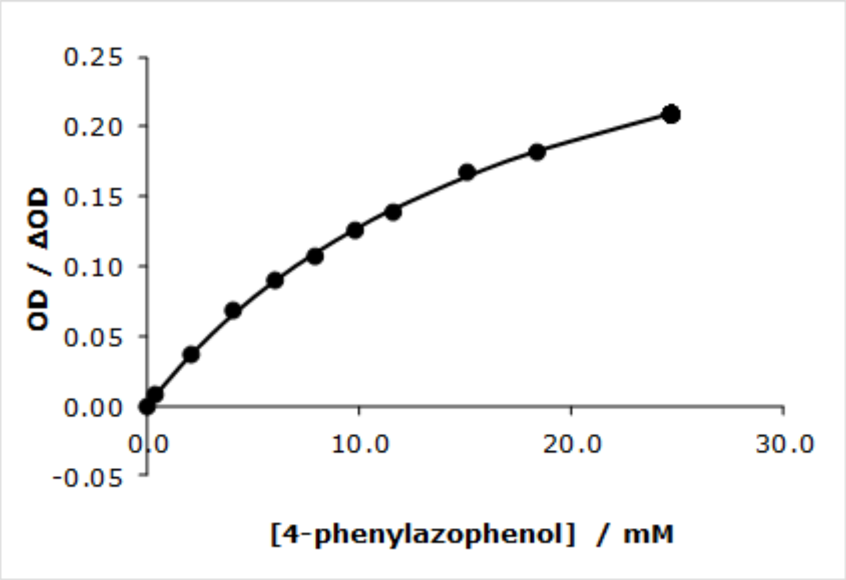

Graph S130. Binding isotherm for titration using 1:1 fitting program and accounting for a non-specific interaction for titration of phosphine oxide against 4-phenylazophenol in acetone. [4-phenylazophenol] = 0.052 mM and [phosphine oxide] = 166 mM, ΔOD = 0.366.

K_a = 48 ± 16 M^{-1} 57% bound

16. Titration Curves for phosphine oxide (2) in Carbon Tetrachloride

a) Titration of 4-phenylazophenol with phosphine oxide in carbon tetrachloride

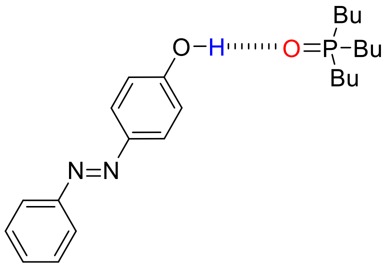

Host: 4-phenylazophenol = 0.029 mM
Guest: phosphine oxide = 34 mM

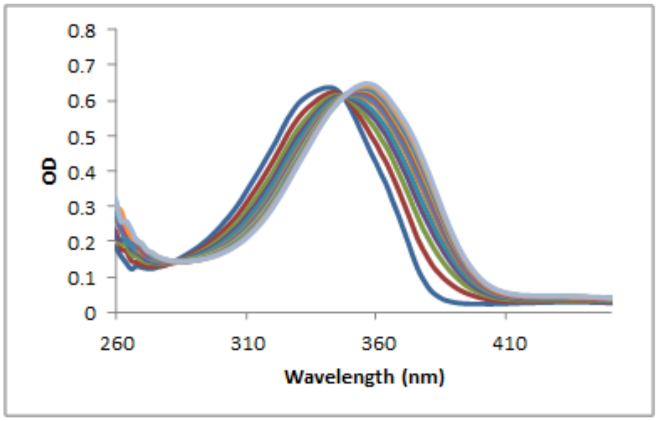

Graph S131. UV/vis spectra of titration of phosphine oxide with 4-phenylazophenol in carbon tetrachloride.

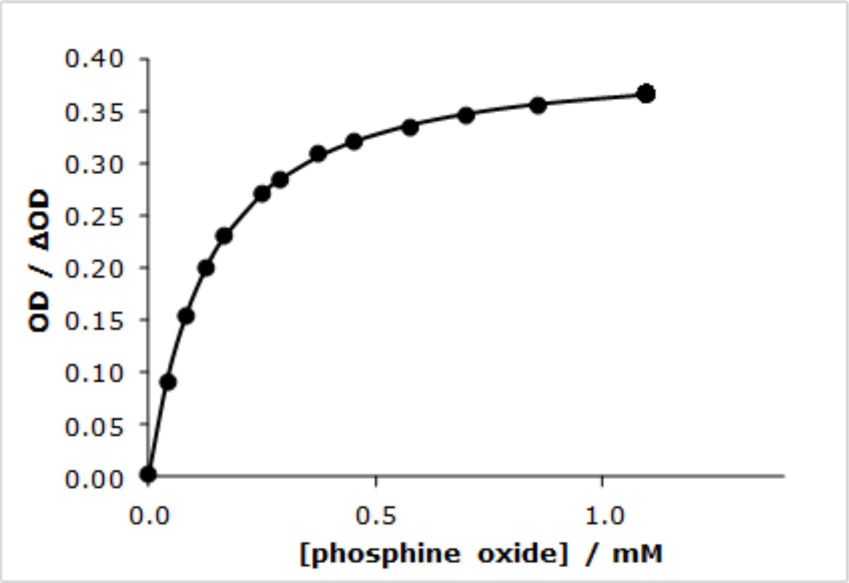

Graph S132. Binding isotherm for titration using 1:1 fitting program and accpunting for a non-specific interaction for titration of phosphine oxide against 4-phenylazophenol in CCl_4. [4-phenylazophenol] = 0.029 mM and [phosphine oxide] = 34 mM, ΔOD = 0.413.

K_a = 9200 ± 1900 M^{-1} 90% bound

b) Titration of 4-nitrophenol with phosphine oxide in carbon tetrachloride

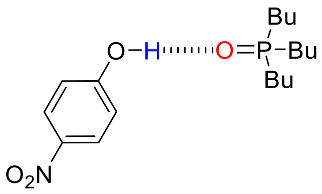

Host: 4-nitrophenol = 0.071 mM
Guest: phosphine oxide = 16 mM

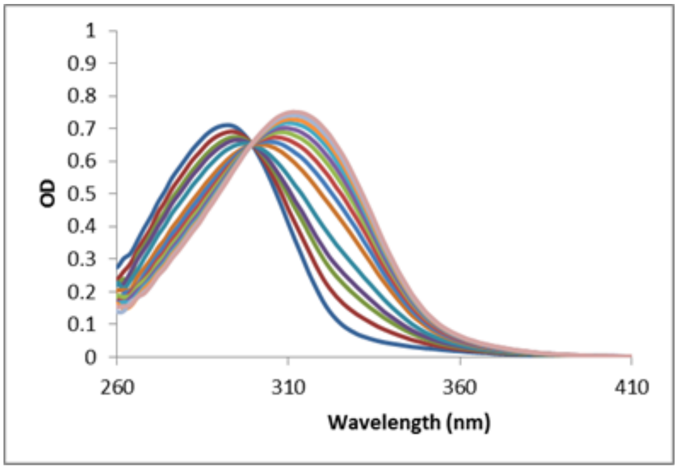

Graph S133. UV/vis spectra of titration of phosphine oxide with 4-nitrophenol in carbon tetrachloride.

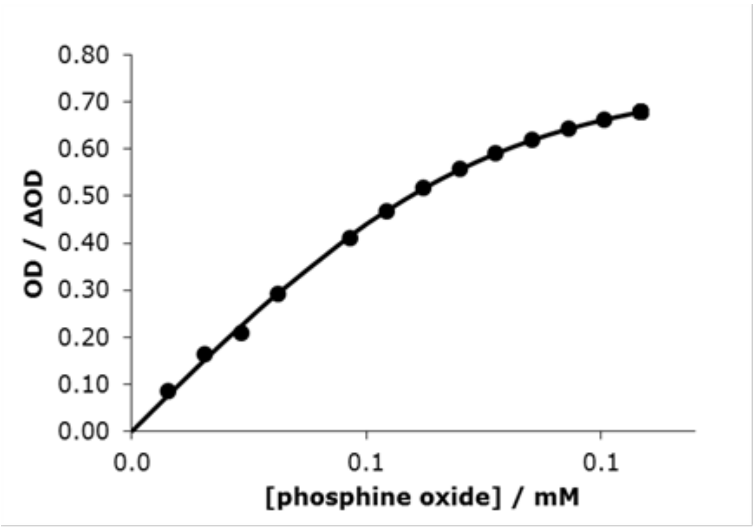

Graph S134. Binding isotherm for titration using 1:1 fitting program and accounting for a non-specific interaction for titration of phosphine oxide against 4-nitrophenol in carbon tetrachloride. [4-nitrophenol] = 0.071 mM and [phosphine oxide] = 16 mM, ΔOD = 0.818.

K_a = 98600 ± 19000 M^{-1} **83% bound**

c) Titration of 5-nitroindole with phosphine oxide in carbon tetrachloride

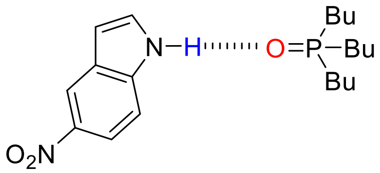

Host: 5-nitroindole = 0.08 mM
Guest: phosphine oxide = 140 mM

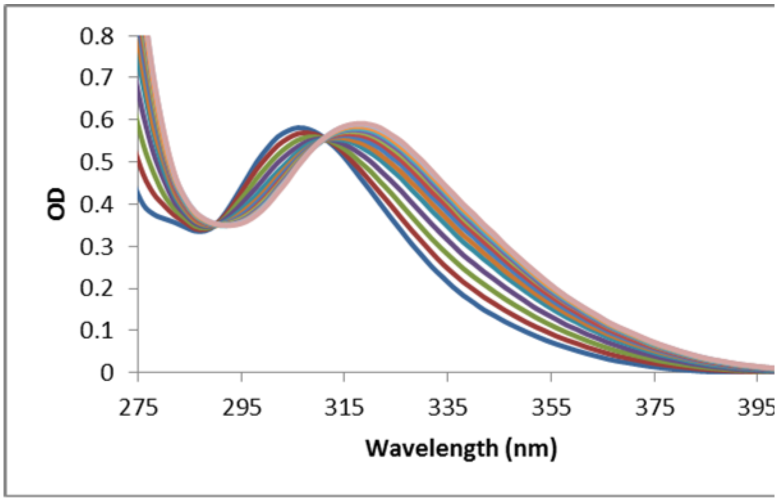

Graph S135. UV/vis spectra of titration of phosphine oxide with 5-nitroindole in carbon tetrachloride.

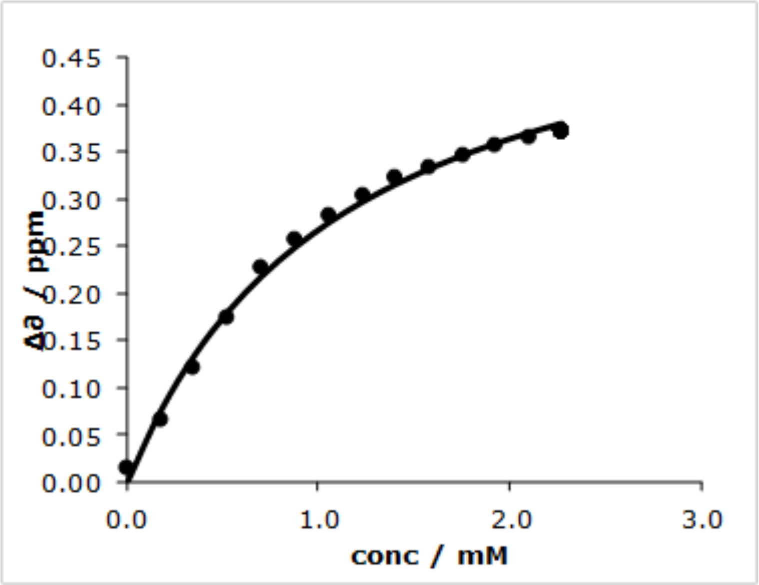

Graph S136. Binding isotherm for titration using 1:1 fitting program for titration of phosphine oxide against 5-nitroindole in carbon tetrachloride. [5-nitroindole] = 0.08 mM and [phosphine oxide] = 140 mM, ΔOD = 0.562.

K_a = 1200 ± 400 M^{-1} 68% bound

Titration Curves for Phosphine Oxide in Acetonitrile.

a) <u>Titration of phosphine oxide with phenol in acetonitrile</u>

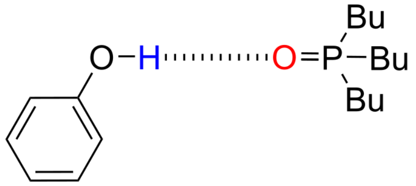

Host: phosphine oxide = 5.5 mM
Guest: phenol = 247 mM

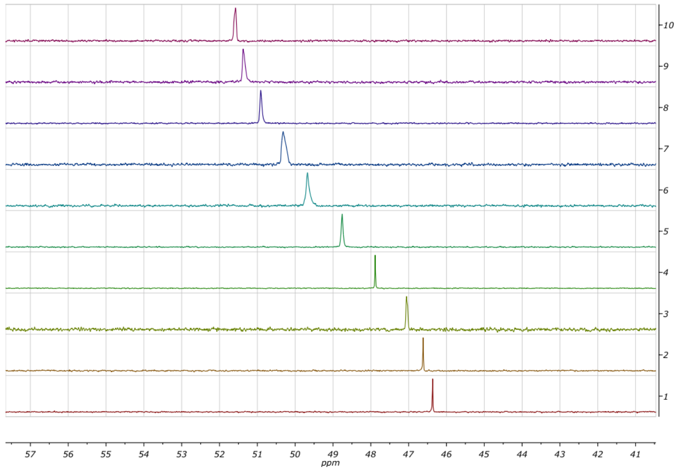

Graph S137. ^{31}P-NMR spectra of titration of phenol with phosphine oxide in acetonitrile.

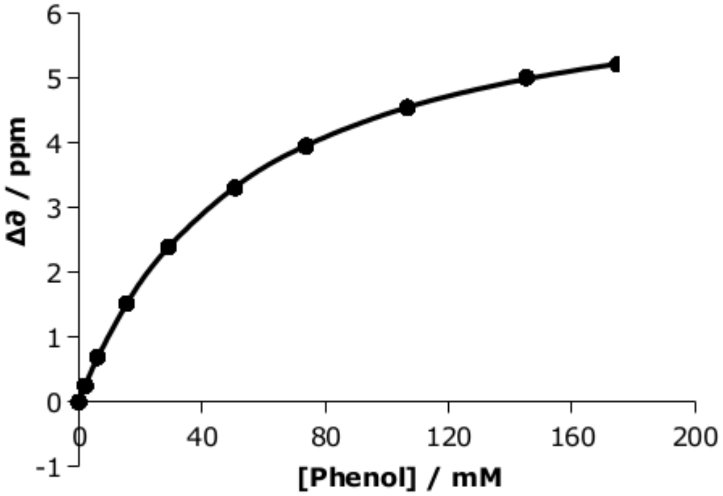

Graph S138. Binding isotherms for titration using 1:1 fitting program for titration of phenol against phosphine oxide in acetonitrile. [phosphine oxide] = 5.5 mM and [phenol] = 247 mM, Δppm = 5.21.

K_a = 20 ± 1 M^{-1} 78 % bound

b) Titration of phosphine oxide with 5-nitroindole in acetonitrile

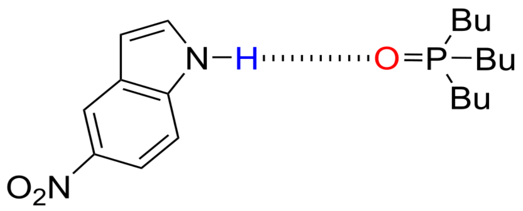

Host: phosphine oxide = 5.5 mM
Guest: 5-nitroindole = 480 Mm

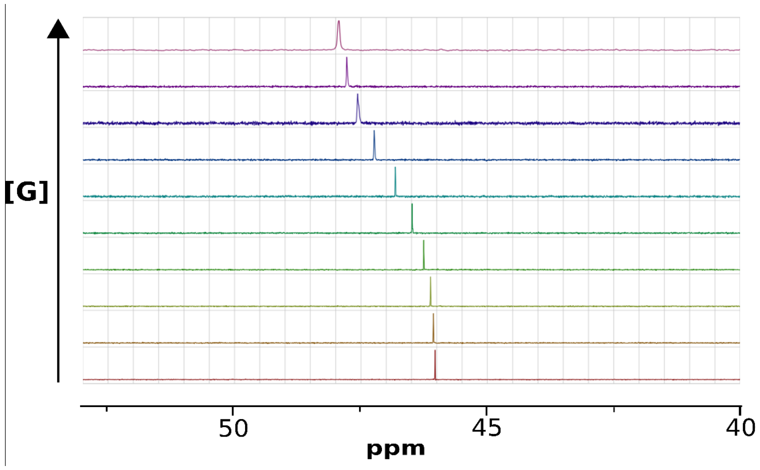

Graph S 39. P^{31}-NMR spectra of titration of 5-nitroindole with phosphine oxide in acetonitrile.

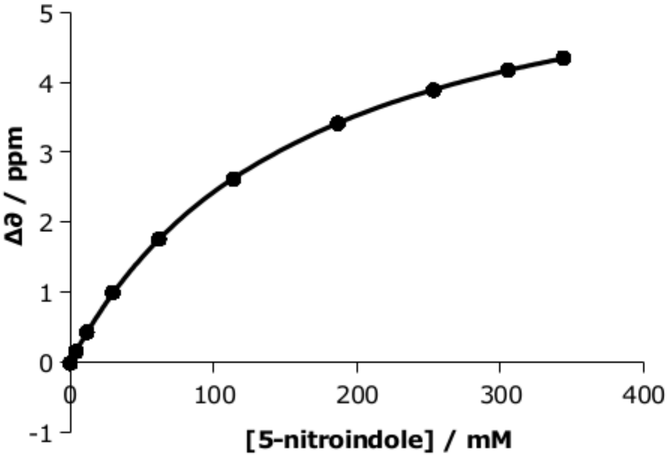

Graph S140. Binding isotherms for titration using 1:1 fitting program for titration of 5-nitroindole against phosphine oxide in acetonitrile. [phosphine oxide] = 5.5 mM and [5-nitroindole] = 480 mM, Δppm = 4.53.

K_a = 6 ± 1 M^{-1} **68% bound**

c) Titration of phosphine oxide with 4-phenylazophenol in acetonitrile

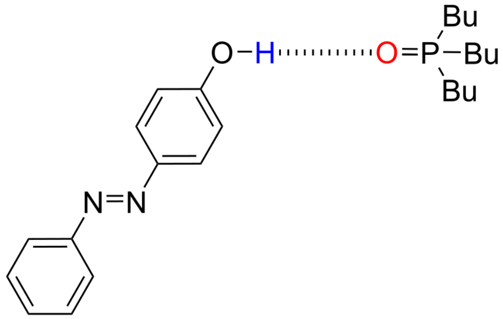

Host: phosphine oxide = 4.5 mM
Guest: 4-phenylazophenol = 70 mM

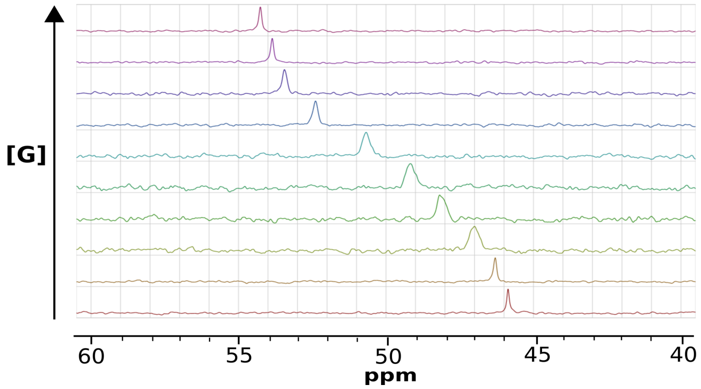

Graph S141. ^{31}P-NMR spectra of titration of 4-phenylazophenol with phosphine oxide in acetonitrile.

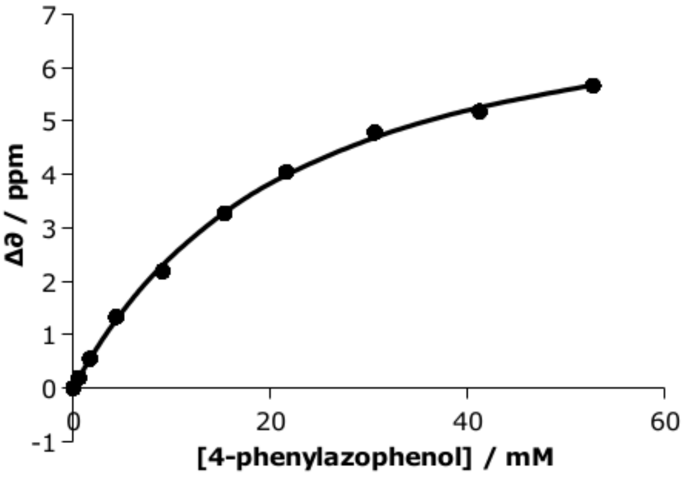

Graph S142. Binding isotherms for titration using 1:1 fitting program for titration of phenol against phosphine oxide in acetonitrile. [phosphine oxide] = 4.5 mM and [4-phenylazophenol] = 70 mM, Δppm = 5.66 .

K_a = 58 ± 4 M^{-1} 74% bound

d) Titration of phosphine oxide with 4-nitrophenol in acetonitrile

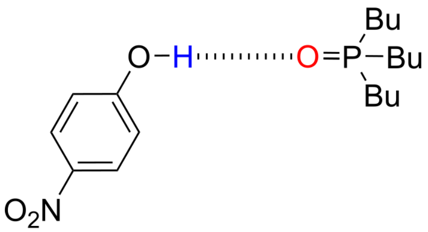

Host: phosphine oxide = 5.7 mM
Guest: 4-nitrophenol = 102 mM

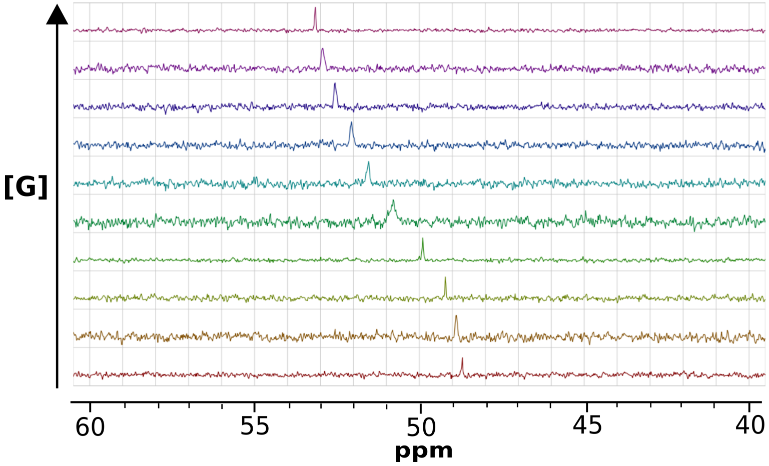

Graph S143. ^{31}P-NMR spectra of titration of 4-nitrophenol with phosphine oxide in acetonitrile.

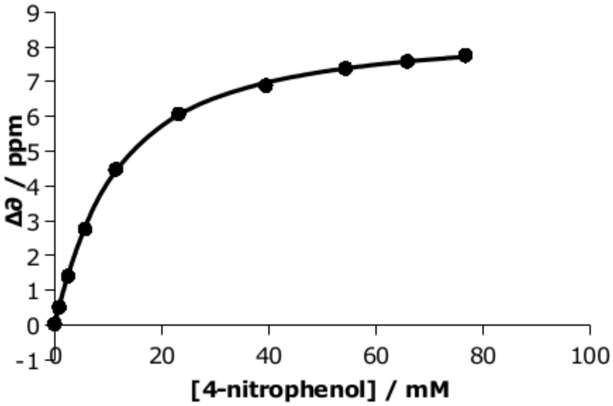

Graph S144. Binding isotherms for titration using 1:1 fitting program for titration of 4-nitrophenol against phosphine oxide in acetonitrile. [phosphine oxide] = 5.7 mM and [4-nitrophenol] = 102 mM, Δppm = 7.08.

K_a = 102 ± 34 M^{-1} 73% bound

e) Titration of phosphine oxide with 4-nitro,3-fluoromethylphenol in acetonitrile

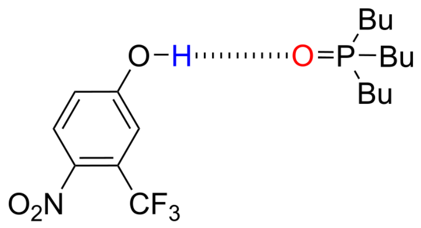

Host: phosphine oxide = 5.0 mM
Guest: 4-nitro,3-fluoromethylphenol = 52 mM

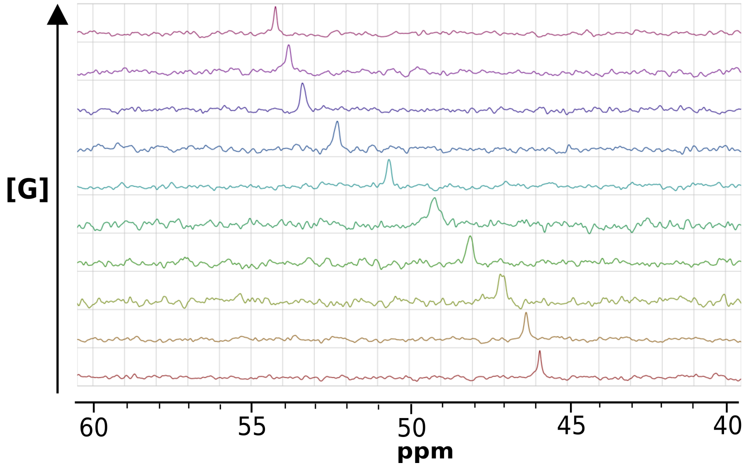

Graph S145. ^{31}P-NMR spectra of titration of 4-nitro,3-fluoromethylphenol with phosphine oxide in acetonitrile.

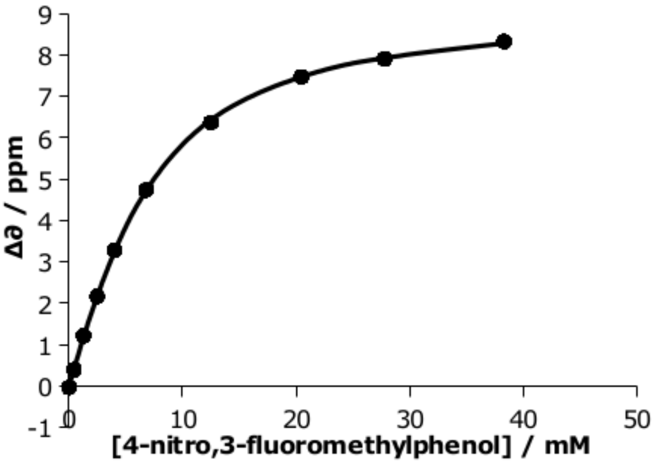

Graph S146. Binding isotherms for titration using 1:1 fitting program for titration of phenol against phosphine oxide in acetonitrile. [phosphine oxide] = 5.0 mM and [4-nitro,3-fluoromethylphenol] = 52 mM, Δppm = 8.32.

K_a = 266 ± 22 M^{-1} 90% bound

g) Titration of phosphine oxide with $AgBF_4$ in acetonitrile

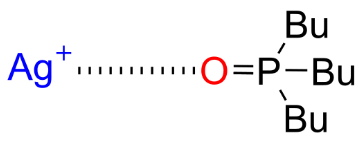

Host: phosphine oxide = 5.6 mM
Guest: $AgBF_4$ = 310 mM

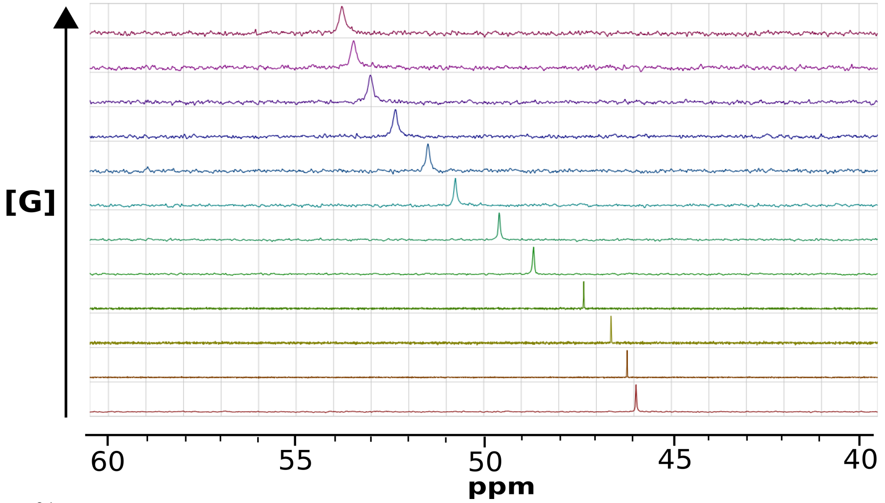

Graph S147. P^{31}-NMR spectra of titration of $AgBF_4$ with phosphine oxide in acetonitrile.

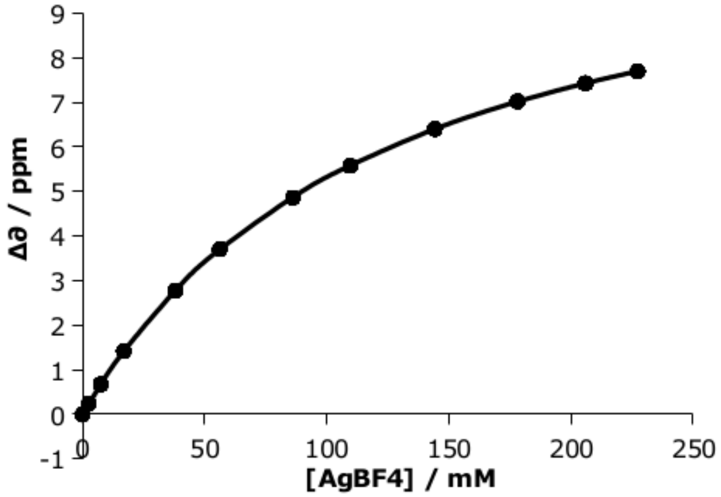

Graph S148. Binding isotherms for titration using 1:1 fitting program for titration of AgBPh4 against phosphine oxide in acetonitrile. [phosphine oxide] = 5.6 mM and [$AgBF_4$] = 310 mM, Δppm = 7.84 .

K_a = 8 ± 1 M^{-1} 65 % bound

h) <u>Titration of phosphine oxide with GuanidiniumBPh$_4$ in acetonitrile</u>

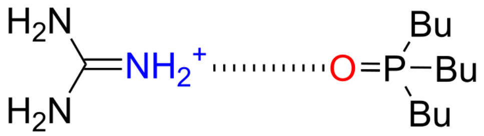

Host: phosphine oxide = 5.1 mM
Guest: GuanidiniumBPh4 = 35 mM

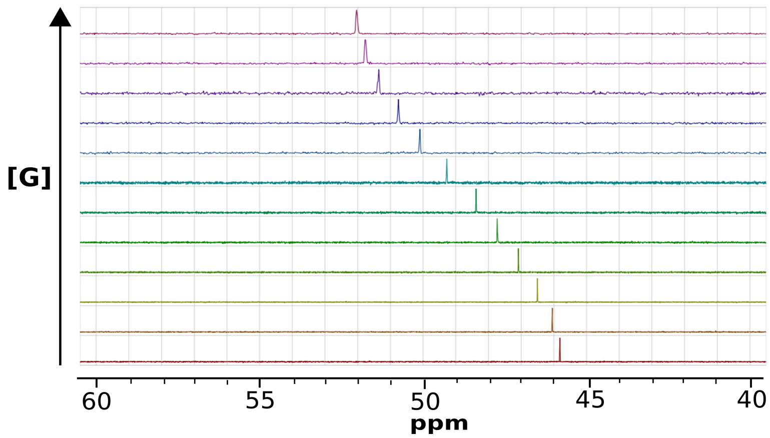

Graph S149. ^{31}P-NMR spectra of titration of GuanidiniumBPh4 with phosphine oxide in acetonitrile.

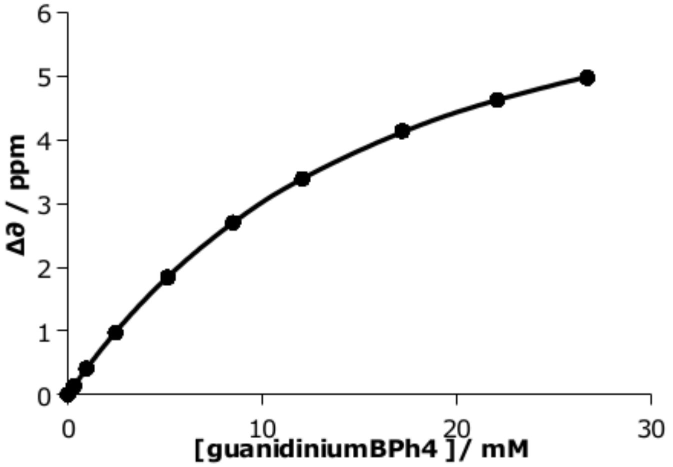

Graph S150. Binding isotherms for titration using 1:1 fitting program for titration of GuanidiniumBPh4 against phosphine oxide in acetonitrile. [phosphine oxide] = 5.1 mM and [4-GuanidiniumBPh4] = 35 mM, Δppm = 6.22 .

K_a = 198 ± 61 M^{-1} 85 % bound

i) Titration of phosphine oxide with Et_3NHBPh_4 in acetonitrile

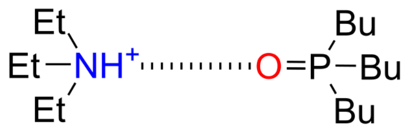

Host: phosphine oxide = 5.0 mM
Guest: Et_3NHBPh_4 = 39 mM

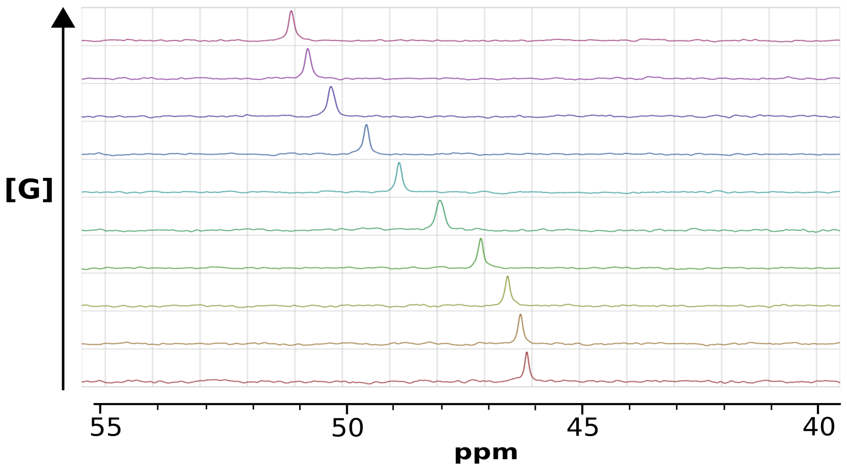

Graph S151. ^{31}P-NMR spectra of titration of Et_3NHBPh_4 with phosphine oxide in acetonitrile.

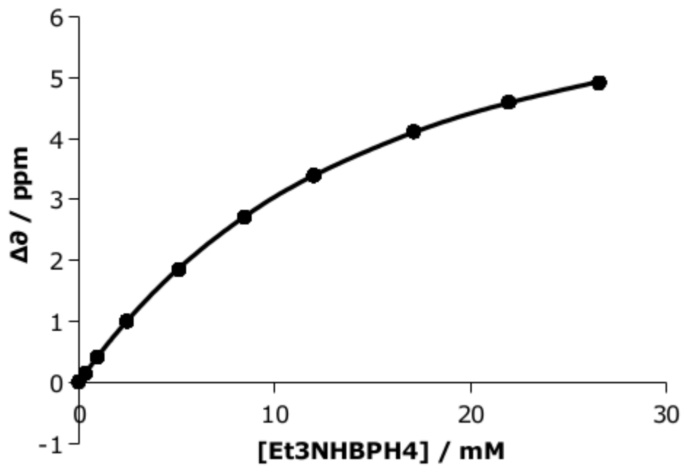

Graph S152. Binding isotherms for titration using 1:1 fitting program for titration of Et_3NHBPh_4 against phosphine oxide in acetonitrile. [phosphine oxide] = 5.0 mM and [Et_3NHBPh_4] = 39 mM, Δppm = 4.91.

K_a = 85 ± 6 M^{-1} 66 % bound

j) Titration of phosphine oxide with 1-MethylimidazoliumBPh4 in acetonitrile

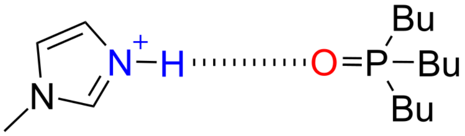

Host: phosphine oxide = 5.0 mM
Guest: 1-MethylimidazoliumBPh4 = 40 mM

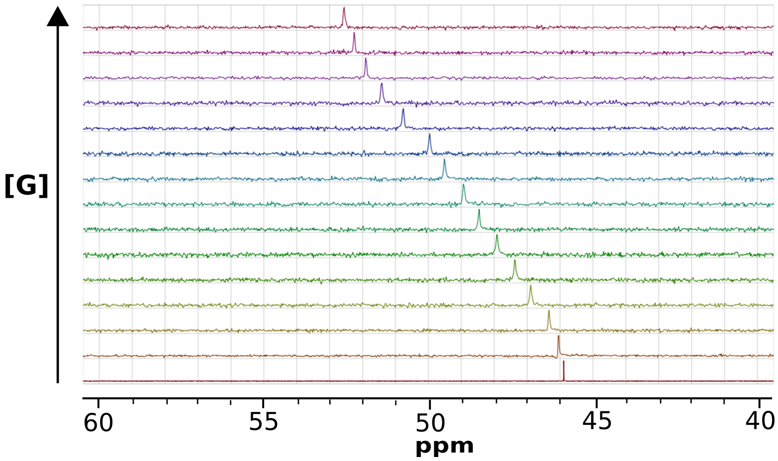

Graph S153. ^{31}P-NMR spectra of titration of 1-MethylimidazoliumBPh4 with phosphine oxide in acetonitrile.

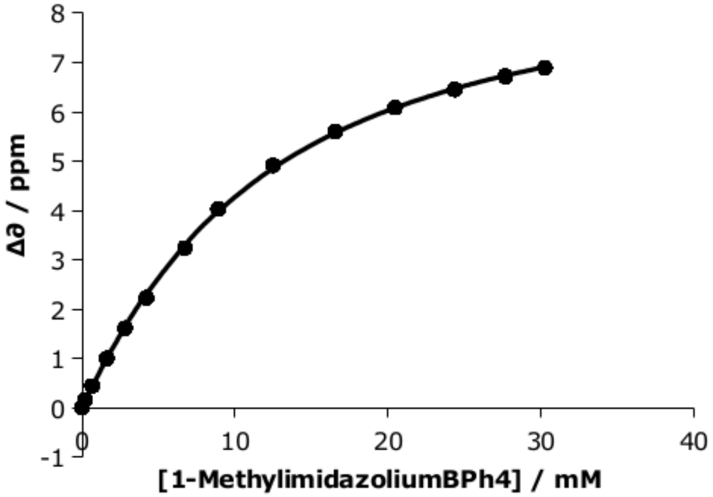

Graph S154. Binding isotherms for titration using 1:1 fitting program for titration of 1-MethylimidazoliumBPh4 against phosphine oxide in acetonitrile. [phosphine oxide] = 5.0 mM and [1-MethylimidazoliumBPh4] = 40 mM, Δppm = 6.89 .

K_a = 102 ± 12 M^{-1} 75% bound

k) Titration of phosphine oxide with 2-EthylhexylammoniumBPh_4 in acetonitrile

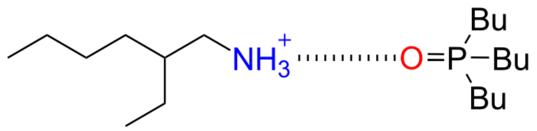

Host: phosphine oxide = 5.7 mM
Guest: 2-EthylhexylammoniumBPh_4 = 50 mM

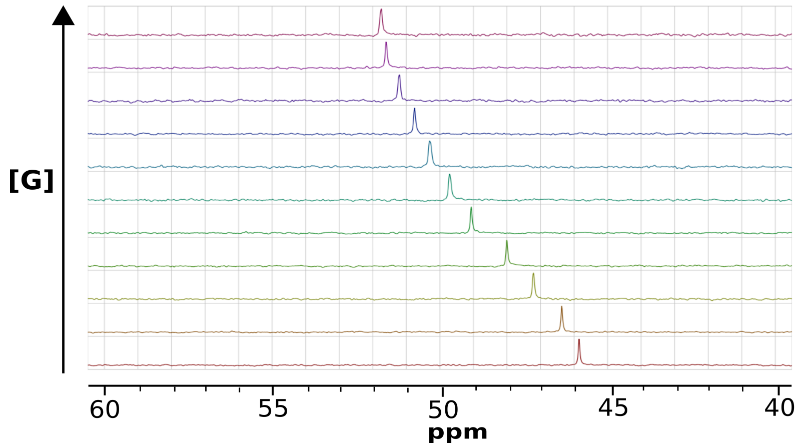

Graph S155. P^{31}-NMR spectra of titration of 2-EthylhexylammoniumBPh_4 with phosphine oxide in acetonitrile.

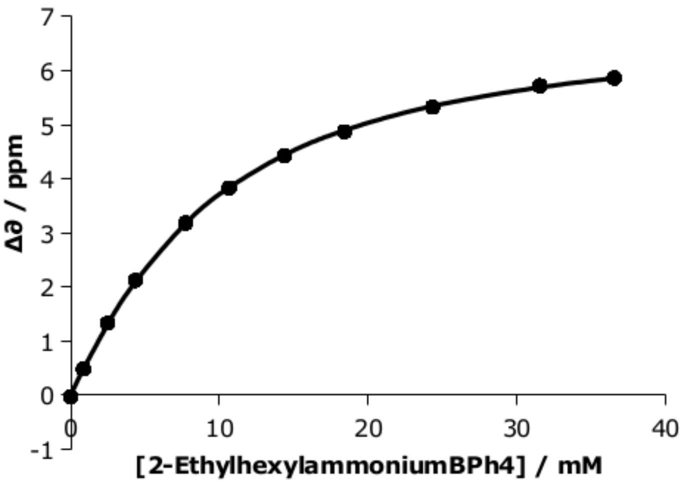

Graph S156. Binding isotherms for titration using 1:1 fitting program for titration of 2-EthylhexylammoniumBPh4 against phosphine oxide in acetonitrile. [phosphine oxide] = 5.7 mM and [2-EthylhexylammoniumBPh_4] = 50 mM, Δppm = 5.90 .

K_a = 147 ± 25 M^{-1} 78 % bound

l) Titration of phosphine oxide with IPh_2BF_4 in acetonitrile

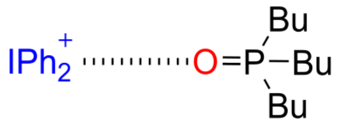

Host: phosphine oxide = 5.3 mM
Guest: IPh_2BF_4 = 48 mM

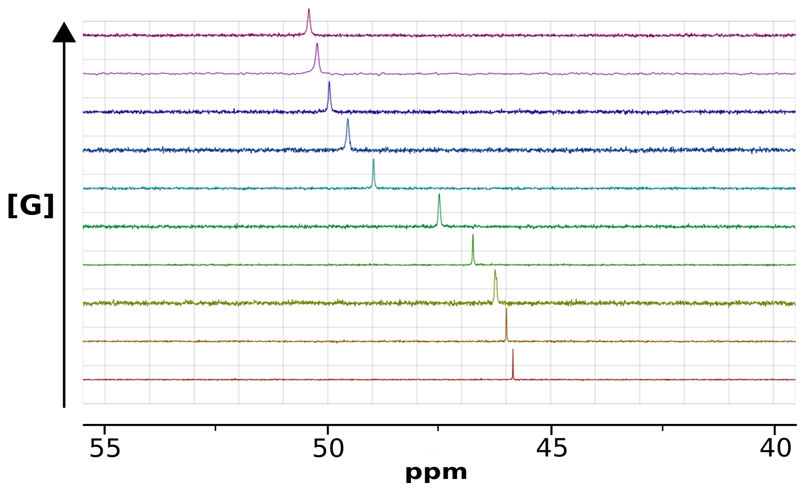

Graph S157. ^{31}P-NMR spectra of titration of IPh_2BF_4 with phosphine oxide in acetonitrile.

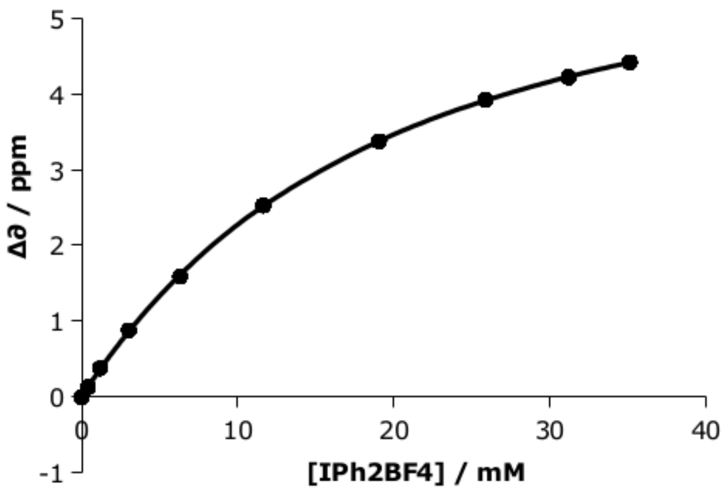

Graph S158. Binding isotherms for titration using 1:1 fitting program for titration of IPh_2BF_4 against phosphine oxide in acetonitrile. [phosphine oxide] = 5.3 mM and [IPh_2BF_4] = 48 mM, Δppm = 4.43.

K_a = 62 ± 3 M^{-1} 67 % bound

m) <u>Titration of phosphine oxide with TriphenylmethylPF_6 in acetonitrile</u>

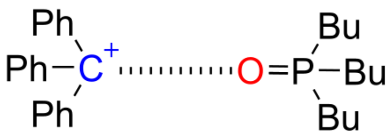

Host: phosphine oxide = 5.2 mM
Guest: TriphenylmethylPF_6 = 54 mM

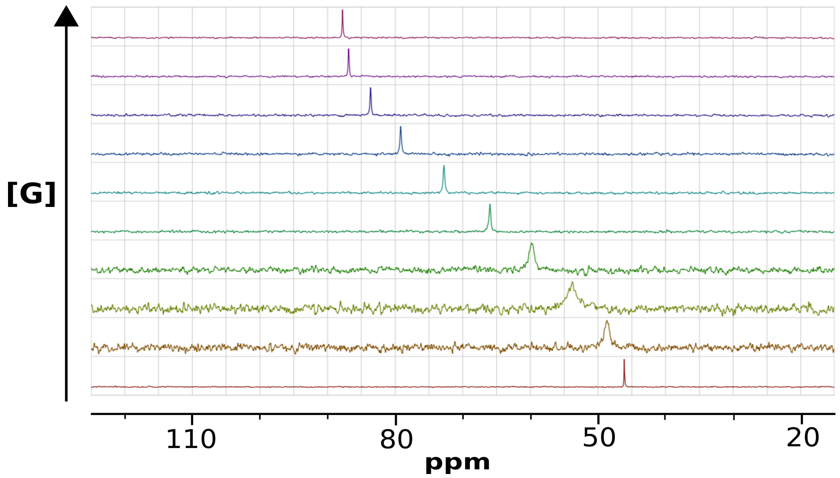

Graph S159. ^{31}P-NMR spectra of titration of TriphenylmethylPF_6 with phosphine oxide in acetonitrile.

n) Titration of phosphine oxide with $NaBF_4$ in acetonitrile

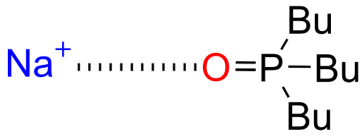

Host: phosphine oxide = 5.70 mM
Guest: $NaBF_4$ = 57 mM

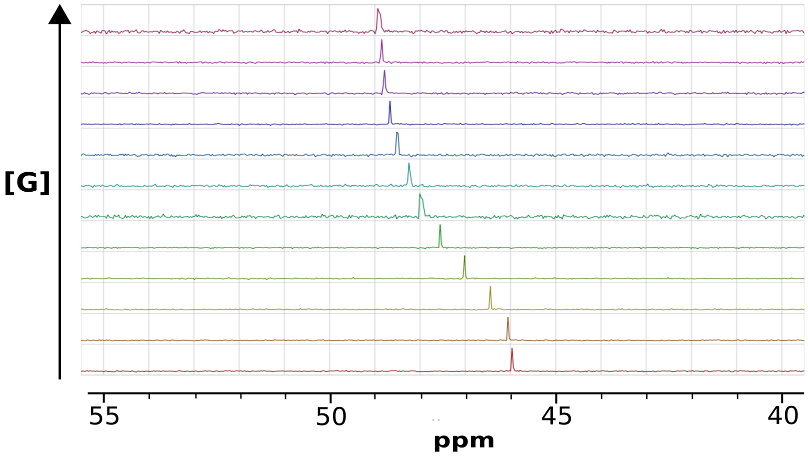

Graph S160. ^{31}P-NMR spectra of titration of $NaBF_4$ with phosphine oxide in acetonitrile.

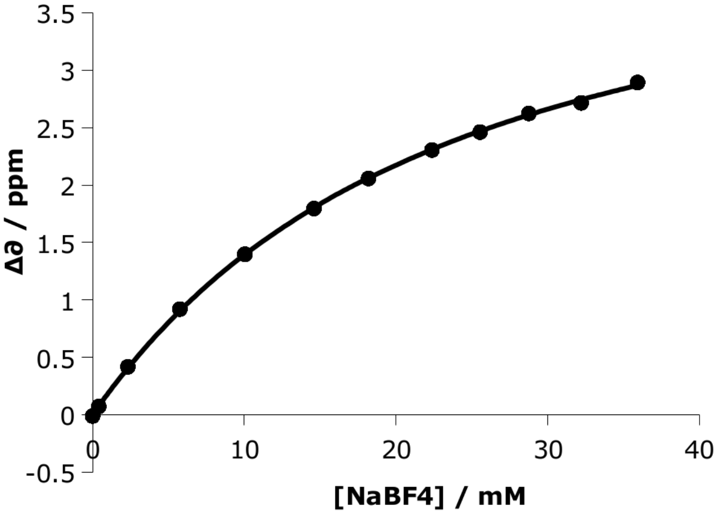

Graph S161. Binding isotherms for titration using 1:1 fitting program for titration of 1-$NaBF_4$ against phosphine oxide in acetonitrile. [phosphine oxide] = 5.7 mM and [$NaBF_4$] = 57 mM, Δppm = 2.98.

K_a = 57 ± 6 M^{-1} 68 % bound

o) Titration of phosphine oxide with $NaBAr^F$ in acetonitrile

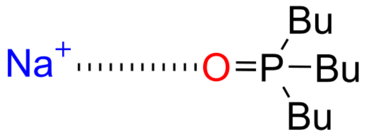

Host: phosphine oxide = 5.2 mM
Guest: $NaBAr^F$ = 50 mM

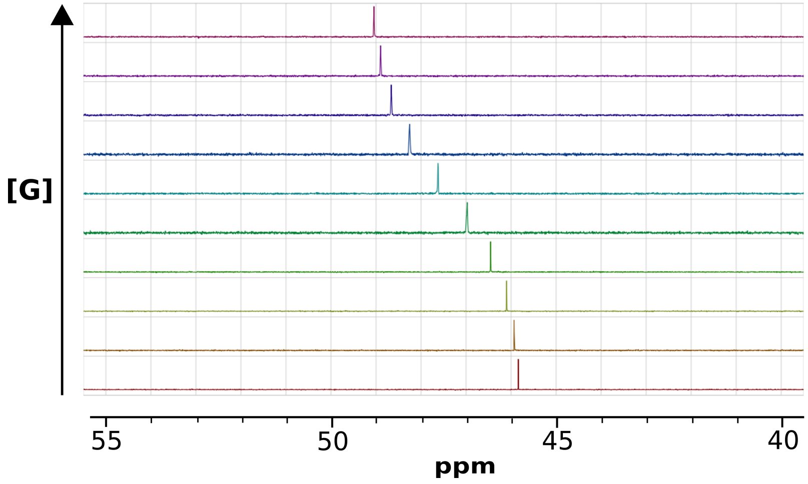

Graph S162. ^{31}P-NMR spectra of titration of $NaBAr^F$ with phosphine oxide in acetonitrile.

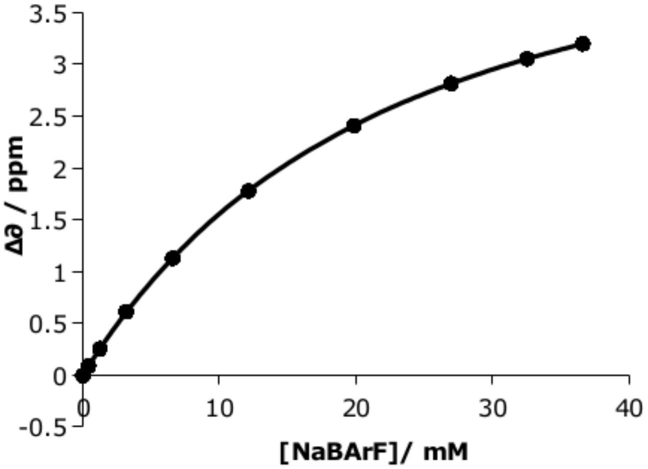

Graph S163. Binding isotherms for titration using 1:1 fitting program for titration of NaBArF against phosphine oxide in acetonitrile. [phosphine oxide] = 5.2 mM and [$NaBAr^F$] = 50 mM, Δppm = 3.2 .

K_a = 53 ± 3 M^{-1} 64 % bound

p) Titration of phosphine oxide with $NaN(SO_2CF_3)_2$ in acetonitrile

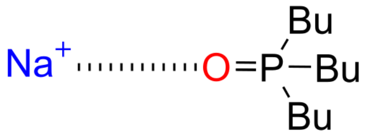

Host: phosphine oxide = 5.2 mM
Guest: $NaN(SO_2CF_3)_2$ = 49 mM

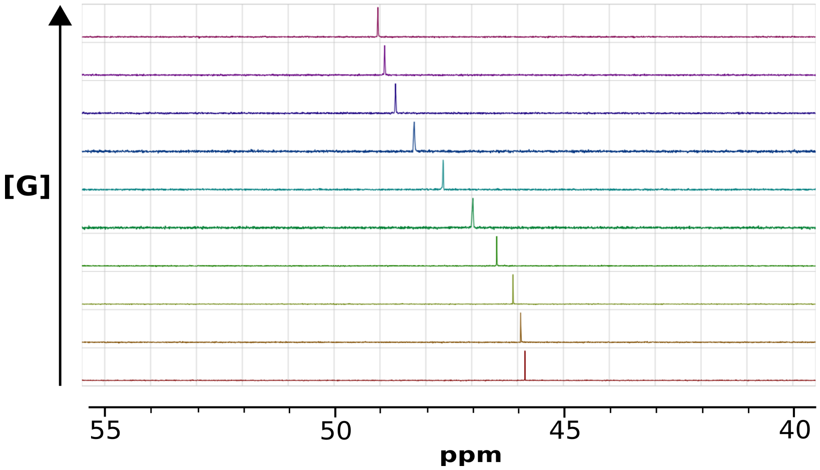

Graph S164. ^{31}P-NMR spectra of titration of $NaN(SO_2CF_3)_2$ with phosphine oxide in acetonitrile.

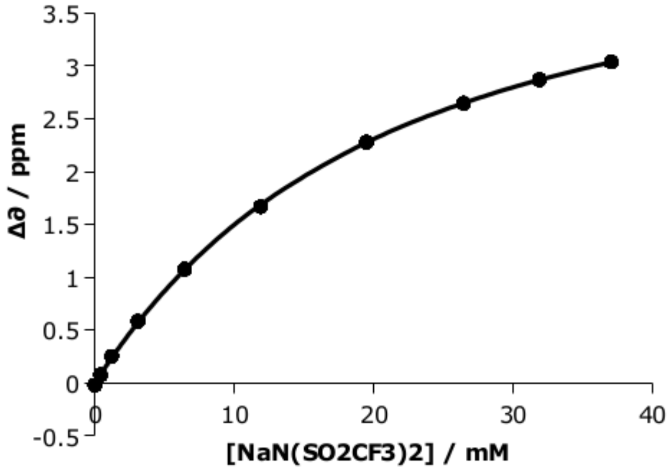

Graph S165. Binding isotherms for titration using 1:1 fitting program for titration of $NaN(SO_2CF_3)_2$ against phosphine oxide in acetonitrile. [phosphine oxide] = 4.9 mM and [$NaN(SO_2CF_3)_2$] = 49 mM, Δppm = 3.05.

K_a = 56 ± 5 M^{-1} 66 % bound

q) Titration of phosphine oxide with NaI in acetonitrile

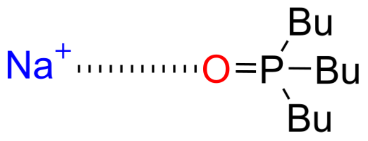

Host: phosphine oxide = 5.2 mM
Guest: NaI = 49 mM

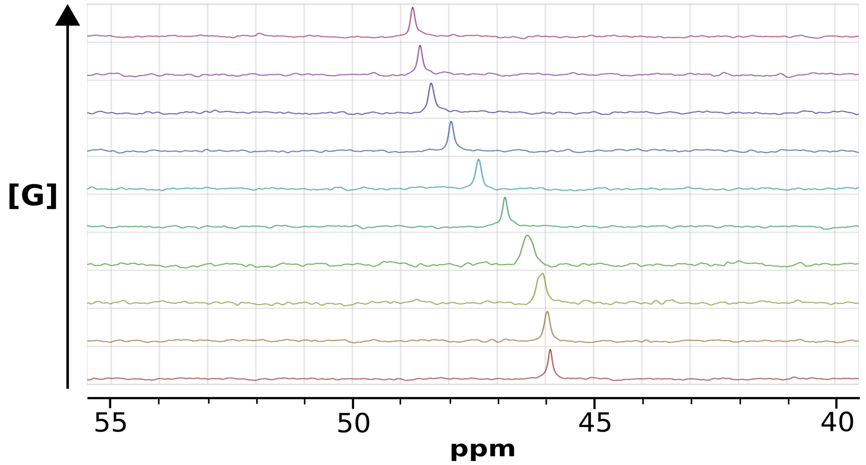

Graph S166. ^{31}P-NMR spectra of titration of NaI with phosphine oxide in acetonitrile.

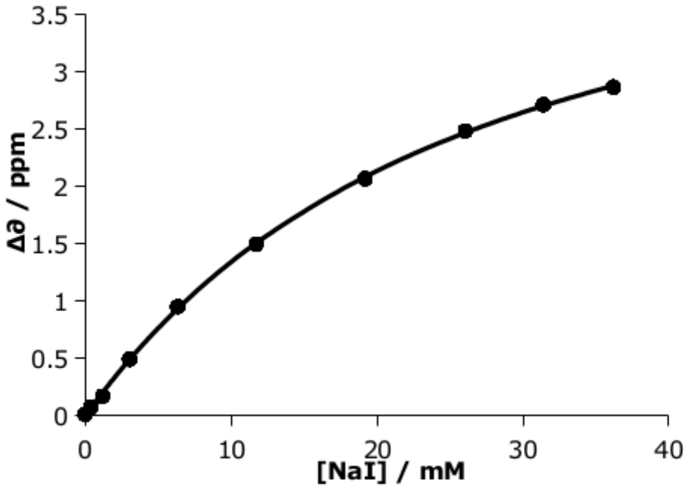

Graph S167. Binding isotherms for titration using 1:1 fitting program for titration of NaI against phosphine oxide in acetonitrile. [phosphine oxide] = 5.2 mM and [NaI] = 49 mM, Δppm = 2.85.

K_a = 45 ± 1 M^{-1} 60 % bound

Titration Curves for Phosphine Oxide in Acetone.

a) Titration of phosphine oxide with phenol in acetone

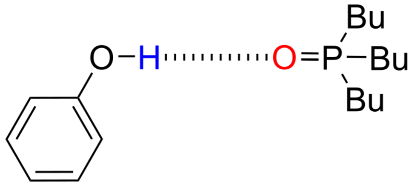

Host: phosphine oxide = 5.00 mM
Guest: = 253 phenol mM

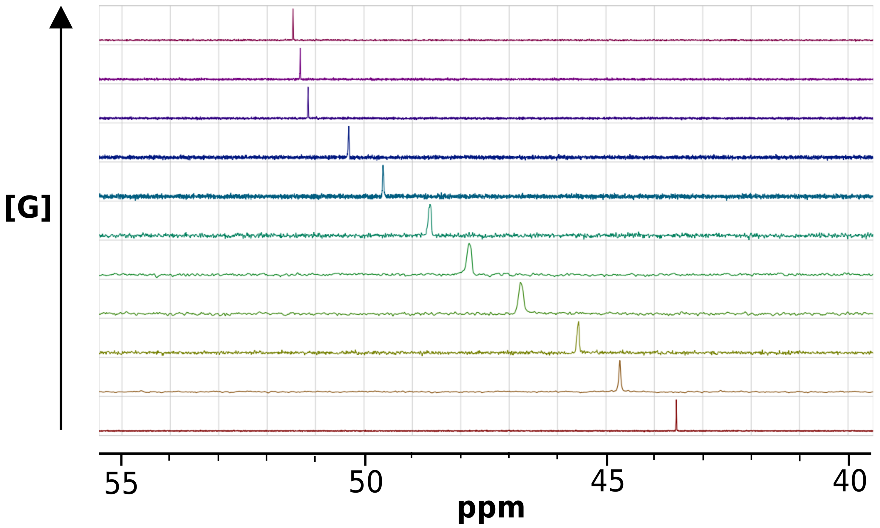

Graph S168. [31] P-NMR spectra of titration of phenol with phosphine oxide in acetonitrile.

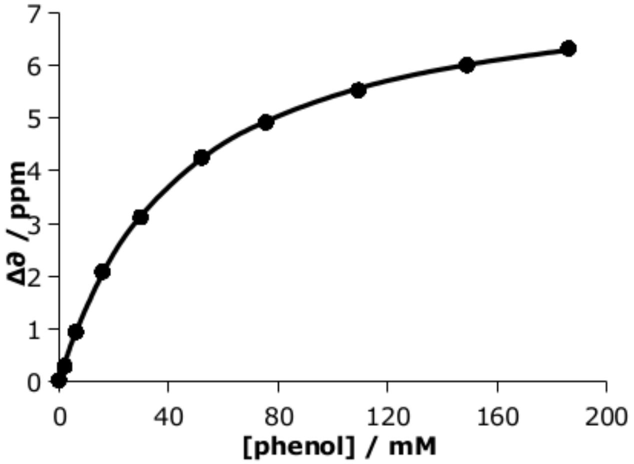

Graph S169. Binding isotherms for titration using 1:1 fitting program for titration of phenol against phosphine oxide in acetonitrile. [phosphine oxide] = 5.00 mM and [phenol] = 253 mM, Δppm = 6.29.

K_a = 26 ± 1 M^{-1} % bound

b) Titration of phosphine oxide with 5-nitroindole in acetone

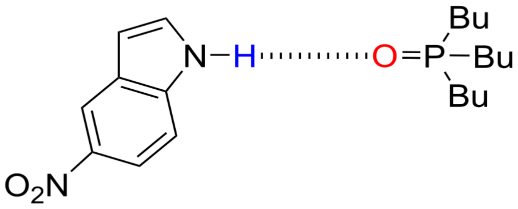

Host: phosphine oxide = 5.2 mM
Guest: 5-nitroindole = 501 mM

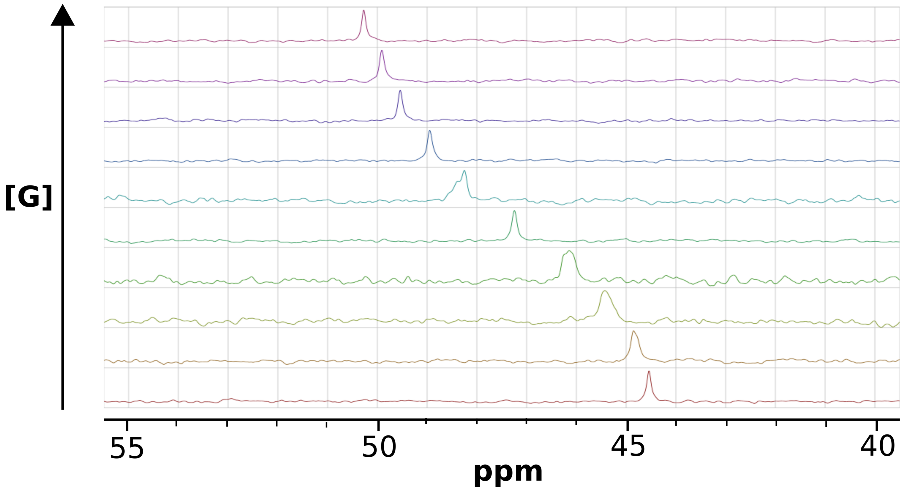

Graph S170. ^{31}P-NMR spectra of titration of 5-nitroindole with phosphine oxide in acetonitrile.

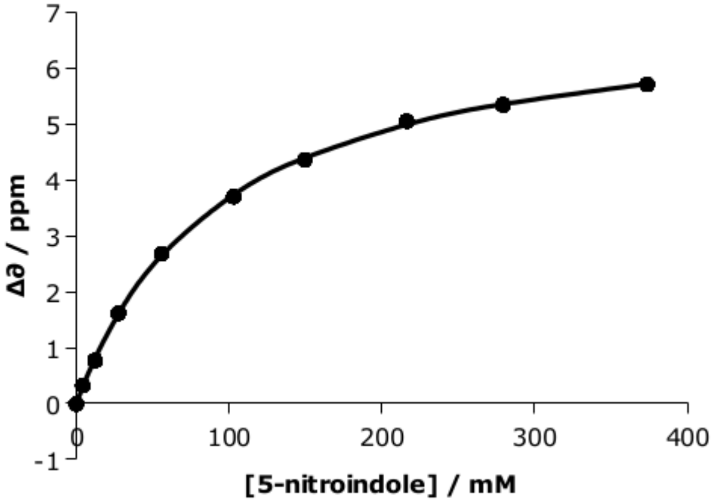

Graph S171. Binding isotherms for titration using 1:1 fitting program for titration of 5-nitroindole against phosphine oxide in acetonitrile. [phosphine oxide] = 5.2 mM and [5-nitroindole] = 501 mM, Δppm = 5.63.

K_a = 11 ± 1M^{-1} 80 % bound

c) Titration of phosphine oxide with 4-phenylazophenol in acetone

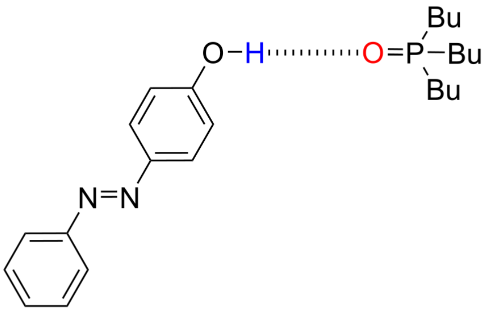

Host: phosphine oxide = 5.6 mM
Guest: 4-phenylazophenol = 73 mM

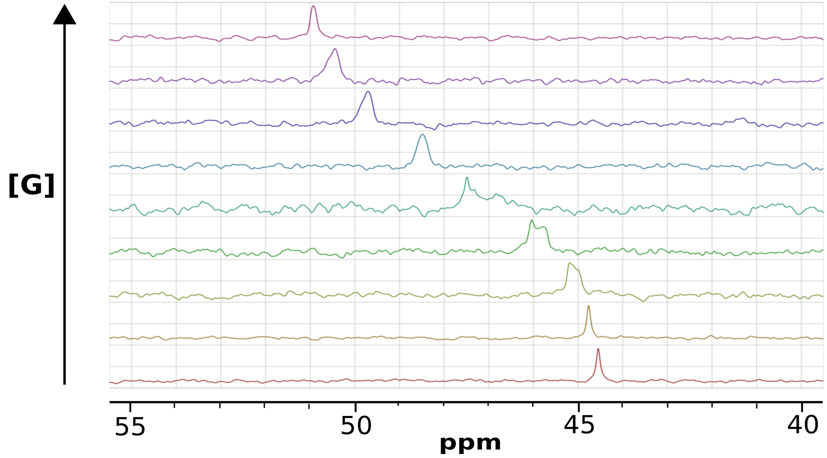

Graph S172. ^{31}P-NMR spectra of titration of 4-phenylazophenol with phosphine oxide in acetone.

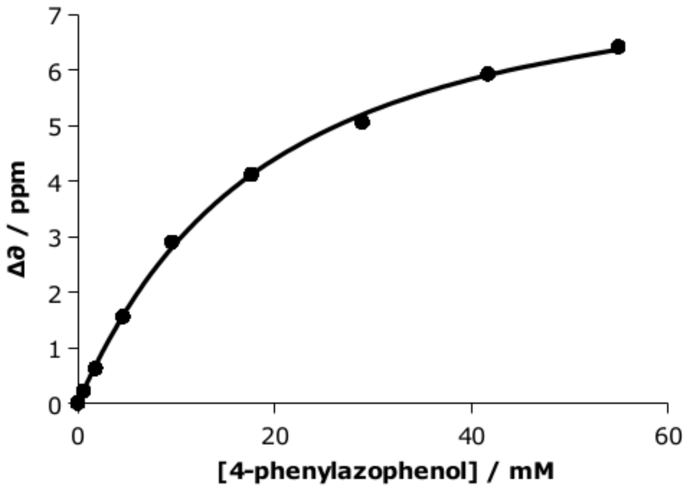

Graph S173. Binding isotherms for titration using 1:1 fitting program for titration of against phosphine oxide in acetone. [phosphine oxide] = 5.6 mM and [4-phenylazophenol] = 73 mM, Δppm = 6.15.

K_a =   65 ± 6 M^{-1} 76% bound

d) Titration of phosphine oxide with 4-nitrophenol in acetone

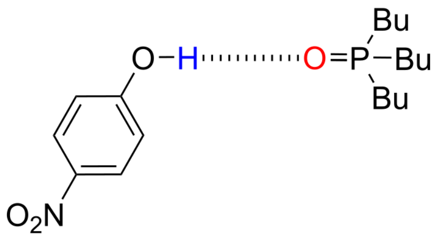

Host: phosphine oxide = 5.2 mM
Guest: 4-nitrophenol = 99 mM

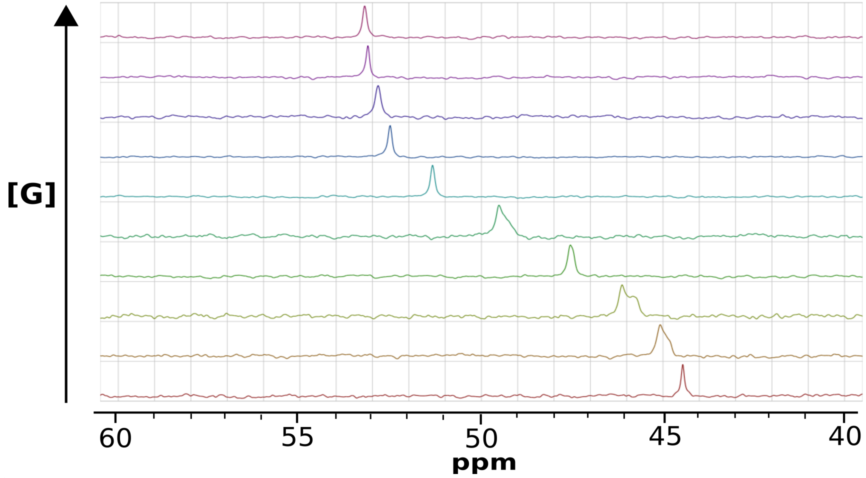

Graph S174. ^{31}P-NMR spectra of titration of 4-nitrophenol with phosphine oxide in acetone.

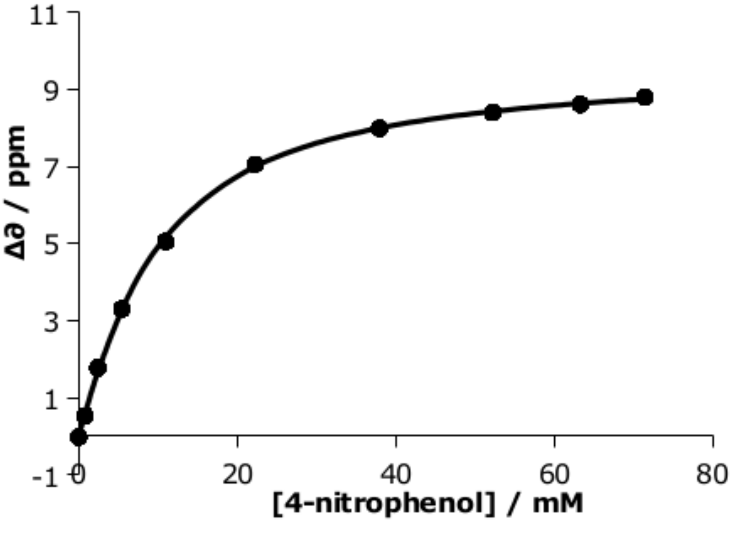

Graph S175. Binding isotherms for titration using 1:1 fitting program for titration of 4-nitrophenol against phosphine oxide in acetone. [phosphine oxide] = 5.2 mM and [4-nitrophenol] = 99 mM, Δppm = 8.76.

K_a = 134 ± 18 M^{-1} 89 % bound

e) Titration of phosphine oxide with 4-nitro,3-trifluoromethylphenol in acetone

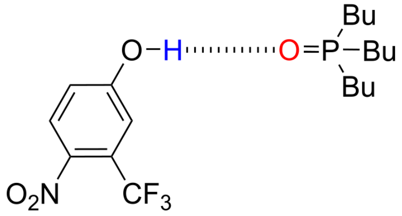

Host: phosphine oxide = 5.0 mM
Guest: 4-nitro,3-trifluoromethylphenol = 48 mM

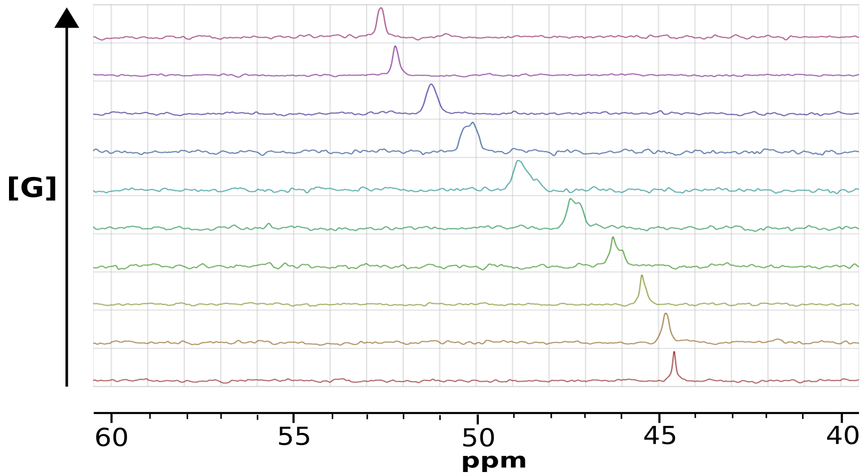

Graph S176. ^{31}P-NMR spectra of titration of 4-nitro,3-trifluoromethylphenol with phosphine oxide in acetone.

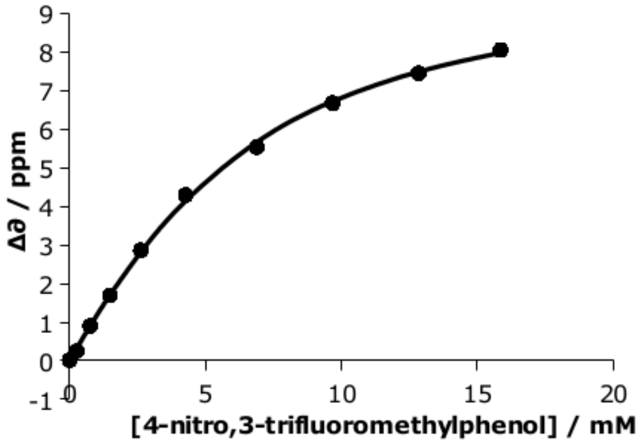

Graph S177. Binding isotherms for titration using 1:1 fitting program for titration of 4-nitro,3-trifluoromethylphenol against phosphine oxide in acetone. [phosphine oxide] =5.0 mM and [4-nitro,3-trifluoromethylphenol] = 48 mM, Δppm = 9.48 .

K_a = 282 ± 108 M^{-1} 90 % bound

f) Titration of phosphine oxide with $NaBPh_4$ in acetone

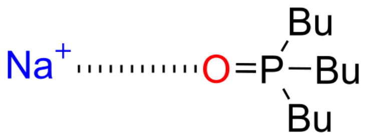

Host: phosphine oxide = 5.0 mM
Guest: $NaBPh_4$ = 51 mM

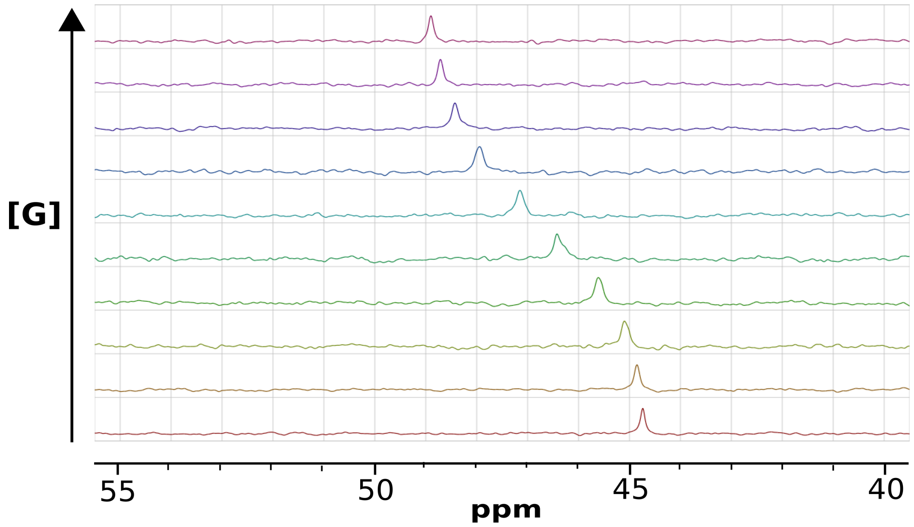

Graph S178. ^{31}P-NMR spectra of titration of NaBPh4 with phosphine oxide in acetone.

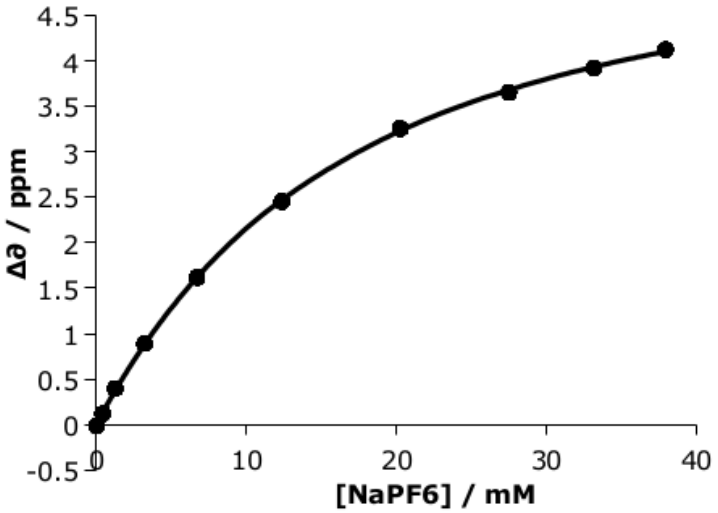

Graph S179. Binding isotherms for titration using 1:1 fitting program for titration of $NaBPh_4$ against phosphine oxide in acetone. [phosphine oxide] = 5.0 mM and [$NaBPh_4$] = 51 mM, Δppm = 4.06.

K_a = 73 ± 5 M^{-1} 71 % bound

g) Titration of phosphine oxide with $AgBF_4$ in acetone

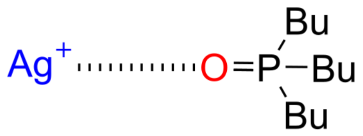

Host: phosphine oxide = 4.9 mM
Guest: $AgBF_4$ = 80 mM

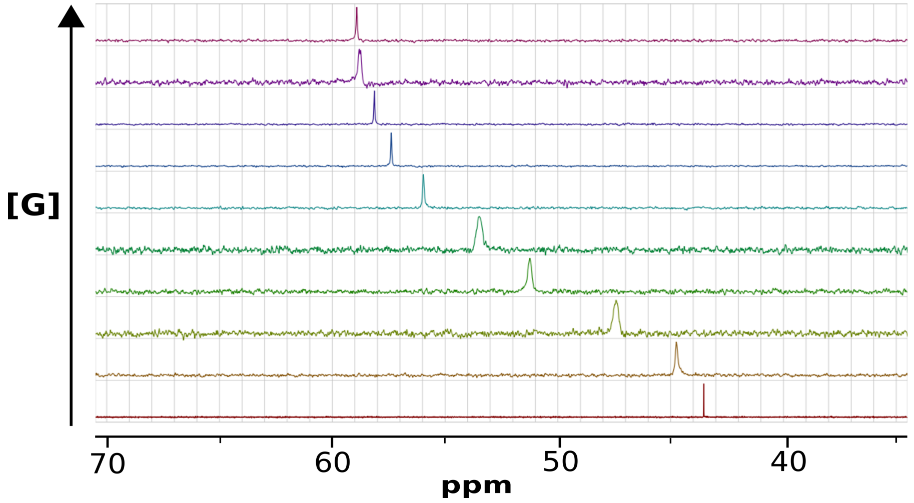

Graph S180. ^{31}P-NMR spectra of titration of $AgBF_4$ with phosphine oxide in acetonitrile.

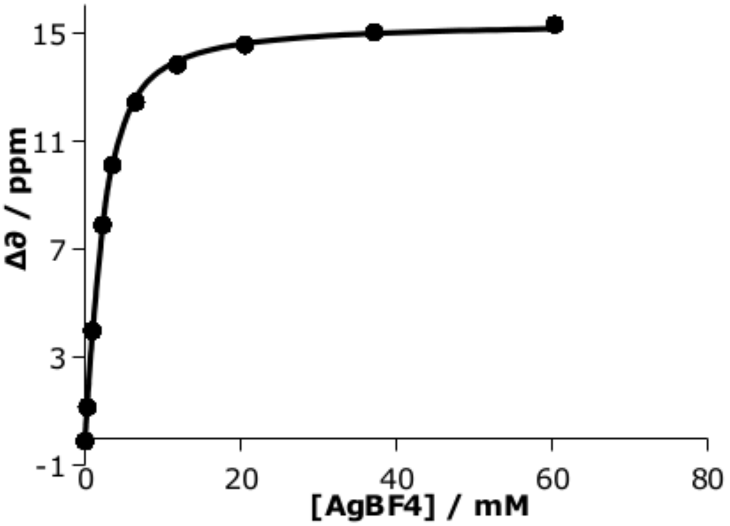

Graph S181. Binding isotherms for titration using 1:1 fitting program for titration of $AgBF_4$ against phosphine oxide in acetonitrile. [phosphine oxide] = 4.9mM and [$AgBPh_4$] = 80 mM, Δppm = 15.4.

K_a = 966 ± 40 M^{-1} 98 % bound

h) Titration of phosphine oxide with GuanidiniumBPh4 in acetone

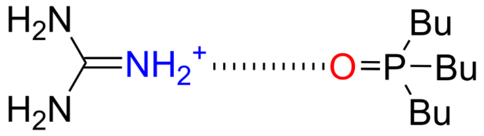

Host: phosphine oxide = 4.9 mM
Guest: GuanidiniumBPh4 = 50 mM

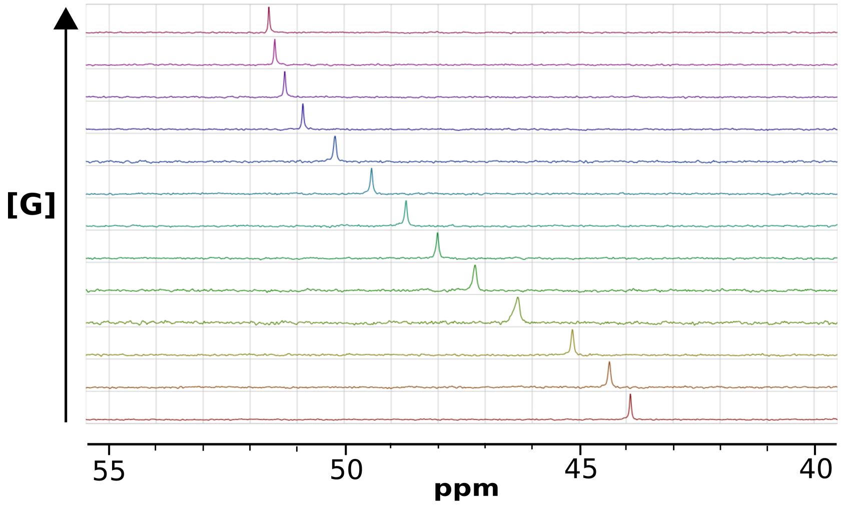

Graph S182. ^{31}P-NMR spectra of titration of GuanidiniumBPh4 with phosphine oxide in acetone.

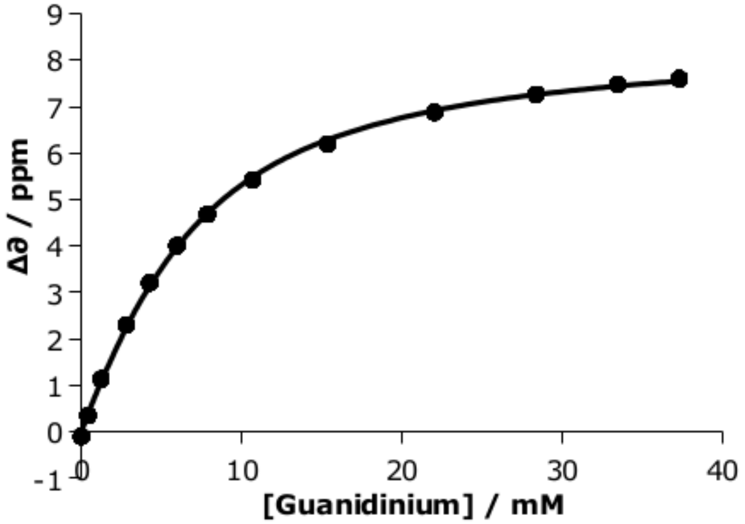

Graph S183. Binding isotherms for titration using 1:1 fitting program for titration of GuanidiniumBPh4 against phosphine oxide in acetone. [phosphine oxide] =4.9 mM and [GuanidiniumBPh4] = 50 mM, Δppm = 7.66 .

K_a = 225 ± 40 M^{-1} 88 % bound

i) Titration of phosphine oxide with Et_3NHBPh_4 in acetone

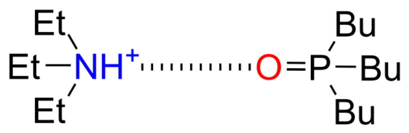

Host: phosphine oxide = 4.8 mM
Guest: Et_3NHBPh_4 = 40 mM

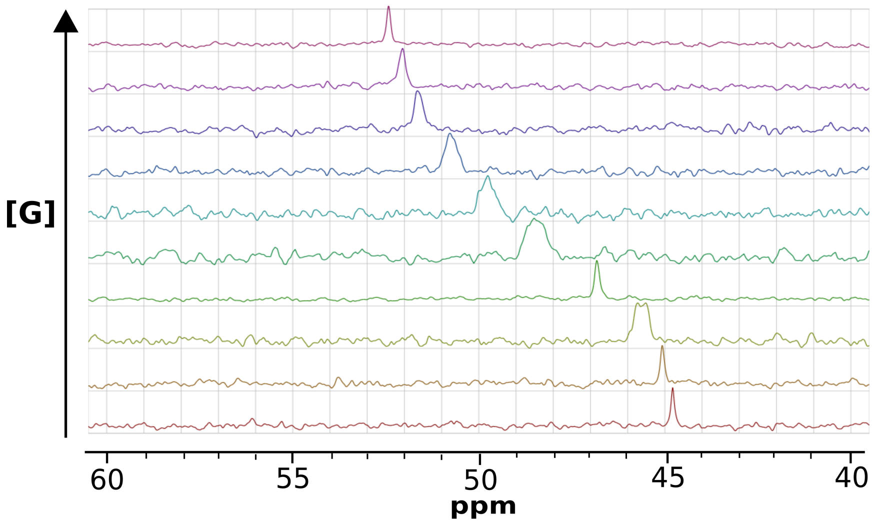

Graph S184. ^{31}P-NMR spectra of titration of Et_3NHBPh_4 with phosphine oxide in acetone.

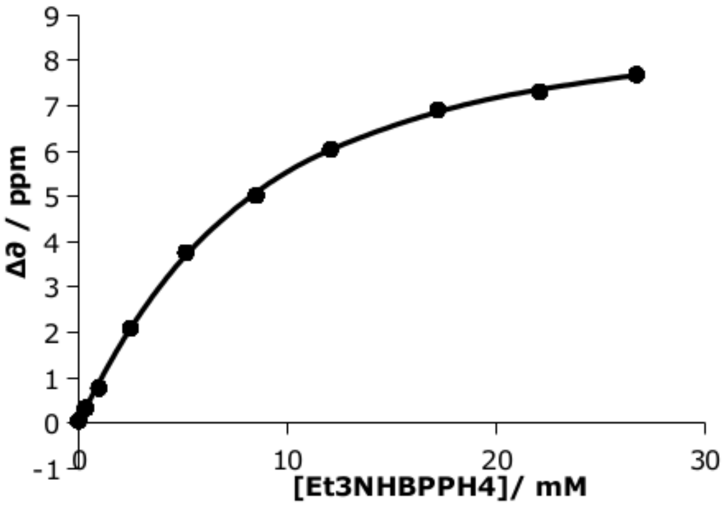

Graph S185. Binding isotherms for titration using 1:1 fitting program for titration of Et_3NHBPh_4 against phosphine oxide in acetone. [phosphine oxide] = 4.8 mM and [Et_3NHBPh_4] = 40 mM, Δppm = 7.41.

K_a = 203 ± 50 M^{-1} 80 % bound

j) Titration of phosphine oxide with 1-MethylimidazoliumBPh$_4$ in acetone

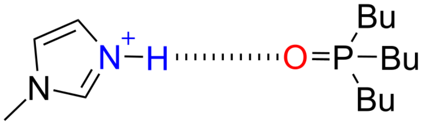

Host: phosphine oxide = 5.2 mM
Guest: 1-MethylimidazoliumBPh$_4$ = 40 mM

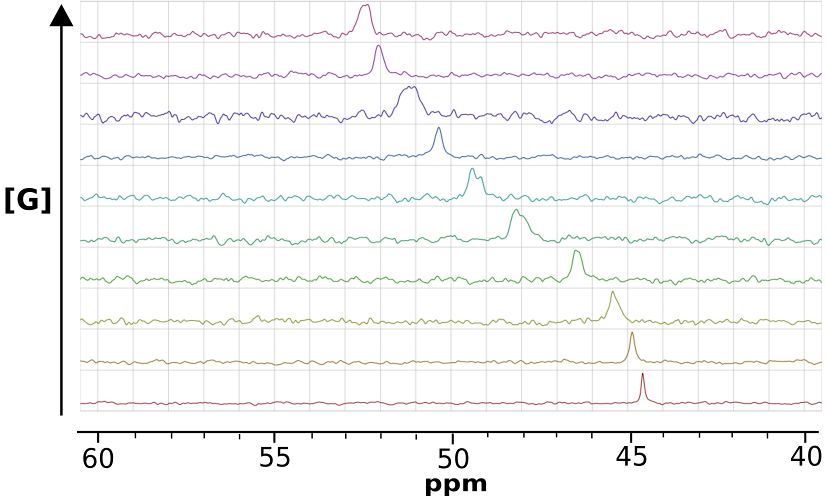

Graph S186. ^{31}P-NMR spectra of titration of 1-MethylimidazoliumBPh$_4$ with phosphine oxide in acetone.

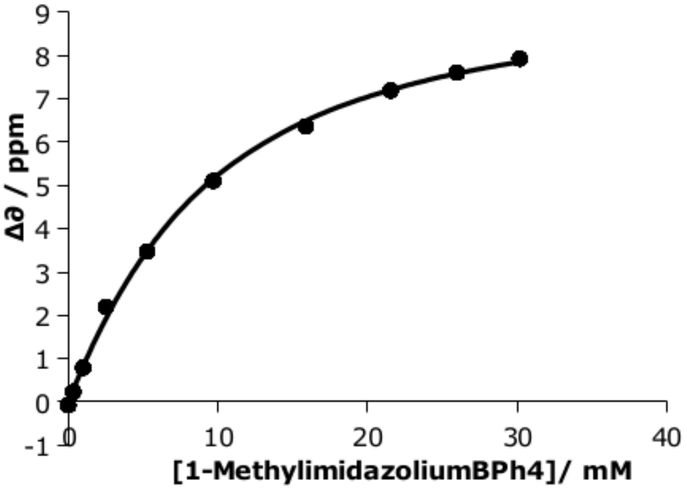

Graph S187. Binding isotherms for titration using 1:1 fitting program for titration of 1-MethylimidazoliumBPh$_4$ against phosphine oxide in acetone. [phosphine oxide] = 5.2 mM and [1-MethylimidazoliumBPh$_4$] = 40 mM, Δppm = 7.96.

K_a = 156 ± 18 M^{-1} 80 % bound

j) Titration of phosphine oxide with 2-EthylhexylammoniumBPh$_4$ in acetone

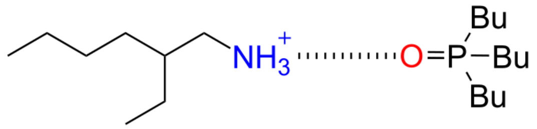

Host: phosphine oxide = 5.2 mM
Guest: 2-EthylhexylammoniumBPh$_4$ = 49 mM

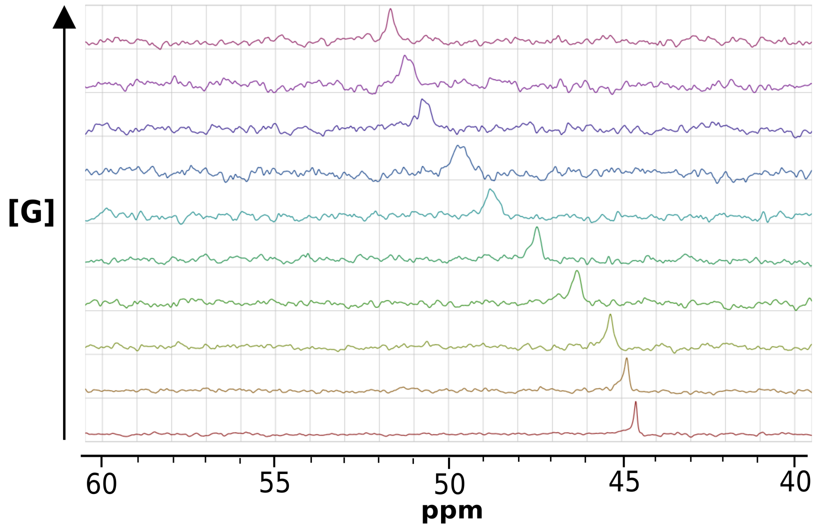

Graph S188. ^{31}P-NMR spectra of titration of 2-EthylhexylammoniumBPh$_4$ with phosphine oxide in acetone.

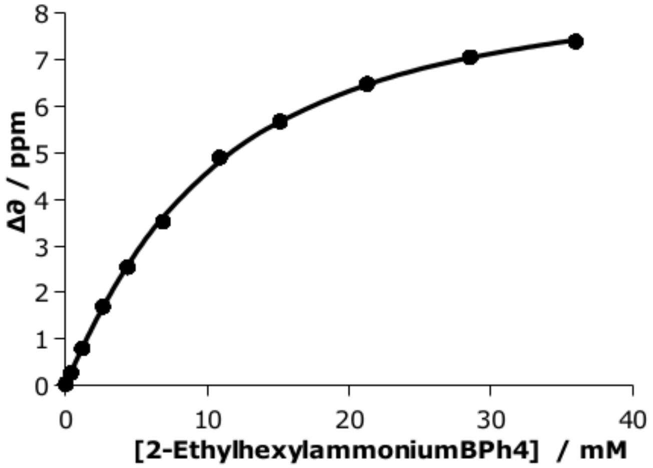

Graph S189. Binding isotherms for titration using 1:1 fitting program for titration of 2-EthylhexylammoniumBPh$_4$ against phosphine oxide in acetone. [phosphine oxide] = 5.2 mM and [2-EthylhexylammoniumBPh$_4$] = 4.9 mM, Δppm = 7.09.

K_a = 123 ± 34 M^{-1} 80 % bound

k) Titration of phosphine oxide with IPh_2BPF_6 in acetone

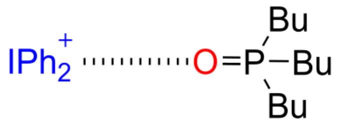

Host: phosphine oxide = 5.1 mM
Guest: IPh_2PF_6 = 49 mM

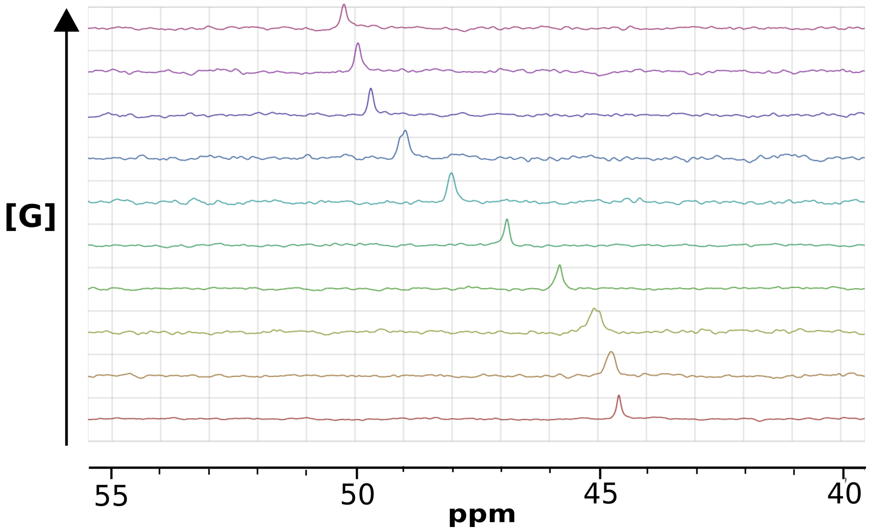

Graph S190. ^{31}P-NMR spectra of titration of IPh_2PF_6 with phosphine oxide in acetone.

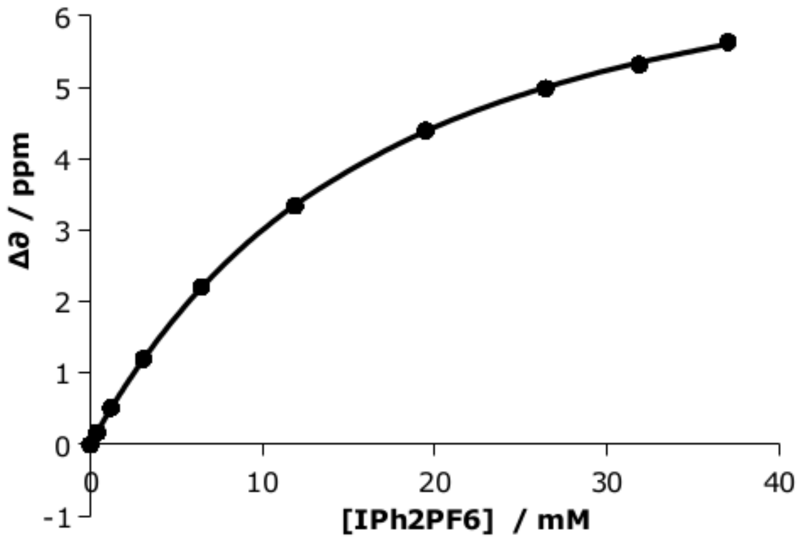

Graph S191. Binding isotherms for titration using 1:1 fitting program for titration of IPh_2BF_4 against phosphine oxide in acetone. [phosphine oxide] = 5.1 mM and [IPh_2PF_6] = 49 mM, Δppm = 5.64.

K_a = 83 ± 12 M^{-1} 72 % bound

l) Titration of phosphine oxide with TriphenylmethylPF$_6$ in acetone

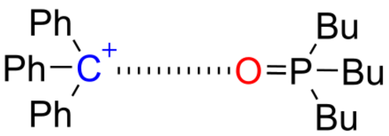

Host: phosphine oxide = 5.5 mM
Guest: TriphenylmethylPF$_6$ = 50 mM

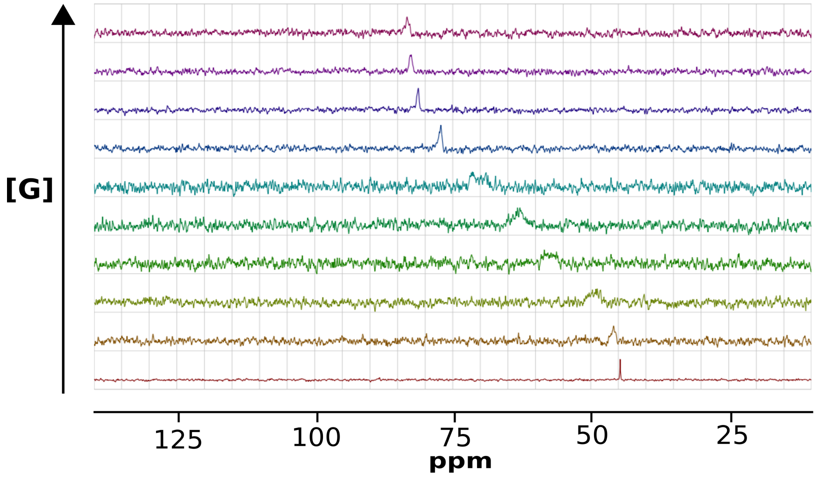

Graph S192. ^{31}P-NMR spectra of titration of TriphenylmethylPF$_6$ with phosphine oxide in acetone.

m) Titration of phosphine oxide with $NaBF_4$ in acetone

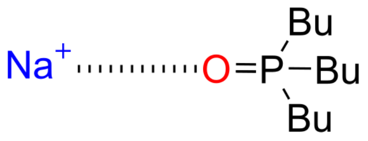

Host: phosphine oxide = 5.5 mM
Guest: $NaBF_4$ = 58 mM

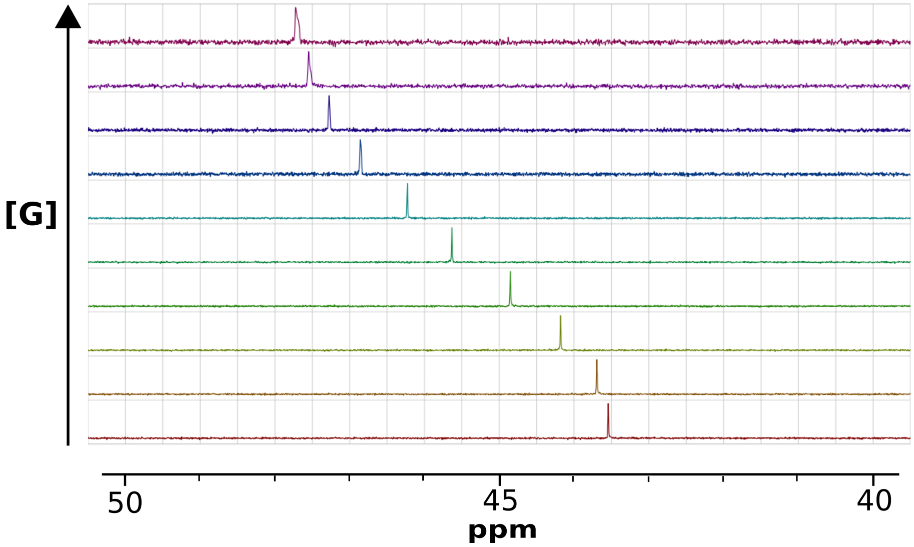

Graph S193. P^{31}-NMR spectra of titration of $NaBF_4$ with phosphine oxide in acetone.

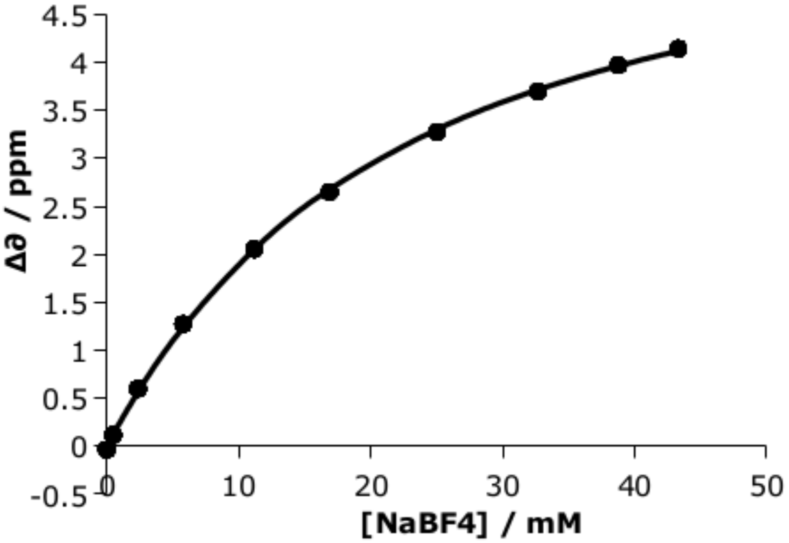

Graph S194. Binding isotherms for titration using 1:1 fitting program for titration of $NaBF_4$ against phosphine oxide in acetone. [phosphine oxide] = 5.5 mM and [$NaBF_4$] = 58 mM, Δppm = 4.15.

K_a = 57 ± 2 M^{-1} 69 % bound

n)Titration of phosphine oxide with NaBArF in acetone

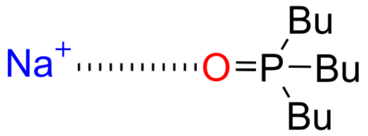

Host: phosphine oxide = 4.5 mM
Guest: NaBArF = 50 mM

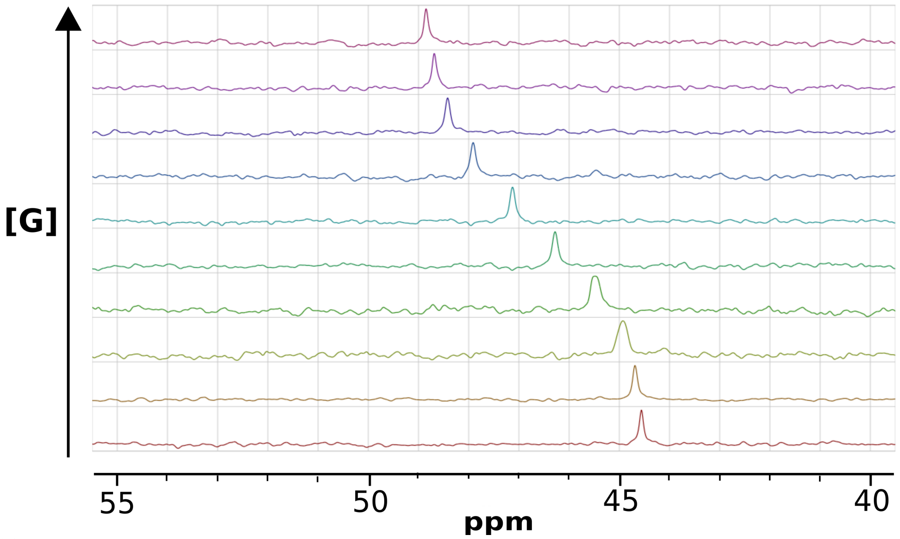

Graph S195. ^{31}P-NMR spectra of titration of NaBArF with phosphine oxide in acetone.

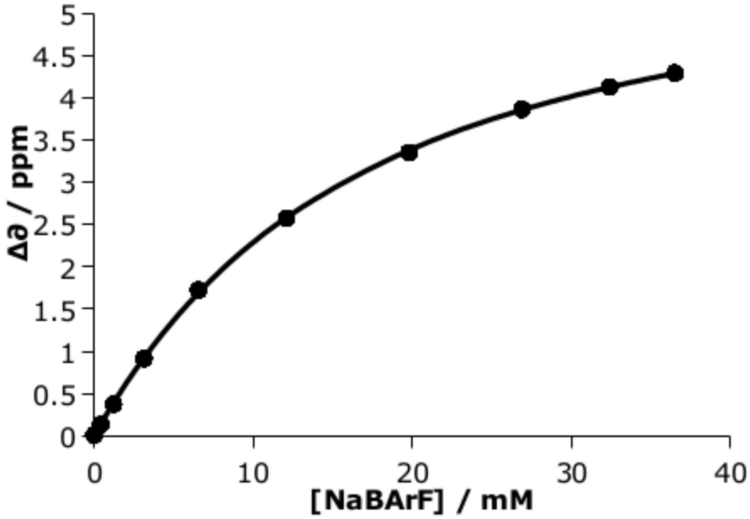

Graph S196. Binding isotherms for titration using 1:1 fitting program for titration of NaBArF against phosphine oxide in acetone. [phosphine oxide] = 4.5 mM and [NaBArF] = 50 mM, Δppm = 4.12 .

K_a = 72 ± 2 M^{-1} 71 % bound

o) Titration of phosphine oxide with $NaN(SO_2CF_3)_2$ in acetone

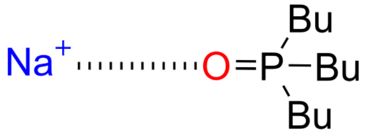

Host: phosphine oxide = 5.0 mM
Guest: $NaN(SO_2CF_3)_2$ = 51 mM

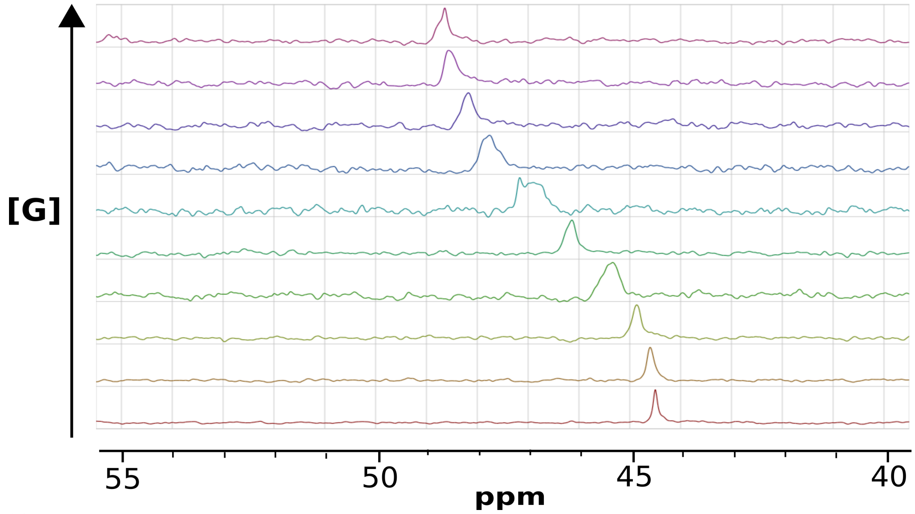

Graph S197. ^{31}P-NMR spectra of titration of $NaN(SO_2CF_3)_2$ with phosphine oxide in acetone.

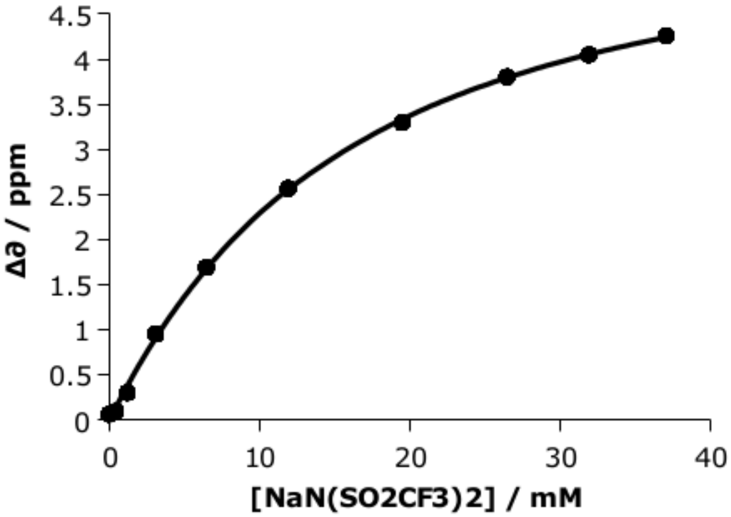

Graph S198. Binding isotherms for titration using 1:1 fitting program for titration of $NaN(SO_2CF_3)_2$ against phosphine oxide in acetone. [phosphine oxide] = 5.0 mM and [$NaN(SO_2CF_3)_2$] = 51 mM, Δppm = 4.14.

K_a = 78 ± 12 M^{-1} 71 % bound

p) Titration of phosphine oxide with NaI in acetone

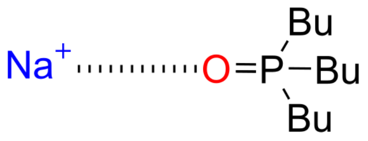

Host: phosphine oxide = 4.9 mM
Guest: NaI = 61 mM

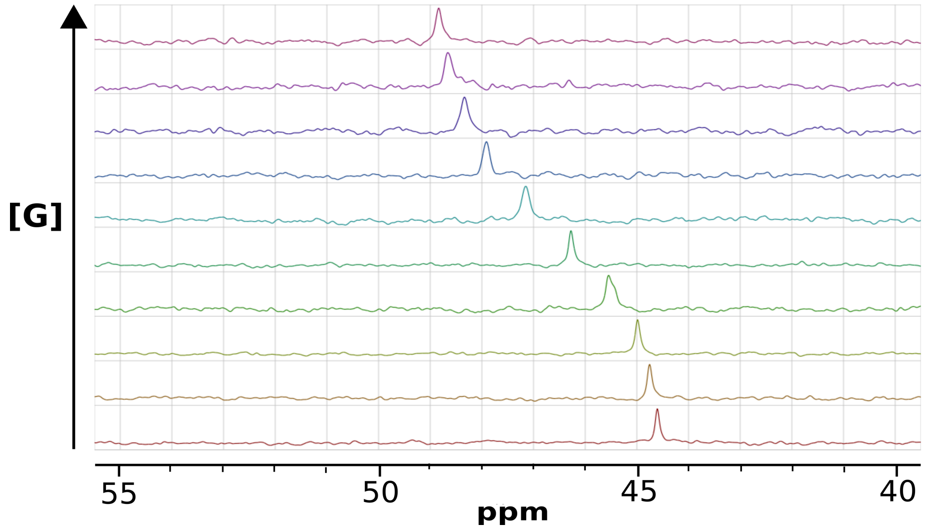

Graph S199. ^{31}P-NMR spectra of titration of NaI with phosphine oxide in acetone.

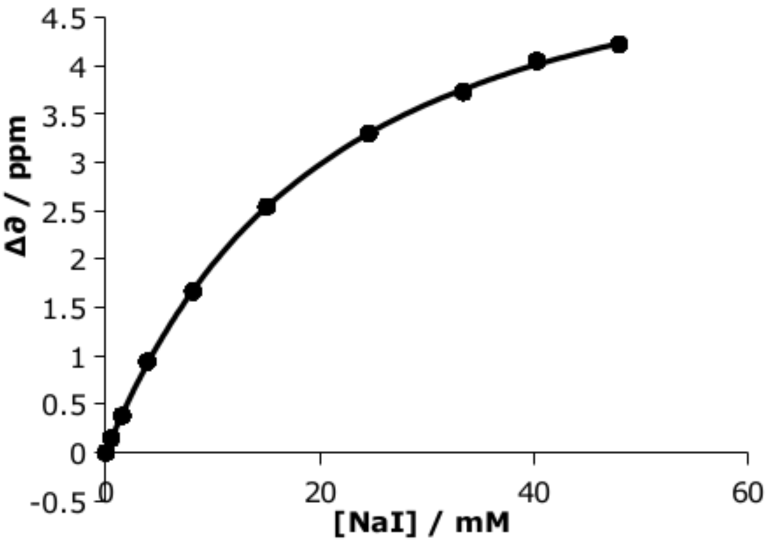

Graph S200. Binding isotherms for titration using 1:1 fitting program for titration of NaI against phosphine oxide in acetone. [phosphine oxide] = 4.9 mM and [NaI] = 61 mM, Δppm = 4.23.

K_a = 59 ± 6 M^{-1}   **70 % bound**

q) Titration of phosphine oxide with $NaPF_6$ in acetone

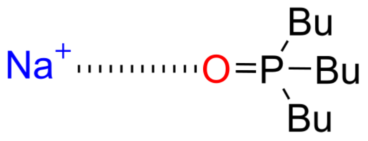

Host: phosphine oxide = 5.5 mM
Guest: $NaPF_6$ = 57 mM

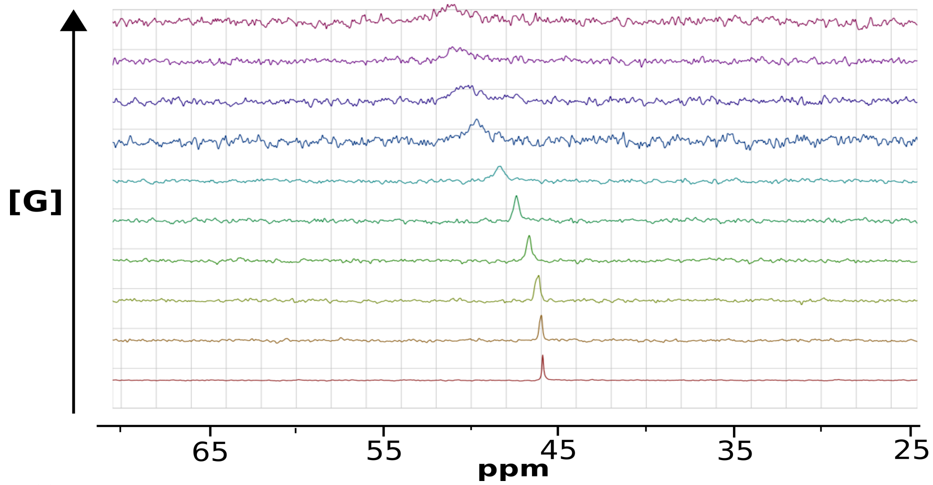

Graph S201. ^{31}P-NMR spectra of titration of $NaPF_6$ with phosphine oxide in acetone.

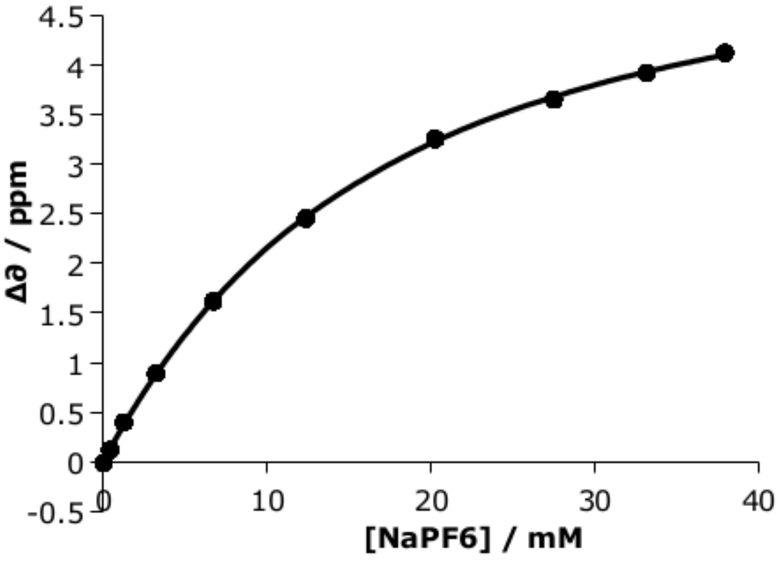

Graph S202. Binding isotherms for titration using 1:1 fitting program for titration of $NaPF_6$ against phosphine oxide in acetone. [phosphine oxide] = 5.5 mM and [$NaPF_6$] = 57 mM, Δppm = 4.09.

$K_a = 55 \pm 13\ M^{-1}$ 68 % bound

r) Titration of phosphine oxide with $KBPh_4$ in acetone

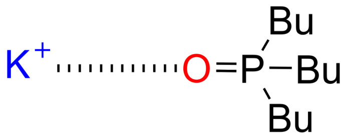

Host: phosphine oxide = 5.2 mM
Guest: $KBPh_4$ = 110 mM

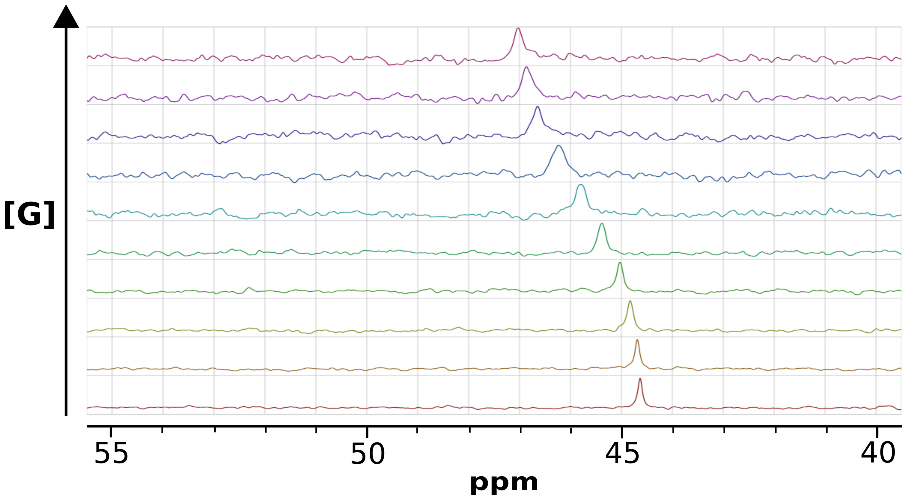

Graph S203. ^{31}P-NMR spectra of titration of $KBPh_4$ with phosphine oxide in acetone.

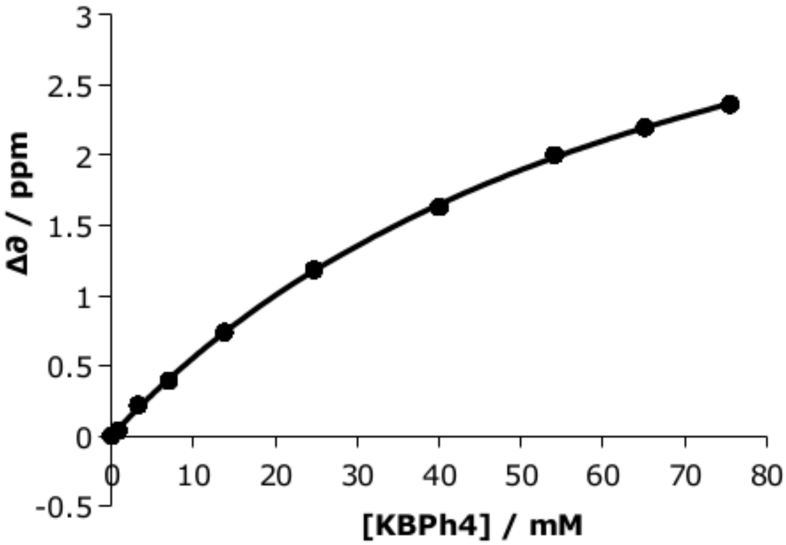

Graph S204. Binding isotherms for titration using 1:1 fitting program for titration of $KBPh_4$ against phosphine oxide in acetone. [phosphine oxide] = 5.2 mM and [$KBPh_4$] = 110 mM, Δppm = 2.31 .

$K_a = 13 \pm 3$ M^{-1} 51 % bound

Titration Curves for Phosphine Oxide in chloroform.

a) Titration of phosphine oxide with phenol in chloroform

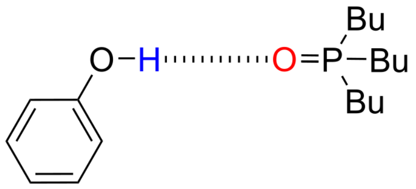

Host: phosphine oxide = 1.9 mM
Guest: phenol = 54 mM

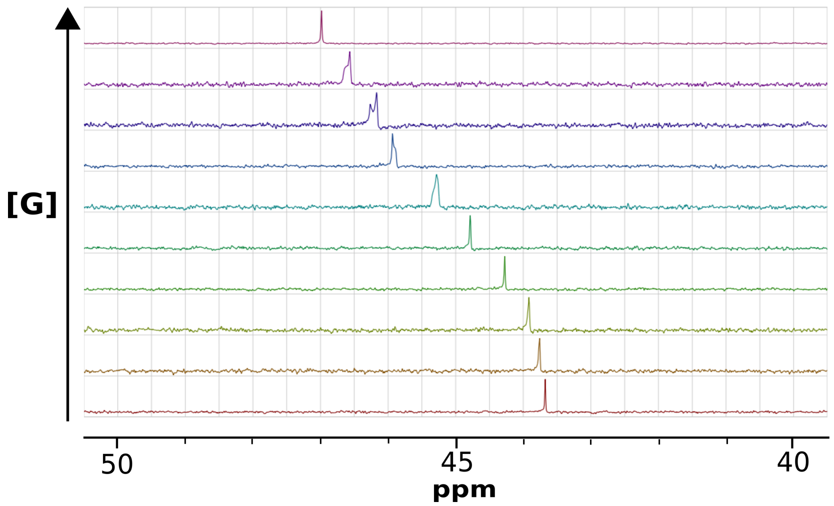

Graph S205. ^{31}P-NMR spectra of titration of phenol with phosphine oxide in chloroform.

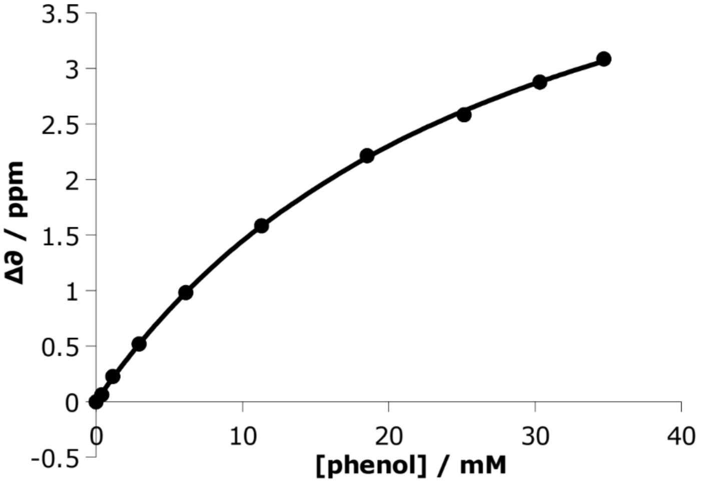

Graph S206. Binding isotherms for titration using 1:1 fitting program for titration of phenol against phosphine oxide in chloroform. [phosphine oxide] = 1.9 mM and [Phenol] = 54 mM, Δppm = 3.31.

K_a = 38 ± 2 M^{-1} 60 % bound

b) Titration of phosphine oxide with 4-phenylazophenol in chloroform

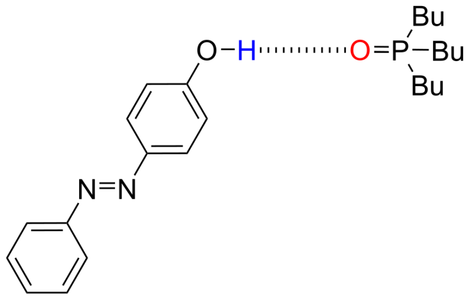

Host: phosphine oxide = 0.52 mM
Guest: 4-phenylazophenol = 11 mM

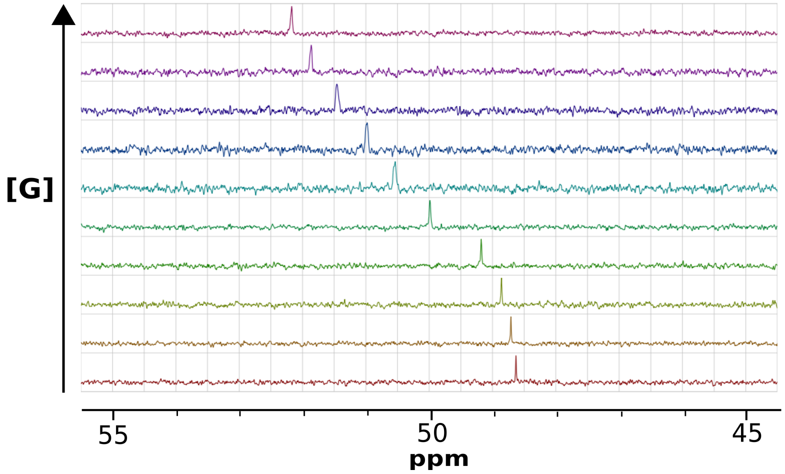

Graph S207. ^{31}P-NMR spectra of titration of 4-phenylazophenol with phosphine oxide in chloroform.

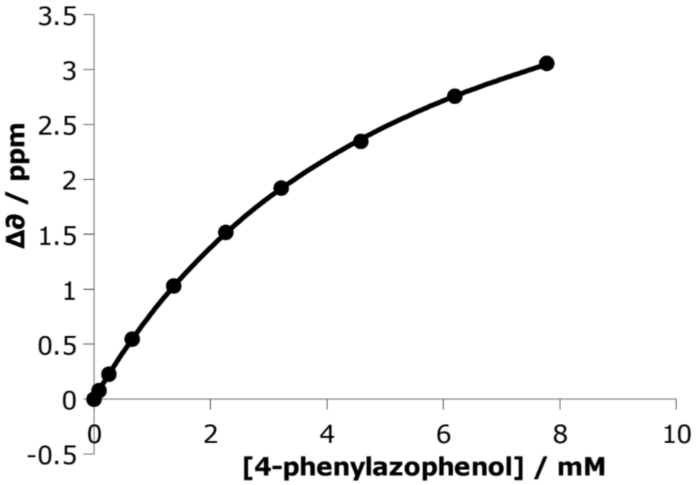

Graph S208. Binding isotherms for titration using 1:1 fitting program for titration of 4-phenylazophenol against phosphine oxide in chloroform. [phosphine oxide] = 0.52 mM and [4-phenylazophenol] = 11 mM, Δppm = 3.06.

K_a = 193 ± 27 M^{-1} 60 % bound

c) Titration of phosphine oxide with 4-nitrophenol in chloroform

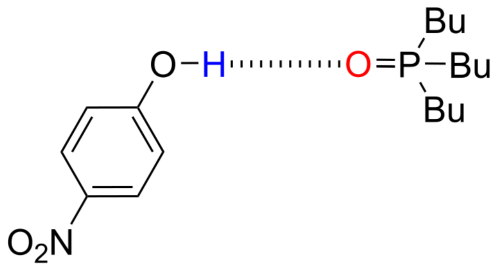

Host: phosphine oxide = 0.46 mM
Guest: 4-nitrophenol = 4.8 mM

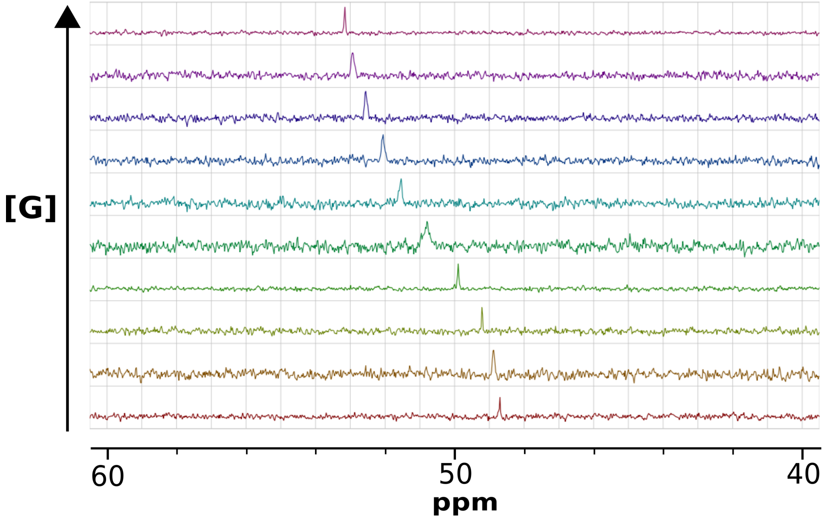

Graph S209. ^{31}P-NMR spectra of titration of 4-nitrophenol with phosphine oxide in chloroform.

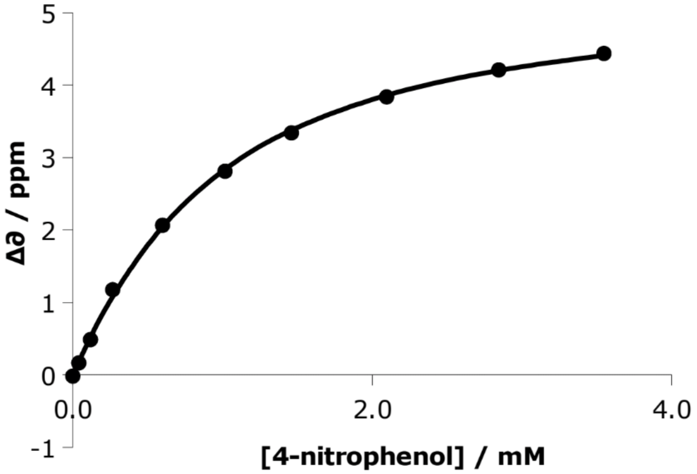

Graph S210. Binding isotherms for titration using 1:1 fitting program for titration of 4-nitrophenol against phosphine oxide in chloroform. [phosphine oxide] = 0.46 mM and [4-nitrophenol] = 4.8 mM, Δppm = 4.25.

K_a = 1416 ± 208 M^{-1} **82 % bound**

d) Titration of phosphine oxide with 4-nitro,3-trifluoromethylphenol in chloroform

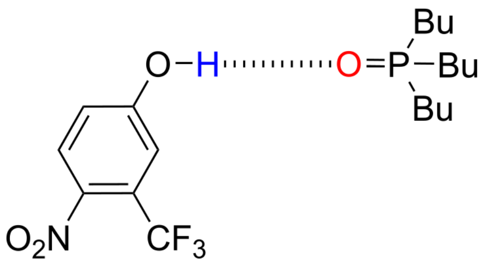

Host: phosphine oxide = 0.58 mM
Guest: 4-nitro,3-trifluoromethylphenol = 14 mM

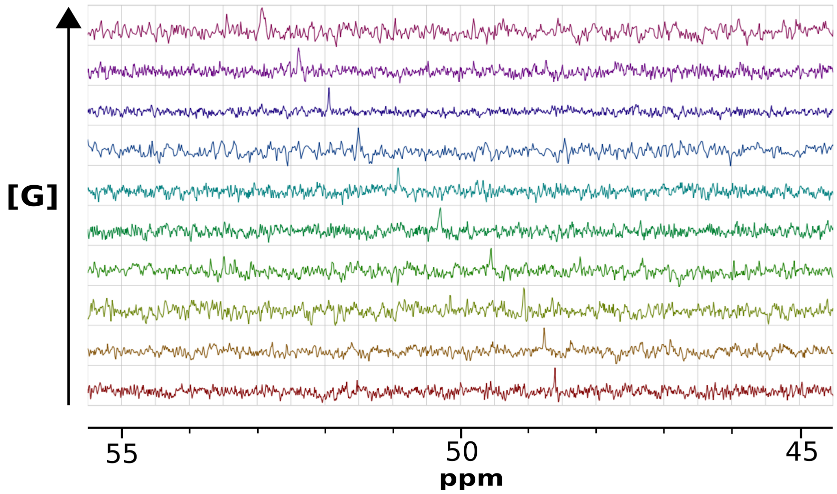

Graph S211. ^{31}P-NMR spectra of titration of 4-nitro,3-trifluoromethylphenol with phosphine oxide in chloroform.

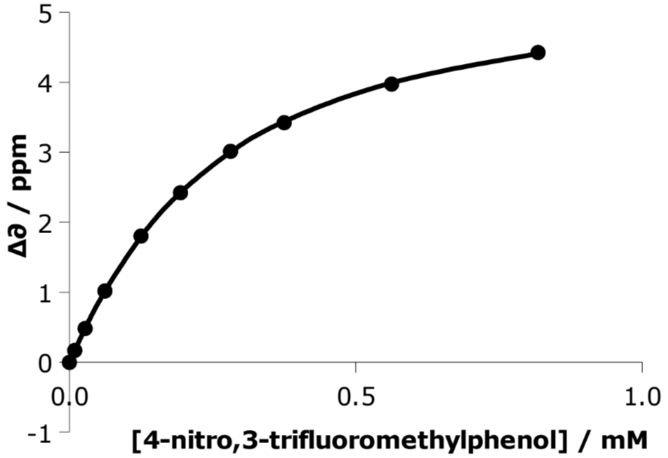

Graph S212. Binding isotherms for titration using 1:1 fitting program for titration of 4-nitro,3-trifluoromethylphenol against phosphine oxide in chloroform. [phosphine oxide] = 0.58 mM and [4-nitro,3-trifluoromethylphenol] = 14 mM, Δppm = 4.31.

K_a = 5370 ± 294 M^{-1} 66 % bound

Titration Curves for Phosphine Oxide in Carbon Tetrachloride.

a) Titration of phosphine oxide with phenol in carbon tetrachloride

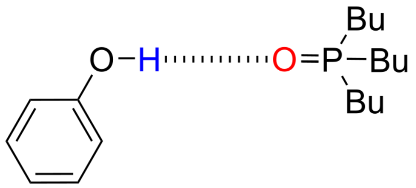

Host: phenol = 0.49 mM
Guest: phosphine oxide = 4.3 mM

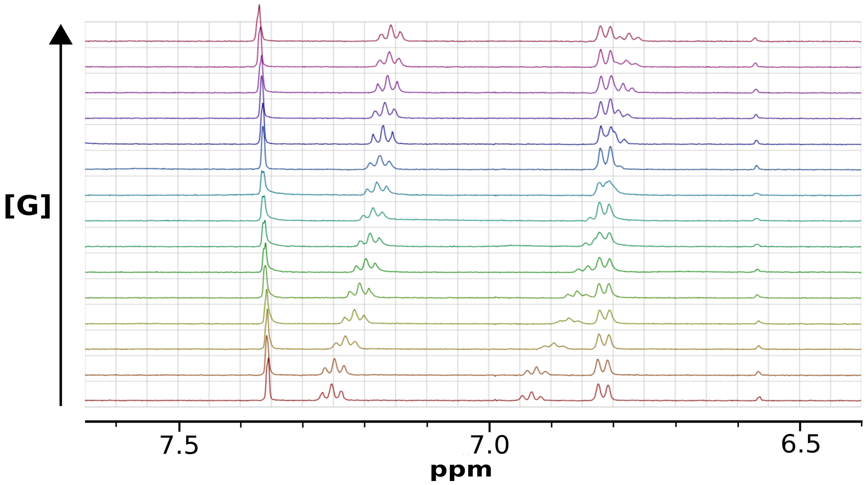

Graph S213. ^{1}H-NMR spectra of titration of phenol with phosphine oxide in carbon tetrachloride.

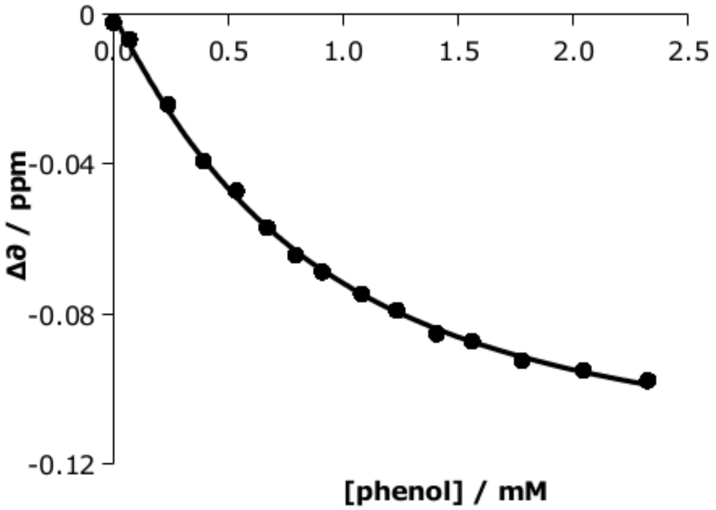

Graph S214. Binding isotherms for titration using 1:1 fitting program for titration of phenol against phosphine oxide in carbon tetrachloride. [phosphine oxide] = 4.3 mM and [Phenol] = 0.49 mM, Δppm = 0.095.

K_a = 1860 ± 226 M^{-1} 78% bound

b) Titration of phosphine oxide with 5-nitroindole in carbon tetrachloride

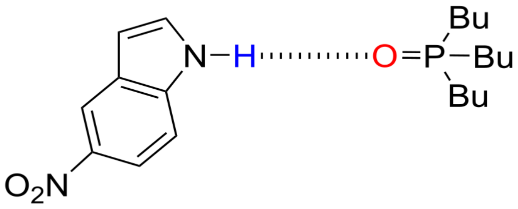

Host: 5-nitroindole = 1.5 mM
Guest: phosphine oxide = 11 Mm

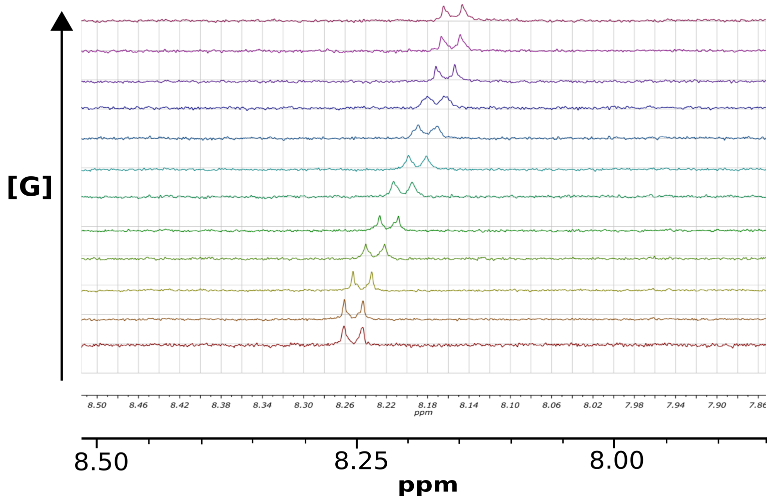

Graph S215. ^{31}P-NMR spectra of titration of 5-nitroindole with phosphine oxide in carbon tetrachloride.

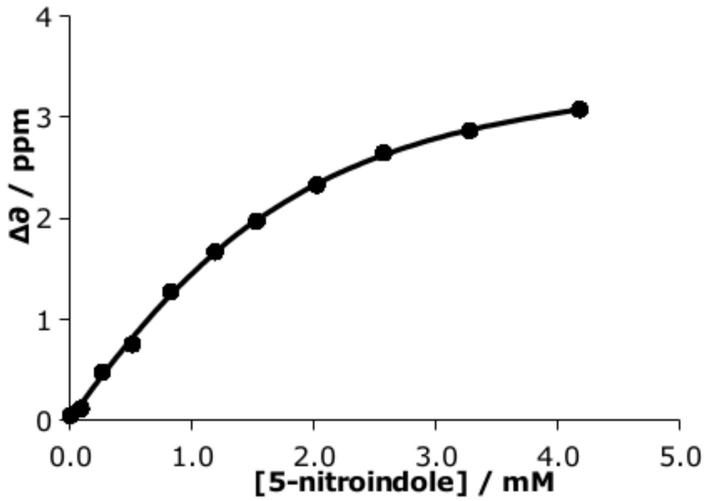

Graph S216. Binding isotherms for titration using 1:1 fitting program for titration of 5-nitroindole against phosphine oxide in carbon tetrachloride . [phosphine oxide] = 11 mM and [5-nitroindole] = 1.5 mM, Δppm = 4.53.

K_a = 1350 ± 480 M^{-1}   **82% bound**

c) Titration of phosphine oxide with 4-phenylazophenol in carbon tetrachloride

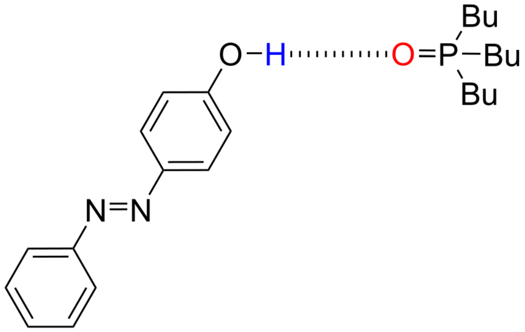

Host: 4-phenylazophenol = 0.19 mM
Guest: phosphine oxide = 0.84 mM

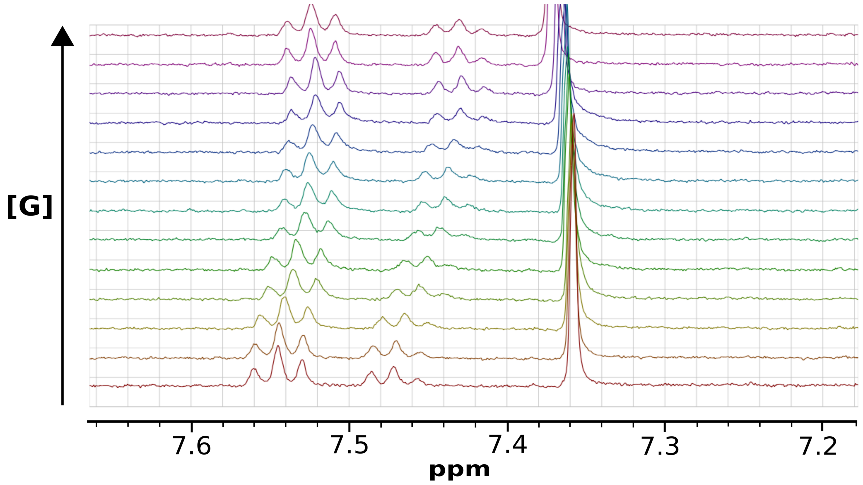

Graph S217. ^{1}H-NMR spectra of titration of 4-phenylazophenol with phosphine oxide in carbon tetrachloride.

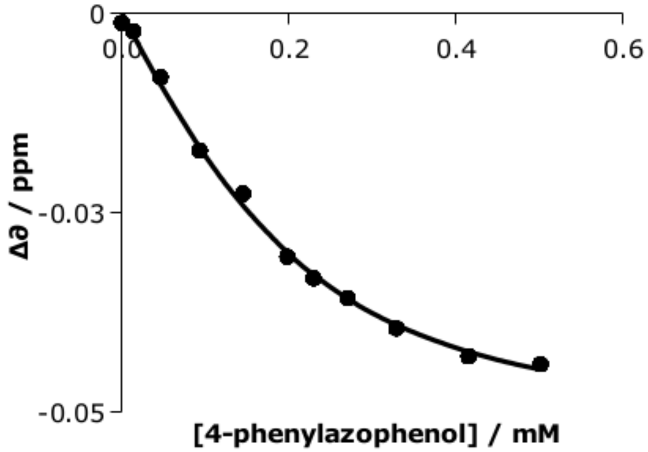

Graph S218. Binding isotherms for titration using 1:1 fitting program for titration of Phenol against phosphine oxide in carbon tetrachloride. [phosphine oxide] = 0.84 mM and [4-phenylazophenol] 0.19 = mM, Δppm = -0.043 .

K_a = 14350 ± 2404 M^{-1} 82 % bound

d) Titration of phosphine oxide with 4-nitrophenol in carbon tetrachloride

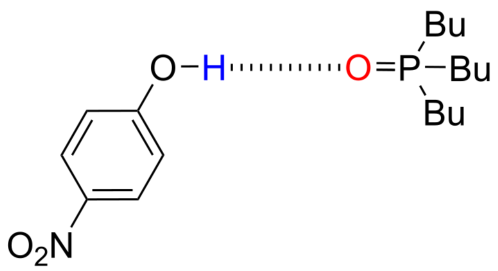

Host: 4-nitrophenol = 0.10 mM
Guest: phosphine oxide = 0.47 mM

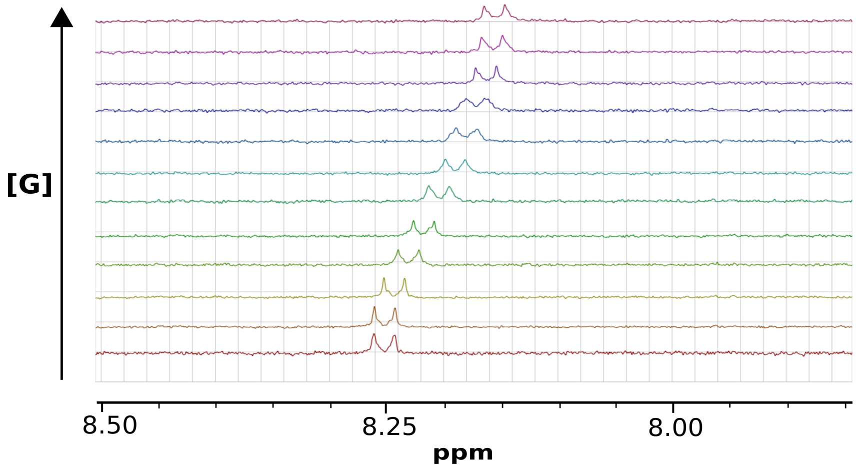

Graph S219. ^{1}H-NMR spectra of titration of 4-nitrophenol with phosphine oxide in carbon tetrachloride.

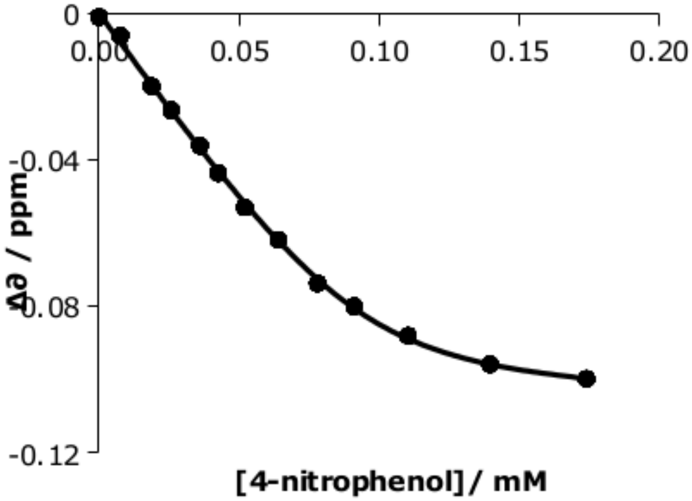

Graph S220. Binding isotherms for titration using 1:1 fitting program for titration of Phenol against phosphine oxide in carbon tetrachloride. [phosphine oxide] =0.47 mM and [4-nitrophenol] = 0.10 mM, Δppm = -2.8082.

K_a =142000 ± 26870 M^{-1} 93% bound

21. Summary of UV/vis Spectroscopy Titration Experiments.

Hydrogen Bond Donor	Acceptor	Solvent	*K* (M^{-1})[a]	*% bound*	ε_{bound} (M^{-1} cm^{-1})
4-phenylazophenol	Reichardt's dye	Acetonitrile	3100 ± 650	63	1269
4-phenylazophenol	Bu_3PO	Acetonitrile	69 ± 9	77	3828
4-phenylazophenol	Reichardt's dye	Chloroform	4000 ± 700	65	3642
4-phenylazophenol	Bu_3PO	Chloroform	240 ± 45	80	3714
4-phenylazophenol	Reichardt's dye	Acetone	2000 ± 500	58	2252
4-phenylazophenol	Phosphine oxide	Acetone	48 ± 16	57	2492
4-phenylazophenol	Phosphine oxide	Carbon tetrachloride	9200 ± 1900	90	7310
phenol	Reichardt's dye	Acetonitrile	260 ± 63	61	2225
phenol	Reichardt's dye	Chloroform	260 ± 55	59	2590
phenol	Bu_3PO	Chloroform	55 ± 7	64	2642
phenol	Reichardt's dye	Acetone	340 ± 6	64	1920
5-nitroindole	Reichardt's dye	Acetonitrile	200 ± 50	58	264
5-nitroindole	Reichardt's dye	Chloroform	1200 ± 500	61	2486
5-nitroindole	Bu_3PO	Chloroform	18 ± 6	68	1189
5-nitroindole	Reichardt's dye	Acetone	860 ± 350	79	353
5-nitroindole	Bu_3PO	Carbon tetrachloride	1200 ± 400	68	4125
4-nitro,3-trifluoromethylphenol	Bu_3PO	Acetonitrile	300 ± 98	89	1591
4-nitro,3-trifluoromethylphenol	Bu_3PO	Chloroform	5400 ± 1700	75	3133
4-nitrophenol	Bu_3PO	Acetonitrile	110 ± 36	62	3024
4-nitrophenol	Bu_3PO	Chloroform	1400 ± 110	75	6403
4-nitrophenol	Bu_3PO	Carbon tetrachloride	98600 ± 19000	83	5267.
$NaBPh_4$	Reichardt's dye	Acetonitrile	390 ± 51	63	1505
$NaBPh_4$	Reichardt's dye	Acetone	1700 ± 160	74	1327
$NaPF_6$	Reichardt's dye	Acetone	1700 ± 140	53	1773
$NaBF_4$	Reichardt's dye	Acetonitrile	300 ± 90	70	2223
$NaBF_4$	Reichardt's dye	Acetone	1900 ± 100	77	613
$NaN(SO_2CF_3)_2$	Reichardt's dye	Acetonitrile	410 ± 30	71	551
$NaN(SO_2CF_3)_2$	Reichardt's dye	Acetone	1500 ± 200	68	1207
$NaBAr^F$	Reichardt's dye	Acetonitrile	400 ± 160	78	625
$NaBAr^F$	Reichardt's dye	Acetone	1400 ± 400	71	1433
NaI	Reichardt's dye	Acetonitrile	320 ± 120	60	470
NaI	Reichardt's dye	Acetone	1000 ± 300	70	1323
$KBPh_4$	Reichardt's dye	Acetone	270 ± 75	51	1053
$CsBPh_4$	Reichardt's dye	Acetone	210 ± 35	60	289
$RbBPh_4$	Reichardt's dye	Acetone	220 ± 50	56	840
$TBABF_4$	Reichardt's dye	Acetone	10 ± 2	55	1093
$LiBPH_4$ tris(1,2-dimethoxyethane)	Reichardt's dye	Acetonitrile	27400 ± 5900	70	1800
GuanidiniumBPh_4	Reichardt's dye	Acetonitrile	33800 ± 2000	79	2287
IPh_2BF_4	Reichardt's dye	Acetonitrile	38300 ± 1100	87	1746
IPh_2BF_4	Reichardt's dye	Acetone	48000 ± 3000	79	6867
4-methyl-1-butylpyridniumPF_6	Reichardt's dye	Acetonitrile	12 ± 2	59	506
4-methyl-1-butylpyridniumPF_6	Reichardt's dye	Acetone	180 ± 40	84	1207
1-methyl,3-octylimidazolium	Reichardt's dye	Acetonitrile	23 ± 9	51	731
1-methyl,3-octylimidazolium	Reichardt's dye	Acetone	200 ± 60	67	653
4-phenylazophenol	Reichardt's dye	Acetonitrile + 0.1% H_2O	2700 ± 560	60	1533
4-phenylazophenol	Reichardt's dye	Acetonitrile + 0.25% H_2O	1600 ± 380	50	792
4-phenylazophenol	Reichardt's dye	Acetonitrile + 0.5% H_2O	1100 ± 180	51	2183
4-phenylazophenol	Reichardt's dye	Acetonitrile + 1% H_2O	800 ± 20	65	1438

$NaN(SO_2CF_3)_2$	Reichardt's dye	Acetonitrile + 0.1% H_2O	380 ± 38	75	1195
$NaN(SO_2CF_3)_2$	Reichardt's dye	Acetonitrile + 0.25% H_2O	290 ± 18	75	683
$NaN(SO_2CF_3)_2$	Reichardt's dye	Acetonitrile + 0.5% H_2O	250 ± 60	71	783
GuanidiniumBPh$_4$	Reichardt's dye	Acetonitrile + 0.1% H_2O	34000 ± 3800	91	1952
GuanidiniumBPh$_4$	Reichardt's dye	Acetonitrile + 0.25% H_2O	27000 ± 2000	90	1990
GuanidiniumBPh$_4$	Reichardt's dye	Acetonitrile + 0.5% H_2O	12600 ± 4200	88	1488
GuanidiniumBPh$_4$	Reichardt's dye	Acetonitrile + 1% H_2O	11000 ± 2000	89	469
4-phenylazophenol	Reichardt's dye	Acetone + 0.1% H_2O	1800 ± 280	67	1963
4-phenylazophenol	Reichardt's dye	Acetone + 0.25% H_2O	1300 ± 71	50	2887
4-phenylazophenol	Reichardt's dye	Acetone + 0.5% H_2O	860 ± 240	50	613
4-phenylazophenol	Reichardt's dye	Acetone + 1% H_2O	600 ± 200	51	247
$NaN(SO_2CF_3)_2$	Reichardt's dye	Acetone + 0.1% H_2O	1500 ± 500	71	1238
$NaN(SO_2CF_3)_2$	Reichardt's dye	Acetone + 0.25% H_2O	1100 ± 200	63	2227
$NaN(SO_2CF_3)_2$	Reichardt's dye	Acetone + 0.5% H_2O	840 ± 280	58	940
$NaN(SO_2CF_3)_2$	Reichardt's dye	Acetone + 1% H_2O	550 ± 120	55	1104
$KBPh_4$	Reichardt's dye	Acetone + 0.1% H_2O	260 ± 21	72	1167
$KBPh_4$	Reichardt's dye	Acetone + 0.25% H_2O	190 ± 15	68	867
$KBPh_4$	Reichardt's dye	Acetone + 0.5% H_2O	170 ± 18	56	173
$KBPh_4$	Reichardt's dye	Acetonitrile + 1% H_2O	132 ± 70	54	912

Table S1. Association constants for the H-bonded complexes formed between **1** and **3-27** in the different solvents at 298 K. [a]Experimental results are the average of at least two titrations and errors are quoted at the 95% confidence limit.

22. Summary of ^{31}P NMR Spectroscopy Titration Experiments.

Hydrogen Bond Donor	Acceptor	Solvent	K (M^{-1})^{a}	*% bound*	δ_{bound}
Phenol	Phosphine oxide	Chloroform	38 ± 2	60	54.104
Phenol	Phosphine oxide	Acetone	26 ± 1	64	52.273
Phenol	Phosphine oxide	Acetonitrile	20 ± 1	78	53.096
5-nitroindole	Phosphine oxide	Acetone	11 ± 1	80	51.649
5-nitroindole	Phosphine oxide	Acetonitrile	6 ± 1	68	52.278
4-phenylazophenol	Phosphine oxide	Chloroform	193 ± 27	60	53.717
4-phenylazophenol	Phosphine oxide	Acetone	65 ± 6	76	53.007
4-phenylazophenol	Phosphine oxide	Acetonitrile	58 ± 4	74	53.590
4-nitrophenol	Phosphine oxide	Chloroform	1416 ± 208	82	54.076
4-nitrophenol	Phosphine oxide	Acetone	134 ± 18	89	54.286
4-nitrophenol	Phosphine oxide	Acetonitrile	102 ± 34	73	54.495
4-nitro,3-CF_3phenol	Phosphine oxide	Chloroform	5370 ± 294	66	54.166
4-nitro,3-CF_3phenol	Phosphine oxide	Acetone	282 ± 108	90	54.851
4-nitro,3-CF_3phenol	Phosphine oxide	Acetonitrile	266 ± 22	90	55.084
GuanidiniumBPh_4	Phosphine oxide	Acetone	225 ± 40	88	52.555
GuanidiniumBPh_4	Phosphine oxide	Acetonitrile	198 ± 61	85	53.477
RNH_3BPh_4	Phosphine oxide	Acetone	123 ± 34	80	54.076
RNH_3BPh_4	Phosphine oxide	Acetonitrile	147 ± 25	78	53.010
Et_3NHBPh_4	Phosphine oxide	Acetone	203 ± 50	80	54.076
Et_3NHBPh_4	Phosphine oxide	Acetonitrile	85 ± 6	66	53.671
Imidazolium$HBPh_4$	Phosphine oxide	Acetone	156 ± 18	80	54.396
Imidazolium$HBPh_4$	Phosphine oxide	Acetonitrile	102 ± 12	75	55.027
$NaBPh_4$	Phosphine oxide	Acetone	73 ± 5	71	50.579
$KBPh_4$	Phosphine oxide	Acetone	13 ± 3	51	49.249
$AgBF_4$	Phosphine oxide	Acetonitrile	8 ± 1	65	57.751
$AgBF_4$	Phosphine oxide	Acetone	966 ± 40	98	59.117
IPh_2PF_6	Phosphine oxide	Acetone	83 ± 12	72	52.346
IPh_2PF_6	Phosphine oxide	Acetonitrile	62 ± 3	67	52.517
$NaPF_6$	Phosphine oxide	Acetone	55 ± 13	68	52.530
$NaBF_4$	Phosphine oxide	Acetone	57 ± 2	69	49.525
$NaBF_4$	Phosphine oxide	Acetonitrile	57 ± 6	68	50.148
$NaBAr^F$	Phosphine oxide	Acetone	72 ± 2	71	50.632
$NaBAr^F$	Phosphine oxide	Acetonitrile	53 ± 3	64	50.819
$NaN(SO_2CF_3)_2$	Phosphine oxide	Acetone	78 ± 12	71	50.393
$NaN(SO_2CF_3)_2$	Phosphine oxide	Acetonitrile	56 ± 5	66	50.569
NaI	Phosphine oxide	Acetone	59 ± 6	70	50.606
NaI	Phosphine oxide	Acetonitrile	45 ± 1	60	50.677

Table S2. Association constants for the H-bonded complexes formed between **2** and **8-27** in the different solvents at 298 K. ^{a}Experimental results are the average of at least two titrations and errors are quoted at the 95% confidence limit.

23. Summary of ^{1}H NMR Spectroscopy Titration Experiments.

Hydrogen Bond Donor	Acceptor	Solvent	*K* (M^{-1})[a]	*% bound*	δ_{bound}
Phenol (α-CH)	Phosphine oxide	Carbon tetrachloride	1860 ± 226	78	7.126
5-nitroindole (NH)	Phosphine oxide	Carbon tetrachloride	1350 ± 480	82	12.171
4-phenylazophenol (α-CH)	Phosphine oxide	Carbon tetrachloride	14350 ± 2404	82	7.415
4-nitrophenol (α-CH)	Phosphine oxide	Carbon tetrachloride	142000 ± 26870	93	8.151

Table S3. Association constants for the H-bonded complexes formed between **2** and **3-6** in the different solvents at 298 K. [a]Experimental results are the average of at least two titrations and errors are quoted at the 95% confidence limit.

22. Influence of the Counterion in Acetonitrile on *K* values of H-bonded complexes

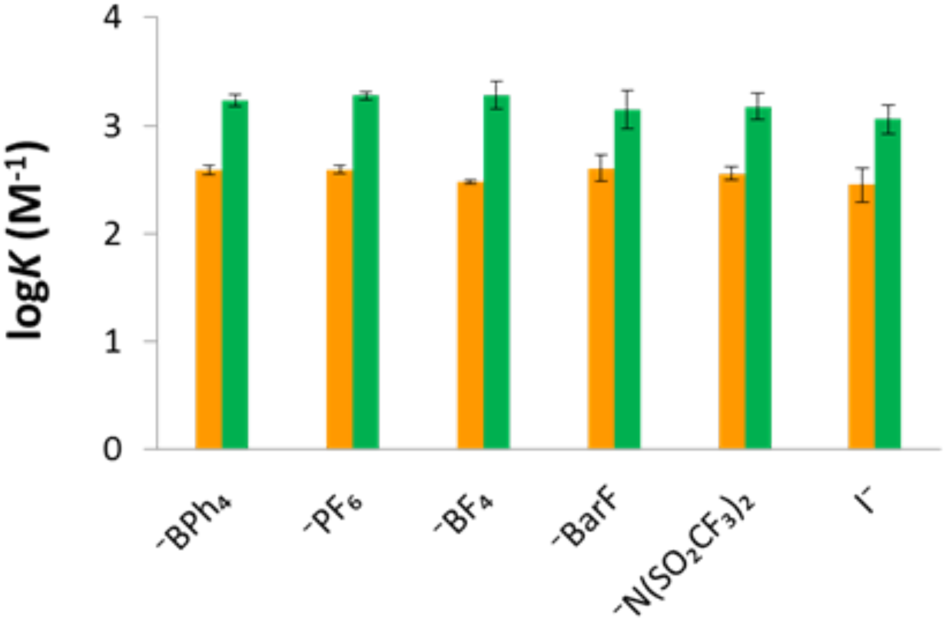

Graph S221. log*K* (M^{-1}) values for association constants of H-bonded complexes **1**·Na^+ in acetonitrile and acetone obtained using UV/Vis absorption titration experiments with different counter-anions. The association constants obtained in acetonitrile are shown in orange and those obtained in acetone are shown in green.

Graph S222. log$K$ (M^{-1}) values for association constants of H-bonded complexes **1**·Na^{+} in acetonitrile and acetone obtained using UV/Vis absorption titration experiments with different counter-anions. The association constants obtained in acetonitrile are shown in orange and those obtained in acetone are shown in green.

23. Influence of the H_2O content in Acetonitrile on *K* values of H-bonded complexes

a) Titrations of 1 with 4-phenylazophenol in the presence of H_2O

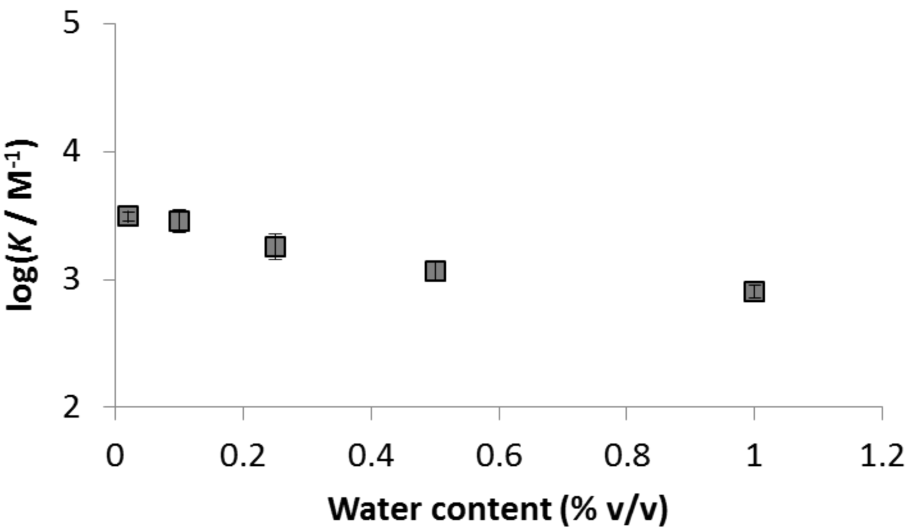

Graph S223. Graph showing correlation between % H_2O present in acetonitrile host solution and the log*K* value obtained from the titration of 4-phenylazophenol with **1** with varying amounts of H_2O added to the host solution.

b) Titrations of 1 with $NaN(SO_2CF_3)_2$ in the presence of H_2O

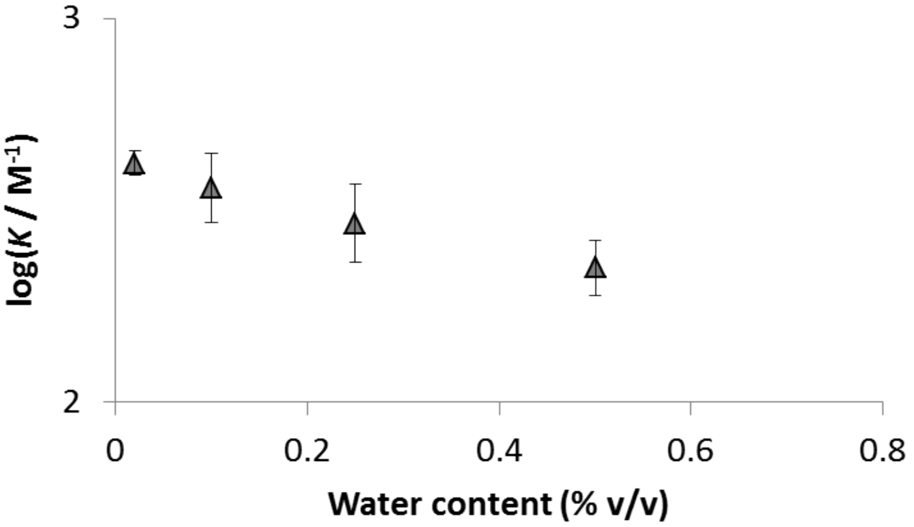

Graph S224. Graph showing correlation between % H_2O present in acetonitrile host solution and the *K* value obtained from the titration of $NaN(SO_2CF_3)_2$ with **1** with varying amounts of H_2O added to the host solution.

c) Titrations of 1 with guanidiniumBPh_4 in the presence of H_2O

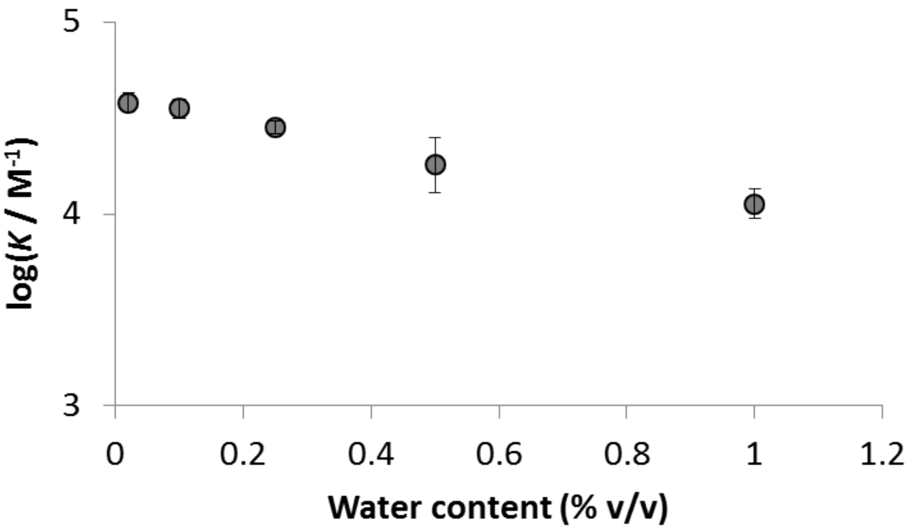

Graph S225. Graph showing correlation between % H_2O present in acetonitrile host solution and the *K* value obtained from the titration of guanidiniumBPh_4 with **1** with varying amounts of H_2O added to the host solution.

d) Titrations of 1 with 4-phenylazophenol, $NaN(SO_2CF_3)_2$ and guanidiniumBPh_4 in the presence of H_2O

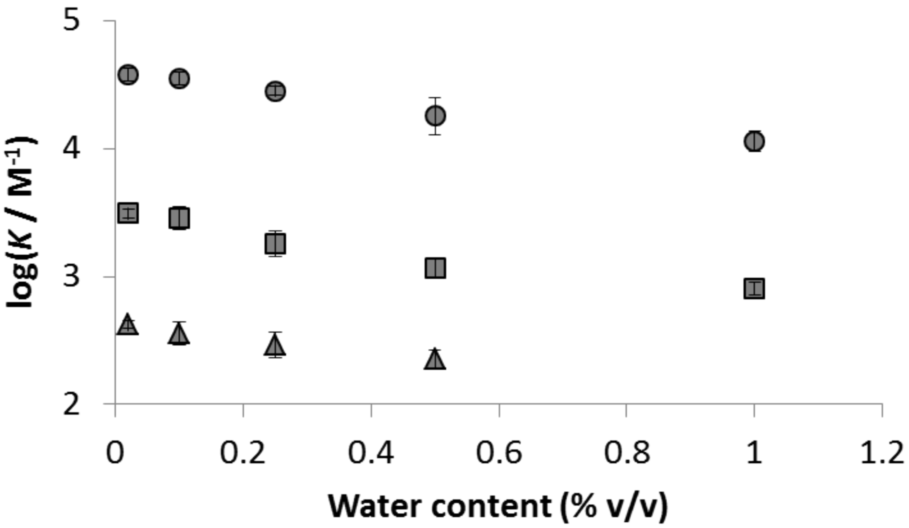

Graph S226. Graph showing correlation between % H_2O present in acetonitrile host solution and the *K* value obtained from the titration of 4-phenylazophenol, $NaN(SO_2CF_3)_2$ and guanidiniumBPh_4 with **1** with varying amounts of H_2O added to the host solution. **1·guandiniumBPh_4** complexes shown in circles, **1·4-phenylazophenol** complexes shown in squares, **1· $NaN(SO_2CF_3)_2$** complexes shown in triangles.

e) Summary of the influence of water on the *K* and *α* values for titrations of Reichardt's dye with Guanidinium BPh_4, $Na(NSO_2CF_3)_2$ and 4-phenylazophenol in acetonitrile

Hydrogen Bond Donor	H_2O Content in Stock Solution	*K* (M^{-1})[a]
4-phenylazophenol	0.02	3100 ± 650
4-phenylazophenol	0.1	2700 ± 560
4-phenylazophenol	0.25	1600 ± 380
4-phenylazophenol	0.5	1100 ± 180
4-phenylazophenol	1	800 ± 20
$Na(NSO_2CF_3)_2$	0.02	410 ± 30
$Na(NSO_2CF_3)_2$	0.1	380 ± 38
$Na(NSO_2CF_3)_2$	0.25	290 ± 18
$Na(NSO_2CF_3)_2$	0.5	250 ± 60
Guanidinium.BPh_4	0.02	33800 ± 2000
Guanidinium.BPh_4	0.1	34000 ± 3800
Guanidinium.BPh_4	0.25	27000 ± 2000
Guanidinium.BPh_4	0.5	12600 ± 4200
Guanidinium.BPh_4	1	11000 ± 2000

Table S2. Summary of *K* and *α* values for the H-bonded complexes involving **1** and Guanidinium BPh_4, $Na(NSO_2CF_3)_2$ and 4-phenylazophenol in acetonitrile with varying quantities of H_2O added to the stock solution.[a]Experimental results are the average of at least two titrations and errors are quoted at the 95% confidence limit.

24. Influence of the H_2O content in Acetone on *K* values of H-bonded complexes

a) Titrations of 1 with 4-phenylazophenol in the presence of H_2O

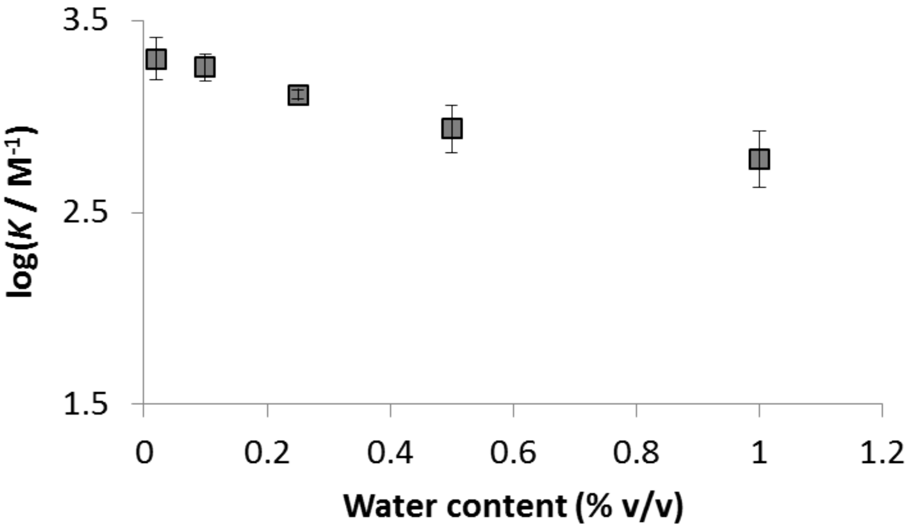

Graph S227. Graph showing correlation between % H_2O present in acetone host solution and the log*K* value obtained from the titration of 4-phenylazophenol with **1** with varying amounts of H_2O added to the host solution.

b) Titrations of 1 with $NaN(SO_2CF_3)_2$ in the presence of H_2O

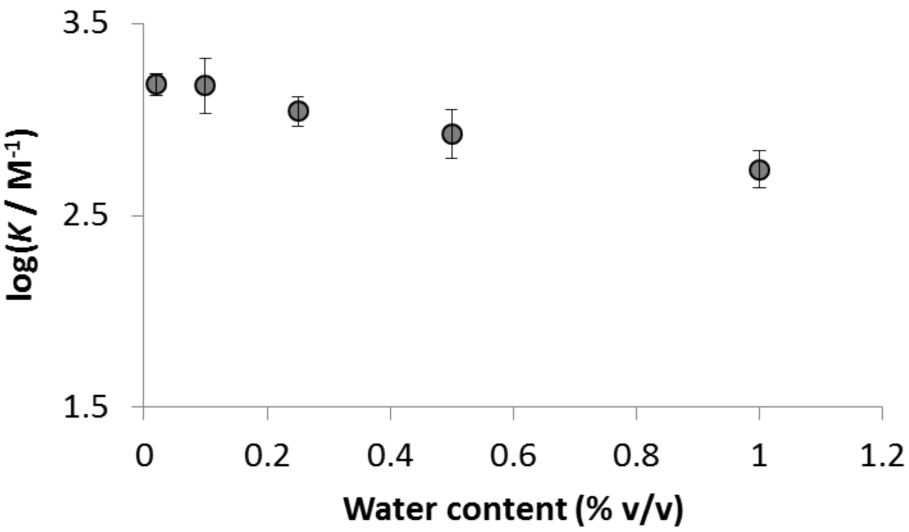

Graph S228. Graph showing correlation between % H_2O present in acetone host solution and the *K* value obtained from the titration of $NaN(SO_2CF_3)_2$ with **1** with varying amounts of H_2O added to the host solution.

c) Titrations of 1 with $KBPh_4$ in the presence of H_2O

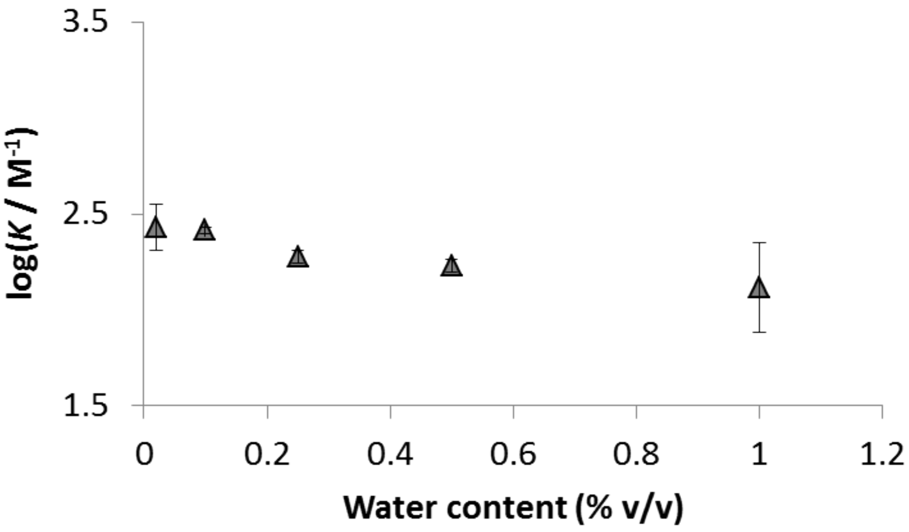

Graph S229. Graph showing correlation between % H_2O present in acetone host solution and the *K* value obtained from the titration of $KBPh_4$ with **1** with varying amounts of H_2O added to the host solution.

d) Titrations of 1 with 4-phenylazophenol, $NaN(SO_2CF_3)_2$ and guanidiniumBPh_4 in the presence of H_2O

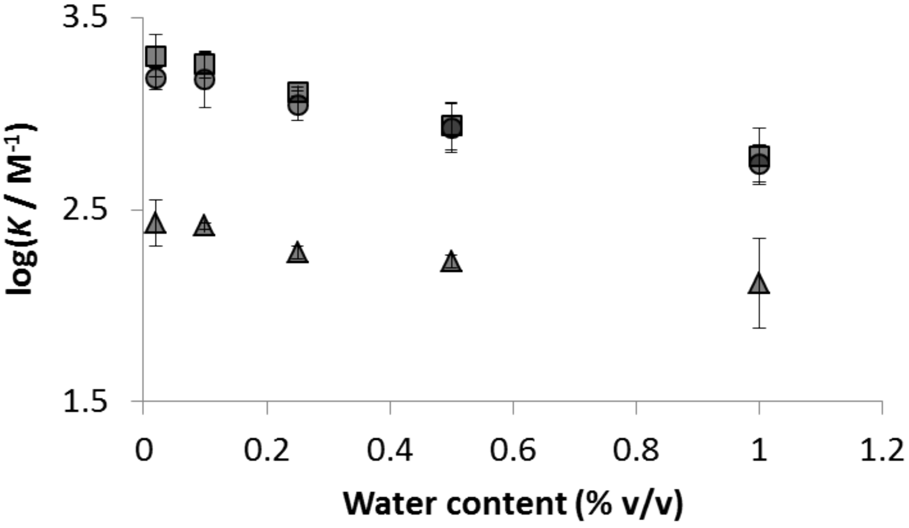

Graph S230. Graph showing correlation between % H_2O present in acetone host solution and the *K* value obtained from the titration of 4-phenylazophenol, $NaN(SO_2CF_3)_2$ and $KBPh_4$ with **1** with varying amounts of H_2O added to the host solution. **1·$NaN(SO_2CF_3)_2$** complexes shown in circles, **1·4-phenylazophenol** complexes shown in squares, **1·$KBPh_4$** complexes shown in triangles.

e) Summary of the influence of water on the *K* and *α* values for titrations of Reichardt's dye with 4-phenylazophenol, $Na(NSO_2CF_3)_2$ and $KBPh_4$ in acetone

Hydrogen Bond Donor	H_2O Content in Stock Solution	*K* (M^{-1})[a]
4-phenylazophenol	0.02	2000 ± 500
4-phenylazophenol	0.1	1800 ± 280
4-phenylazophenol	0.25	1300 ± 71
4-phenylazophenol	0.5	860 ± 240
4-phenylazophenol	1	600 ± 200
$Na(NSO_2CF_3)_2$	0.02	1500 ± 50
$Na(NSO_2CF_3)_2$	0.1	1500 ± 200
$Na(NSO_2CF_3)_2$	0.25	1100 ± 200
$Na(NSO_2CF_3)_2$	0.5	840 ± 280
$Na(NSO_2CF_3)_2$	1	550 ± 120
$KBPh_4$	0.02	270 ± 75
$KBPh_4$	0.1	260 ± 21
$KBPh_4$	0.25	190 ± 15
$KBPh_4$	0.5	170 ± 18
$KBPh_4$	1	130 ± 70

Table S3. Summary of *K* and *α* values for the H-bonded complexes involving **1** and 4-phenylazophenol, $Na(NSO_2CF_3)_2$ and $KBPh_4$ in acetone with varying quantities of H_2O added to the stock solution.[a]Experimental results are the average of at least two titrations and errors are quoted at the 95% confidence limit.

25. Appendix: ^{1}H and ^{13}C NMR spectra and mass spec

1H NMR spectrum of guanidinium.BPh_4 in CD_3CN

^{1}H NMR spectrum of imidazolium.BPh_4 in $(CD_3)_2SO$

^{1}H NMR spectrum of $Et_3NH^{+-}BPh_4$ in $(CD_3)_2SO$

^{1}H NMR spectrum of 2-ethylhexylammonium.BPh_4 in $(CD_3)_2CO$

^{13}C NMR spectrum of guanidinium.BPh_4 in CD_3CN

^{13}C NMR spectrum of imidazolium.BPh_4 in $(CD_3)_2SO$

^{13}C NMR spectrum of $Et_3NH^{+-}BPh_4$ in $(CD_3)_2SO$

^{13}C NMR spectrum of 2-ethylhexylammonium.BPh_4 in THF-d_8

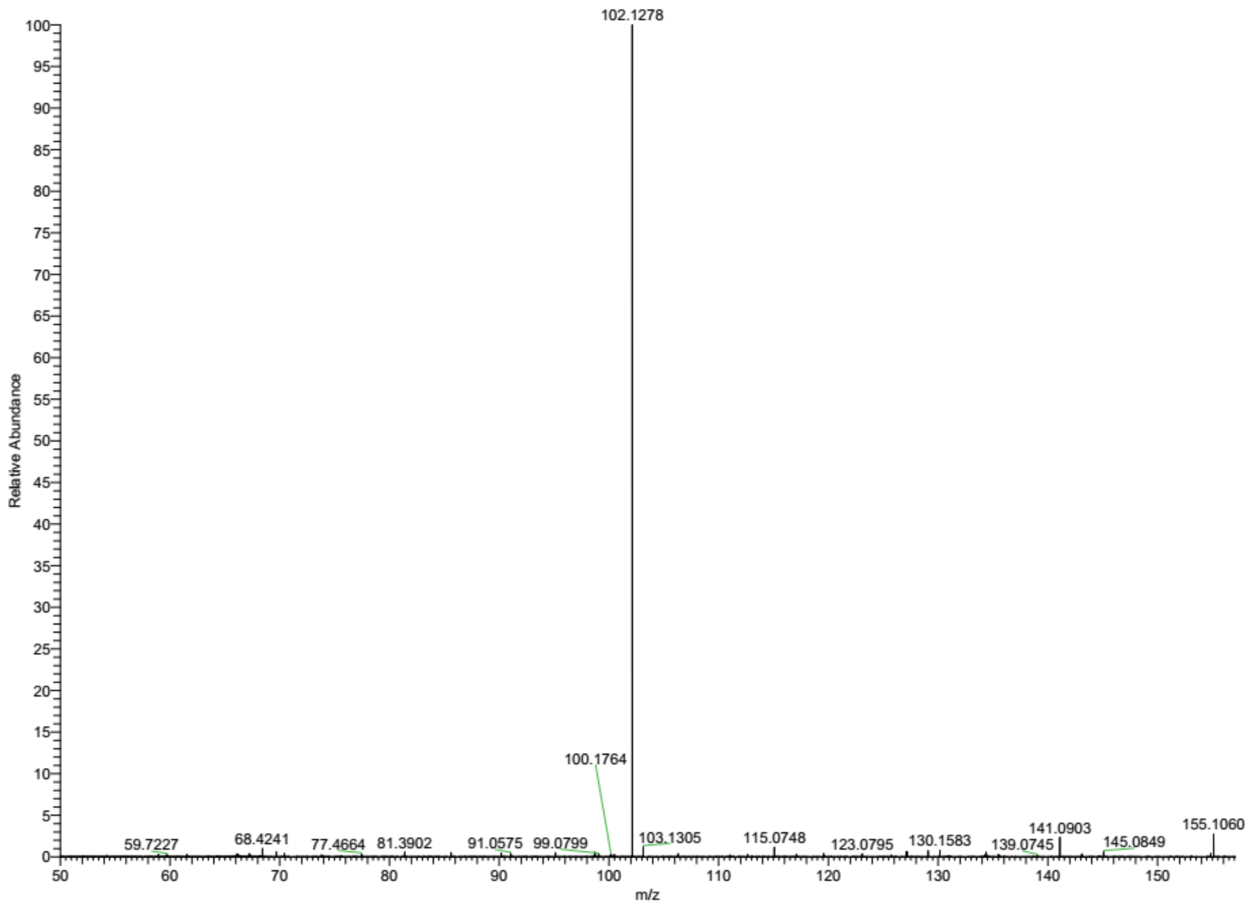

Mass spectrum of $Et_3NH^{+-}BPh_4$

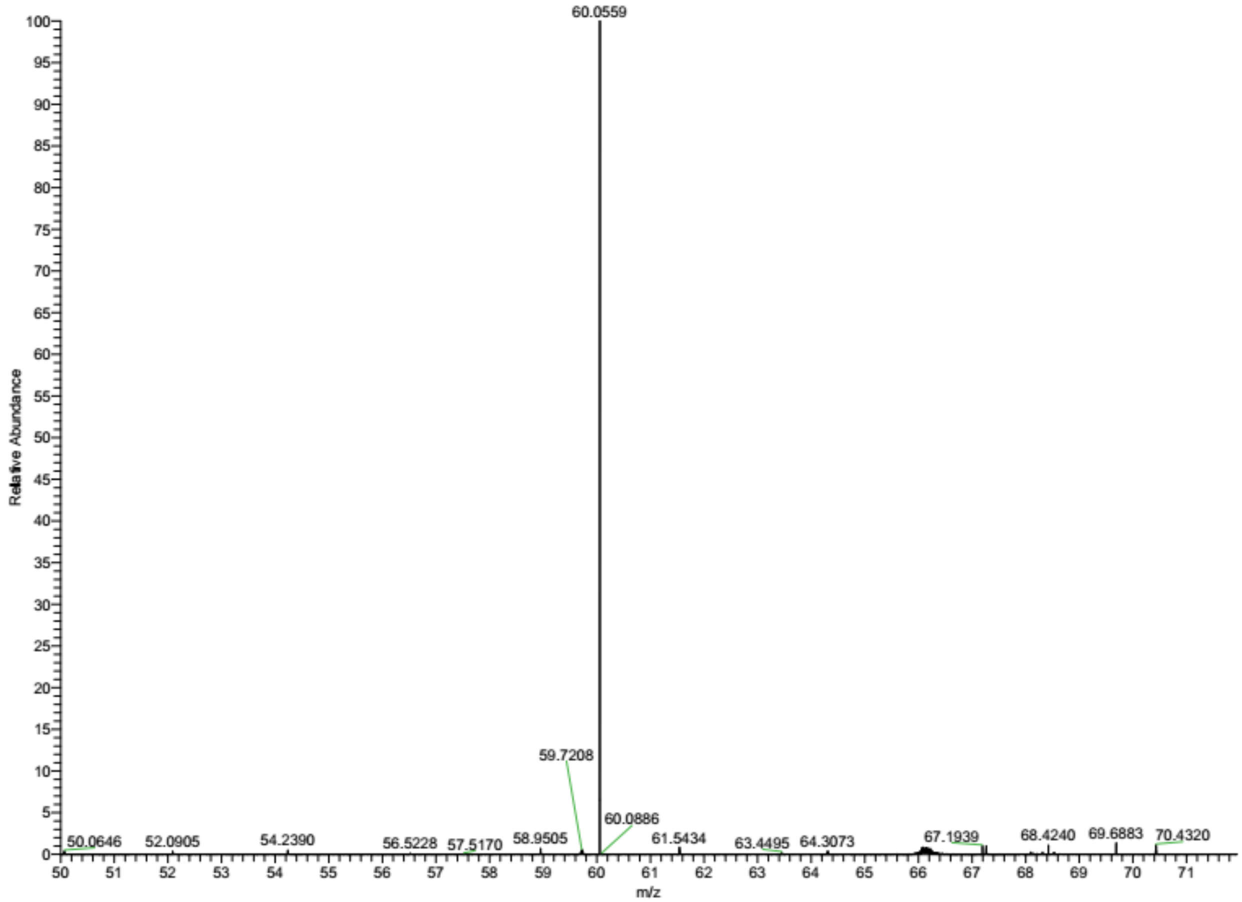

Mass spectrum of guanidinium.BPh$_4$

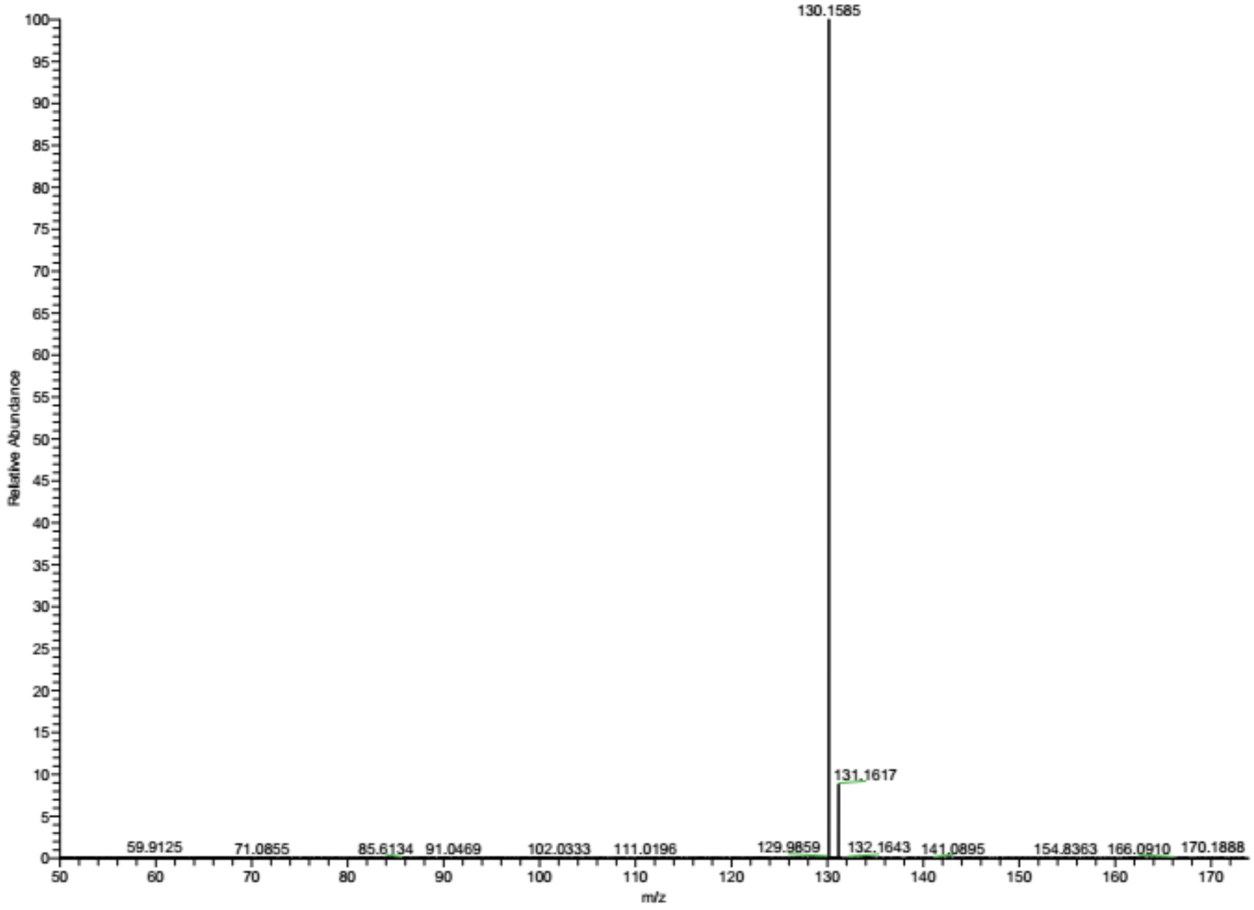

Mass spectrum of 2-ethylhexylammonium.BPh_4

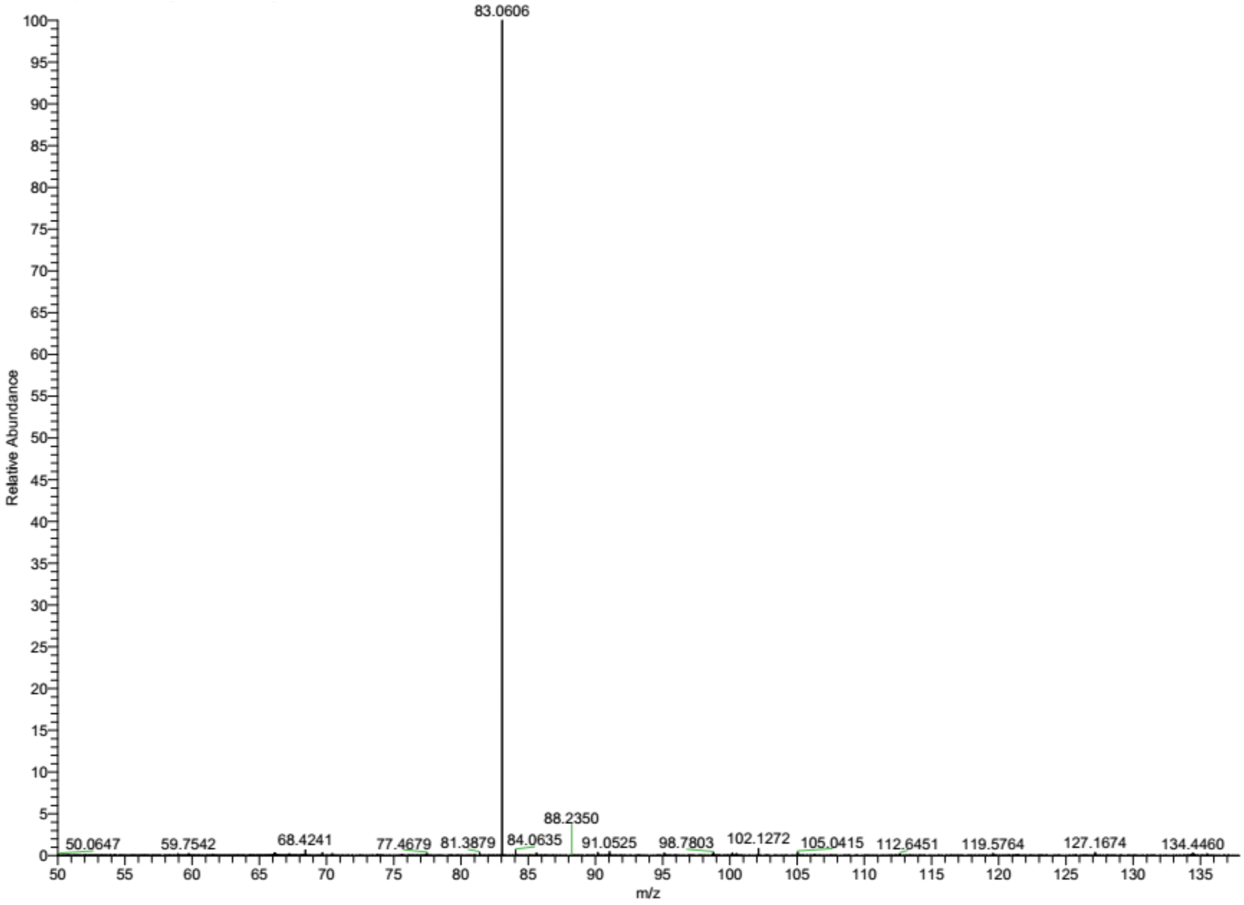

Mass spectrum of 1-methylimidazolium.BPh_4

32. References

1. V. Amenta, J. L. Cook, C. A. Hunter, C. M. R. Low, J. G. Vinter, *J. Phys. Chem. B.,* **2012**, *116*, 14443-14440.

2. V. Amenta, J. L. Cook, C. A. Hunter, C. M. R. Low, J. G. Vinter, *Org. Biomol. Chem.,* **2011**, *9*, 7571-7578.

3. S. J. Pike, J. J. Hutchinson, C. A. Hunter, *J. Am. Chem. Soc.,* **2017**, *139*, 6700-6706.

4. R. A. Cabot, C. A. Hunter, L. M. Varley, *Org. Biomol. Chem.,* **2010**, *8*, 1455-1462.

5. J. L. Cook, C. A. Hunter, C. M. R. Low, A. Perez-Velasco, J. G. Vinter, *Angew. Chem. Int. Ed.,* **2007**, *46*, 3706-3709.

6. J. McKenzie, N. Feeder, C. A. Hunter, *Cryst. Eng. Comm.,* **2016**, *18*, 394.

7. C. A. Hunter, *Angew. Chem. Int. Ed.,* **2004**, *43*, 5310-5324.
